# Single-cell atlas of transcriptomic vulnerability across brain disorders

**DOI:** 10.1038/s41586-025-09573-z

**Published:** 2026-09-23

**Authors:** Donghoon Lee, Mikaela Koutrouli, Nicolas Y. Masse, Gabriel E. Hoffman, Seon Kinrot, Xinyi Wang, Prashant N. M., Milos Pjanic, Tereza Clarence, Fotios Tsetsos, Deepika Mathur, David Burstein, Karen Therrien, Aram Hong, Clara Casey, Zhiping Shao, Marcela Alvia, Stathis Argyriou, Jennifer Monteiro Fortes, Sarah R. Murphy, Pavel Katsel, Pavan K. Auluck, Lisa L. Barnes, Stefano Marenco, David A. Bennett, PsychAD Consortium, Donghoon Lee, Mikaela Koutrouli, Nicolas Y. Masse, Gabriel E. Hoffman, Seon Kinrot, Xinyi Wang, Prashant N. M., Milos Pjanic, Tereza Clarence, Fotios Tsetsos, Deepika Mathur, David Burstein, Karen Therrien, Aram Hong, Clara Casey, Zhiping Shao, Marcela Alvia, Stathis Argyriou, Jennifer Monteiro Fortes, Sarah R. Murphy, Pavel Katsel, Pavan K. Auluck, Lisa L. Barnes, Stefano Marenco, David A. Bennett, Athan Z. Li, Biao Zeng, Chenfeng He, Chirag Gupta, Christian Dillard, Christian Porras, Colleen A. McClung, Collin Spencer, Daifeng Wang, Gennadi Ryan, Hui Yang, Jerome J. Choi, Kalpana H. Arachchilage, Lars J. Jensen, Logan C. Dumitrescu, Lyra Sheu, Madeline R. Scott, Marios Anyfantakis, Maxim Signaevsky, Monika Ahirwar, Noah Cohen Kalafut, Pengfei Dong, Pramod B. Chandrashekar, Rachel Bercovitch, Roman Kosoy, Sanan Venkatesh, Saniya Khullar, Sayali A. Alatkar, Steven Finkbeiner, Steven P. Kleopoulos, Timothy J. Hohman, Ting Jin, Vivek G. Ramaswamy, Xiang Huang, Zhenyi Wu, Kiran Girdhar, Georgios Voloudakis, Vahram Haroutunian, Jaroslav Bendl, John F. Fullard, Panos Roussos, Lars Juhl Jensen, Kiran Girdhar, Georgios Voloudakis, Vahram Haroutunian, Jaroslav Bendl, John F. Fullard, Panos Roussos

**Affiliations:** 1 Center for Disease Neurogenomics, Icahn School of Medicine at Mount Sinai, New York, NY USA; 2 Friedman Brain Institute, Icahn School of Medicine at Mount Sinai, New York, NY USA; 3 Department of Psychiatry, Icahn School of Medicine at Mount Sinai, New York, NY USA; 4 Department of Genetics and Genomic Sciences, Icahn School of Medicine at Mount Sinai, New York, NY USA; 5 Novo Nordisk Foundation Center for Protein Research, Faculty of Health and Medical Sciences, University of Copenhagen, Copenhagen, Denmark; 6 Center for Precision Medicine and Translational Therapeutics, James J. Peters VA Medical Center, New York, NY USA; 7 Mental Illness Research, Education and Clinical Center VISN2, James J. Peters VA Medical Center, New York, NY USA; 8 Human Brain Collection Core, National Institute of Mental Health-Intramural Research Program, Bethesda, MD USA; 9 Rush Alzheimer’s Disease Center, Rush University Medical Center, Chicago, IL USA; 10 Department of Neurological Sciences, Rush University Medical Center, Chicago, IL USA; 11 Department of Artificial Intelligence and Human Health, Icahn School of Medicine at Mount Sinai, New York, NY USA; 12 Department of Neuroscience, Icahn School of Medicine at Mount Sinai, New York, NY USA; 13 Department of Computer Sciences, University of Wisconsin-Madison, Madison, WI USA; 14 Waisman Center, University of Wisconsin-Madison, Madison, WI USA; 15 Department of Biostatistics and Medical Informatics, University of Wisconsin-Madison, Madison, WI USA; 16 Department of Psychiatry, University of Pittsburgh School of Medicine, Pittsburgh, PA USA; 17 Center for Systems and Therapeutics, Gladstone Institutes, San Francisco, CA USA; 18 Taube/Koret Center for Neurodegenerative Disease Research, Gladstone Institutes, San Francisco, CA USA; 19 Department of Population Health Sciences, University of Wisconsin-Madison, Madison, WI USA; 20 Vanderbilt Genetics Institute, Vanderbilt University Medical Center, Nashville, TN USA; 21 Vanderbilt Memory & Alzheimer’s Center, Vanderbilt University Medical Center, Nashville, TN USA; 22 Department of Neurology, University of California San Francisco, San Francisco, CA USA; 23 Department of Physiology, University of California San Francisco, San Francisco, CA USA; 24 Neuroscience and Biomedical Sciences Graduate Programs, University of California San Francisco, San Francisco, CA USA

**Keywords:** Alzheimer's disease, Parkinson's disease, Schizophrenia, Neurodegeneration, Bipolar disorder

## Abstract

Neurodegenerative and neuropsychiatric diseases impose a considerable societal and public health burden. However, our understanding of the molecular mechanisms underlying these highly complex conditions remains limited^1,2^. Here, to gain deeper insights into the aetiology of different brain diseases, we used specimens from 1,494 unique donors to generate a population-scale single-cell transcriptomic atlas of the human dorsolateral prefrontal cortex, comprising over 6.3 million individual nuclei. The cohort includes neurotypical controls, as well as donors affected by eight common and complex brain disorders: Alzheimer’s disease (AD), diffuse Lewy body disease (DLBD), vascular dementia (Vas), Parkinson’s disease (PD), tauopathy, frontotemporal dementia, schizophrenia, and bipolar disorder. We show that interindividual variation accounts for a substantial portion of gene expression variation. By comparing transcriptomic variation across diseases, we reveal universal signatures enriched in basic cellular functions such as mRNA processing and protein localization. After discounting these cross-disease signatures, we show stronger genetic and transcriptomic concordance among AD, DLBD, Vas and PD. Furthermore, we characterize transcriptomic variation among different AD phenotypes, distinct from those observed in healthy ageing, revealing a reduction in neuronal abundance in individuals with more severe AD, coupled with an increase in immune and vascular cell populations. Exploring the neuropsychiatric symptoms (NPSs) that frequently accompany AD, we find an increased abundance of deep-layer excitatory neurons associated with a broad range of NPSs. By constructing transcriptome trajectories that capture AD progression, we implicate cell-type-specific responses in the early and late stages of AD. Our disease atlas provides a perspective of the transcriptomic landscape in neurodegenerative and neuropsychiatric disorders, shedding light on shared and distinct processes involving the neurological–immune–vascular systems, and identifying potential targets for therapeutic intervention.

## Abstract (web summary)

A population-scale single-cell transcriptomic atlas of the human dorsolateral prefrontal cortex provides a perspective of the transcriptomic landscape in neurodegenerative and neuropsychiatric disorders, and offers potential targets for therapeutic intervention.

## Main

The human brain is a highly complex organ composed of billions of functionally diverse cells. Under pathogenic stress, cellular and molecular responses are often convoluted and contextual, so understanding their dysfunction in disease is challenging. Single-cell approaches that facilitate analysis of the molecular changes that occur within individual cells have been particularly helpful in understanding the interplay between the different cell types found within complex tissues, as well as the roles of those cells in various disease contexts. In AD, a thorough exposition of cellular heterogeneity in the brain has revealed that studying the coordinated interactions between neurons and glia, and the selective depletion of vulnerable inhibitory neuronal subtypes, is critical for understanding AD pathology^1,2^.

Building a large-scale disease atlas at the single-cell resolution creates an opportunity to understand molecular responses at the cellular level and estimate population-level variation in the brain transcriptome. A large sample size provides the power needed to establish robust basal-level conditions and to sufficiently capture the full spectrum of disease pathology. By leveraging cross-disease atlases, studies have revealed shared and distinct patterns of gene expression perturbations in major psychiatric diseases, as well as shared genetic factors leading to molecular convergence^3,4^. Thus, characterizing shared transcriptomic vulnerabilities and pathophysiology together has considerable implications for early treatment and the development of effective therapeutics.

Here we introduce the PsychAD cohort, which consists of 1,494 unique brain donors, including neurotypical controls and individuals affected by various neurodegenerative and neuropsychiatric diseases (NPDs). Focusing on the dorsolateral prefrontal cortex (DLPFC), we generated a single-nucleus RNA sequencing (snRNA-seq) dataset comprising over 6.3 million nuclei representing 27 distinct subclasses of cells. The dataset is sufficiently powered to identify molecular signatures from multiple traits while accounting for individual variation. To support interpretation and enable future analyses, we evaluated power and effect size variability across cell types (Methods and Supplementary Fig. 5a–g). We identify shared transcriptomic vulnerability, defined as overlapping patterns of gene expression changes across diseases, which point to common pathogenic mechanisms. These shared signatures provide insights into potential molecular mechanisms of early intervention that go beyond traditional disease boundaries. Furthermore, we find that shared transcriptomic vulnerability between disorders is concordant with genetic co-heritability, suggesting that common genetic risk factors partly drive shared gene expression changes. Deep phenotyping of AD trajectories using tau pathology and clinical dementia status suggests a potential link between immune and brain vasculature dysfunctions. In summary, our study provides a rich and comprehensive resource for exploring the cellular and molecular mechanisms of brain function and dysfunction across multiple neurodegenerative diseases and NPDs.

## The PsychAD cohort

**Fig. 1:**
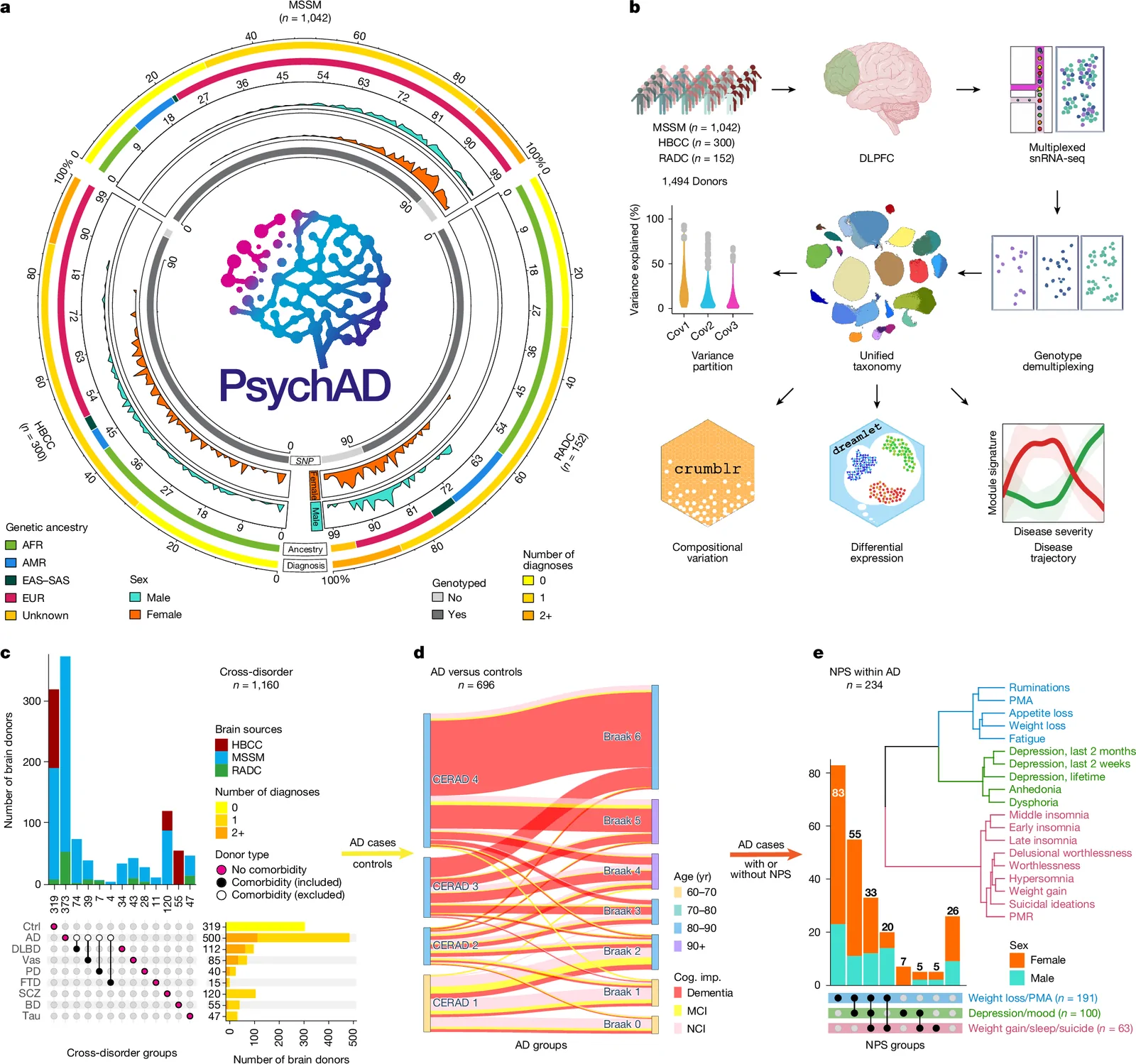
Overview of the PsychAD cohort and study design. **a**, The composition of the PsychAD cohort, comprising 1,494 donors from three tissue sources: MSSM (*n* = 1,042), HBCC (*n* = 300) and RADC (*n* = 152). Donor demographics, including genetic ancestry (AFR, African; AMR, ad mixed American; EAS, East Asian; SAS, South Asian; EUR, European; and unknown), sex distribution (male and female) and the percentage of available genotype data are shown. The number of diagnoses per donor is represented by colour-coded bars, distinguishing individuals with zero, one or multiple NDD and NPD diagnoses. Age at death is illustrated in radial histograms, showing the variability across tissue sources. The age distribution is binned and capped at 99+ years. **b**, Overview of the data generation and analytical workflow. DLPFC specimens from three tissue sources underwent multiplexed snRNA-seq. Libraries were demultiplexed by genotype and annotated using a unified cellular taxonomy. Subsequent analyses included variance partitioning to quantify the contribution of cell type and clinical covariates to gene expression variation, compositional variation using crumblr and differential expression analyses using dreamlet, and disease trajectory modelling to reveal cellular and transcriptional dynamics associated with disease progression. **c**, A subset of the PsychAD cohort (*n* = 1,160 donors) focused on cross-disorder contrasts. This subset includes a group of neurotypical control individuals (*n* = 319), and donors diagnosed with one of eight NDD and NPDs. The total number of brain donors per cross-disorder group is shown, stratified by brain sources (top) or by the number of diagnoses per donor (right). The UpSet plot (bottom left) illustrates unique and overlapping disorder phenotypes, highlighting patterns of comorbidity. For AD contrasts, comorbid cases were not considered. For non-AD contrasts, comorbid cases were included to ensure sufficient statistical power for the analysis. Owing to the inclusion of both comorbid and non-comorbid cases, there is an overlap in donor counts across disorder groups. As a result, the total number of donors represented in the plot may exceed the actual number of unique donors, reflecting instances in which the same donor appears in multiple diagnostic intersections. **d**, A subset of the PsychAD cohort (*n* = 696) focused on AD phenotype contrasts. Comparison of different measures of AD severity, including neuritic plaque density (CERAD), NFT pathology (Braak) and cognitive impairments (cog. imp.). Note that *n* = 54 donors with incomplete pathology or cognitive status were omitted from the visualization. **e**, A subset of the AD cases (*n* = 234) was used to characterize the co-occurrence of NPSs with AD. The columns represent the intersection of NPS categories and vertical bars indicate the number of donors in each intersection. The horizontal bars represent the total number of donors within each individual NPS category: depression and mood changes (Depression/mood; *n* = 100); weight loss and PMA (Weight loss/PMA; *n* = 191); and weight gain, sleep disturbances, suicidal ideation, guilt and psychomotor retardation (PMR) (Weight gain/sleep/suicide; *n* = 63); no NPSs represents AD donors without any recorded NPSs. Donor proportions are further stratified by sex.

The PsychAD cohort comprises 1,494 unique donors (Fig. 1a, Supplementary Fig. 2 and Supplementary Table 1). Brain tissue specimens were obtained from three sources: 1,042 donors from Mount Sinai NIH Neurobiobank (MSSM), 300 from Human Brain Collection Core (HBCC) and 152 from Rush Alzheimer’s Disease Center (RADC). The cohort covers the whole lifespan of postnatal ages between 0 and 108 years, roughly equal numbers of male (*n* = 723) and female (*n* = 771) individuals, and represents a diverse range of disease phenotypes, including AD, DLBD, Vas, tauopathy (Tau), frontotemporal dementia (FTD), PD, schizophrenia (SCZ) and bipolar disorder (BD). The cohort covers a diverse genetic background and over 30% of the donors (*n* = 509) were of non-European (EUR) ancestry.

We streamlined the unified processing of the data, as well as the harmonization of clinical and technical metadata (Fig. 1b, Supplementary Fig. 1a and Methods). Frozen brain specimens were randomized and processed in batches of six. Equal numbers of nuclei from each sample were pooled together and each pool was processed for two independent snRNA-seq reactions to generate a technical replicate. After quality control (Supplementary Fig. 1c–i), the final datasets consisted of 6,320,459 nuclei. To characterize transcriptomic vulnerability across multiple neuropsychiatric and neurodegenerative diseases (NDDs), we organized the analysis into three tiers.

First, we performed cross-disorder analyses (Fig. 1c). To ensure analytical power and interpretability, we focused on eight well-represented disorders with clear pathological definitions and sufficient donor representation, including balanced numbers of cases and controls. In detail, using a subset of 1,160 donors, at least 17 years of age, with minimal comorbidities, we targeted six NDDs (AD, DLBD, Vas, Tau, FTD and PD) and two NPDs (SCZ and BD). To estimate the sharing of transcriptomic vulnerability, we compared donors affected with NDDs and NPDs against the baseline of 319 neurotypical controls.

The second tier of analysis focused on the stage of AD progression based on pathological and cognitive impairment measures using a subset of 696 individuals (Fig. 1d). To identify cell-type-specific roles in disease onset and disease trajectory, we compared two characteristic neuropathological abnormalities of the AD brain: the accumulation of amyloid-β (Aβ) plaques, measured using the CERAD plaque density score^5^, and tau-based neurofibrillary tangle (NFT) pathology measured using Braak staging^6–8^, along with cognitive impairment.

In the third tier, we surveyed NPSs within 234 individuals with AD (Fig. 1e and Supplementary Fig. 1b). NPSs are core features of AD and are common in patients with dementia^9^. We broadly categorized NPSs into three groups—(1) depression or mood-related, (2) weight loss or psychomotor agitation (PMA), and (3) weight gain, insomnia or suicidal ideation—on the basis of co-occurrence estimates, sharing molecular mechanisms that lead to increased prevalence with disease severity.

## Unified cellular taxonomy of the human DPLFC

**Fig. 2:**
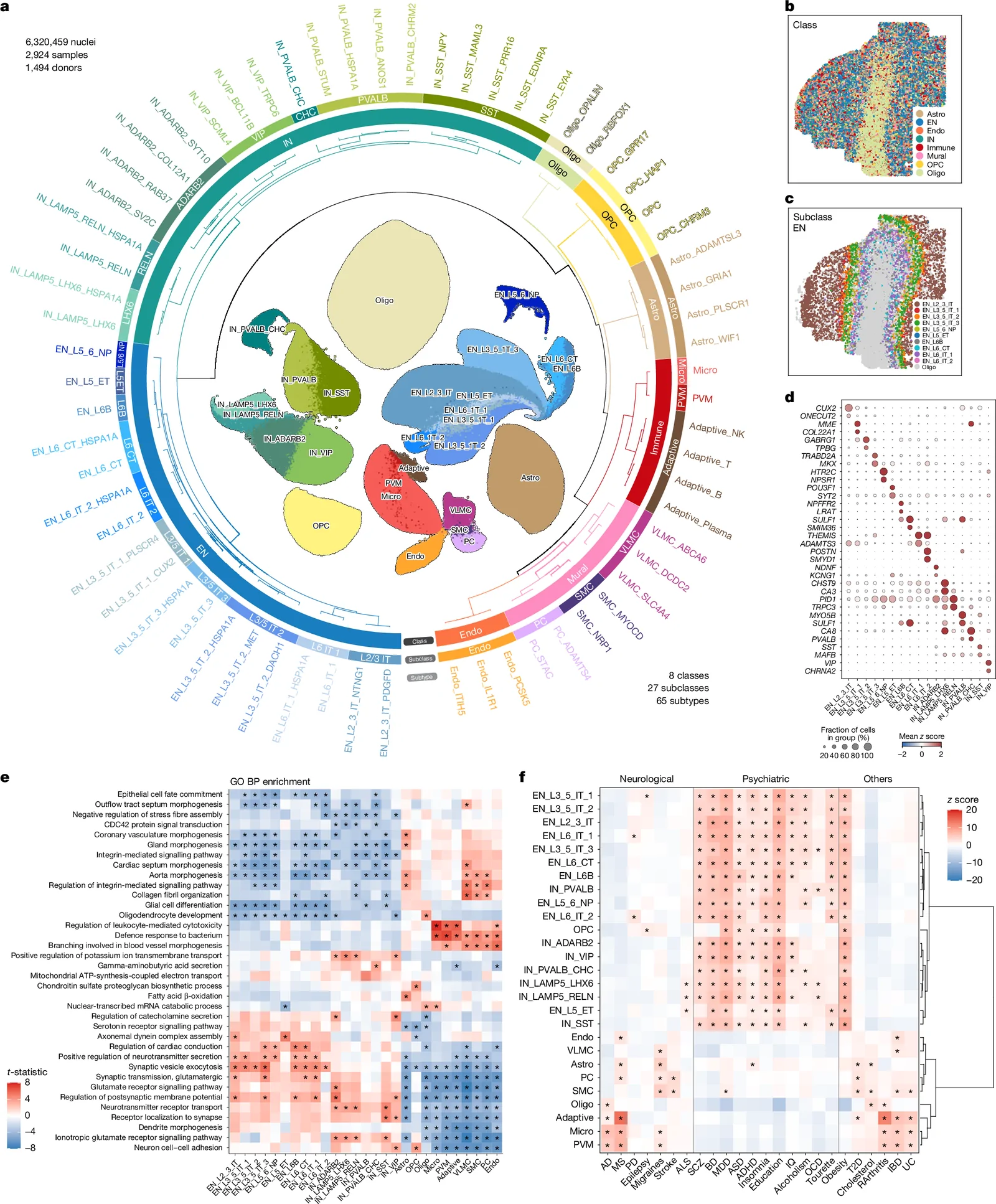
Unified processing of the single-nucleus transcriptomics atlas and hierarchical cellular taxonomy. **a**, Hierarchical structure of transcriptome-based cellular taxonomy. Taxonomic annotation at three levels of granularity: class (*n* = 8), subclass (*n* = 27) and subtype (*n* = 65). **b**, The spatial distribution of major cell classes. **c**, The spatial distribution of EN subclasses. **d**, Markers defining neuronal subclasses. **e**, Functional enrichment of cellular subclasses using GO BP. Cell-type-specific genes were selected for each subclass using differential gene expression analysis, comparing one versus the rest of subclass-level clusters, and filtering for genes with FDR ≤ 0.05. **f**, Enrichment of heritable traits for cellular subclasses using previous GWASs (scDRS). AD^96^, MS^97^, PD^98^, epilepsy focal (epilepsy)^99^, migraines^100^, stroke^101^, amyotrophic lateral sclerosis (ALS)^102^, SCZ^103^, BD^104^, major depressive disorder (MDD)^105^, autism spectrum disorder (ASD)^106^, attention deficit hyperactivity disorder (ADHD)^107^, insomnia^108^, educational attainment (education)^109^, intelligence (IQ)^110^, alcoholism^111^, obsessive–compulsive disorder (OCD)^112^, Tourette syndrome^113^, obesity^114^, type 2 diabetes mellitus (T2D)^115^, cholesterol total (cholesterol)^116^, rheumatoid arthritis (RArthritis)^117^, inflammatory bowel disease (IBD)^118^ and ulcerative colitis (UC)^119^.

To understand heterogeneous human cortical tissues in disease contexts, we require a cell type taxonomy that is robust to ageing, disease phenotypes and various sampling and technical biases. After unified computational processing, quality control and batch normalization of snRNA-seq libraries representing 1,494 dissections, processed in duplicate (Methods), we annotated cell types of the human DLPFC using the cell taxonomy of the primate DLPFC^10^ and human primary motor cortex^11^ as baseline references. The resulting taxonomy was organized into three hierarchical levels, identifying eight broad cell classes, 27 subclasses and 65 functionally distinct subtypes (Fig. 2a and Supplementary Table 2). Each level of the annotation hierarchy represents a slice in the clustering dendrogram. At the top, the class level defines eight major cell types, including two broad neuronal cell types: glutamatergic excitatory (EN) and GABAergic inhibitory neurons (IN); three glial: astrocytes (Astro), oligodendrocytes (Oligo) and oligodendrocyte progenitor cells (OPCs); and three non-neuronal cell types: immune cells (Immune), mural and vascular cells (Mural) and endothelial cells (Endo). Subsequent levels, namely subclass and subtype, were derived by iteratively reclustering the subset of cells by gene matrix using a new set of variable genes relevant to the particular cell type (see the ‘Defining cellular taxonomy using iterative clustering’ section of the Methods). The cellular taxonomy was consistent and well represented across all three brain sources (Supplementary Fig. 3a,b). Our most granular-level subtype annotation was robust and invariant to donor and technical variables (Supplementary Fig. 3c). Neuronal cells made up 38.4% (EN 23.0% and IN 15.4%) of all cells, with oligodendrocytes being the next most abundant, at 36.1%. Major cell types and subtypes matched well when compared to previous DLPFC cellular taxonomies^1,10^ (Supplementary Fig. 3d–f and Supplementary Table 11).

The subclass level distinguished the EN and IN classes into 10 and 7 subclasses, respectively (Supplementary Fig. 3g,h). Different types of neurons, especially ENs, are organized into six horizontal layers (L1–L6) that are distinct in both cytoarchitecture and function^12^. In situ spatial transcriptomics data were used to confirm that the EN subclasses were spatially distinct and found in their respective neocortical layers (Fig. 2b,c and Supplementary Fig. 4j). The EN subclasses were denoted by their laminar organization (L2–6) and axon projection characteristics (intratelencephalic (IT), extratelencephalic (ET), near projecting (NP), corticothalamic (CT) and L6B). IN subclasses were determined using their characteristic marker genes (Fig. 2d and Supplementary Fig. 3i); *LAMP5*^+^*LHX6*^+^ ivy cells (IN_LAMP5_LHX6), *LAMP5*^+^*RELN*^+^ neurogliaform cells (IN_LAMP5_RELN), parvalbumin^+^ basket cells (IN_PVALB), parvalbumin^+^ chandelier cells (IN_PVALB_CHC), somatostatin^+^ Martinotti and non-Martinotti cells (IN_SST), *VIP*^+^ inhibitory neurons (IN_VIP) and *ADARB2*^+^ homologues of mouse *Sncg*^*+*^ inhibitory neurons^10^ (IN_ADARB2). Consistent with previous reports^10,13,14^, IN subclasses did not display the same degree of laminar organization as EN subclasses (Supplementary Fig. 4i). A more careful examination of the spatial density of nuclei from each subclass within coarsely defined laminar regions (Methods) revealed that IN distribution is not uniform across cortical layers. For example, we observed a preference for the *ADARB2*^+^ and *VIP*^+^ interneurons for outer cortical layers, while both *PVALB*^+^ interneuron subclasses were distributed across layers L2–6 with less prominent bias (Supplementary Fig. 4e,f). Notably, we observed *SST*^+^ inhibitory neuron subtypes had distinct layer-specific enrichment patterns (Supplementary Fig. 4f,k). Some previously annotated rare inhibitory neuron types, such as *SST*^+^*NPY*^+^ or *SST*^+^*HGF*^+^, were not distinguished at the subclass level, but were identified at the subtype level.

As expected, neuronal subclasses exhibit distinct functional characteristics from those of non-neurons (Fig. 2e). ENs are enriched in pathways associated with synaptic vesicle priming and neurotransmitter secretion, whereas INs are enriched with functions related to receptor signalling and ion transport. Immune cell types show unique enrichments in functions related to cytotoxic immune responses, while mural cells are involved in vessel morphogenesis. It has been shown that cell types are differentially implicated in mediating disease risk^15,16^. We therefore further annotated cell types with disease genome-wide association studies (GWAS) using single-cell disease-relevance score (scDRS) and found that neurological diseases, including NDDs such as AD and neuroimmune diseases such as MS, largely involve immune and glial cell types, whereas psychiatric diseases are predominantly associated with neurons (Fig. 2f and Supplementary Fig. 3j).

## Sources of transcriptomic variation

**Fig. 3:**
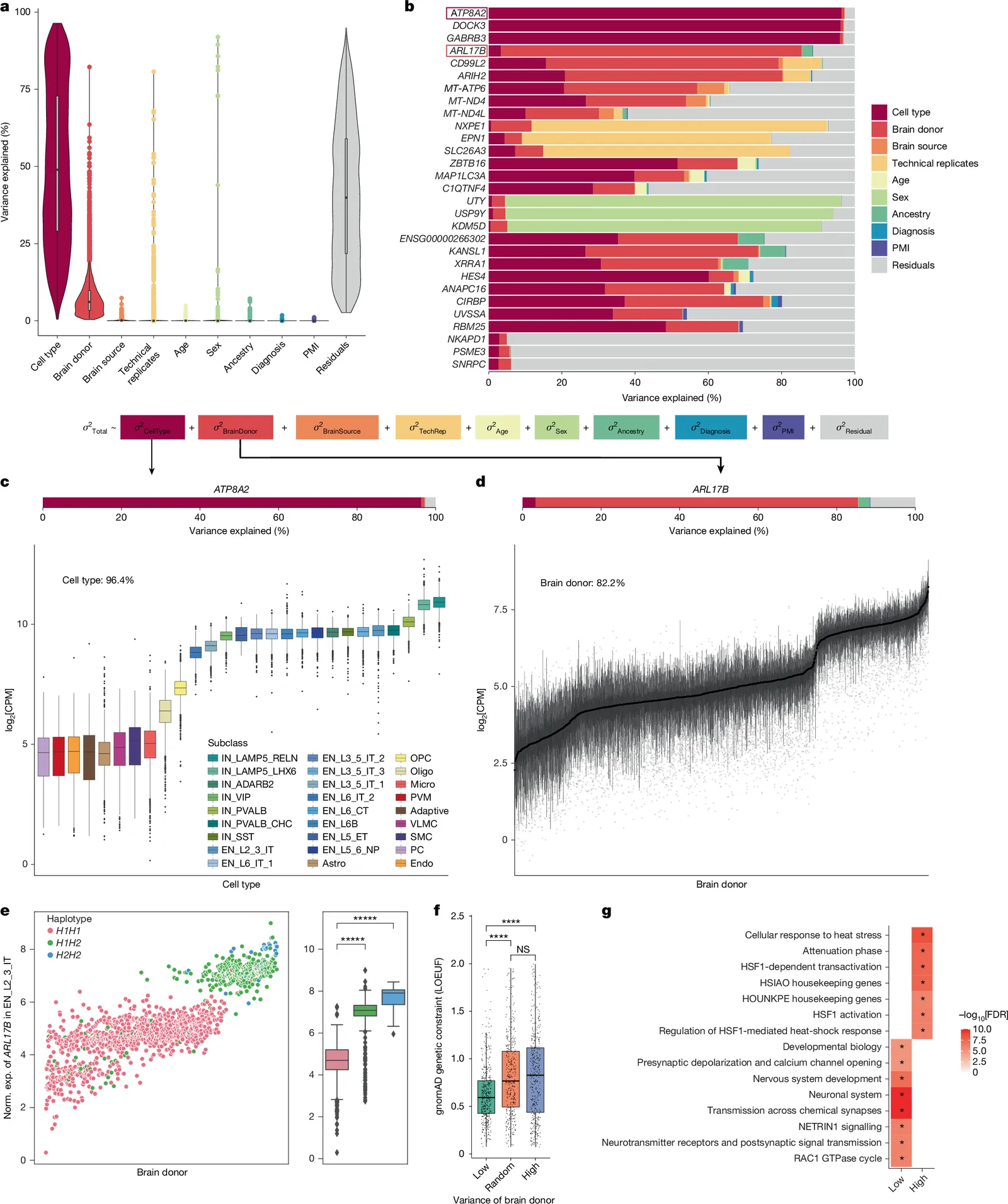
Sources of transcriptomic variation. **a**, Partition of transcriptomic variation by technical and clinical covariates. *n* = 34,890 genes examined over 2,924 libraries with 1,494 donors across 27 subclasses. *n* = 9,256 protein-coding genes are visualized. For the box plots, the box limits indicate the first and third quartiles, the centre line represents the median, and the whiskers extend to the minimum and maximum values. **b**, The top genes for each technical and clinical covariate category. **c**, *ATP8A2* gene expression across cell types. Subclass-level cell types were sorted on the basis of median expression levels. Expression levels were depth normalized (norm. exp.) using counts per million (CPM) and then log_2_ transformed. *n* = 1,494 donors examined across 27 subclasses. For the box plots, the box limits indicate the first and third quartiles, the centre line represents the median and the whiskers extend to 1.5× the interquartile range. The points beyond the whiskers were considered outliers. **d**, *ARL17B* gene expression across donors. Donors were sorted on the basis of median expression. **e**, Stratification of *MAPT* haplotypes using normalized expression of *ARL17B* in EN_L2_3_IT neurons. Statistical analysis was performed using Wilcoxon rank-sum tests between haplotypes using two-sided as the alternative hypothesis. *****P ≤ 1 × 10^−5^. The exact *P* value between *H1H1* and *H1H2* was 1.78 × 10^−116^; and, between *H1H1* and *H2H2*, 2.41 × 10^−19^. *n* = 1,380 donors with genotypes were examined. For the box plots, the box limits indicate the first and third quartiles, the centre line represents the median, and the whiskers extend to 1.5× the interquartile range. The points beyond the whiskers were considered outliers. **f**, Genetic constraints of gene groups measured by LOEUF score (upper bound of 90% confidence interval for observed/expected ratio for high-confidence predicted loss-of-function (pLoF) variants; lower values indicate higher genetic constraints). ‘Low’ indicates genes in the bottom 5% quantile of the brain donor variance, and ‘high’ indicates genes in the top 5%. ‘Random’ indicates a random 5% of genes that are in neither the low nor the high category. Statistical analysis was performed using Wilcoxon rank-sum tests with *n* = 8,603 protein-coding genes examined. *n* = 1,247 genes visualized. For the box plots, the box limits indicate the first and third quartiles, the centre line represents the median, and the whiskers extend to 1.5× the interquartile range. The points beyond the whiskers were considered outliers. **g**, Functional enrichment for genes with high or low interindividual variation. SRP, signal-recognition particle; HSIAO_HOUSEKEEPING_GENES, housekeeping genes identified as expressed across 19 normal tissues^120^; HOUNKPE_HOUSEKEEPING_GENES, 1,130 human and mouse housekeeping genes^121^. *P ≤ 0.05, **P ≤ 0.01, ***P ≤ 0.001, ****P ≤ 0.0001.

Transcriptomic variation is influenced by genetic differences among individuals, phenotypes and technical factors. To understand the determinants of the overall transcriptome variation in our cohort, we used population-scale snRNA-seq data to partition gene expression variance by cellular variables (including cell type and fraction of mitochondrial and ribosomal genes), donor-level variables (including individual ID, age, sex, genetic ancestry and diagnosis) and technical variables (including source of the tissue specimen, post-mortem interval, technical replicates and sequencing depth) (Fig. 3a and Methods). Across all genes, a mean of 50.5% of the total expression variance can be attributed to cell type variation, while interindividual variation explains 7.5% and unexplained residual variation accounts for 41.1%. The remaining variables explained, on average, less than 1% of the total variance. We prioritized genes with the highest expression variation across variables (Fig. 3b and Supplementary Fig. 6b–f). Among the genes with high cell type variation (including *ATP8A2*, *DOCK3* and *GABRB3*), most are differentially expressed between neuronal and non-neuronal cell types (Fig. 3c). As expected, genes varying across sexes were located in sex chromosomes (Supplementary Fig. 6d), while those with high variation across tissue sources were mostly mitochondrial genes (Supplementary Fig. 6e), possibly due to technical differences in physiological and environmental factors from dissection and handling at the respective tissue sources^17,18^. Genes with high variation across diagnosis were implicated in stress-response and housekeeping functions (Supplementary Fig. 6a,f). The top gene, cold-inducible RNA-binding protein (*CIRBP*), responds to hypothermic stress and protects cells from hypoxia-induced neurotoxicity and its expression has been shown to be inversely associated with patient survival in cancer^19–21^. Note that, in addition to its variation across diagnoses, *CIRBP* also had the highest variation with PMI. As this approach examines expression variation shared across cell types, the low fraction of variance explained by diagnosis indicates the need for cell-type-specific analysis.

Notably, we observed that interindividual differences explained 82.2% of the variation in expression of the ADP ribosylation factor-like GTPase 17B (*ARL17B*) gene (Fig. 3d). Several adjacent genes, such as KAT8 regulatory NSL complex subunit 1 (*KANSL1*) and *ARL17A* (a paralogue of *ARL17B*), also had high interindividual variation (Supplementary Fig. 6c). *ARL17B* and *KANSL1* are localized in the disease-associated *MAPT* locus (17q21.31), are often found as a fusion transcript (*KANSL1*::*ARL17B*), frequently undergo polymorphic translocation^22,23^ and have been implicated in neurological disorders, including ALS, PD and MS^24–27^. To examine possible genetic causes for variation in expression, we examined normalized gene expression at the donor level, stratified by *MAPT* haplotypes (Fig. 3e and Methods). We observed two distinct patterns of *ARL17B* expression that could be potentially linked to *H1* and *H2 MAPT* haplotypes, with lower expression associated with the *H1H1* genotype. We observed stratification of haplotypes by genetic ancestry, where the *H2H2* genotype was almost exclusively found within EUR ancestry, consistent with previous reports^28,29^ (Supplementary Fig. 6g). We also replicated a previous finding indicating that the *H1* haplotype is associated with PD susceptibility, and observed that the *H1H1* genotype increases PD risk with an odds ratio (OR) of 4.125 (Supplementary Fig. 6h; *P* ≤ 0.0273), albeit much higher than previously reported (1.42 (ref. ^30^) or 1.46 (ref. ^31^). Moreover, we tested the contribution of the *H1* haplotype to AD among non-*APOE4* carriers^32^ but did not find a significant association (*P* ≤ 0.302). The *H1* haplotype exhibited *cis*-regulatory effects on several genes near the *MAPT* locus that are relevant to neurodegeneration^33^ (Supplementary Fig. 6i). Specifically, *H1* haplotypes in neurons and astrocytes were associated with increased expression of leucine rich repeat containing 37A genes (*LRRC37A2/A3*). LRRC37A proteins have been shown to interact with α-synuclein and co-localize with Lewy bodies in PD brains^34^. Another gene upregulated by the *H1* haplotype was pleckstrin homology domain-containing family M member 1 (*PLEKHM1*), which has been linked to PD susceptibility by GWAS^35,36^. The *H1* haplotype also demonstrated interchromosomal effects, notably on phosphodiesterase 8B (*PDE8B*), a gene implicated in movement disorders^37–40^. Furthermore, heterogeneous nuclear ribonucleoprotein K (*HNRNPK*), a gene involved in synaptic plasticity regulation^41^, was downregulated by the *H1* haplotype.

Genes with lower interindividual variation had higher genetic constraints, as measured by the gnomAD^42^ loss-of-function observed/expected upper-bound fraction (LOEUF) score or GeneBayes^43^ constraint metric (*S*_het_) (Fig. 3f and Supplementary Fig. 6l). These conserved genes were functionally implicated in nervous system development and synaptic functions, indicating that the gene program in neuronal development is conserved across individuals (Supplementary Fig. 6m). On the other hand, genes with high interindividual variability were enriched with basal cellular functions (that is, housekeeping genes, RNA metabolism and, in particular, cellular response to heat stress), corroborating previous findings^44,45^ (Fig. 3g and Supplementary Fig. 6a,m).

## Cross-disorder variation of cell type composition

**Fig. 4:**
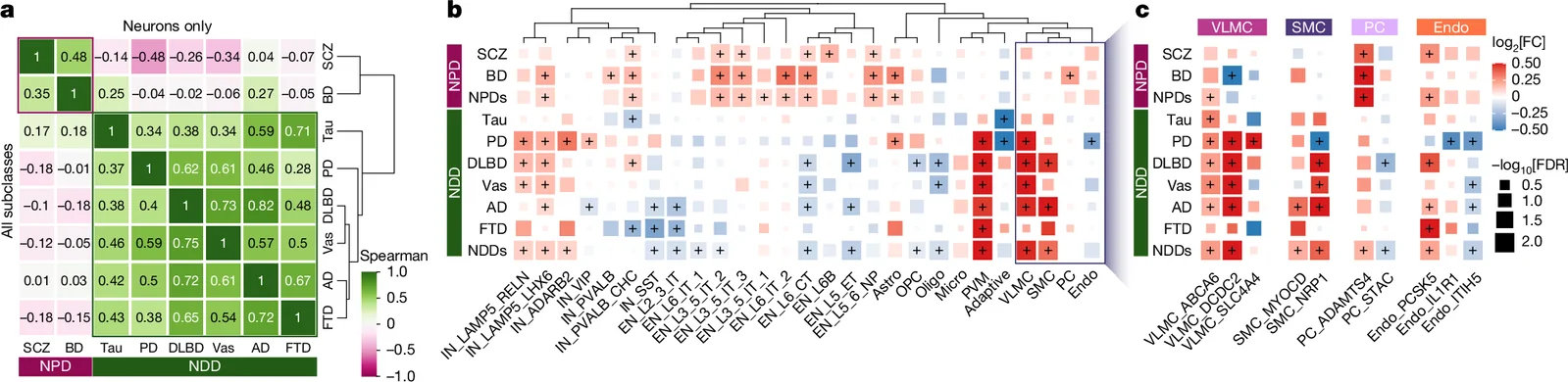
Cross-disorder variation of cell type composition comparing NDDs and NPDs to neurotypical controls. **a**, Pairwise Spearman’s correlations of cell type proportions across donors for each disorder. Hierarchical clustering based on neuronal composition highlights separation between NPDs and NDDs. Correlations using all cell subclasses (below the diagonal) or neuronal cell types only (above the diagonal). **b**, log_2_-transformed fold changes in the variation of each subclass (columns) relative to the controls across the eight disorders (rows). NPD and NDD labels reflect group-level summaries across multiple conditions. **c**, Variation of subtype-level composition in the vascular cell class. The colour intensity indicates the effect size and the dot size reflects the statistical significance of correlations.

Using the 318 neurotypical donors as a baseline, we systematically evaluated variation in the cellular composition of the DLPFC across eight different NDDs (AD, DLBD, Vas, Tau, PD and FTD) and NPDs (SCZ and BD). Using all subclass-level cell types, we found that the overall cell type composition changes were broadly stratified by NDDs and NPDs, and that they formed distinct clusters (Fig. 4a). On average, correlations were greater when focusing solely on neurons, underscoring their critical role in the aetiology of neurological diseases. Notably, stronger similarities were observed between DLBD and both AD and Vas. Moreover, similarities between FTD–AD (all cells) and FTD–Tau (neurons) were observed. Exploring each subclass further, we identified a notable overlap in the prevalence of neuronal and glial cell types within the same class of diseases (Fig. 4b and Supplementary Table 3). Specifically, NDDs were characterized by a higher abundance of non-neuronal cells, particularly VLMCs and other vascular cell types (Supplementary Fig. 7c,e,f), as well as elevation of specific inhibitory neuron subclasses, namely IN_LAMP5_RELN, IN_LAMP5_LHX6 and IN_ADARB2. By contrast, NPDs were predominantly associated with an increase in neuronal cells, particularly deep-layer ENs in L5–6. To further identify specific subtypes responsible for driving the compositional changes in vascular cell types, we used subtype-level annotation to analyse compositional variation in 8 NDDs and NPDs (Fig. 4c and Supplementary Fig. 7d). From this, we identified dominant subtypes of each subclass that further differentiated NDDs and NPDs. For example, vascular leptomeningeal cells (VLMCs) are barrier-forming fibroblasts of the brain^46^, and they are transcriptionally segregated into three subtypes; two meningeal VLMCs (VLMC_DCDC2 and VLMC_SLC4A4) and one perivascular VLMC (VLMC_ABCA6). Our subtype-level analysis indicates a polarized response of meningeal VLMC_DCDC2, where their increased proportions are specifically associated with most NDDs.

## Cross-disorder variation of gene expression

**Fig. 5:**
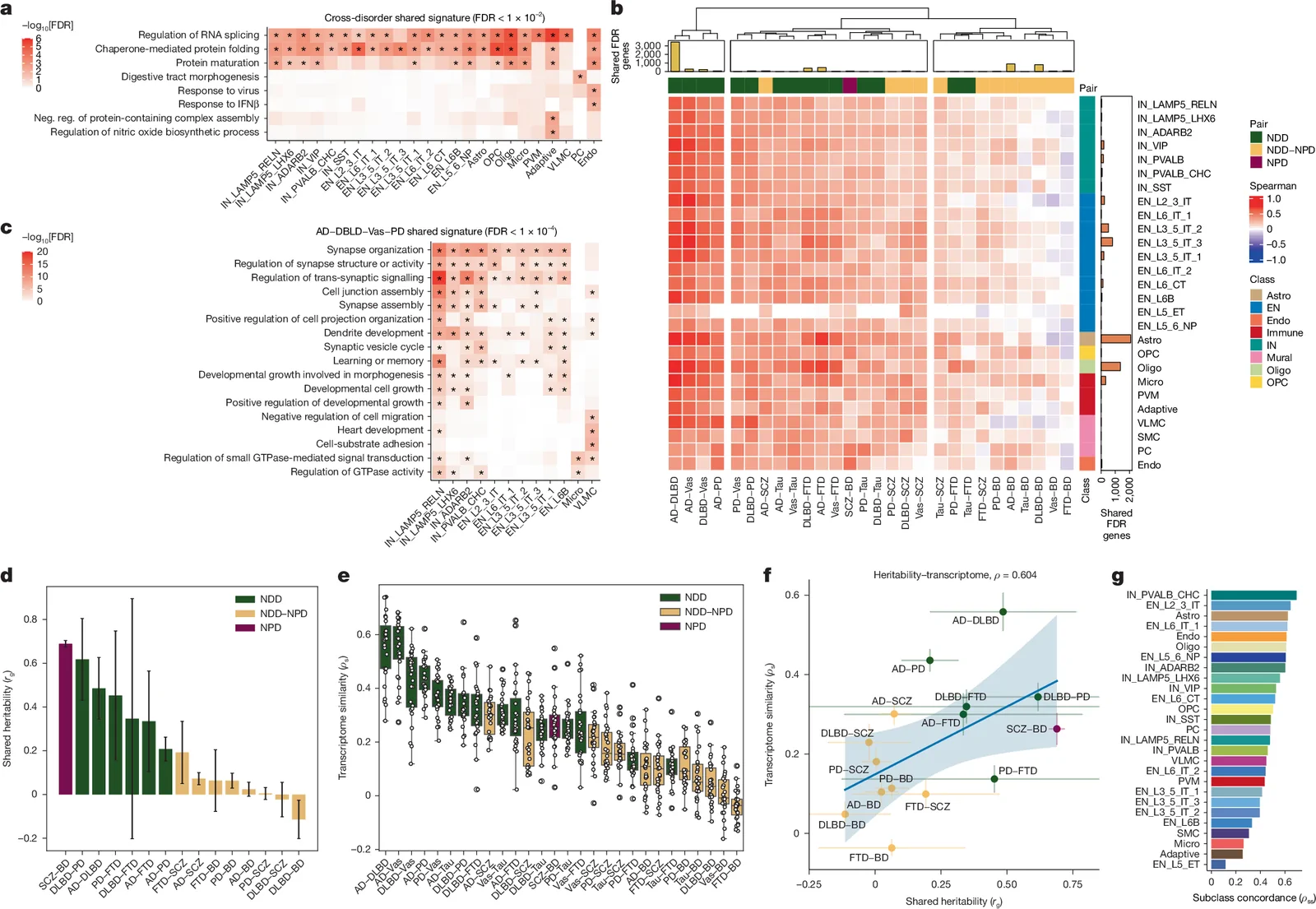
Cross-disorder variation of gene expression. **a**, Pathways implicated by shared gene expression changes across eight brain disorders. Hypergeometric test with FDR ≤ 0.01 is shown. Neg. reg., negative regulation. **b**, The transcriptome similarities between disease pairs as measured by Spearman’s correlation. Cross-disease shared genes are discounted from the comparison. **c**, The pathways implicated by shared signatures from AD, DLBD, Vas and PD. Hypergeometric test with FDR ≤ 0.001 is shown. **d**, Estimated shared heritability between disease pairs using LD score regression (LDSC). Data are *r*_g_ ± s.e. from LDSC estimation. *n* = 1,290,028 SNPs examined from the reference panel. **e**, The transcriptome similarity as measured using Spearman’s correlation. Each dot represents the correlation between two traits within a specific subclass. The box plot summarizes the distribution of these correlations across all 27 subclasses. *n* = 756 co-expression examined (28 trait pairs by 27 subclasses). For the box plots, the box limits indicate the first and third quartiles, the centre line represents the median, and the whiskers extend to 1.5× the interquartile range. The points beyond the whiskers were considered outliers. **f**, The correlation between shared heritability and median transcriptome similarity, as calculated using Spearman’s correlation coefficient. The error bars show the 95% confidence intervals. **g**, Subclass-specific concordance (*ρ*_ss_) between shared heritability and transcriptome similarity. Spearman’s correlation coefficient was calculated using the transcriptome similarity of each subclass.

Analysing gene expression signatures across multiple disorders can reveal shared molecular pathways, facilitating improved diagnostics and targeted therapeutics. Thus, we sought to assess the extent of sharing across NDDs and NPDs. Using a pseudobulk approach and precision-weighted regression modelling for large-scale single cell datasets, implemented in the dreamlet software^47^ (Methods), we performed a comprehensive analysis of differentially expressed genes (DEGs) across eight different NDDs and NPDs. After identifying DEGs for each disease contrast and tissue source, we performed a standard frequentist fixed-effect meta-analysis across the three tissue sources to define consensus disease trait signatures. We then applied a multivariate Bayesian meta-analysis^47,48^ across cell types and disease traits to produce posterior estimates of the effect size and the posterior probability that each effect is non-zero. Using a composite testing approach, we used these results to estimate the posterior probabilities that each effect is specific to a trait or shared across traits. We then evaluated, in a cell-type-specific manner, cross-disease sharing and decomposed the total disease effects into shared and disease-specific components (Supplementary Table 4). We demonstrate that cross-disorder signatures have a higher degree of disease-effect sharing (Supplementary Fig. 8a) and encompass crucial transcriptional processes, such as mRNA splicing and processing, as well as protein localization to mitochondria (Fig. 5a and Supplementary Fig. 8b). The observed shared signatures affect genes that are critical for proper cell function align with the omnigenic model of disease risk^49^, and further support the pleiotropy of these genes, which influence multiple disorders both genetically and transcriptionally. Genes with disease-specific effects exhibited a higher burden of rare variants compared with those with shared effects (Supplementary Fig. 8e), with statistically significant enrichments observed in *VIP*^+^ and *PVALB*^+^ interneurons. While this supports the relevance of disease-specific components to core disease biology^50^, the signal was limited to a subset of cell types. To facilitate the identification of core disease-relevant functional genes, we discounted genes with shared effects from the overall DEG expression profiles. Using the residual disease-specific effects, we quantified the pairwise transcriptomic similarity between traits (Fig. 5b). Similarities between pairs of NDDs or NPDs were greater compared with the NDD–NPD contrast, with AD, DLBD, Vas and PD being the most similar. Subsequent meta-analysis using disease-specific effects of these four transcriptomically similar traits implicated neuronal development and synaptic signalling pathways in interneurons (IN_LAMP5_RELN, IN_ADARB2 and IN_PVALB), as well as vasculature development in the VLMC subclass (Fig. 5c and Supplementary Fig. 8c).

Comparison of pairwise transcriptome similarity against pairwise trait co-heritability (common genetic variation) can estimate the contribution of shared genetic influences on molecular phenotypes. We calculated the shared heritability (*r*_g_) between disease pairs using GWAS and found that pairs within NDDs or NPDs exhibited a higher degree of shared heritability (that is, DLBD–PD, SCZ–BD) compared with pairs spanning NDDs and NPDs (Fig. 5d). Quantifying the pairwise transcriptome similarity (*ρ*_s_) across NDDs and NPDs revealed varying degrees of transcriptomic overlap, with some exhibiting high similarity, particularly in disease pairs such as AD and DLBD (Fig. 5e). By correlating the shared heritability against the average transcriptome similarity across cell types, we obtained a heritability–transcriptome concordance (*r*_g_ − *ρ*_s_), which indicated a strong positive correlation (Spearman’s *ρ* = 0.604) between genetic and transcriptomic similarities (Fig. 5f and Supplementary Fig. 8f). The results show that diseases with higher genetic overlap also tend to have more similar transcriptomic dysregulation, suggesting that shared transcriptomic vulnerabilities are coupled with underlying genetic architecture, an unsurprising result that corroborates previous observations^3,51,52^. We next extended the *r*_g_ − *ρ*_s_ comparison by considering the transcriptional concordance for each cell type (Fig. 5g and Supplementary Fig. 8d). Among all cell types, Chandelier cells (IN_PVALB_CHC), a subset of GABAergic interneurons, had the highest concordance with the pairwise trait heritability. In summary, our approach enabled us to dissect disease signatures into shared and distinct components, revealing that core disease signatures have significant overlap in gene expression and genetic risk across NDDs and NPDs. These findings further our understanding of disease mechanisms in the brain, paving the way for novel therapeutic strategies targeting shared or distinct pathways.

## Transcriptomic variation with AD pathology

**Fig. 6:**
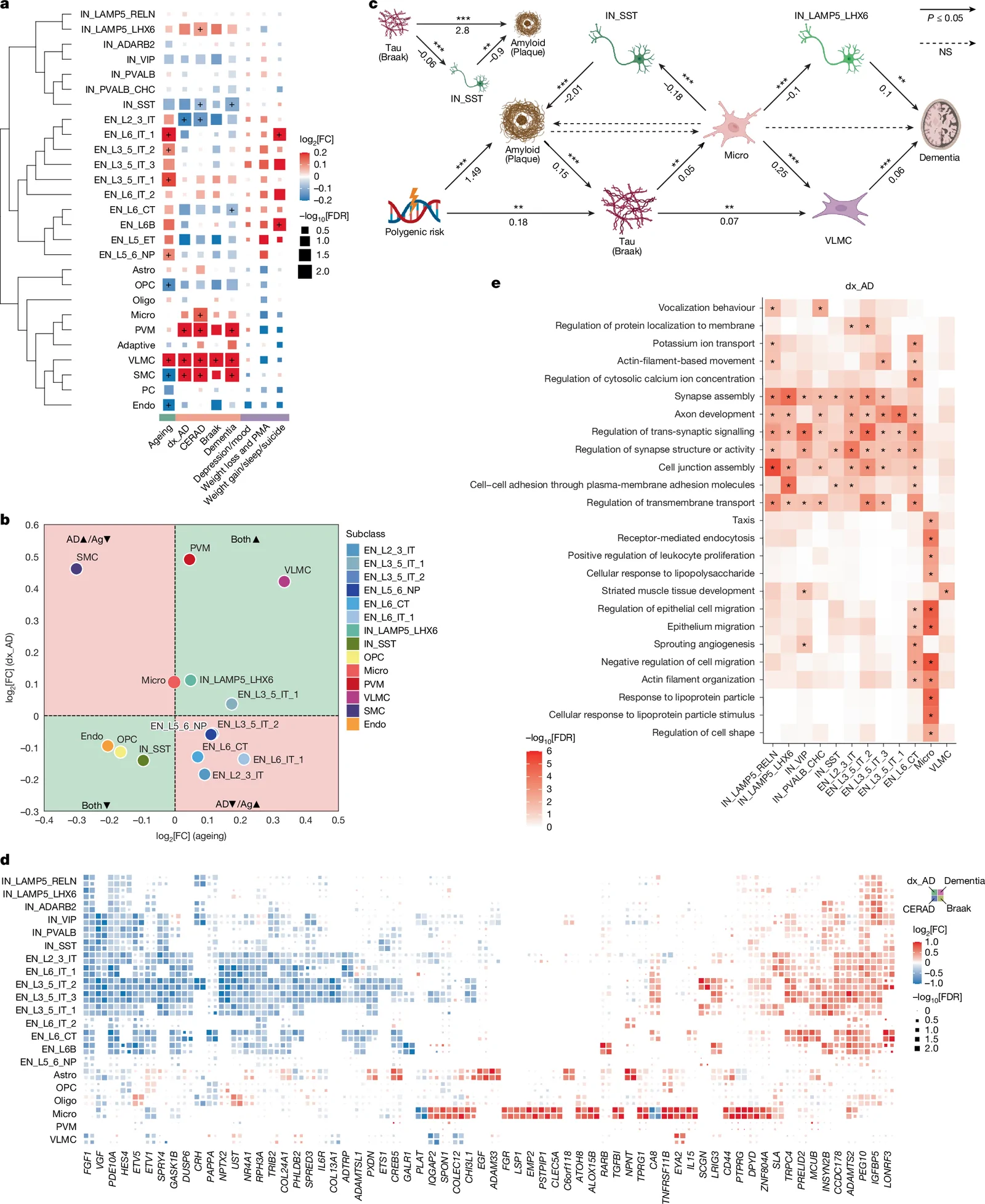
Transcriptomic variation with AD pathology. **a**, Compositional variation analysis using normal ageing, different measures of AD pathology (binary AD diagnosis (dx_AD), CERAD score, Braak staging and ordinal dementia scale), and three categories of NPSs within AD. **b**, Comparison of compositional changes between ageing (Ag) and AD. Green indicates concordant changes. Red indicates discordant changes. Only subclasses with at least one FDR significant contrast are shown. **c**, Causal mediation analysis using PRS, mean plaque, Braak staging and dementia scale. CLR-transformed subclass fractions were used for modelling. Statistical significance is indicated above the arrow (bootstrapping was used to estimate *P* values; ***P ≤ 0.001, **P ≤ 0.01 and *P ≤ 0.05), and the numbers below indicate the coefficients. The mediation effects of *SST*^+^ interneurons on Aβ plaque accumulation are shown separately. **d**, DEGs in AD phenotypes. Meta-analysis was performed between brain banks. The top genes are shown (FDR < 0.01 and effect size ≥ 0.35). **e**, Functional enrichment analysis of DEGs by subclass using GO BP pathways. FDR ≤ 0.01, as determined using the hypergeometric test, is shown. GO terms were reduced using rrvgo.

Next, we focused on characterizing the transcriptomic variation in AD, including analysis of case–control comparisons and different phenotypes that capture disease severity, such as plaque density (that is, CERAD scores), NFT progression (that is, Braak stage) and level of cognitive impairment (such as CDR). The variation in cell type composition reveals distinct patterns associated with AD pathologies compared with normal ageing (Fig. 6a, Supplementary Fig. 9a and Supplementary Table 5) and was consistent with previous studies^1,2^ (Supplementary Fig. 10a,b). Furthermore, we replicated our findings using a published MERFISH dataset^2^, showing that the observed change is robust to the technology used (Supplementary Fig. 10c). Comparing compositional shifts during normal ageing and AD (Fig. 6b), we identified an AD-specific increase in smooth muscle cells (SMCs), a vascular cell type that normally declines with age, suggesting an AD-specific vulnerability in these cells. Likewise, changes in most excitatory neurons were discordant, suggesting that the mechanisms that led to neuronal loss were AD specific. Notably, changes more pronounced in AD, such as the increase in *LAMP5*^+^*LHX6*^+^ inhibitory neurons (IN_LAMP5_LHX6) and the loss of *SST*^+^ inhibitory neurons (IN_SST) with higher CERAD scores indicates a potential association between this neuronal subtype and Aβ pathology. Moreover, the loss of L2–3 IT neurons in AD (based on case–control and CERAD comparisons), which is not evident in normal ageing, suggests a specific vulnerability for this neuronal subtype to AD. We next experimentally validated the compositional shifts using RNAscope (Methods), confirming the decline in L2–3 IT excitatory and *SST*^+^ inhibitory neurons and the prevalence of VLMCs in AD (Supplementary Fig. 9e,f). Taken together, our findings highlight the cell type specificity of AD vulnerability.

To better understand transcriptomic variation in AD, and to identify the cell subclasses most affected, we analysed the changes in cases of AD due to co-occurring conditions. It is estimated that more than 80% of patients with AD will exhibit at least one NPS over the course of their illness that substantially impacts their clinical outcome^9,53^, suggesting that there are at least some shared molecular mechanisms between serious mental illness and AD. To evaluate the cell types associated with NPS prevalence, we applied compositional variation analysis to three categories of NPS based on co-occurrence estimates. Notably, using age-matched groups of patients with AD with or without NPSs (Supplementary Fig. 9b), we found that patients with AD experiencing weight loss and PMA have an increased ratio of excitatory neurons, especially deep-layer neurons in L5–6 (Fig. 6a and Supplementary Fig. 9a,c,d). Our results corroborate previous findings^54^ that changes in deeper PFC layers (L5–6) are associated with certain types of NPS.

After identification of vulnerable cell subclasses in AD, we questioned whether their roles in AD pathology are damaging, protective, causal or derived. As demonstrated in previous studies^55^, we used mediation analysis to decipher the causal relationships between various cascades of events leading to AD onset and progression. We performed the analysis on the base hypothesis that polygenic risk (AD PRS; Methods) contributes to plaque accumulation (mean density of neuritic plaques), which in turn affects tau progression (Braak), leading to dementia. We tested 14 subclasses of which the cell type composition significantly changed in any of the AD contrasts (false-discovery rate (FDR) < 0.05) and identified that microglia, VLMCs, *SST*^+^ and *LAMP5*^+^*LHX6*^+^ inhibitory neurons exhibit significant average causal mediation effects (ACME, *P* < 0.05) (Fig. 6c and Supplementary Table 13). Beyond the direct effects of polygenic risk on tau pathology, we observed significant indirect effects mediated by plaque accumulation (ACME = 0.0903, *P* < 2 × 10^−16^). Tau progression results in more VLMCs mediated by an increase in microglia (ACME = 0.00477, *P* = 0.005). Conversely, an increase in microglia leads to a decrease in *LAMP5*^+^*LHX6*^+^ inhibitory neurons. Such changes in the levels of both VLMCs (ACME = 0.00515, *P* = 0.0002) and *LAMP5*^+^*LHX6*^+^ inhibitory neurons (ACME = −0.00269, *P* = 0.031) appear to exacerbate dementia. Furthermore, plaque accumulation is mitigated by *SST*^+^ inhibitory neurons (ACME = 0.1475, *P* < 2 × 10^−16^). Increased microglia lower *SST*^+^ inhibitory neurons (coef = −0.18, *P* = 5.04 × 10^−7^), as does increased tau (coef = −0.06, *P* = 1.82 × 10^−4^), contributing to more plaque accumulation (coef = −2.01, *P* = 7.47 × 10^−6^).

To determine how gene programs change in response to increasing severity of AD pathology, we characterized the DEGs in 27 subclasses (Fig. 6d, Supplementary Fig. 11b and Supplementary Table 6). In general, DEGs had high concordance across different AD pathology variables, consistent with previous reports^1^, and the disease signatures were highly concordant with previous studies^1,2^ (Supplementary Fig. 12a–e). Overall, DEGs in AD (FDR < 0.05) can be summarized as upregulation of genes in vascular cell classes (Mural and Endo), while genes in neurons tend to be downregulated (Supplementary Fig. 11a). We observed that microglia-specific DEGs, including *DPYD*, *IL15* and *PTPRG*^47,56,57^, generally exhibited higher effect sizes compared with those of other subclasses. Further characterizing microglia-associated gene signatures, we found that they are specifically enriched for pathways involved in negative regulation of cell motility and migration, as well as response to lipoproteins (Fig. 6e and Supplementary Fig. 11c). Gene expression changes in neurons were largely related to synaptic functions, including synapse assembly, development, signalling and membrane transport. Lastly, genes altered in VLMCs were implicated in muscle tissue development.

## Nonlinear dynamics of the AD pathological trajectory

**Fig. 7:**
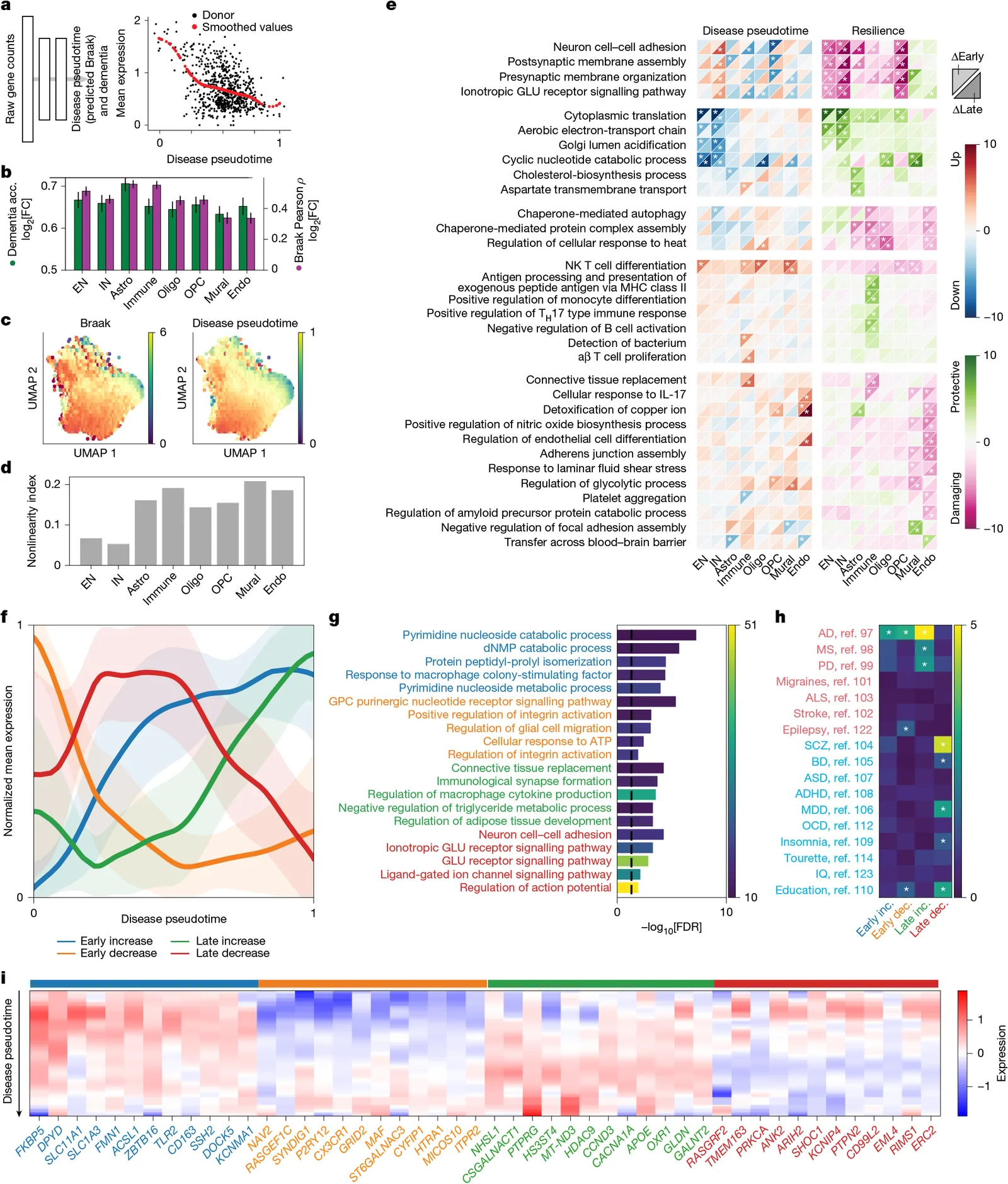
Modelling of AD using disease trajectory analysis. **a**, Neural network models to predict Braak stage and dementia status from the raw gene counts of individual cells, from which we constructed gene trajectories (Methods). The trajectory of the *NAV2* gene for the immune cell class is shown as an example. **b**, The accuracy (acc.) of the models by cell class. The error bars show the s.d. from donor-level bootstrap resampling; the number of eligible donors per cell class is provided in the Methods. **c**, UMAP projection of the microglia subtype. The hue indicates the mean actual Braak (left) and disease pseudotime prediction (right) at each location on the UMAP space. **d**, The nonlinearity index by cell class. **e**, Pathway enrichment by early (top left triangles) and late (bottom right triangles) phases of the AD trajectory, as predicted by disease pseudotime and dementia resilience. The hue indicates the *z* score (clipped between −10 and 10); *FDR < 0.05. **f**, Four representative disease trajectory modules for the immune cell class. The mean-normalized expression of the 250 genes with the greatest early-increasing (early inc.; blue curve), early-decreasing (orange), late-increasing (green) and late-decreasing (red) slopes based on disease pseudotime trajectories. **g**, Pathway enrichment of disease trajectory modules. Text is coloured by the four trajectory modules, as in **f**. The hue indicates the number of genes in the pathway. **h**, Enrichment of heritability estimates (MAGMA) for each disease trajectory module. The text colour indicates neurological traits (red) and psychiatric traits (blue). The hue indicates −log_10_[FDR] (clipped at 5); *FDR < 0.05. **i**, Top 12 genes for each trajectory module. The hue indicates the rate of normalized expression change (slope) based on the predicted disease pseudotime. For visualization, we show each gene only once even if it appears in more than one module. References ^122,123^.

Recent studies highlight the complex, time-dependent changes in gene expression and cellular function during AD progression. Early stages are characterized by increased inflammation, astrocyte reactivity, blood–brain barrier dysfunction and heightened vulnerability in select neuronal populations^1,2,46,55,58,59^. These are followed by a more dysregulated immune response and broader neuronal loss in the late stages^60–65^. To further investigate the gene expression dynamics underlying AD pathogenesis, we used neural network models to generate two independent cell-type-specific disease trajectories from semi-quantitative measures of AD progression (that is, tau proteinopathy and cognitive decline) (Fig. 7a). As tau proteinopathy and dementia are correlated, models that do not disentangle their effects may miss genes with opposing expression patterns. For example, if a gene’s expression rises with tau but falls with dementia, these effects could cancel one another out, obscuring significant changes. Thus, to decorrelate Braak and dementia model predictions, we equally sampled all combinations of Braak stage and dementia status during model training (Methods and Supplementary Fig. 14; potential consequences of not decorrelating are shown in Supplementary Fig. 15). The accuracies of the Braak and dementia model predictions were significantly above chance for all eight cell classes (*P* < 1 × 10^−4^, bootstrap) (Fig. 7b), with predictions generated on cross-validated data, using non-overlapping donors for training and testing. Visualizing the model predictions of Braak (hereafter, disease pseudotime) in a UMAP space for microglia shows that actual Braak values closely align with the model predictions (Fig. 7c and Methods).

Many past studies have suggested that gene expression can evolve nonlinearly with disease progression^66^. Thus, we performed principal component analysis (PCA) analysis of the concatenation of all disease pseudotime trajectories (Fig. 7a) across all genes and cell types, revealing the presence of a nonlinear transition between early and late disease stages (Supplementary Fig. 16), and showing that glial and vascular trajectories were more nonlinear than neuronal trajectories (Fig. 7d and Supplementary Fig. 17; *P* < 0.05, Wilcoxon rank-sum test). In addition to the disease pseudotime axis, we also calculated a dementia resilience score that measured how gene expression is correlated with predicted dementia, conditioned on the predicted disease pseudotime (Methods). A gene is considered protective if increased expression is associated with a decrease in predicted dementia, conditioned on the disease pseudotime, and damaging if increased expression is associated with an increase in predicted dementia. We then determined the top 32 Gene Ontology (GO) Biological Process (BP) pathways that best summarize the biological changes associated with early and late changes in disease pseudotime and resilience (Fig. 7e, Supplementary Figs. 18–20 and Supplementary Table 7).

A semantic clustering of the pathways identified five functional clusters. The first cluster was primarily related to synaptic function, characterized by downregulation in OPCs in the early stages of AD, and then upregulation in neurons and downregulation in immune cells in later stages. For these cell types, increased expression was associated with diminished resilience (that is, damaging). A visualization of these damaging synaptic pathways is shown in Supplementary Fig. 21a,b. The late increase in genes implicated in these pathways could be linked to compensatory mechanisms after synaptic loss^67^. The second cluster was related to cell metabolism pathways implicated in protein translation^2,68^, mitochondrial function^2,69^ and acidification^70^, which showed strong downregulation in neurons with increasing disease pseudotime, consistent with past studies^71^. Decreased expression in these metabolic pathways was associated with decreased resilience (increase in metabolism was protective). The third cluster was related to cell stress, including pathways related to chaperone-mediated protein assembly, autophagy and response to heat. In non-neuronal cells, both early and late increases in cell stress were strongly associated with cognitive decline. The fourth cluster was related to immune response and inflammation. Immune cells were implicated immune responses such as antigen presentation and helper T cell response, as well as monocyte differentiation, all of which were associated with dementia resilience. A visualization of these protective immune pathways is shown in Supplementary Fig. 21c,d. Lastly, vascular cells were implicated in damaging changes associated with adherens junction assembly, response to interleukin-17 (IL-17), glycolysis, endothelial cell differentiation and nitric oxide synthesis.

While this analysis included all pathways, we also aimed to identify potentially causal pathways driving disease pathogenesis. To do so, we repeated our analysis, focusing only on GO BP pathways enriched in risk genes (Methods). One particularly notable example is the negative regulation of peptidyl-threonine phosphorylation (Supplementary Figs. 22 and 23), which has been implicated in tau phosphorylation^72^. The mean expression of this pathway selectively decreases early in both neural cell types and includes the risk gene *SPRED2* (ref. ^73^).

As previous studies have highlighted the importance of the immune response in AD^57,61–64,74–76^, we examined this cell class in more detail (Fig. 7f–i). To help visualize the nonlinear dynamics of the immune response in AD, we categorized gene expression trajectories into four modules on the basis of their response to increasing tau proteinopathy: early increasing, early decreasing, late increasing and late decreasing (Fig. 7f). Gene enrichment of these four trajectory modules showed that pathways involved in macrophage colony stimulation and pyrimidine metabolism were all upregulated in the early stage (Fig. 7e), whereas migration, purinergic signalling and integrin activation were downregulated. In the later stages of disease, lipid-related pathways such as adipose tissue development and triglyceride metabolism, in addition to cytokine production, increased, while synapse-related pathways were downregulated. Gene-set enrichment analysis using GWAS summary statistics for the top 250 genes in each trajectory module revealed that late-increasing genes were the most strongly associated with AD (FDR ≤ 1.7 × 10^−7^), although early-increasing (FDR ≤ 1.7 × 10^−3^) and early-decreasing (FDR ≤ 7.9 × 10^−4^) genes were also significantly associated (Fig. 7h and Supplementary Table 8). Notably, the late-increasing trajectory module was also significantly correlated with other NDDs, including MS and PD. We also find that changes across many cell classes significantly overlap with NPD risk loci (Supplementary Fig. 24 and Supplementary Table 8).

We further examined the genes associated with trajectory modules (Fig. 7i; the top genes for all cell classes are listed in Supplementary Table 9). Several markers for homeostatic microglia^57^, including *CX3CR1*, *NAV2* and *P2RY12*, were among the top 5 early decreasing genes with increasing disease pseudotime (*FRMD4A* and *CERC2* were ranked in the top 20 out of 17,265 coding genes). By contrast, several early-increasing genes, such as *ACSL1*, *DPYD* and *CD163* were recently implicated in a pathogenic lipid-droplet accumulation phenotype in patients with *APOE4/4* AD^61^ (another implicated gene, *NAMPT*, ranked 134th; Supplementary Fig. 26a and Supplementary Table 9).

To further resolve the immune response at a finer time resolution, we performed pathway enrichment using a sliding window across disease pseudotime (Supplementary Fig. 25). Our results suggest two distinct immune phases^60^: an initial innate response, followed by a more adaptive and monocyte-driven response, along with increased cytokine production. Lipid homeostasis and efflux pathways, many involving known AD risk genes, increased throughout disease progression, particularly in later stages (Supplementary Figs. 25 and 26b), supporting the hypothesis that microglia develop a lipid-droplet accumulation phenotype that may exacerbate disease progression^61–63^.

## Discussion

Here we report a comprehensive single-nucleus transcriptomic atlas of the human DLPFC, generated using 1,494 donors, including neurotypical controls and individuals affected by a range of complex neurodegenerative and/or neuropsychiatric conditions. Our analyses provide insights into the cellular heterogeneity of the DLPFC, including the observation that about 10% of total transcriptomic variation can be attributed to interindividual differences. Notably, we find that genes with lower interindividual variability are under higher genetic constraints. These gene programs are enriched with neuronal development pathways, indicative of their critical roles in development and fitness.

We used our disease atlas to characterize cross-disease and disease-specific responses to pathologic conditions, revealing shared and distinct cellular composition profiles among NDDs and NPDs. Shared signatures include genes critical for proper cellular function (mRNA processing and protein localization), and align with the omnigenic model^49^, in which a portion of heritability in complex traits can be explained by effects on peripheral, often regulatory, genes that can have indirect, subtle and cumulative roles in disease. By focusing on core disease-specific functional genes, we found that disease pairs with higher genetic risk overlap tend to have greater cell-type-specific transcriptomic concordance, corroborating previous observations from bulk assays^3^ that genetic factors contributing to disease susceptibility can influence transcriptomic alterations in similar ways. The concordance between shared differential expression patterns and genetic co-heritability suggests that overlapping transcriptomic signatures reflect core biological processes influenced by pleiotropic risk variants. This concordance provides insight into molecular pathways that may be causally linked to disease aetiology across disorders. Cell types with higher concordance, including deep-layer excitatory neurons and *PVALB*^+^ Chandelier cells, probably represent critical sites of genetic risk convergence and may warrant prioritization in mechanistic studies. While disease-specific differential expression signatures remain important for understanding disorder-specific pathophysiology, shared signatures offer a window into fundamental processes contributing to comorbidity and pleiotropy.

Among our findings is the observation that the brain vascular system is intricately linked to immune dysfunction in AD. In general, we saw a relative increase in vascular cell types in most NDDs (Supplementary Fig. 6e,f) and demonstrate that levels of VLMCs are particularly elevated in AD among individuals with more severe cognitive impairment (Fig. 6a,c and Supplementary Fig. 9e,f). It has previously been shown that the meningeal lymphatic system has multiple roles in the brain, including waste removal^77–79^ and the adaptive immune response^46,80^. Given the role of VLMCs in these processes^81–84^, their contribution to disease warrants further investigation.

Our analysis of the pathological trajectory of AD aligns with various proposed hypotheses^71,85–89^, particularly for pathways affected in the earliest stages of the disease. Neuronal, immune and vascular cells exhibit distinct vulnerabilities, with shared alterations across correlated pathways. In neurons, we observed a decrease in metabolic functions (cyclic nucleotide catabolic process, cytoplasmic translation and ATP synthesis) that have been closely linked to synaptic dysfunction and cognitive decline^71^. On the other hand, the immune response appears to be generally protective, whereby an early innate immune activation^85^ followed by an adaptive immune response^64,75,76^ was associated with resilience to dementia. In particular, monocyte differentiation appeared to have a protective role during both early and late disease stages, consistent with the idea that monocyte-derived macrophages serve as reinforcements when the immune system is under sustained stress^90^. The one exception was the response of immune, mural and endothelial cell classes to IL-17, which was damaging in the early stages of AD. IL-17 has been associated with cognitive decline^91,92^ and disruption of the blood–brain barrier^93^, and blocking IL-17 function has been shown to mitigate cognitive defects in murine models of AD^91,92^. Moreover, chaperones have been closely associated with a number of disease related processes, including tau misfolding and aggregation, and neurotoxicity^86–89,94,95^; however, their precise roles in AD pathogenesis remains unclear. Our results suggest that chaperones can have opposing roles depending on the cellular context—damaging for glia, immune and endothelial cells but possibly protective for neurons. Lastly, upregulation of endothelial cell stress, differentiation and adhesion/junction pathways were associated with cognitive decline, consistent with the idea that blood–brain barrier dysfunction^58^—a known hallmark of dementia—may trigger compensatory increases in these pathways.

Taken together, these insights deepen our understanding of AD pathogenesis and implicate cellular responses that warrant further investigation. While the human brain comprises numerous regions with distinct functions, in this study, we limited our analysis to the DLPFC. Future studies that include additional brain regions will provide insights into a broader range of phenotypes and offer a more holistic view of the molecular foundations of brain function in both health and disease. Moreover, more detailed spatial analyses, including whole-cell transcriptomics, could further elucidate the physiological contexts that contribute to disease. Analysing larger numbers of cells may also uncover rarer disease-associated cell types or subtypes, while integrating additional omics modalities (for example, proteomics or epigenomics) will provide deeper molecular insights. Overall, the PsychAD single-cell disease atlas serves as a unique and foundational resource to further our understanding of population-level disease-associated transcriptomic variation in the human brain.

## Methods

### Collection and harmonization of clinical, pathological and demographic metadata

Brain tissue specimens were sourced from two brain banks: the Mount Sinai NIH Neurobiobank (MSSM) (1,042 samples) and the NIMH-IRP Human Brain Collection Core (HBCC) (300 samples). Furthermore, samples were obtained from five prospective cohort studies conducted at the Rush Alzheimer’s Disease Center (RADC) (152 samples)^124,125^. Thus, the availability of clinical metadata varied as a function of source. We used the following scheme to harmonize available clinical, pathological and demographic metadata: the CERAD scoring scheme for neuritic plaque density^5^ was harmonized for consistency across multiple brain banks, for which the scores range from 1 to 4, with increasing CERAD number corresponding to an increase in AD burden; 1, no neuritic plaque (normal brain); 2, sparse (possible AD); 3, moderate (probable AD); 4, frequent (definite AD). Samples from RADC used consensus summary diagnosis of no cognitive impairment (NCI), mild cognitive impairment (MCI), and dementia and its principal cause, Alzheimer’s dementia^126–128^. MSSM samples used clinical dementia rating (CDR), which was based on a scale of 0–5; 0, no dementia; 0.5, questionable dementia (very mild); 1, mild dementia; 2, moderate dementia; 3, severe dementia; 4, profound dementia; 5, terminal dementia. After consulting with clinicians, we created a harmonized ordinal variable in which dementia is categorized into three levels of cognitive decline, independently of AD diagnosis: 0, no cognitive impairment; 0.5, MCI; and 1–5, dementia. In addition to AD phenotype, we collected comprehensive demographic (age, sex and genetic ancestry) and technical variables (tissue source, technician, sample batch, postmortem interval (PMI; measured in min), *APOE* genotype) to describe each cohort (Supplementary Table 1). We described the process for assigning genetic ancestry in a previous data descriptor paper^129^. In brief, we leveraged quadratic discriminant analysis (QDA) to infer genetic ancestry by training our model using data from the 1000 Genomes Project. We used tenfold stratified cross validation to optimize the regularization parameter within QDA^130^, as well as forward selection to identify the optimal number of PCs for genetic ancestry assignments. For samples without genotypic data, we used race/ethnicity as a proxy for inferring genetic ancestry. We emphasize that, while genetic ancestry is a distinct concept from the social constructs of race and ethnicity^131^, we leveraged the correlated race/ethnicity variables as proxies to retain those samples in the analyses. Values for superpopulations included EAS, SAS, AFR, AMR, EUR and EAS_SAS, where the category EAS_SAS was assigned to samples with unavailable genotypes with an ‘Asian’ value for race/ethnicity, which can potentially correspond to both EAS and SAS.

### Clinical diagnosis of AD

For analysis comparing donors with AD cases and neurotypical controls, a binary clinical diagnosis variable for AD, dx_AD, was defined, as follows: individuals with CERAD 2, 3 or 4, Braak ≥ 3 and CDR ≥ 1 for MSSM or Alzheimer’s dementia for RADC were classified as AD cases. Controls were defined as individuals in the controls_neuropathological_clinical category, where CERAD = {1}, Braak = {0,1,2,3} and secondary diagnosis (including dementia) is not allowed except for MCI.

### Measuring AD neuropathology

For analysis comparing donors with pathologic AD, the following variables were used to measure the severity of AD neuropathology: CERAD score^5^. A quantitative measure of Aβ plaque density in which 1 is normal, 2 is possible AD, 3 is probable AD and 4 is definite AD^126^. Braak AD-staging score^6–8^ measuring progression of NFT neuropathology (Braak and Braak score, or BBScore). A quantitative measure of the regional patterns of NFT density across the brain, in which 0 is normal and asymptomatic, 1–2 indicates initial stages where NFTs begin to appear in the locus coeruleus and the transentorhinal region, 3–4 indicates progression to limbic regions, such as the hippocampus and amygdala, and 5–6 indicates that NFTs are widespread, affecting multiple cortical regions.

### Measuring cognitive impairment

For analysis comparing donors with AD-related dementia, the following variable was used to measure the severity of cognitive impairment: clinical assessment of dementia. A harmonized variable of cognitive status based on the CDR scale for MSSM or NCI, MCI and Alzheimer’s dementia for RADC. We used the three-level ordinal categories of clinical dementia to measure the severity of dementia, in which 0 indicates no dementia, 0.5 indicates minor cognitive impairment, and 1.0 indicates definite clinical dementia.

### Definition of cross-disorder contrasts

For cross-disorder contrasts, we limited the analysis to any individual with age ≥ 17. Neurotypical controls are defined as any individual CERAD = {1} and Braak = {0,1,2,3} and secondary diagnosis is not allowed. AD is any individual with CERAD = {2,3,4}, Braak = {3,4,5,6} and clinically diagnosed as dementia, and secondary diagnosis not allowed. SCZ is any individual with SCZ diagnosis (SCZ|Schizoaffective_bipolar|Schizoaffective_depressive) and secondary diagnosis not allowed, except for metabolic and eating disorders. DLBD is any individual with DLBD diagnosis (DLBD), and secondary diagnosis can be only AD. Vascular is any individual with vascular diagnosis (vascular) and secondary diagnosis can be only AD. BD is any individual with BD diagnosis (BD_unspecific|BD_I|BD_II|Schizoaffective_bipolar) and secondary diagnosis not allowed except for metabolic and eating disorders. Tauopathy is any individual with CERAD = {1}, Braak = {4,5,6} and secondary diagnosis allowed. PD is any individual with PD diagnosis (PD|PD_uncertain_plus_encephalitic) and secondary diagnosis can be only AD. FTD is any individual with FTD diagnosis (FTD) and secondary diagnosis can be only AD. All disease contrasts used in the study can be found in Supplementary Table 1.

### Isolation and FANS of nuclei from frozen brain specimens with hashing

All buffers were supplemented with RNase inhibitors (Takara). Then, 25 mg of frozen postmortem human brain tissue was homogenized in cold lysis buffer (0.32 M sucrose, 5 mM CaCl_2_, 3 mM magnesium acetate, 0.1 mM, EDTA, 10 mM Tris-HCl, pH8, 1 mM DTT, 0.1% Triton X-100) and filtered through a 40 µm cell strainer. The flow-through was underlaid with sucrose solution (1.8 M sucrose, 3 mM magnesium acetate, 1 mM DTT, 10 mM Tris-HCl, pH 8) and centrifuged at 107,000*g* for 1 h at 4 °C. The pellets were resuspended in PBS supplemented with 0.5% BSA. Six samples were processed in parallel. Up to 2 million nuclei from each sample were pelleted at 500*g* for 5 min at 4 °C. Nuclei were resuspended in 100 µl staining buffer (2% BSA, 0.02% Tween-20 in PBS) and incubated with 1 µg of a unique TotalSeq-A nuclear hashing antibody (BioLegend) for 30 min at 4 °C. Before fluorescence-activated nuclear sorting (FANS), volumes were brought up to 250 µl with PBS and 7-aminoactinomycin D (7-AAD) (Invitrogen) added according to the manufacturer’s instructions. 7-AAD-positive nuclei were sorted into tubes precoated with 5% BSA using the FACSAria flow cytometer (BD Biosciences) (Supplementary Fig. 30). FACSDiva software (BD Biosciences, v.8.0.2) was used for data collection.

### snRNA-seq and hashing library preparation

After FANS, nuclei were subjected to two washes in 200 µl staining buffer, after which they were resuspended in 15 µl PBS and quantified (Countess II, Life Technologies). Concentrations were normalized and equal amounts of differentially hash-tagged nuclei were pooled. Using 10x Genomics single cell 3′ v3.1 reagents (10x Genomics), 60,000 (10,000 per donor) nuclei were run in each of two 10x Genomics lanes to create a technical replicate. At the cDNA amplification step (step 2.2) during library preparation, 1 µl of 2 µm HTO cDNA PCR ‘additive’ primer was added^132^. After cDNA amplification, supernatant from 0.6× SPRI selection was retained for HTO library generation. cDNA libraries were prepared according to the 10x Genomics protocol. HTO libraries were prepared as previously described^132^. cDNA and HTO libraries were sequenced at NYGC using the NovaSeq platform (Illumina).

### Processing of snRNA-seq data

#### Alignment

Paired-end snRNA-seq library reads were aligned to the hg38 reference genome using STAR solo^133,134^ and sample pools were demultiplexed using genotype matching via vireoSNP^135^. After per-library count matrices were generated, cell by gene counts were aggregated into a single count matrix, which has 8,898,978 cells by 60,605 genes. The downstream processing was performed using Pegasus (v.1.7.0)^136^ and scanpy (v.1.9.1)^137^.

#### QC

We applied a rigorous three-step quality control (QC) process to filter out low-quality nuclei for subsequent downstream analyses. First, QC was applied at the cell level. Poor-quality nuclei were detected by thresholding based on UMI counts, gene counts and mitochondrial content. The QC thresholds were defined from log-transformed gene and UMI counts. Three median absolute deviations were used as the lower limit. This resulted in 986 for genes and 1,179 for UMI counts as lower bounds. We used a hard cut-off of 15,000 genes and 200,000 UMI counts as upper limits. Any cells with mitochondrial genes greater than 1% were filtered out. We checked for possible contamination from ambient RNA using CellBender^138^. We also checked for cells with high fractions of reads that mapped to non-mRNAs, such as rRNA, sRNA and pseudogenes, as well as known confounding features, such as the lncRNA *MALAT1*. Second, QC was applied at the feature-level by removing those that were not robustly expressed in at least 0.05% of nuclei. Lastly, QC was applied at the donor-level by removing any sample represented by <50 nuclei. Finally, we excluded donors with low genotype concordance and sex discrepancies. Detailed QC statistics (median gene and UMI counts, median percent mitochondrial genes and cell counts), separated by 2,924 single-cell libraries used, before and after the removal of low-quality cells are available in Supplementary Table 12.

#### Normalization

We applied standard pegasus log-normalization with default parameters after QC.

#### Doublet detection

After QC and log-normalization, we performed the first-pass Leiden clustering analysis on the whole dataset to prepare input for doublet inference step. On the basis of the first-pass clusters, further filtering was carried out by removing inferred doublets using the Scrublet method^139^.

#### Batch correction

We assessed the correlation between all pairs of donor and technical variables using canonical correlation analysis (Supplementary Fig. 7a). We attempted to remove unwanted technical batch effects during downstream analysis. During highly variable gene selection, we use the tissue source as a batch variable to remove the effect of brain tissue source. After the PCA step, the effects of UMI counts, mitochondrial percentage and the cell cycle were regressed out. We further removed the effect of sequencing batch using the Harmony method^140^.

### Defining cellular taxonomy using iterative clustering

#### Hierarchical cellular taxonomy

Cellular taxonomy was defined using a divide-and-conquer strategy. From the full dataset containing over 6 million nuclei, 8 major cell classes were defined using the following steps: 6,000 highly variable genes (HVGs) were selected from mean and dispersions trends^141^ using the default parameters (min_mean=0.0125, max_mean=3, min_disp=0.5) and the brain source as a batch variable after manually excluding sex and mitochondrial chromosomes. We used the *k*-nearest-neighbour (*k*-NN) graph calculated on the basis of harmony-corrected PCA embedding space to cluster nuclei of the same cell type using Leiden^142^ clustering algorithms. We used UMAP^143^ to visualize the resulting clusters. From the class-level clusters, we subsetted the data by each class. Recalculating HVGs among cells in the same class enabled us to refocus on a feature space that is more relevant for the same class of cells. A *k*-NN graph was then calculated on the basis of the harmony-corrected PCA of the selected HVGs. Leiden clustering was used to annotate 27 subclass-level annotations. We iterated the same HVG–*k*-NN–Leiden clustering for all 27 subclasses yielding 67 subtypes of human brain cells. After obtaining annotations in three levels of hierarchy, the resulting clusters were aggregated into pseudobulk and cluster-wise Pearson correlation coefficient was calculated using existing human DLPFC^10^ and M1^11^ annotations. We matched the annotations on the basis of both high correlation and specificity of the cell type. The final cellular taxonomy was compared to the following snRNA-seq datasets: PFC from ROSMAP cohort^1^ and PFC (Brodmann area 9) from SEA-AD cohort^2^ (Supplementary Table 11).

#### Evaluation of cellular taxonomy

To ensure that subsequent clustering truly identifies biological subtypes that are not confounded by technical variables such as brain sources or donor variables such as age and sex, we evaluated the quality of our most granular subtype-level cell annotations with other published datasets^1,2^. To quantify the separability of cell groupings across datasets, we followed the approach of previous work in which local inverse Simpson’s index (LISI) was used to quantify the contribution of biological and technical factors to transcriptional variation^140^. We computed the LISI scores using various covariates, including cell type, donor, age, tissue source and sex. LISI measures the degree of local label mixing in low-dimensional space and is sensitive to the number of distinct clusters present in a dataset. To ensure comparability across datasets, we applied LISI using a harmonized set of metadata categories and calculated scores using the compute_lisi function with a perplexity of 30 and nn_eps = 0, based on embeddings derived from a randomly sampled subset of cells. As datasets with more granular annotations (that is, greater numbers of clusters) tend to yield higher LISI values due to increased opportunities for label separation in local neighbourhoods, we normalized the mean LISI by dividing it by the natural logarithm of the number of clusters (adjusted LISI = mean LISI/log[*n*_clusters_]). This log-based normalization compresses the scale of the cluster count, helping to avoid over-penalizing datasets with finer annotations while still accounting for granularity-related bias. The resulting adjusted LISI scores allow for more equitable comparisons of separability across datasets with varying label resolution.

### Spatial validation of cellular taxonomy

#### Xenium in situ panel design

The Xenium Human Brain Gene Expression Panel (1000599, 10x Genomics) and a custom panel of 100 genes (Supplementary Table 10) were selected for the Xenium experiment. The 100 gene custom panel consisted mainly of subclass markers selected on the basis of specificity and gene expression level. The custom gene list was sent to 10x genomics, and the probe design was performed using their in-house pipeline.

#### Tissue preparation

Fresh-frozen tissue specimens of DLPFC were dissected into small blocks on ice. Tissue blocks were snap frozen by submerging in an isopentane (320404-1L, Sigma-Aldrich) bath chilled with dry ice and stored in −80 °C. Before cryosectioning, tissue blocks were allowed to equilibrate to the cryostat (HM505, Microm) chamber temperature, and were mounted with OCT (Tissue-Tek O.C.T. Compound, 4583, Sakura Finetek). After trimming, good-quality 10 µm sections were flattened on the cryostat stage and placed onto pre-equilibrated Xenium slides (Xenium Slides & Sample Prep Reagents, 1000460, 10x Genomics). Then, 2–3 sections were placed on each slide. The sections were further adhered by placing a finger on the backside of the slide for a few seconds and were then refrozen in the cryostat chamber. The slides were sealed in 50 ml tubes and stored at −80 °C until Xenium sample preparation.

#### Sample preparation

Xenium sample preparation was performed according to the manufacturer’s protocol (‘Xenium In Situ for Fresh Frozen Tissues – Fixation & Permeabilization, CG000581, Rev C’ and ‘Xenium In Situ Gene Expression - Probe Hybridization, Ligation & Amplification, User Guide, CG000582, Rev C’). In brief, fresh frozen sections mounted onto Xenium slides from the previous step were removed from −80 °C storage on dry ice before incubation at 37 °C for 1 min. The samples were then fixed in 4% paraformaldehyde (formaldehyde 16% in aqueous solution, 100503-917, VWR) in PBS for 30 min. After rinsing in PBS, the samples were permeabilized in 1% SDS for 2 min and then rinsed in PBS before being immersed in the prechilled 70% methanol and incubated for 60 min on ice. After rinsing the samples in PBS, the Xenium Cassettes were assembled onto the slides. The samples were incubated with a probe hybridization mix containing both the Xenium Human Brain Gene Expression Panel (1000599, 10x Genomics) and a 100 custom gene panel at 50 °C overnight to allow the probes to hybridize to targeted mRNAs. After probe hybridization, the samples were rinsed with PBST and incubated with Xenium post hybridization wash buffer at 37 °C for 30 min. The samples were then rinsed with PBST and ligation mix was added. Ligation was performed at 37 °C for 2 h to circularize the hybridized probes. After rinsing the samples with PBST, amplification master mix was added to enzymatically amplify the circularized probes at 30 °C for 2 h. After washing with TE buffer, autofluorescence was quenched according to the manufacturer’s protocol and nuclei were stained with DAPI before Xenium in situ analysis.

#### Data processing

The prepared samples were loaded into the Xenium analyser and run according to the manufacturer’s instructions ‘Xenium Analyzer User Guide CG000584 Rev B’. After the Xenium analyser was initiated, the correct gene panel was chosen, and decoding consumables (Xenium Decoding Consumables, PN-1000487, 10x Genomics) and reagents (Xenium Decoding Reagents, PN-1000461, 10x Genomics) were loaded. The bottom of the slides was carefully cleaned with ethanol before loading. Once the samples were loaded and the run was initiated, the instrument scanned the whole sample area of the slides using the DAPI channel, and regions of interest were selected to maximize the capture area. Results were generated by the instrument using the default settings. Instead of using 15 µm nuclei expansion distance for segmentation of cells, the default for the Xenium analyser, we applied nuclei only segmentation (Supplementary Fig. 4a) by resegmenting the results with 0 µm nuclei expansion using the Xenium ranger: xeniumranger resegment --id=demo --xenium-bundle=/path/to/xenium/files --expansion-distance=0 --resegment-nuclei=True.

#### QC and major cell type identification

After nuclei were segmented, cell × gene matrices were generated from the overlap of each segmented nuclear boundary with detected transcripts in the Xenium experiment. Nuclei were subsequently filtered by the number of detected transcripts (Supplementary Fig. 4b), and only those containing at least 30 nuclear transcripts were retained for downstream analysis (Supplementary Fig. 4c). Gene expression data from each sample were then log-normalized and normalized data were used for PCA, *k*-NN graph calculation and Leiden clustering. Clusters were then assigned to one of eight major cell types using label transfer (see the next section). Clusters in which <90% of nuclei shared the same predicted class, as well as nuclei whose predicted class differed from the cluster they were assigned to, were removed (Supplementary Fig. 4c). Finally, all retained nuclei were assigned the predicted class assigned to them (Supplementary Fig. 4d).

#### Class label prediction

We used scANVI^144^ to perform reference-based label transfer from the RADC dataset. In brief, we performed the following steps. First, snRNA-seq gene expression data were subset to the genes shared with the Xenium gene panel. Next, we used the scvi-tools package^145,146^ to train machine learning models for dimensionality reduction based on the reference dataset and its assigned labels (such as class and subclass). Models were run with 5 layers and 30 latent variables, and the scANVI model was trained for 20 epochs with a minimal sample of 100 cells per cluster per epoch. Lastly, a transfer model was trained for 100 epochs and applied to query data to assign labels on the basis of those the model was trained on from the reference. To assess the performance of each transfer model, we asked the model to predict labels in the reference data (using the subset gene pool) and evaluated the rate of correct prediction and biases in label misassignment for each predicted label.

#### Subclass and subtype label transfer for neuronal cells

After subsetting to nuclei labelled as EN or IN, these were then used as a query for a second scANVI label transfer—this one trained on subclass (or subtype) labels. Accuracy for both major cell type and subclass models assessed by predicted labels in the RADC dataset based only on the Xenium gene panel was estimated at >98%.

#### Pseudo-bulk concordance between subtypes in Xenium and RADC

To estimate the concordance of defined labels between the reference (RADC cohort) and Xenium datasets, we first generated pseudo-bulk estimates of each group (subtype) by summing the counts of each gene in all cells belonging to that group in each dataset. These pseudo-bulk count matrices were then subjected to TPM and log-transformation, before each gene was normalized by *z* scoring. The *z*-score matrices of the two datasets were then used to calculate the Pearson correlation between each pair of subtypes across the datasets.

#### Definition of rough laminar layers within tissue

After assignment of class and subclass identities to Xenium nuclei, we identified regional domains of interest and laminar layers using a thresholded local composition approach. First, we calculated the 30 nearest neighbours of each nucleus in each sample, using squidpy’s spatial_neighbors function^147^ and constructed a matrix counting how many nearest neighbours of each subclass existed for each nucleus (also known as a neighbourhood composition vector^148^). Next, we aggregated counts from EN subclasses corresponding to physically proximal laminar layers, to increase the signal. Specifically, we aggregated counts from EN_L3_5_IT_1, EN_L3_5_IT_2, EN_L3_5_IT_3 and EN_L5_ET into a single pseudolayer (L3–5), and similarly aggregated counts form EN_L6_IT_1, EN_L6_IT_2, EN_L6_IT_CT, EN_L6B and EN_L5_6_NP into another pseudolayer (L5–6). Counts from EN_L2_3_IT were treated as a laminar layer without aggregation (L2–3). After aggregation, we ran identical domain identification processes for four spatial domains, each with a corresponding nearest neighbour count column: white matter (oligodendrocytes), and each of the three laminar layers described above. Independent domain calling was performed as follows. (1) We generated an image representation of the relevant neighbour abundance count for each tissue section, with a pixel size of 25 μm. If multiple nuclei were assigned to the same pixel, neighbour abundances were averaged. Moreover, pixels with no assigned nuclei were assigned the value of their nearest non-empty pixel to avoid edge effects. (2) Next, the nearest neighbour count image was smoothed using Gaussian blurring (with a sigma of 4 pixels, equivalent to 100 μm) and smoothed values were associated with nuclei on the basis of the pixels they belonged to. An initial domain call was then generated by applying an Otsu threshold to the smoothed nearest neighbour counts per nucleus. (3) To further smooth the domain boundary and make it spatially continuous, we coarsened the image resolution and removed holes. In brief, the binary domain calls from step 2 were again used to generate a coarser image (with twice the pixel size = 50 μm). Pixel values were summarized as binary measures—any nucleus-containing pixels were assigned 1 if at least one of those nuclei was associated with the domain in the mask generated by step 2, and 0 otherwise—then empty pixels were assigned values on the basis of their nearest non-empty neighbours. Lastly, we applied scipy’s binary_fill_holes function to make the domain spatially continuous^149^. (4) Values from this image were assigned to each associated nucleus to generate the final single-domain assignment. We applied this process to associate nuclei with each of the spatial domains described above independently and found that around 86.9% of nuclei were uniquely assigned to a single domain (~5.2% were not assigned a domain and ~7.8% were assigned to 2 or more).

#### Density estimation and comparison for IN subclasses

To estimate the density of each IN subclass within each spatial domain, we used the nucleus association from the laminar layer identification described above. To calculate density, we also needed to calculate the physical area occupied by each domain in each tissue section. This was done using the following method. (1) We generated a composite image with the same pixel size as the final domain calls (50 μm), in which pixels had a categorical value corresponding to one of the relevant domains if they were uniquely associated with that domain (and an NA value otherwise). (2) We then counted the number of pixels uniquely associated with each domain in each tissue section and converted it to μm^2^. For density analysis, only nuclei uniquely associated with a single spatial domain were considered. Moreover, we excluded five samples from the comparative density analysis due to a lack of representation of at least one of the spatial domains identified above. Densities were then simply defined as the number of nuclei from each subclass uniquely associated with each of the three rough laminar domains, divided by the total area occupied by that domain in each relevant tissue section. Statistical testing between each collection of (*n* = 6) densities in physically proximal domains was then performed using a paired-sample Wilcoxon rank-sum test, as implemented by scipy’s Wilcoxon function^149^. Density estimation and analysis for subtypes were performed similarly.

### Processing of genotypes

DNA extraction and genotyping was performed as described previously^150^. In brief, genomic DNA was extracted from frozen brain tissue using the QIAamp DNA Mini Kit (Qiagen), according to the manufacturer’s instructions. The samples were genotyped using the Infinium Psych Chip Array (Illumina) at the Mount Sinai Sequencing Core. Pre-imputation processing consisted of running the QC script HRC-1000G-check-bim.pl from the McCarthy Lab Group (https://www.well.ox.ac.uk/~wrayner/tools/), using the Trans-Omics for Precision Medicine (TOPMed)^151^. Genotypes were then phased and imputed on the TOPMed Imputation Server (https://imputation.biodatacatalyst.nhlbi.nih.gov). The samples with a mismatch between one’s self-reported and genetically inferred sex, suspected sex chromosome aneuploidies, high relatedness as defined by the KING kinship coefficient^152^ (KING > 0.177) and outlier heterozygosity (±3 s.d. from the mean) were removed. Moreover, samples with a sample-level missingness of >0.05 were removed and calculated within a subset of high-quality variants (variant-level missingness ≤ 0.02).

For ancestry assignment, genotypes were first merged with GRCh38 v2a 1000 Genomes Project data (https://wellcomeopenresearch.org/articles/4-50)^153^ using BCFtools (v.1.9)^154^. PLINK (v.2.0)^155^ was then used to calculate the merged genotypes’ principal components (PCs), after filtering (minor allele frequency (MAF) ≥ 0.01, Hardy–Weinberg equilibrium (HWE) *P* ≥ 1 × 10^−10^, variant-level missingness ≤ 0.01, regions with high linkage disequilibrium (LD) removed) and LD pruning (window size = 1,000 kb, step size = 10, *r*^2^ = 0.2) steps. For the samples of EUR ancestry assigned using the QDA method, autosomal biallelic variants with an imputation *R*^2^ > 0.8, HWE *P*  ≥  1 × 10^−6^ and variant-level missingness ≤ 0.02 were retained. Genotypes were then annotated with ancestry-specific MAF values from the National Center for Biotechnology Information’s Allele Frequency Aggregator (ALFA) (https://ftp.ncbi.nih.gov/snp/population_frequency/latest_release/). Only variants with an ancestry-specific ALFA MAF ≥ 0.01 were retained.

### PRS calculation

Polygenic risk scores (PRS) were computed for the PsychAD cohort using summary statistics from AD GWAS^96^. The PRS-CS-auto method^156^ was used, which incorporates continuous shrinkage priors to adjust the effect sizes from these summary statistics. An LD reference panel from the developers of PRS-CS, based on data from the 1000 Genomes Project^153^, was used (https://github.com/getian107/PRScs). The default settings for PRS-CS were applied, including parameters a = 1 and b = 0.5 for the *γ*–*γ* prior, 1000 Markov chain Monte Carlo iterations, 500 burn-in iterations and a thinning factor of 5. The global shrinkage parameter phi was determined using a fully Bayesian method. PLINK (v.2.0)^155^ was used to calculate the individual-level PRS. For AD PRS, two separate scores were calculated, both including and excluding the *APOE* locus. We conducted a sensitivity analysis comparing the PRS generated with and without the *APOE* locus. We found negligible differences between the two PRS, suggesting that the exclusion of *APOE* did not significantly alter the overall genetic risk profile (Supplementary Fig. 13b). The PRS with the *APOE* locus was used for the primary analysis.

### Genetic heritability analysis of polygenic risk

We established a standardized pipeline for multi-marker analysis of GenoMic annotation (MAGMA) followed by scDRS. MAGMA incorporates the association *P* values of genetic variants from the latest genome-wide association study (GWAS). We used the following GWAS summary stats in the scDRS/MAGMA pipeline: AD^96^ (excluding *APOE* locus), MS^97^, PD^98^, epilepsy^99^, migraines^100^, stroke^101^, ALS^102^, SCZ^103^, BD^104^, MDD^105^, ASD^106^, ADHD^107^, insomnia^108^, education^109^, IQ^110^, alcoholism^111^, OCD^112^, Tourette syndrome^113^, obesity^114^, T2D^115^, cholesterol^116^, rheumatoid arthritis^117^, IBD^118^ and UC^119^. We applied MAGMA using a standard window of 35 kb upstream and 10 kb downstream around the gene body. We executed scDRS using the top 1,000 gene weights, sorted by *z* score. The MAGMA and scDRS pipeline were run using the following parameters. MAGMA was run using -snp-loc g1000_eur.bim (SNP location file corresponding to the Phase 3 1000 Genome Project) and --gene-loc NCBI38.gene.loc (gene location file from NCBI build 38). Both files were obtained from https://ctg.cncr.nl/software/magma. For scDRS, the default setting was used.

### Statistical power and effect-size considerations in single-cell analyses

To complement the primary analyses presented in the Article, we conducted additional power and effect-size assessments to guide interpretation of the results and provide context for the sensitivity of our study. These analyses include both theoretical calculations and empirical evaluations designed to quantify the ability to detect changes in cellular composition and gene expression, as well as to highlight variability in power across cell types. On the basis of theoretical calculations, the statistical power to identify changes in cell type composition increases with the magnitude of the difference and the total number of cells observed. With the sample size and cell count in this study, we have >80% power to identify changes in cell type composition of ~0.1% (Supplementary Fig. 5a). For differential expression analyses, power increases with the sample size, standardized effect size and correlation between measured and true gene expression. Given the sample size in this study and moderated *R*^2^ values between measured and true gene expression, we have >80% power to detect effect sizes of 1 on Cohen’s *d* scale (Supplementary Fig. 5b). In practice, there are many factors affecting statistical power in gene expression studies. In bulk RNA-seq experiments, power is determined by biological signal and sample size in addition to total read count and magnitude of gene expression. In single-cell datasets, additional technical factors include the number of cells collected for each cell cluster and the number of reads per cell. These numbers vary widely across cell types, as does the number of individuals with sufficient cell and read counts. Thus, empirical statistical power varies across cell types and genes even when testing the case/control analysis for one disease. Moreover, the Winner’s Curse phenomenon means that effect size estimates that pass a 5% FDR cut-off in low-powered cell types will be substantially overestimated compared with effect sizes in well-powered cell types. In our analysis of AD versus controls, there is wide variation in the number of expressed genes and the number of individuals passing filters based on cell and read count. We observe that the number of DEGs increases with the number of individuals passing filters and the number of expressed genes (Supplementary Fig. 5c). Yet the mean of the absolute value of the effect size estimates decreases with numbers of individuals and expressed genes (Supplementary Fig. 5d). Indeed, there is a strong negative correlation (Spearman’s rho = 0.58) between the number of DEGs and the mean absolute effect size (Supplementary Fig. 5e). Examining the estimated effect size in more detail, we see estimated effect size centred at zero for all cell subclasses (Supplementary Fig. 5f), but the mean of the absolute estimated effect sizes varies widely for DEGs (Supplementary Fig. 5g).

### Variance partition analysis of gene expression

After creating a pseudobulk by aggregating single-cell library (Channel) per subclass-level annotation (Assay), expression values by assays were stacked using the StackedAssay function of Dreamlet. The resulting stacked pseudobulk enabled us to perform variance partition across cell types (stackedAssay). We used the following regression formula: Gene expression ~ (1|stackedAssay) + (1|Channel) + (1|SubID) + (1|Source) + (1|Ethnicity) + dx_bit + scale(Age) + Sex + scale(PMI) + log(n_genes) + percent_mito + mito_genes + ribo_genes + mito_ribo, where dx_bit indicates binary disease status excluding metabolic and eating disorders. Technical covariates log(n_genes), percent_mito, mito_genes, ribo_genes and mito_ribo were removed from the plotting and subsequent analysis because they explained less than 1 × 10^−4^% of overall gene expression variation.

To better interpret the variance partitioning analysis, we examined the relationship between variance explained and baseline gene expression across subclasses. Specifically, we plotted the proportion of variance attributed to donor, cell type and residual components as a function of mean expression (log_2_[CPM]) (Supplementary Fig. 5h). Genes with higher expression showed greater variance explained by donor and cell type, and lower residual variance, reflecting a technical effect whereby higher read counts yield more precise estimates and therefore bias the analysis toward highly expressed genes.

We performed sensitivity analyses to ensure that performing variance partition analysis across all subclasses does not obscure the cell-type-specific signals. We performed variance partition analyses within more homogeneous cell populations by stacking cell-type-specific assays into three superclasses, namely, EN, IN and non-neuron, similar to previous approach by BICCN exploring interindividual variation of human brain^157^. This setup enables better resolution of potentially meaningful covariates within a cell class while still adjusting for sample- and technical-level confounders. Overall, we observed highly concordant variance partition results between variance partition performed separately in each superclass and variance partition performed with all subclasses. The dominant source of variance remained the same as the CellType followed by the BrainDonor (Supplementary Fig. 6j). Spearman’s correlation of estimated variance between two models were high across all covariates, including brain donor, age, sex, ancestry and diagnosis (Supplementary Fig. 6k).

### *MAPT* locus haplotyping

From our harmonized genotype calls, we selected common variants in 17q21.31 locus (chromosome 17: 45307631–46836264), performed PCA analysis of genotypes using 10 PCs, and used *k*-means clustering with *k* = 3 to call three genotype clusters, *H1H1*, *H1H2* and *H2H2*. We additionally confirmed the *H1* haplotype using two published SNPs, rs17763050 and rs8070723, known to associate^34^. Haplotypes were estimated using Beagle (v.5.4)^158^ on the selected genotypes of the 17q21.31 region. The estimation of the initial haplotype frequency model converged after one burn-in iteration, and the estimate of the genotype phase converged after 23 phasing iterations. For testing association with PD diagnosis, we used logistic regression with age, sex, 10 genotype PCs, and *H1H1* status: PD ~ Age + Sex + Source + PC1 + PC2 + PC3 + PC4 + PC5 + PC6 + PC7 + PC8 + PC9 + PC10 + H1H1.

Moreover, we tested the contribution of the *H1* haplotype to AD among non-*APOE4* carriers^32^ but did not find a significant association (*P* ≤ 0.302). For testing association with AD diagnosis, we first subsetted for individuals who are not carriers of the *APOE4* allele and tested for AD association using logistic regression with the formula: AD ~ Age + Sex + Source + PC1 + PC2 + PC3 + PC4 + PC5 + PC6 + PC7 + PC8 + PC9 + PC10 + H1H1.

### Compositional variation analysis using crumblr

We applied the crumblr method (https://diseaseneurogenomics.github.io/crumblr) for testing the variation of cell type composition^159^, as reflected in Figs. 4a–c and 6a–c. In summary, Crumblr scales the cell count ratio (that is, fractions) data using centred log-ratio (CLR) transformation and applies linear models. As CLR-transformed data are still highly heteroskedastic, the precision of measurements varies widely. Crumblr uses a fast asymptotic normal approximation of CLR-transformed counts from a Dirichlet-multinomial distribution to model the sampling variance of the transformed counts. Crumblr enables incorporating the sampling variance as precision weights to linear (mixed) models to increase power and control the false-positive rate. Crumblr also uses a variance stabilizing transform based on the precision weights to improve the performance of PCA and clustering. Hypothesis testing was computed using the following formula: Cell composition ~ scale(Age) + Sex + (phenotype of interest).

By including these variables, we account for potential confounders and improve the accuracy and reliability of our hypothesis testing.

### Replication of compositional variation using SEA-AD MERFISH data

We downloaded MERFISH from the SEA-AD project as described in the original study^2^. To compare results to our current work, we first created a slightly modified cell type annotation. In brief, we retained the subclass annotation present in the original data but merged some subclasses if multiple annotations in the MERFISH data mapped to a single subclass in the current study (Supplementary Table 11). We then performed compositional analysis using crumblr in the MERFISH data using this partially merged subclass annotation, using the following formula: Cell composition ~ (1|Donor.ID) + Sex + Age.at.Death + PMI + Brain.pH + (trait).

We performed this analysis using three AD-associated traits found both in the current study and the SEA-AD data: (1) CERAD; (2) Braak; and (3) cognitive status. We then compared the estimated log-transformed fold changes associated with each trait and each pair of matching subclasses (Supplementary Table 11) between the two studies. To jointly test the concordance of the compositional changes for all three traits, we transformed all log-transformed fold change values with the rank-based inverse normal transform, then tested for concordance using a linear mixed model with the lmerTest R package, using the following formula: RankNorm(logFC1) ~ RankNorm(logFC2) + (1|trait).

### Validation of compositional variation using RNAscope

#### Tissue selection

We selected five AD and five neurotypical donors, matched for age and gender, and their DLPFC tissue blocks were obtained from the Mount Sinai Neuropathology Brain Bank and Research CoRE.

#### Probe design

We focused on four subclasses that showed robust variation in AD (Fig. 6a). Cell-type markers were selected on the basis of gene expression level and specificity (Fig. 2d and Supplementary Fig. 3i). The following probes were used: *CUX2* (RNAscope probe Hs-CUX2, 425581) for EN_L2_3_IT, *SST* (RNAscope probe Hs-SST-C2, 310591-C2) for IN_SST, *BMP5* (RNAscope probe Hs-BMP5-C3, 472461-C3) for VLMC and *MYOCD* (RNAscope probe Hs-MYOCD-C4, 416211-C4) and *CASQ2* (RNAscope probe Hs-CASQ2-C3, 447111-C3) for SMCs.

#### Cryosectioning

Before sectioning, frozen brain tissue blocks were allowed to equilibrate to the cryostat (CryoStar NX50, Thermo Fisher Scientific) chamber temperature. Tissue blocks were mounted to chuck with OCT (Tissue-Tek O.C.T. Compound, 4583, Sakura Finetek). After trimming, regions of interest (ROIs) with high quality and intact grey and white matter were selected and scored with razor blades. Sections were cut at 16 µm thickness and collected onto the slides by gently touching the sections with the slides and two sections were collected per donor. The sections were further adhered to the slides by drying at room temperature before sealed in slide mailers (Fisherbrand 5-Place Slide Mailer, Thermo Fisher Scientific) and stored at −80 °C until the RNAscope assay.

#### RNAscope assay and TrueBlack treatment pipeline

Sample preparation was performed according to the ACD RNAscope Multiplex Fluorescent v2 for Fresh Frozen tissues protocol (UM 323100/Rev B) with an extended C2 TSA dye incubation of 45 min (determined on the basis of pilot experiment testing). The sections were fixed in freshly made 10% formalin (formaldehyde solution, F8775-25mL, Millipore Sigma) in 1× PBS at 4 °C for 1 h, after which the sections were rinsed twice with 1X PBS. The sections were dehydrated by placing the slides into a series of diluted ethanol and finally 100% ethanol. Before the creation of hydrophobic barriers, slides were air dried and thick hydrophobic barriers were drawn around each section with the hydrophobic barrier pen (ImmEDG Hydrophobic Barrier Pen, NC9545623, Vector Laboratories). After the barriers were dried, hydrogen peroxide was applied to the sections and incubated for 10 min at room temperature; after the removal of the hydrogen peroxide, the sections were washed twice with distilled water. The sections were then treated with protease IV for 30 min at room temperature and washed with distilled water. To hybridize the gene-specific probes, the sections were incubated with diluted and mixed probes for 2 h at 40 °C, then the sections were washed twice with 1× wash buffer for 2 min at room temperature. The sections were either stored in 5× SSC overnight or immediately processed with AMP hybridization. During AMP hybridization, the sections were incubated sequentially with AMP1, AMP2 and AMP3 for 30 min each at 40 °C, with two washes using 1× wash buffer for 2 min between each incubation. After the AMP steps, fluorescence signal development was performed by incubating with HRP-C1 for 15 min at 40 °C. After two 2 min washes with 1× wash buffer, a diluted fluorophore was added and incubated for 30 min at 40 °C, followed by two 2 min washes with 1× wash buffer. After the washes, HRP blocker was applied for 15 min at 40 °C, followed by two final 2 min washes with 1× wash buffer. The same procedure was repeated for HRP-C2 and HRP-C3, each with distinct fluorophores. Nuclei were counterstained with DAPI for 30 s, and washed with 1× wash buffer. After the RNAscope assay, the samples underwent a TrueBlack (20× TrueBlack reagent, 23007, Biotium) treatment according to the Biotium TrueBlack Lipofuscin Autofluorescence Quencher Protocol 2: Post-treatment with TrueBlack protocol (PSF006). Buffers on the sections were carefully removed, and 1× TrueBlack in 70% ethanol was applied to completely cover the tissue sections. After 30 s of incubation, the sections were washed three times with PBS. Coverslip mounting was performed using ProLong Gold Antifade Mountant (P36930, Thermo Fisher Scientific).

#### Imaging

RNAscope results were acquired with EVOS M7000 at ×40 (OLY XAPO 40X NA0.95/WD0.18, AMEP4907, Thermo Fisher Scientific). For each section, 2–3 ROIs were captured, the selected ROIs covered the entire grey matter and approximately two-thirds of the white matter, with good tissue quality and well represented the whole section. In total, four channels were imaged for nuclei and the C1–3 of the RNAscope signal, with DAPI, GFP, RFP and CY5 fluorophore cubes. At ×40, the ROIs contain 100–200 single fields of view, which were captured with the scan area function of the EVOS M7000. The scan was performed with serpentine horizontal scan pattern and quick scan model with less overlapping areas. Auto focus on every field of the DAPI channel was chosen to gain proper focus. Single-field images were saved as 16-bit TIF files.

#### Image processing and result analysis

Individual images were stitched into the whole ROIs and four channel images of the same ROI were stacked together using a custom Fiji ImageJ macro using the Grid/Collection Stitching Plugin. For the DAPI channel, the Grid: snake by rows function was used, and the compute_overlap and subpixel_accuracy options were chosen. After the DAPI channel images were stitched, the resulting ‘TileConfiguration.registered.txt’ files were used as guidance to stitch images of the other channels to make the resulting ROI images having the same dimension. Whole-ROI images of four channels were stacked using the Images to Stack and Stack to Hyperstack functions of Fiji imageJ. To calculate the gene expression level at the single-cell level, all stacked whole-ROI images were further analysed in quPATH, by counting the number of RNAscope punctate signals inside segmented cell borders. quPATH annotation was drawn to cover the whole ROI while avoiding areas of poor tissue quality or high-fluorescence background. The cells in the ROIs were segmented on the basis of DAPI nucleus signals with the cell detection function of the quPATH. Next, RNAscope punctate signals were detected by the subcellular detection function of the quPATH. The results were saved as csv files with the saveDetectionMeasurements function of the quPATH. To compare the difference in cell composition between the AD and control donors, a custom Python script was developed to quantify the cells of a certain cell type. The threshold of a positive cell is determined by evaluating the distribution of the cell and also by inspecting the images. The cell type was determined on the basis of the number of clusters detected for each channel (2 for *CUX2*, 0 for *SST* and *BMP5*). We observed a higher sensitivity for *CUX2*, so we used a higher minimum threshold to compensate. For each sample, cell counts and fraction were calculated. Cell fractions were transformed using the CLR method, which divides each part of the composition by the geometric mean of all parts and then takes the logarithm of the resulting ratio. Statistical differences between AD cases and controls (*n* = 10) were assessed using the Mann–Whitney–Wilcoxon test.

### Differential gene expression analysis using Dreamlet

Owing to the increased variable complexity in a large-scale disease atlas, scaling single-cell based approaches to millions of cells across a wide range of phenotypes presents computational challenges^160^ and can be suboptimal^161–164^. To account for the scale of these data, complex study designs with repeated measures, and low read count per cell, we applied Dreamlet for differential expression analysis, which applies a pseudobulk approach, as reflected in Figs. 5a–c and 6d,e. Building from the previously developed statistical tool Dream^165^, it applies linear mixed models to the differential expression problem in single-cell omics data. It starts by aggregating cells by the donor using a pseudobulk approach^161,162^ and fits a regression model. For each feature and cell cluster, the following mixed model was applied: Gene expression ~ scale(Age) + Sex + scale(PMI) + log(n_genes) + percent_mito + mito_genes + mito_ribo + ribo_genes + (phenotype of interest), where categorical and numerical variables were modelled as random and fixed effects, respectively. If the phenotype of interest was a categorical variable, we set the intercept as 0 and used predefined contrasts between two factors. We performed a gene set analysis using the full spectrum of gene-level *t*-statistics using Zenith^166^.

#### Note on the *APOE* genotype

We considered including the *APOE* genotype as a covariate in the differential expression analysis of AD, but this was not supported by the data. First, performing differential expression analysis on the basis of the *APOE* genotype did not identify any genes as differentially expressed at a study-wide FDR of 5%. Second, we then included the *APOE* genotype as a covariate in the differential expression analysis of AD versus controls. The estimated effect sizes for AD were very similar when comparing models with and without the *APOE* genotype (Supplementary Fig. 13a). No genes were significantly different in estimated effect size between the two models. On the basis of these empirical results, our analysis excluded the *APOE* genotype in the model.

### Meta-analysis between brain sources

We conducted a meta-analysis to integrate results from different brain banks for the same disorder. Data tables from multiple brain banks were combined into a single list for each disorder and annotated with their respective sources. The meta_analysis() function in dreamlet was used to perform the meta-analysis, which involved combining data tables into a single data frame, grouping the data by assay. The s.e. was computed from the estimated log-transformed fold change and moderated *t*-statistics according to s.e. = log[FC]/*t*. The meta-analysis was performed using the rma() function from the metafor package^167^ with a fixed-effects model. *P* values were adjusted using the FDR method, and the −log_10_ of the FDR values were calculated. This method was applied to datasets of AD, DLBD, Vas, PD, Tau, FTD, SCZ and BD.

### Meta-analysis across the same disease category

To further synthesize findings across multiple disorders, a meta-of-meta analysis was conducted, grouping the disorders into neurodegenerative and neuropsychiatric categories. The results from the initial meta-analyses for each disorder were combined into lists on the basis of their categories. The meta-of-meta function was used to perform this higher-level analysis. This function combined the meta-analysis results into a single data frame, grouped the data by assay and calculated the s.e. values using the formula abs(estimate/statistic). This approach was applied to create meta-of-meta analyses for all disorders, neurodegenerative disorders and neuropsychiatric disorders.

### Evaluation of shared disease signatures

We modelled the total disease signature for a given condition as the sum of shared and disease-specific DEGs:

$$\Delta {\tau }_{k}=\sum _{i\in {G}_{\mathrm{shared}}}\Delta {\tau }_{i,k}+\sum _{j\in {G}_{\mathrm{distinct}}}\Delta {\tau }_{j,k}$$

where Δ*τ*_*k*_ denotes the total disease signature for a disease *k*, *G*_shared_ denotes a set of genes having cross-disorder effects, and *G*_distinct_ denotes a set of genes with non-shared (disease-specific) effects. This notation highlights that DEG profiles for each disease arise from contributions of both shared genes (common to all diseases) and distinct genes (specific to each disease).

To determine how DEG effects are shared across cell types and disease, we applied a multivariate Bayesian meta-analysis approach using the mashr software^48^. The software uses a Bayesian approach to shrink effect sizes across genes and cell types to estimate the posterior effect sizes and posterior probability that an effect has the correct sign. In accompanying work, we have extended this approach to develop a formal statistical test to identify cell-type- and disease-specific effects^168^. For this analysis, we conducted composite tests in a cell-type-specific manner, assessing the probability that a gene exhibits a non-zero effect across all eight disease contrasts. Genes with a posterior probability of ≥0.05 were classified as part of the shared component.

### Construction of the correlation matrix

To calculate the correlation matrix, we used a systematic approach to quantify the relationships between genetic estimates across different neuropsychiatric and neurodegenerative disorders. Spearman’s correlation coefficients were calculated to assess the strength and direction of association between genetic estimates across different disorders. The calculation was performed for the common to each pair of disorders, grouped by assay, after the exclusion of the shared disease signatures.

The wide-format correlation matrix was converted to a matrix suitable for heat-map visualization. Missing values were replaced with zeros. Annotations indicating the number of significant genes were added to the rows and columns using the rowAnnotation and HeatmapAnnotation functions of the ComplexHeatmap package. The heat map was generated with hierarchical clustering of both rows and columns, and coloured on the basis of the Spearman’s correlation values using a gradient from blue (negative correlation) to red (positive correlation).

### Co-heritability analysis

We used cross-trait LD score regression using the LDSC tool^169^ to estimate the genetic correlation between a pair of traits. We used summary stats for the following GWAS traits (AD^96^, DLBD^170^, PD^98^, FTD^171^, SCZ^103^, BD^104^) and calculated the heritability for each of the traits and the genetic covariance and correlation between each of the pair of traits (in total 15 pairs of traits). The size of the cohort was provided to the function munge_sumstats.py for heritability estimates. Precomputed LD scores for 1000 Genomes EUR data were downloaded from https://data.broadinstitute.org/alkesgroup/LDSCORE/eur_w_ld_chr.tar.bz2. The SNP list for munge_sumstats.py was downloaded from https://data.broadinstitute.org/alkesgroup/LDSCORE/w_hm3.snplist.bz2. Standard error was obtained from the LDSC output. The script munge_sumstats.py was modified to include the parameter --chunksize 5e5.

For each of the 15 possible combinations of traits, we calculated the level of correlation of gene expression using the Spearman rank correlation test. Genes were selected by applying the following criteria: log_2_[FC] ≥ 0.5, FDR < 0.05. Co-expression coefficient was calculated for the overall dataset and for each of the cell types. Next, to correlate co-expression and co-heritability, we calculated the Spearman rank correlation coefficient between the LDSC genetic correlation score and the co-expression coefficient using 15 possible combinations of traits as datapoints for the Spearman rank correlation. Spearman was calculated for the overall dataset providing the genetic estimates of the expression similarities in the PsychAD cohort, and also per cell type to obtain the ranking of the cell types that contribute the most to the genetic to transcriptomic similarity in PsychAD.

### Causal mediation analysis

#### Methods

Causal mediation analysis was performed on a subset of 645 individuals with European ancestry in AD contrast (tier 2, *n* = 696), who have PRS calculations from the latest AD GWAS^96^. Two different R packages were used, namely mediation (https://cran.r-project.org/web/packages/mediation/) and psych (https://cran.r-project.org/web/packages/psych/). The results were cross-checked between the two methods (identical within a threshold) to ensure the estimated coefficients, and the mediation effects are statistically robust.

#### Statistical assumptions

A key assumption in causal mediation analysis is that there are no unobserved confounding effects between the mediator and the outcome. To address this, we carefully selected and included covariates in our model that are known or suspected to confound the mediator–outcome relationship. Furthermore, we ensured that the dependent, independent, and mediator variables were continuous, normally distributed and linearly related. For each regression, we used the following covariates: Age + Sex + PMI + PC1 + PC2 + PC3 + PC4 + PC5 + PC6 + PC7 + PC8 + PC9 + PC10, where PC1–10 indicate the genotype PCs. For the variation of subclass-level cell type composition, we used CLR-transformed cell count fractions from the crumblr compositional analysis. For bootstrapping, we used 10,000 simulations with the 50th percentiles of the treatment variable used as the control condition and the 90th percentile of the treatment variable used as the treatment condition, similar to a previous publication^172^.

#### Sensitivity analysis

We performed additional sensitivity analyses to strengthen the robustness of the mediation analysis. To ensure that the mediation effect was specific to AD, we performed the causal mediation analysis using PRSs derived from other neurological diseases (PD, MS, ALS, SCZ, BD and ASD). We found that none of these alternative PRSs yielded significant mediation effects (Supplementary Table 13), supporting the specificity of our findings to AD.

### Trajectory analysis using neural network models

#### Rationale

Traditionally, changes in gene expression as a function of disease state are measured using linear-based models. This approach has proven to be highly valuable and has enhanced our understanding of the biological mechanisms underlying many diseases. However, it is increasingly recognized that changes in gene expression can be highly nonlinear^66^; the interaction among numerous signalling pathways, many involving multiple feedback loops, can lead to complex dynamics that linear models may fail to capture. One approach to capturing potentially nonlinear changes in gene expression is pseudotime analysis (that is, trajectory inference)^173,174^. Most trajectory inference tools rely on identifying continuous transitions within the dominant sources of variation in the data. However, as described in Fig. 3a, the variance in gene expression attributable to disease state is dwarfed by variance introduced by inter-donor heterogeneity, sex, brain source, and other technical or biological confounders. When disease related variance is not the principal axis of variation, these tools may construct trajectories that primarily reflect confounding factors rather than true disease progression. To address this limitation, we trained neural network models in a supervised manner that directly estimates the state of disease progression from single-cell gene expression data. In contrast to unsupervised trajectory inference methods, our approach explicitly models the relationship between gene expression and disease labels (for example, Braak stage and dementia status), enabling us to assign each cell a continuous disease progression score. This enables more accurate isolation and sensitive detection of disease-related signals, even when they account for a relatively small fraction of the overall variance.

Furthermore, in the case of AD, it is known that the spread of NFTs and Aβ plaques is strongly, but not perfectly, correlated with dementia. Of special interest is understanding cases in which individuals are resilient to dementia despite a high NFT or Aβ plaque burden. Thus, we aimed to disentangle changes in gene expression associated with disease burden from those associated with dementia. Standard trajectory inference methods do not allow us to separate these two covariates effectively. In our approach, we trained the neural network model by equally sampling all combinations of Braak stage and dementia status, thereby discouraging the model from learning spurious correlations between the two target variables. The model’s predictions of the Braak stage and dementia status were then used as two independent pseudotime axes.

#### Model architecture

The neural network model was a relatively simple feedforward network with two hidden layers. The input to the neural network were the log1p transformed gene counts from individual cells. Each hidden layer consisted of 1,024 units using the ReLU activation function. Layer and batch normalization were not used. The output of the network were Braak stage and the dementia status predictions.

#### Model training

Single-cell gene counts from the top 10,000 protein coding genes for each cell class, based on the percentage of cells the gene was expressed in, were used for training. We found that overfitting became more problematic when using a greater number of genes (data not shown). Genes found on sex chromosomes were excluded to discourage the model from learning to associate Braak or dementia status with sex. The network was trained to minimize the loss for both the Braak and dementia outputs. For the binary target dementia, the softmax function was applied to the output, and the loss was the cross-entropy. For Braak, target values were first normalized to zero mean and unit s.d., and the mean-squared error loss was used. Both loss terms were trained simultaneously. To prevent the model from overfitting the data, we applied dropout with probability of 0.5 to all hidden layers (after the ReLU activation). For each cell class, we divided the cells into 20 splits. In each split, ~95% of the cells was used for training, and the remaining ~5% was used for inference. Within each split, cells from a single donor exclusively belonged to either the training set or the inference set, but never both. Thus, model predictions were always based on cross-validated data from different donors. We trained one model for each of the 20 splits to generate predictions for all donors for that cell class. Models were trained using all donors from the MSSM and RADC brain banks and then ran inference on the 696 donors that focused on the AD phenotype contrast (Fig. 1d). There were different numbers of cells for each cell class; thus, the amount of training differed between classes. For neurons, astrocytes, oligodendrocytes and the immune cell class, we trained for 20 epochs. For mural and endothelial cells, which contained less data, we trained for 100 epochs. As model accuracy for the OPC cell class evolved more slowly over model training, we trained for 30 epochs. Cross-validated model accuracy across model training is shown in Supplementary Fig. 29. The models were trained using stochastic gradient descent (SGD) without momentum and with a learning rate of 0.02. We found that training with SGD led to greater accuracy compared with adaptive optimizers such as ADAM (data not shown). We used a batch size of 256 and linearly increased the learning rate across 2,000 training steps until it reached 0.02. Finally, we clipped the gradient norm to 1.0 to stabilize training. The model accuracy was determined as follows. We averaged the cell-level Braak and dementia model predictions to obtain donor-averaged scores. For dementia, we calculated the balanced classification score by determining the percentage of donors without dementia with a prediction score ≤ 0.5, the percentage of donors with dementia with a prediction score > 0.5 and then averaging these two values. For Braak, we calculated the Pearson *R* value between the actual Braak stage and the Braak predictions. Error bars were generated using a bootstrap procedure, in which we randomly sampled donors (with replacement), calculated the Braak and dementia prediction accuracy, and repeated this process 20,000 times. For each cell class, we included all donors with at least five cells. The number of donors with at least five cells and a defined Braak stage/Dementia status for each cell class was as follows: EN, 680/650; IN, 684/653; Astro, 692/662; Immune, 685/655; Oligo, 688/657; OPC, 688/659; Mural, 663/634; Endo, 589/560.

#### Disentangling Braak and dementia

Braak stage and dementia status are significantly correlated (Pearson *R* = 0.582, *P* < 1 × 10^−60^). This strong correlation between target variables implies that input features (that is, changes in gene expression) associated with the two target variables are also likely to be correlated, which makes it challenging for the model to learn which input feature is predictive of which target variable. The result is when the model is trained in a standard manner, the Pearson correlation between the Braak and dementia model predictions is ~0.94 at the donor-level (Supplementary Fig. 14a; immune class shown), suggesting that the model has learned spurious correlations between the input features and target variables. Fully removing spurious correlation in machine learning models is still an unresolved question. However, balancing the training data, such that each of the 14 combinations of Braak and dementia (7 Braak values 2 dementia values) are equally sampled, can effectively reduce spurious correlations learned during training^175^. In practice, we equally sampled from 15 groups, in which the extra group consisted of donors whose Braak stage or dementia status had not been determined. Training with group balancing reduced the correlation between the predicted Braak and dementia values (Supplementary Fig. 14b), did not adversely affect the model accuracy at the donor-level (Supplementary Fig. 14d).

#### Calculating gene trajectories

We wished to measure how gene expression varied as a function of the predicted Braak stage. First, gene counts were normalized so that each cell’s total count was 10,000, followed by the log1p transformation. Second, for each cell class and each donor, we calculated the mean predicted Braak stage (termed disease pseudotime), and the mean-normalized expression for each gene. Averaging within each donor reduced variability and ensured that donors with greater cell counts did not contribute disproportionately to downstream analysis. Third, we smoothed both the predicted donor-averaged disease pseudotime, and the donor-averaged gene expression with a Gaussian kernel. Specifically, for each donor *i*, we weighted all other donors *j* as

$${w}_{i,j}={Z}_{i}\exp ({({p}_{j}-{p}_{i})}^{2}/2{\sigma }^{2})$$

where *p*_*i*_ is the disease pseudotime of donor *i*, *σ*^2^ was set to an eighth (1/8) of the variance of the disease pseudotime distribution and the normalization term *Z*_*i*_ was set such that ${\sum }_{j}{w}_{i,j}=1$. This enabled us to calculate smoothed disease pseudotime, $\widetilde{{p}_{i}}$, and smoothed gene expression, $\widetilde{{g}_{i}}$, values

$$\widetilde{{p}_{i}}=\sum _{j}{w}_{i,j}{p}_{j}$$

$$\widetilde{{g}_{i}}=\sum _{j}{w}_{i,j}{g}_{j}$$

where *g*_*j*_ is the gene expression vector of log1p normalized counts for donor *j*. After ordering the smoothed disease pseudotime values, gene trajectories are now represented as the tuple $(\widetilde{{p}_{i}},\widetilde{{g}_{i}})$. We only included donors with at least five cells for the cell class

#### Resilience against dementia

As tau proteinopathy and dementia status are highly correlated, gene expression as a function of the two variables is also correlated, and therefore partially redundant. We therefore aimed to measure how gene expression covaried with predicted dementia given the predicted Braak staging. To do so, we first calculated the expected predicted dementia and expected gene expression for donors with similar disease pseudotime. Specifically, we defined the expected dementia given disease pseudotime, ${d}_{i}^{p}$, and the expected gene expression given disease pseudotime, ${g}_{i}^{p}$,

$${d}_{i}^{p}=\sum _{j}{w}_{i,j}{d}_{j}$$

$${g}_{i}^{p}=\sum _{j}{w}_{i,j}{g}_{j}$$

where *d*_*j*_ is the predicted dementia status. As above, *w*_*i*,*j*_ was calculated on the basis of the difference in disease pseudotime between donors *i* and *j*, except that we set *w*_*i*,*i*_ = 0 so that each donor does not contribute to its own expected value. We then calculated the residuals between the donor’s predicted dementia status and gene expression with its expected values:

$${d}_{i}^{R}={d}_{i}-{d}_{i}^{p}$$

$${g}_{i}^{R}={g}_{i}-{g}_{i}^{p}$$

The dementia resilience score for each donor was then the product of these two terms. When calculating early and late resilience, donors were separated into early and late groups on the basis of disease pseudotime before averaging within each group. Using this metric, we define genes as protective if gene expression increases as predicted dementia decreases, given the disease pseudotime (that is, the product of the terms defined above is negative). Conversely, we define genes as damaging if gene expression increases as predicted dementia increases, given the disease pseudotime.

#### Identifying trajectory transition points

We sought to determine whether specific points during disease progression corresponded to shifts in gene dynamics. First, we included all donors with at least five cells in each of the eight cell classes (*n* = 578 donors). Next, we concatenated the smoothed trajectories of all coding genes across the eight classes (*n* = 17,265 genes × 8 cell classes) and performed PCA of the resulting 578 × 138,120 donor-by-gene/cell matrix. The trajectory projected onto the first three PCs, which together explain more than 91% of the variance, is shown in Supplementary Fig. 16, where the hue represents the disease pseudotime prediction averaged across all cell types for each donor. Visual inspection suggests points at which the trajectory changes direction. To quantify these transitions, we fit the trajectory projection (using the first three PCs) with a piecewise linear function consisting of two domains. Each domain represents a continuous block of donors ranked by mean disease pseudotime, separated by a transition point *n* (where *n* is the rank of the last donor in the first block). We calculated the explained variance for the piecewise fit across all possible transition points. The transition point that maximized explained variance was identified at *n* = 225 (out of 578 donors), which we define as the early-to-late transition point (Supplementary Fig. 16a (magenta circle)). A second, earlier transition is visible in the trajectory projection onto PC1 and PC3, although PC3 explains significantly less variance than PC2, making this shift weaker. To identify this preliminary transition, we repeated the same analysis using only the first 225 donors. This identified an earlier transition at *n* = 53 (out of 578 donors), marked by the cyan circle in Supplementary Fig. 16a. In subsequent analyses using these transition points to characterize dynamics within individual cell classes (Fig. 7), we adjusted the transition values proportionally on the basis of the number of donors available for each class. For example, in the EN cell class, 682 donors have at least five EN cells. To determine the early-to-late transition point for this class, we scaled the original transition point (225 out of 578 donors) according to the total number of EN donors: (682 × 225)/578 ≈ 265, rounding down.

#### Trajectory nonlinearity

We sought to determine whether gene trajectories were more nonlinear in certain cell classes than others. To assess this, we first fit the trajectory of all coding genes with a linear model and calculated the mean explained variance for each cell class (blue bars in Supplementary Fig. 17a). Next, we applied a piecewise linear fit, systematically varying the transition point across donors (as described in the ‘Identifying trajectory transition points’ section). For each cell class, we selected the transition point that maximized the mean explained variance across all coding genes. The explained variance at these optimal transition points is shown with orange bars in Supplementary Fig. 17a. To quantify trajectory nonlinearity, we defined a nonlinearity index as the difference between the explained variance of the optimal piecewise fit and that of the single linear fit, averaged across all coding genes (Supplementary Fig. 17b). Finally, we compared the nonlinearity index between neuronal and non-neuronal cell classes using a Wilcoxon rank-sum test.

#### Trajectory gene enrichment

We wished to determine which genetic pathways were most significantly up or downregulated during the progression of AD. To do so, we first extracted the slopes of the early and late linear fits for the Braak trajectories, and the mean early and late resilience scores (defined above). We used these slopes input to Zenith (https://bioconductor.org/packages/release/bioc/html/zenith.html) to calculate the changes across all GO BP pathways across the eight cell classes. For each GO BP pathway, we calculated the minimum Zenith FDR across the eight cell classes and across early and late stages, for both disease pseudotime and resilience. Only pathways with a minimum FDR ≤ 0.01 were included. Furthermore, we included only pathways with at least ten genes to ensure that the results were statistically robust, and no more than 250 genes to ensure that the pathways were not overly broad. Next, as we were interested only in pathways that could be informative of the mechanisms underlying AD progression, we excluded pathways containing words referring to overly broad behaviours or cognitive functions (learning, memory, vocalization, social, auditory, startle response, behaviour, locomotor, startle, prepulse inhibition), terms referring to anatomical structures other than the cortex (substantia nigra development, cardiac, coronary, aortic, ventricular, kidney, metanephric, retina, optic, bone, respiratory, pulmonary, olfactory, sperm, placenta, egg, embryonic, ovulation, estrous, placenta, sperm, mamary, germ layer, outflow tract septum, adrenal, epithelial, skeletal, otic, head) or overly broad neural terms (nervous system process, cerebral cortex, recognition, host, organ, developmental growth). To condense the remaining pathways into a more manageable size, we used rrvgo (https://www.bioconductor.org/packages/release/bioc/html/rrvgo.html). We selected the Wang semantic similarity metric^176^, and set the threshold at 0.8 to obtain 86 GO BP pathways (Supplementary Fig. 18). For easier visualization, we selected 32 representative pathways from this set for Fig 7e. We also performed similar steps to obtain the top GO Molecular Function (MF) and Cellular Component (CC) pathways (Supplementary Figs. 19 and 20). As above, only pathways with a minimum FDR ≤ 0.01 were included, but the rrvgo threshold was set to 0.5 as there were fewer significant pathways.

#### Comparison to PLS regression

To compare our neural network approach with linear regression, we applied partial least squares (PLS) regression (using sklearn.cross_decomposition.PLSRegression) to predict Braak stage and dementia status in the immune cell class. As with our neural network models, the PLS model was jointly trained to predict Braak and dementia across 20 train/test splits, ensuring that predictions were generated on donors that were not included in model training. We empirically determined that setting n_components to 10 yielded the highest cross-validated accuracy. The PLS model achieved slightly greater Braak prediction accuracy than the neural network model, although the difference was not statistically significant (*P* > 0.05, bootstrap; Supplementary Fig. 15a). By contrast, the dementia classification accuracy was significantly lower (*P* < 0.001, bootstrap). However, we note that training a regression model on a binary classification task is not ideal, and improvements to this approach are possible. Moreover, PLS predictions for Braak and dementia were significantly more correlated than those from the neural network (Supplementary Fig. 15b). As Braak stage and dementia status are correlated, models that fail to disentangle their effects may overlook genes with opposing expression patterns. For example, if a gene’s expression increases with the Braak stage but decreases with dementia, these opposing trends could cancel out, rendering the change statistically insignificant. To evaluate this, we performed gene set enrichment analysis using Zenith on the early disease stage for both models, considering pathways for which the Zenith FDR was below 0.05 for either disease pseudotime or resilience. In Supplementary Fig. 15c, we compare the disease pseudotime Zenith *z* scores from the PLS and neural network models. The left panel shows pathways with concordant expression patterns (for example, increasing with Braak and associated with higher dementia risk), while the right panel highlights pathways with divergent patterns (for example, increasing with Braak but associated with lower dementia risk). For pathways with divergent trends, the PLS model yielded a lower Zenith *z* score magnitude. Finally, Supplementary Fig. 15d presents the top ten pathways with the greatest negative difference in *z* scores between the PLS and neural network models. These include key immune response pathways implicated in the early stages of AD.

#### Comparison to traditional trajectory inference methods

We wished to compare the accuracy of our neural network model predictions against two traditional trajectory inference methods: Monocle 3 (ref. ^177^) and Palantir^177,178^. For Monocle 3, we use the Python wrapper py-monocle (https://github.com/bioturing/py-monocle). We compared our method against these alternative approaches on the subset of immune cells consisting of microglia; we excluded adaptive and PVM cells as their different expression compared to microglia could be problematic for traditional trajectory inference approaches. The UMAP was computed as described in the ‘Defining cellular taxonomy using iterative clustering’ section. For both Monocle 3 and Palantir, we randomly selected a starting cell from among those with donor metadata indicating Braak stage = 0, CERAD score = 1, and no dementia or MCI. Trajectory inference was then performed, and the Pearson correlation between the resulting pseudotime and the actual Braak stage was calculated. This process was repeated 200 times, and the starting cell yielding the highest correlation was retained. The results in Supplementary Fig. 27a show the mean actual Braak, neural network model predicted Braak, Monocle3 pseudotime and Palantir pseudotime across UMAP space. The Pearson correlation between the actual Braak and model predictions or trajectory inference pseudotime values are shown in Supplementary Fig. 27b. Although it may be possible to optimize trajectory inference performance by refining hyperparameters, selecting different starting points or improving the underlying UMAP embedding, it is unclear whether such improvements would be sufficient to surpass the predictive accuracy of our neural network model (Supplementary Fig. 27b). Moreover, it is not evident how traditional trajectory inference approaches could be adapted to disentangle Braak stage from dementia status—an aspect our model addresses directly.

#### Pathway enrichment without smoothing

To ensure that our results were not biased by data smoothing (as described in the ‘Calculating gene trajectories’ section), we repeated our pathway enrichment analysis without smoothing gene trajectories. We maintained the same early-to-late transition point identified in Fig. 7c, which was derived from smoothed trajectories. To compute unsmoothed gene expression changes, we grouped donors into early and late stages on the basis of their mean disease pseudotime predictions. We then calculated slopes between donor-averaged gene expression and donor-averaged disease pseudotime predictions, without smoothing either variable. For resilience scores, we computed the partial covariance between donor-averaged gene expression and dementia predictions, regressing out disease pseudotime predictions. As before, these calculations were performed separately for early- and late-stage donors. Supplementary Fig. 28 compares Zenith *z* scores from the smoothed (*x* axis) and unsmoothed (*y* axis) approaches. Each dot represents a GO BP pathway, and we only included pathways in which the unsmoothed Zenith FDR was below 0.05. Rows correspond to cell classes, while columns compare early and late gene expression changes and resilience scores. Pearson correlations for increasing versus decreasing, or protective versus damaging pathways, ranged from 0.843 to 0.996 (median = 0.981). These results suggest that our findings are not an artifact of oversmoothing.

#### Identifying putative causal pathways

To identify GO BP pathways enriched in AD risk genes, we compared the *z* scores from the Alzheimer’s GWAS^96^ between genes within each pathway and all other protein-coding genes not in the pathway. This was assessed using a one-sided *t*-test, and pathways with *P* values below 0.01 were considered to be potentially causal.

#### Mean trajectories and MAGMA enrichment

For both the mean normalized expression (Fig. 7f) and Magma enrichment analysis (Fig. 7h), we used the top 250 coding genes based on the early and late slopes of the Braak trajectories. Late-decreasing genes tend to also appear to contain an early increase (Fig. 7f). However, we cannot say whether this is biologically meaningful or the result of selection bias, as a strong late decrease must be preceded by a high baseline. For both the mean expression and MAGMA enrichment calculations, results were qualitatively similar if we used the top 500 or 1,000 genes instead (data not shown).

#### Immune cell dynamics

To further characterize how the immune response evolves during disease progression, we performed pathway enrichment using a sliding-window analysis. We sorted donors on the basis of their mean disease pseudotime predictions and calculated the change in pathway expression using the smoothed gene trajectories using windows of 60 donors and shifting the window by 20 donors at each interval. The Zenith *z* scores were calculated for each window of 60 donors. We included only pathways for which the Zenith FDRs were below 0.01 for at least 5 windows and included all pathways with between 8 and 250 genes. Furthermore, we only included putatively causal pathways (see the ‘Identifying putative causal pathways’ section), in addition to an immune and metabolic pathway that were presented in Fig. 7e (cytoplasmic translation and detection of bacterium). Using the mean disease pseudotime value from the midpoint of each window, we linearly interpolated the z-scores to obtain 100 values evenly spaced across the disease pseudotime axis. The vertical lines indicating the preliminary and early-to-late transition points were obtained from the PCA analysis in Supplementary Fig 16.

### Ethics oversight

All procedures and research protocols were approved by the respective ethical committees of our collaborator’s institutions. The Ethics committee/IRB of Mount Sinai gave ethical approval for this work. The ethics committee/IRB of James J. Peters Department of Veterans Affairs Medical Center gave ethical approval for this work. The ethics committee/IRB of Rush Alzheimer’s Disease Center gave ethical approval for this work. The ethics committee/IRB of National Institute of Mental Health Human Brain Collection Core gave ethical approval for this work.

### Reporting summary

Further information on research design is available in the Nature Portfolio Reporting Summary linked to this article.

## Online content

Any methods, additional references, Nature Portfolio reporting summaries, source data, extended data, supplementary information, acknowledgements, peer review information; details of author contributions and competing interests; and statements of data and code availability are available at https://doi.org/10.1038/s41586-025-09573-z.

## Supplementary information

Supplementary Figs. 1–30 Supplementary Figs. 1–30.

Reporting Summary

Supplementary Tables 1–13 Supplementary Tables 1–13.

Supplementary Data 1 Metadata and model predictions for each cell. Each cell class contains a table that includes the cell barcode, donor information, the cell subclass and both the actual Braak and dementia status along with the model predictions.

Peer Review File

## Data Availability

Single-nucleus transcriptomics data are available through the AD Knowledge Portal (https://adknowledgeportal.org). The AD Knowledge Portal is a platform for accessing data, analyses and tools generated by the Accelerating Medicines Partnership (AMP-AD) Target Discovery Program and other National Institute on Aging (NIA)-supported programs to enable open-science practices and accelerate translational learning. The data, analyses and tools are shared early in the research cycle without a publication embargo on secondary use. Data are available for general research use according to the following requirements for data access and data attribution (https://adknowledgeportal.synapse.org/Data%20Access). For access to content described in this Article, see https://doi.org/10.7303/9618136. The data are available under controlled use conditions set by human privacy regulations. To access the data, a data-use agreement is needed. The registration is in place solely to ensure the anonymity of the study participants. Moreover, we have a data descriptor manuscript^129^ detailing the data processing and data collection. Interactive visualization of the single-cell data is available from CELLxGENE (https://cellxgene.cziscience.com/collections/84ce6837-548d-4a1f-919f-0bc0d9a3952f). Xenium in situ spatial transcriptomics data are available at Zenodo^179^ (https://doi.org/10.5281/zenodo.14606776). Additional phenotypic data can be requested at www.radc.rush.edu.

## Code availability

All the source codes used in this study are available at GitHub (https://github.com/DiseaseNeuroGenomics/PsychADxD).
