# Supplementary material for: Single-cell atlas of transcriptomic vulnerability across brain disorders
Source: Nature. 2026 Sep 23;657(8133):988–1002. doi: 10.1038/s41586-025-09573-z (PMC13600946; doi:10.1038/s41586-025-09573-z)
Supplement: Supplementary file 1 [file 41586_2025_9573_MOESM2_ESM.pdf]

nature portfolio

Corresponding author(s): Donghoon Lee; Panos Roussos

Last updated by author(s): Jul 18, 2025

Reporting Summary

Nature Portfolio wishes to improve the reproducibility of the work that we publish. This form provides structure for consistency and transparency in reporting. For further information on Nature Portfolio policies, see our Editorial Policies and the Editorial Policy Checklist.

Statistics

For all statistical analyses, confirm that the following items are present in the figure legend, table legend, main text, or Methods section.

n/a	Confirmed	
☐	☒	The exact sample size (n) for each experimental group/condition, given as a discrete number and unit of measurement
☐	☒	A statement on whether measurements were taken from distinct samples or whether the same sample was measured repeatedly
☐	☒	The statistical test(s) used AND whether they are one- or two-sided<br>*Only common tests should be described solely by name; describe more complex techniques in the Methods section.*
☐	☒	A description of all covariates tested
☐	☒	A description of any assumptions or corrections, such as tests of normality and adjustment for multiple comparisons
☐	☒	A full description of the statistical parameters including central tendency (e.g. means) or other basic estimates (e.g. regression coefficient) AND variation (e.g. standard deviation) or associated estimates of uncertainty (e.g. confidence intervals)
☐	☒	For null hypothesis testing, the test statistic (e.g. F, t, r) with confidence intervals, effect sizes, degrees of freedom and P value noted<br>*Give P values as exact values whenever suitable.*
☐	☒	For Bayesian analysis, information on the choice of priors and Markov chain Monte Carlo settings
☐	☒	For hierarchical and complex designs, identification of the appropriate level for tests and full reporting of outcomes
☐	☒	Estimates of effect sizes (e.g. Cohen's d, Pearson's r), indicating how they were calculated

Our web collection on statistics for biologists contains articles on many of the points above.

Software and code

Policy information about availability of computer code

Data collection	FACSDiva software, version 8.0.2 (BD Biosciences)
Data analysis	All the source codes used in this study are available via GitHub: https://github.com/DiseaseNeuroGenomics/PsychADxD. Python codes require Python >= v3.8. R codes require BioC v3.17 for R >= v4.3.0. The following versions of software were used for data analysis: anndata v0.9.1, aplot v0.2.0, Biobase v2.60.0, BiocGenerics v0.46.0, BiocParallel v1.34.2, cowplot v1.1.1, crumblr v0.99.8, dplyr v1.1.2, dreamlet v0.99.25, GenomeInfoDb v1.36.1, GenomicRanges v1.52.0, ggplot2 v3.4.3, ggtree v3.8.2, IRanges v2.34.1, limma v3.56.2, louvain v0.7.1, MatrixGenerics v1.12.3, matrixStats v1.0.0, numpy v1.24.4, pandas v1.5.0, pynndescent v0.5.6, python-igraph v0.9.10, RColorBrewer v1.1-3, S4Vectors v0.38.1, scanpy v1.9.3, scikit-learn v1.3.0, scipyv1.10.1, SingleCellExperiment v1.22.0, statsmodels v0.14.0, SummarizedExperiment v1.30.2, tidyr v1.3.0, umap v0.5.3, variancePartition v1.31.15, zellkonverter v1.10.1, zenith v1.2.0

For manuscripts utilizing custom algorithms or software that are central to the research but not yet described in published literature, software must be made available to editors and reviewers. We strongly encourage code deposition in a community repository (e.g. GitHub). See the Nature Portfolio guidelines for submitting code & software for further information.

Data

Policy information about availability of data

All manuscripts must include a data availability statement. This statement should provide the following information, where applicable:

- Accession codes, unique identifiers, or web links for publicly available datasets
- A description of any restrictions on data availability
- For clinical datasets or third party data, please ensure that the statement adheres to our policy

Single nucleus transcriptomics data is available via the AD Knowledge Portal (https://adknowledgeportal.org). The AD Knowledge Portal is a platform for accessing data, analyses, and tools generated by the Accelerating Medicines Partnership (AMP-AD) Target Discovery Program and other National Institute on Aging (NIA)-supported programs to enable open-science practices and accelerate translational learning. The data, analyses, and tools are shared early in the research cycle without a publication embargo on secondary use. Data is available for general research use according to the following requirements for data access and data attribution (https://adknowledgeportal.synapse.org/Data%20Access). For access to content described in this manuscript see: https://doi.org/10.7303/syn60084804. The data are available under controlled use conditions set by human privacy regulations. To access the data, a data use agreement is needed. The registration is in place solely to ensure the anonymity of the study participants. In addition, we have a data descriptor manuscript129 detailing the data processing and data collection. Interactive visualization of the single-cell data is available from CELLxGENE https://cellxgene.cziscience.com/collections/84ce6837-548d-4a1f-919f-0bc0d9a3952f. Xenium in situ spatial transcriptomics data is available via Zenodo (https://doi.org/10.5281/zenodo.14606776).

Research involving human participants, their data, or biological material

Policy information about studies with human participants or human data. See also policy information about sex, gender (identity/presentation), and sexual orientation and race, ethnicity and racism.

Reporting on sex and gender	Sex was used as a covariate for linear models and the sex effect was evaluated where applicable. For samples with genotype data, we confirmed the match between one's self-reported and genetically inferred sex.
Reporting on race, ethnicity, or other socially relevant groupings	Genetic ancestry was used as a covariate for linear models and the genetic ancestry effect was evaluated where applicable. Genetic ancestry was assigned based on the superpopulation described by the 1000 Genomes Project.
Population characteristics	In addition to sex and genetic ancestry, age was used as a covariate for linear models.
Recruitment	All brain specimens were obtained through informed consent via brain donation programs at the respective organizations.
Ethics oversight	All procedures and research protocols were approved by the respective ethical committees of our collaborator's institutions. Ethics committee/IRB of Mount Sinai gave ethical approval for this work. Ethics committee/IRB of James J. Peters Department of Veterans Affairs Medical Center gave ethical approval for this work. Ethics committee/IRB of Rush Alzheimer's Disease Center gave ethical approval for this work. Ethics committee/IRB of National Institute of Mental Health Human Brain Collection Core gave ethical approval for this work.

Note that full information on the approval of the study protocol must also be provided in the manuscript.

Field-specific reporting

Please select the one below that is the best fit for your research. If you are not sure, read the appropriate sections before making your selection.

☒ Life sciences ☐ Behavioural & social sciences ☐ Ecological, evolutionary & environmental sciences

For a reference copy of the document with all sections, see nature.com/documents/nr-reporting-summary-flat.pdf

Life sciences study design

All studies must disclose on these points even when the disclosure is negative.

Sample size	No sample-size calculation was performed. Our goal was to assemble the most extensive datasets possible on human PFC. Therefore, we included as many brain specimens in our study as our budget would permit.
Data exclusions	No data were excluded from the analysis.
Replication	Technical replicates were designed to improve the reproducibility of the experimental findings where applicable. For snRNA-seq data, each donor had two technical replicates. The statistical method was applied with this study design in mind to partition the inter-individual differences from within-individual technical differences. Samples were processed in batches of 6 samples each. 286 batches were processed for snRNA-seq. Nuclei from each sample were normalized and pooled with each pool run over x2 10x lanes to achieve technical replicates, for a total of 572 10x lanes. Failure in 1 of the replicates occurred in 5 (i.e. 1.7%) of the batches. In each case, failure was due to a clog occurring in one of the 10x chip lanes, a known issue with 10x microfluidics.
Randomization	All the brain specimens used in our study were acquired through brain donation programs facilitated by the involved institutions. The

Randomization	availability of these specimens was subject to chance, with no selection bias in the procurement process. Given the large number of sample attributes (diagnosis, brain bank, sex, and other metadata), we processed samples without enforcing explicit randomization, ensuring instead that these variables were broadly distributed across processing batches without systematic bias.
Blinding	The investigators were blinded to allocation of brain specimens.

Reporting for specific materials, systems and methods

We require information from authors about some types of materials, experimental systems and methods used in many studies. Here, indicate whether each material, system or method listed is relevant to your study. If you are not sure if a list item applies to your research, read the appropriate section before selecting a response.

Materials & experimental systems

n/a	Involved in the study	
☐	☒	Antibodies
☒	☐	Eukaryotic cell lines
☒	☐	Palaeontology and archaeology
☒	☐	Animals and other organisms
☒	☐	Clinical data
☒	☐	Dual use research of concern
☒	☐	Plants

Methods

n/a	Involved in the study	
☒	☐	ChIP-seq
☐	☒	Flow cytometry
☒	☐	MRI-based neuroimaging

Antibodies

Antibodies used	TotalSeq-A nuclear hashing antibodies were obtained from BioLegend. Samples were processed in batches of 6 samples and 2M nuclei from each sample were labeled with 1µl of a unique TotalSeq-A antibody, in accordance with manufacturer's instructions. No two samples within a batch were labeled with the same TotalSeq-A antibody. A total of 10 different TotalSeq-A antibodies were used across the study (BioLegend Cat no. #1 682205, #2 682207, #3 682209, #4 682211, #5 682213, #6 852215, #7 682217, #8 682219, #9 682221 & #10 682223).
Validation	The specificity of the nuclei hashing antibody has been cited in numerous publications, including PMID: 2661560, PMID: 2661560 and PMID: 11448990, while the TotalSeq product has been cited several times, including PMID: 36071158 and PMID: 38781388.

Plants

Seed stocks	n/a
Novel plant genotypes	n/a
Authentication	n/a

Flow Cytometry

Plots

Confirm that:

☒ The axis labels state the marker and fluorochrome used (e.g. CD4-FITC).

☒ The axis scales are clearly visible. Include numbers along axes only for bottom left plot of group (a 'group' is an analysis of identical markers).

☒ All plots are contour plots with outliers or pseudocolor plots.

☒ A numerical value for number of cells or percentage (with statistics) is provided.

Methodology

Sample preparation	Flow cytometry was utilized to remove debris and to generate heterogenous populations of nuclei representative of each tissue specimen. Frozen tissue was homogenized in lysis buffer and subjected to sucrose gradient ultracentrifugation . The

	resulting nuclei pellet was reuspended in PBS and stained with 7-Aminoactinomycin D (7-AAD) (Invitrogen) to facilitate isolation using a FACSAria flow cytometer (BD Biosciences).
Instrument	FACSAria Flow cytometer (BD Biosciences)
Software	FACSDiva software, version 8.0.2 (BD Biosciences)
Cell population abundance	Post FANS, sorted nuclei were quantified using a Countess II cell counter (Life Technologies).
Gating strategy	Forward and side scatter were utilized to differentiate single nuclei from debris. 7-AAD staining was utilized to isolate individual nuclei.

☒ Tick this box to confirm that a figure exemplifying the gating strategy is provided in the Supplementary Information.
