# Supplementary material for: Single-cell atlas of transcriptomic vulnerability across brain disorders
Source: Nature. 2026 Sep 23;657(8133):988–1002. doi: 10.1038/s41586-025-09573-z (PMC13600946; doi:10.1038/s41586-025-09573-z)
Supplement: Supplementary file 2 [file 41586_2025_9573_MOESM5_ESM.pdf]

nature portfolio

Peer Review File

Single-cell atlas of transcriptomic vulnerability across brain disorders

Corresponding Author: Dr Donghoon Lee

Any redactions in this file are there to maintain patient confidentiality, the confidentiality of unpublished data, or to remove third-party material.

This file contains all reviewer reports in order by version, followed by all author rebuttals in order by version.

Version 0:

Reviewer comments:

Referee #1

(Remarks to the Author)
Summary of key results. This manuscript presents a discussion of the development and analysis of a novel, extensive single-cell transcriptomic atlas from samples obtained from the human dorsolateral cortex. The work is considered “population scale” since samples were obtained from 1,494 unique donors. The significance of the work is that the study is designed to assess transcriptomic vulnerability across several common neurodegenerative and neuropsychiatric diseases: Alzheimer’s disease, Parkinson’s disease, diffuse Lewy body disease, vascular dementia, tauopathy, frontotemporal dementia, schizophrenia and bipolar disorder. These diseases are heterogeneous in clinical presentation, genetic associations and pathophysiology. Key results include specific biological signatures that are common to the diseases including mRNA splicing and protein localization. The cell-type specific analysis identified a link to excitatory neurons that was prominent when neuropsychiatric symptoms accompany Alzheimer’s disease (AD). Notably, some genes with higher inter-individual variability were involved in housekeeping roles in contrast to the fast that these genes are more likely to be conserved. Another finding of note is that the brain vascular system is linked to immune dysfunction in neurodegenerative diseases. In summary, the paper presents a detailed description of the Psych-AD single-cell disease atlas with comprehensive analysis of the data in context of the specific diseases.

Originality and significance. The data presented is unique and novel. While there are single nucleus studies of the various neurodegenerative and neuropsychiatric diseases, this study represents an effort to compile population-scale data and compare transcriptomic variation across the diseases. The significance of the work is two-fold, first to present this data resource to the research community and second to present novel findings that result from analysis of the data across the diseases. The value of the Atlas is substantial for researchers interested in analysis of single nucleus data for neurodegenerative and neuropsychological disorders.

Data and methodology. The data quality and bioinformatics methods utilized are outstanding. Several brain banks and studies were used to construct the large cohort and dataset. The study utilized rigorous approaches to harmonize the clinical, pathological and demographic metadata.

Appropriate use of statistics and treatment of uncertainties. Overall, the statistical analysis presented in this paper is appropriate and rigorous. The statistical model for differential gene expression utilizes a linear mixed model which is a strength. The sample size is “large” and several places in the manuscript note that the study is adequately powered, although the manuscript would be strengthened to better understand effect sizes since as a resource, investigators are likely to use subsets of the dataset in addition to the full dataset. The It is unclear, in the section on mediation analysis, if the statistical assumptions for mediation analysis were tested. For the differential gene expression analysis using Dreamlet, APOE genotype is not considered as a factor and this decision should either be justified or APOE effects on differential expression could be considered. It is unclear how APOE genotype was handled in the polygenic risk score analysis.

Conclusions: robustness, validity, reliability. Considering the size and composition of the data
set, the availability of other datasets for replication is very limited. Where possible, the manuscript puts results in context of prior studies and reports. Where prior study results are sparse and/or present contradictory results, this should be noted.

Suggested improvements. While the bioinformatics analysis is comprehensive and rigorous, the manuscript needs to be revised so that the results and main conclusions can be accessible and understandable by the broad scientific backgrounds of the readers of the Journal. Both Figures and text must be revised to clarify aspects of the results that may be expressed using terminology, abbreviations and notations that are specific to single nucleus analysis and therefore understandable by scientists working with this type of data. For example, Figure 5b, the labels such as EN_L3_5_IT_2 is difficult to interpret. For lines 327-328 “Additionally, the loss of EN_L2_3_IT in AD which is not evident in formal aging…..” - what is the biological interpretation of this specific neuronal subtype? In Figure 3g the label “SRP_Dependent_cotranslational_protein_targeting_to_membrane” is likewise difficult to interpret since SRP is not defined (signal recognition particle). Likewise, some of the text is difficult to understand even by bioinformaticians who may not be familiar with specific packages/methods. For example, lines 277-278 “Using Dreamlet followed by Mashr, we performed composite tests to evaluate the specificity of disease effect leading to the identification…” While referring to these specific software in Methods is fine, having this text without context in the main part of the manuscript leads to problems in interpretation for scientists who don’t understand what Dreamlet or Mashr do. In areas where specific genes are identified with biological interpretation, the genes should be identified by name, for example, there is extensive discussion of ARL17B (lines 214-220) yet this gene is never named. On line 205, Chandelier cells are noted, please explain that these are a subset of GABAergic interneurons. In some parts of the manuscript, some stated results need to be put in clearer context with prior studies. For example, lines 301-302 – “We observed a positive correlation between genetic and transcriptomic similarities…”. While this result is reported with a specific correlation coefficient, it would be helpful to place the result in context of prior studies that examined genetic and transcriptomic similarities and differences. The section on “non-linear dynamics of the AD pathological trajectory is interesting but could be placed in a clearer context with the overall design of the study to examine the transcriptome across diseases.

References. Appropriate credit is given for previous work.

Clarity and context. As noted in “suggested improvements” the manuscript requires substantial revision so that the results and main conclusions can be understandable by readers with broad scientific backgrounds. No study limitations are noted and this is a weakness. One important limitation is that all of this analysis and the Atlas itself are for one region of the brain, the dorsolateral frontal cortex. While this fact does not negate the value of the Atlas or the paper, it is a limitation and important caveat for interpretation of the data in context of the diseases considered.

(Remarks on code availability)
The code provides sufficient detail to reproduce the major results and is referenced to specific figures.

Referee #2

(Remarks to the Author)
General remarks

In this study, Lee et al. curate and analyse single-nuclei transcriptomic profiles of prefrontal cortex from > 1000 individuals affected by several complex brain disorders. They annotate cell types and perform comparative analyses within and between disorders, to identify genes and cell types involved in pathological processes, with a specific focus on Alzheimer's disease.

Processing and curating such a rich dataset is a substantial effort, and it's likely that many practitioners in single-cell genomics and neuroscience will find this dataset invaluable for further analyses. The integration of evidence from tissue molecular features, human genetics, and clinical data is both timely and relevant, representing a natural and necessary progression for single-cell tissue profiling studies. However, the reporting of data processing and analyses lacks the thoroughness needed to fully understand the dataset. As it stands, this limitation reduces the dataset's potential as a widely accessible resource.

While I am not an expert in neuropathology and therefore cannot fully assess the novelty of the associations between cell subtypes, genes, and disease, I believe that the study would benefit from a more in-depth investigation of a few prioritized cell types and genes, beyond gene ontology enrichment analyses. Although I recognize that collecting additional experimental data may be unfeasible, there is a wealth of publicly available complementary clinical and genomics data on Alzheimer's Disease (AD). At the very least, the results should be discussed in the context of the extensive literature on single-cell genomics and human genetics of AD to enhance their interpretative value.

Major comments

(1) The curated dataset presented in this study has significant potential to be highly impactful. However, the presentation of dataset features and patient metadata feels somewhat synthetic; many details are either missing or difficult to parse, and the methods section for snRNA-seq preprocessing lacks sufficient detail. Here are some specific suggestions:
- In Figure 1a: The circular visualization may not be the most effective choice. The section of the circle dedicated to each cohort is not proportional to the cohort size, which can be confusing. I suggest using a multi-panel linear barplot instead. One panel could display barplots showing percentages (including the number of donors for each category), while another could show the age distribution. This would also make it easier to label the y-axis of the female/male plot, which is currently missing. Additionally, the annotation "SNP" is unclear; consider labeling the y-axis as "% available genotype data."
- The outer bar of figure 1a denotes the number of diagnoses, but providing clear numbers for how many individuals with each diagnosis are present in the cohort would be very helpful. This information is partially shown in Figure 1c but only for a subset of the cohort. What is the rationale for excluding certain samples?
- Figure 1e: The figure could be more informative with better legend descriptions. What distances were used to construct the dendrogram? What is the relationship between the Venn diagrams and the dendrogram, aside from the labels? Consider splitting this into two panels with adequate descriptions in the legend. To facilitate comparison, replacing the Venn diagram with a colored UpSet plot (as in Figure 1c), with bars colored by sex, could be more effective.
- Including more plots from the quality control and preprocessing analyses described in the methods would be beneficial. For example, line 611 mentions that some donors were excluded for low counts, but Supplementary Figure 1h-i only shows the number of cells per sample/donor in aggregate. Could this be broken down by donor, showing which were excluded and how they compare to the rest of the cohort?
- Line 608: "We checked for possible contamination of ambient RNA" - how was this check performed?
- Can the description of the batch correction steps be expanded? Which covariates were used for batch correction? Adding a supplementary figure showing that no donor/cohort-specific cell subtypes remain after integration would be valuable.
- The methods section only describes clustering but does not explain the procedure used for cell type annotation. Was this done through manual inspection of marker genes? Please specify the set of markers used, provide references, and refer to relevant figures (e.g., Figure 2d). Including key marker genes used for annotation in Supplementary Table 2 would greatly enhance the utility of this resource.
- Unfortunately, the methodological details mentioned above are difficult to ascertain from inspecting the code repository. The steps also do not match between the methods section and the code. For instance, the preprocessing code includes a linear regression step (see https://github.com/DiseaseNeuroGenomics/PsychADxD/blob/a8143ffa1ad967619732aa6cf342df4b10786c7d/2_taxonomy/PsychAD_pegasus_end2end_2_pass.py#L64) that is not described in the methods. Furthermore, it appears that batch correction with Harmony is performed before, not after, doublet detection. Please ensure that the methods section accurately reflects the analysis steps outlined in the code.

(2) The variance analysis in figure 3 is potentially very useful to understand the features of the dataset. However, the observation that variable genes are mostly housekeeping genes makes me slightly concerned that the model is biased towards ubiquitously highly expressed genes. Can the authors show the relationship between model coefficients and the mean expression across datapoints (MA plot)? Could the authors provide an MA plot to illustrate the relationship between model coefficients and mean gene expression across datapoints? It may be beneficial to recalibrate the analysis to account for expected gene variability across tissues and populations (see related work: https://www.biorxiv.org/content/10.1101/2024.04.10.588830v1). Another concern is that variability is driven by compositional differences (described in figure 4). Additionally, having asserted that the main driver of gene expression are cell type differences, would this analysis be more informative if repeated for each cell type? See Ramirez Flores et al. (https://elifesciences.org/articles/93161) for a related approach.

(3) The compositional analysis in Figure 4 raises some questions. While Figure 4a suggests significant changes in cell type composition between patients with different disorders, Supplementary Figure 4b shows that only a small fraction of variance is explained by these disorders. Are the correlations in Figure 4a adjusted for compositional bias, or could they be showing artifactual anti-correlations? It might be clearer to either show correlation estimates using the composition estimated by Crumblr or remove the correlations to avoid confusion. Additionally, it would be helpful to include the raw data for some of the cell types highlighted in the compositional analysis in the supplementary materials e.g. boxplots showing the fraction of VLMC cells across samples from donors with NDDs and NPDs could provide insight into the magnitude of these changes. Finally, are these compositional changes expected based on what is known about these disorders? Have they been reported before, and if so, what mechanisms have been proposed?

(4) The robustness of the mediation analysis (figure 6) and its conclusions are challenging to assess. How sensitive is the model to changes in the included cell types? Would the conclusions hold if polygenic risk scores (PRS) for a different disease were used? Could the authors also clarify how this model aligns with other analyses in the paper? The mediation analysis indicates that polygenic risk influences plaque and tau progression, which then impacts microglia abundance. However, the scDRS analysis in Figure 2f shows that microglial cells over-express genes linked to AD-implicated variants. Doesn't this suggest that polygenic risk might be mediated by microglial cells?

(5) Regarding the section on nonlinear dynamics of AD pathology, it's unclear what the proposed VAE-based approach brings to the table. The motivation to employ this modelling strategy is abstractly described in the method section, but there is no data showing that this approach identifies different genes than what you would get from linear regression or common trajectory inference methods. Additionally, it's not entirely clear why the normalized ordinal labels from Braak staging couldn't be used to identify clusters of genes with similar dynamic patterns. If the primary concern is de-correlating tau and dementia, a multi-factorial linear model with an interaction term might be a more straightforward and less error-prone approach. Without calibration and sanity check analyses, it's difficult to assess whether the identified patterns are genuine or the result of over-fitting and over-smoothing.

(6) In figure 5f the authors report that disease pairs with similar differential expression effects across cell types also have a higher shared heritability. To ensure that this association is robust, the authors should adjust the correlation analysis in figure 5f to propagate uncertainties from the shared heritability and transcriptome similarity (which seem rather large from fig. 5d-e). Having validated this association, is this expected? Is this reported or investigated in similar studies comparing genomics and transcriptomics data (e.g. PsychENCODE https://www.nature.com/articles/nn.4156)?

(7) Throughout the text, the authors reference the omnigenic model of inheritance to interpret their findings, but this connection feels somewhat speculative without further supporting analysis. The authors suggest that DE genes shared between diseases may capture common effects on peripheral genes (i.e., those closer to disease-relevant genetic variants) and propose that "discounting cross-disease effects could facilitate the identification of core disease-relevant functional genes." While plausible, this hypothesis isn't further tested in the current study. A counter-argument could be that any gene found to be significantly differentially expressed when the disease is already manifest should be considered several steps downstream of the causal genetic variants. To test whether disease-specific genes can be considered "core" rather than broadly upregulated genes, I recommend using data from burden tests (e.g., https://app.genebass.org/), where the signal for rare coding variants affecting the same genes is aggregated. Genes with a high burden of rare variants tend to be more enriched in functional annotations and are often considered better proxies for "core" genes (see https://www.sciencedirect.com/science/article/pii/S0092867417306293 and https://www.nature.com/articles/s41586-022-05684-z).

(8) In general, the breadth of the analyses performed in this study require a more detailed description to be fully grasped. This would not only facilitate the interpretation of results but also make the analyses easier to replicate in similar cohorts, ultimately enhancing the impact of this work. Here are some specific

suggestions:
- Many figure legends, particularly in the main figures, are brief and do not adequately describe the plots. It's important to clearly state what is on the x-axis and y-axis, what the colors indicate, and which data points were included or excluded.
- The design and setup of some multifactorial comparative analyses are not described in the methods section (or at least, I couldn't locate their descriptions). For example, the analyses in Figure 6a and Figure 6d lack detailed explanations. I recommend dedicating a specific subparagraph to each of these analyses in the methods section, as this would make the results easier to evaluate.
- Figure 2e: the legend for Figure 2e should specify what is being enriched—marker genes, perhaps? Additionally, how are these marker genes defined?
- Where possible, be specific when reporting quantities, or refer to figures showing the data. For example:
Line 94: "roughly equal numbers" - how many?
Line 98: "over 30% of donors" - how many?
S. Fig. 1 legend: 6M - how many cells?
Line 611: "by removing donors with very low nuclei counts" - how many donors?
Line 166: what do the authors mean by "relatively concordant"?

Minor comments

(1) The term "transcriptomic vulnerability" is unclear and not defined.
(2) Line 527: Unclear what is reference 102, I expected to find a paper describing the method for assigning genetic ancestry.
(3) Line 622: 'mitochondrial chromosomes and MT' - typo?
(4) Why are there 2 sections of the methods describing assignment of genetic ancestries from genotype data? Line 526-530 and line 711-720? Which one was used on which data?
(5) Why is suppl. Fig. 4a called CCA? What quantities are beign correlated? Cell type proportions?
(6) The VAE models for dynamics seem to be trained on a very low number of epochs, at least compared to how many epochs are generally needed for an scVI model to converge. Can you show that the models have reached convergence?
(7) Avoid using qualifiers (e.g., "rigorous" line 605, "properly" in line 928).
(8) Make figure text larger where possible (fig. 1a, c-e, suppl. Fig. 1j, figure 4a).
(9) Is the Xenium data shared? Could the authors add at least one supplementary figure on Xenium dataset QC?

AI tool statement: No section of the manuscript was uploaded to AI tools, and no AI tool was used to generate or to assist in generating the content of this report. I acknowledge the use of ChatGPT by OpenAI for assistance in editing specific sections of the peer-review report.

(Remarks on code availability)
The code repository could benefit from improvements to ensure FAIRness (Findability, Accessibility, Interoperability, and Reusability). As mentioned earlier, I wasn't able to find code for all the analyses described in the study, and in some cases, the procedures outlined in the methods section do not match the code. Here are some specific suggestions for improvement:

(1) At a minimum, please ensure that each code subfolder includes a README file that indicates the purpose of each script, as done in https://github.com/DiseaseNeuroGenomics/PsychADxD/tree/main/7_trajectory
(2) Several scripts refer to a custom module pge loaded from a local library (/path/to/leetools/pegasus/). Can this module be added to the repo for reproducibility?
(3) Please ensure that all analyses presented in the manuscript have a corresponding script or subfolder in the repository. For instance, I couldn't locate the scDRS analysis or the analyses shown in Figures 5d-g.
(4) While I was able to download and inspect the snRNA-seq datasets via cellxgene and access other datasets (although not all, e.g. Xenium data), expanding the "Dataset" section of the repository README would be beneficial. This section could include pointers to various types of datasets spread across platforms (e.g., raw snRNA-seq data, processed h5ad files, genotype data, patient metadata, DE analysis results, Xenium data). This would likely reduce the number of inquiries the maintainers receive asking where to find specific pieces of data.

Referee #3

(Remarks to the Author)
Lee et al. have produced an impressively large snRNA-seq dataset from the human prefrontal cortex and analyzed it to identify patterns of differential expression across disease states. Overall, the enormous size of the data resource makes this an important resource, and researchers studying any of the diseases included in the atlas are likely to find valuable information embedded in the supplementary tables. However, the biological insights described in the main text do not provide substantial novel insights for two reasons. The cross-disorder analyses are rather superficial and may be confounded by a wide range of technical and biological factors. The AD-specific analyses are somewhat more detailed. The descriptions of disease trajectories and the dynamics of differential gene expression (Fig. 7) are quite interesting. However, the findings largely recapitulate those of previous snRNA-seq studies of AD and ADRDs without providing any new mechanistic understanding beyond what has already been shown. In addition, some details are missing that aid in the evaluation of the resource's quality and in the interpretation of the results.

1. Fig. S1. This is a massive dataset. The strategy for multiplexing and demultiplexing samples, including replicate samples for each donor, is well constructed. Overall, the quality metrics look good. It would be helpful to see the QC metrics separated out by batch and disease state, here or in a supplementary table.

2. Cell type annotations (starting at line 136): The authors note that they used the Ma 2022 (citation 6) and BICCN 2021 (citation 7) papers, but it is difficult to see how these were used from the description of the methods and the code on github. Many of the subtypes differ in nomenclature from those papers. If the authors are establishing a new cell-type nomenclature, they should explain their reasoning for diverging from the nomenclature in Ma 2022, which also studies the PFC.

3. Given the cell type proportion differences observed across ages and diagnoses (and to a proportional extent brain banks), it is important to identify in the course of iterative clustering what is driving each cluster split. How do the authors ensure that subclustering truly identifies subtypes vs. altered cell states due to age, diagnosis, collection site, etc.? This seems well-defined at he sub-class level, but less so at the finer subtype resolution. A subset of this information is provided in the plots for the distributions of cell types across brain banks and in the spatial transcriptomics data. However, this information is not presented in a sufficiently comprehensive fashion to thoroughly evaluate the quality of the sub-clustering. Were spatial transcriptomics data informative at the subtype level for INs and ENs? A further possibility would be to add metrics such as LISI to systematically evaluate the fine-level sub-types across variables.

4. Figure 3. Applying variancePartition analysis across cell types does not seem to have produced any useful results. Not surprisingly, most of the variation is across cell types. However, variancePartition does not seem to have pulled out interesting cell type markers. Rather, the top genes merely describe differences between major cell classes that are very well known. variancePartition results for other variables seem essentially uninterpretable. The most interesting genes are those that show highly cell type-specific expression patterns, as well as associations with disease, age, or other variables. But the variancePartition analysis as constructed is not designed to pull out that kind of effect. It is possible that a variancePartition analysis performed separately in each cell type could produce more informative and interesting results.

5. A number of analyses (esp. in Figs 4 and 6) compare cell type proportions between disease states, however from Supp. Fig. 2 and Supp Fig. 4 it appears that cell type proportions are different between brain banks. Cell type composition is highly sensitive to subtle variation tissue preservation, dissection, processing of nuclei, etc. In addition, cell type composition is known to change throughout aging, which is also variable across the tissue sources and disease states. These technical challenges make analyses of cell type composition very sensitive and possibly challenging to interpret. Notably, cell type composition has been extensively studied in both neuropsychiatric and neurodegenerative disorders by classical immunostaining approaches. It is concerning that some of the patterns identified in the current analysis do not line up with those previous findings. For instance, Pvalb-expression interneurons have reproducibly been shown to be reduced in SCZ, but at the subtype level they appear to be increased in Supp. Fig 4d. It would be important to clearly show how confounding variables contribute to these analyses. Even if these variables are included as covariate there may be artifacts. The authors should research and cite the previous literature and acknowledge both similarities and discrepancies with other techniques. There is also a need for replication in independent cohorts, especially ones where the cases and controls are better matched by

age, source, and other confounding variables.

6. Line 274. The analysis of differential expression with Dreamlet seems well done. However, I found it very difficult to interpret the shared and disease-specific signatures from Mashr (in the first half of Fig 5 and Supp Fig 5) because the authors do not provide information about the individual genes that contribute to these patterns. Is it possible to provide information about the gene loading in each of the signatures and to show expression patterns for the individual genes that comprise them? It would be easy for these patterns (especially shared signatures) to arise from technical issues as well as disease processes.

7. Line 321 / Fig. 6a. Cell type composition has been extensively studied in AD across stages of disease. The authors' findings seem reasonably consistent with some of this prior knowledge (e.g., dysfunction of vascular smooth muscle cells). However, it is not clear what this analysis adds beyond what has been extensively shown by others using techniques that are probably more reliable for understanding cell type proportions. At the very least, the authors should cite the prior literature. For findings that may be more novel such as a potential vulnerability of EN subclasses specifically in AD patients experiencing weight loss it would be important to validate this finding by some independent techniques, or at least in independent cohorts with transcriptomic data.

8. Mediation models are interesting, but as noted by the authors this analysis largely overlaps previous analyses. To what extent do the mediation analyses here corroborate or disagree with previous findings? My impression, unfortunately, is that previous attempts were more nuanced and provided greater insight into the disease processes. How do the mediation pathways described here compare to alternate pathways to a AD-related and aging-related states as modeled by Green et al. 2024 in Nature?

9. Line 360. Descriptions of cell type-specific DEGs in AD are extremely superficial and add little to what is already known. For instance, the transcriptional states of microglia in AD have been extensively studied, and it is known that distinct microglial states contribute to AD in different ways at different stages of the disease. For instance, lysosomal/DAM/TREM2 microglia appear to be enriched at early stages and may be protective, whereas pro-inflammatory subtypes with neurotoxic effects prevail later. Yet the authors merely state that there were many DEGs in microglia, give the names of a few that overlap previous studies, and do not at all grapple with the known complexities (line 367(. Similarly, a statement that the DEGs in neurons were enriched for synaptic functions is too vague to be informative (line 370).

10. There are no replication analyses to determine whether the patterns described in this cohort are reproducible in other snRNA-seq datasets, despite the fact that large cohorts are available for this purpose.

11. There are no validation experiments using orthogonal techniques to verify predictions. As noted above, this may be especially important for cell type proportions, which can be estimated from standard techniques and are not always reliably detected from single-cell data.

12. The Discussion is very minimal. A missed opportunity to more fully elucidate some of the connections of this work to previous studies.

13. Overall, the manuscript is surprisingly superficial and lacks deep insights into the pathology of the diseases (even for AD). Given the breadth of the datasets, this represents a missed opportunity. The manuscript as presented represents primarily a high-level analysis that basically notes similarities among neurodegenerative and neuropsychiatric, but the richness of the dataset should allow the authors to elucidate these relationships in much more nuanced ways. For instance, they could reconstruct cell-type specific gene regulatory programs that are disease specific, and provide insight into targeted therapies that can be employed in specific disease contexts – ideally in an age/ or disease stage specific manner. There are many connections to the existing/previous literature that are not drawn out. A deeper evaluation of the prior knowledge could aid in producing a more unique analysis.

Additional questions and comments:

14. What are the error bars in Fig. 5e?

15. Line 812. It appears that the meta-analysis across cohorts was performed using the absolute value of the fold change. How does the model account for the possibility that a gene may be differentially expressed in opposite directions across the two samples?

16. Line 870. I think this should be abs(logFC) > 0.5 & FDR < 0.05

17. Based on the low R value presented in Sup Fig. 3h, the correlation between genetic constraint and variance due to donor, while significant does not appear to fully explain gene expression variability between donors. A more informative analysis would be identifying cell-type specific genes that vary, and further correspond with diagnosis.

18. The authors have convincingly shown that the H1 and H2 MAPT haplotypes impacts expression of ARL17B (Fig 3e). Given the predictive power of this locus and potential importance in PD progression, could this analysis be expanded to identify other cell-type specific gene expression changes?

19. Please add a column in sup table 1 to indicate which individuals were used for each subset in fig 1 (1160, 696, 234). After reviewing the criteria for selection detailed in the methods and sorting Sup Table 1 by these criteria, I could not recreate the groups described in Fig 1.

20. Ages – sup table 1 goes up to 89+, fig 1a goes to 99+, and text mentions a 108 year old. Please be consistent. Presenting actual ages in sup table 1 would be preferable unless there are privacy concerns.

21. For sup table 1, please add "Cross-disorder groups" depicted in Fig1c

22. The authors' statement that IN subclasses being randomly distributed (line 159) is counter to existing literature – for example PVALB neuron subtypes have layer-specific enrichments (PMC10660579)

(Remarks on code availability)
The GitHub archive includes a vast number of scripts used in the analysis. I did not have time to go through all of the scripts, but the ones I looked at appeared well-written and sufficiently documented to make the analysis reproducible.

Version 1:

Reviewer comments:

Referee #1

(Remarks to the Author)
The revision is extremely comprehensive and addressed the concerns that I described in detail in the original review.

(Remarks on code availability)
The code is comprehensive and well documented. There is a README file that provides general instructions for installing the code.

Referee #2

(Remarks to the Author)
The authors have substantially expanded the manuscript, providing improved rationale and analytical details that make the datasets and findings more accessible. While many of the main results recapitulate previous findings, this work will serve as a valuable resource for the community studying neurological and psychiatric disease. I have a few minor presentation issues should be addressed to improve clarity and interpretation:

1) The term "transcriptomic vulnerability" still requires clearer definition. I think the statement in the reviewer comments ("Shared transcriptomic vulnerability refers to the common patterns of gene expression perturbations observed across different brain diseases.") is a clearer description than any of the text in the manuscript.
2) The relationship between variance explained and baseline expression level is explored in Supplementary Figure 5h, but not referenced in the main text. This technical effect should be acknowledged in the main text, as it substantially affects interpretation of Figure 3b. The current framing that the analysis "prioritized several drivers of expression variation" is misleading since the approach is inherently biased toward highly expressed genes.
3) While interesting, the association between expression patterns and loss-of-function burden shows overall weak correlation significant in only two cell types (Supplementary Figure 8). This limitation should be acknowledged in the main text alongside the presentation of these results.
4) The expanded section on "cross-disorder variation in gene expression and genetic concordance" would benefit from additional discussion of implications. Key questions merit consideration: What insights does concordance provide? Should future studies focus on shared or distinct DE signatures? Why are cell types with higher concordance more relevant? These findings warrant an additional section in the discussion, in relation to previous neuro-genetics studies.
5) Regarding REF2-7: the comparison with PLS regression presented in suppl. Fig. 15 is not a fair comparison, since the linear model is not jointly trained on Dementia and Braak staging like the NN model is. It is to be expected that the two factors remain correlated. This comparison makes sense against a multi-factorial model but not a univariate model. At the very least, I would add to the text (in Methods) the arguments presented in the reviewer response under section "Possible use of a multi-factorial linear model"

(Remarks on code availability)
While the code repository is substantially improved, I could still catch instances where I wasn't able to locate the code used for a specific analysis (e.g. comparison between NN and PLS model for disease pseudotime described above). I recommend that the authors to make sure the code repository is complete, ideally also including the code used to generate raw figures for all main and supplementary figures in the paper. Complete code availability, including scripts for generating all figures, would better serve the research community and reduce post-publication clarification requests.

Referee #3

(Remarks to the Author)
The authors have thoroughly addressed my concerns. The revised manuscript is very impressive, and the study overall represents a major accomplishment and an important resource for the field. Congratulations!

(Remarks on code availability)
The code repository Is comprehensive. During their revisions, the authors have made the code repository significantly more accessible and usable.

Open Access This Peer Review File is licensed under a Creative Commons Attribution 4.0 International License, which permits use, sharing, adaptation, distribution and reproduction in any medium or format, as long as you give appropriate credit to the original author(s) and the source, provide a link to the Creative Commons license, and indicate if changes were made.
In cases where reviewers are anonymous, credit should be given to 'Anonymous Referee' and the source.
The images or other third party material in this Peer Review File are included in the article's Creative Commons license, unless indicated otherwise in a credit line to the material. If material is not included in the article's Creative Commons license and your intended use is not permitted by statutory regulation or exceeds the permitted use, you will need to obtain permission directly from the copyright holder.
To view a copy of this license, visit https://creativecommons.org/licenses/by/4.0/

Editor

<table>
<tr><td colspan="2">REF0-1. Decision on Nature manuscript 2024-07-15352</td></tr>
<tr><td colspan="2">Your manuscript, "Single-cell atlas of transcriptomic vulnerability across multiple neurodegenerative and neuropsychiatric diseases", has now been seen by 3 referees, whose comments are attached below. While they find your work of potential interest, as do we, they have raised important concerns that in our view need to be addressed before we can consider publication in Nature.

Should further experiments allow you to address these criticisms, we would be happy to consider a revised manuscript (unless something similar has been accepted at Nature or appeared elsewhere in the meantime). We hope to receive your revised paper within four to six months. If you cannot complete the required revisions within this time frame, please let us know when you would anticipate being able to submit a revised manuscript. We also strongly suggest that your revised manuscript has tracked changes, which is increasingly requested by referees to aid in their re-review.

We are committed to providing a fair and constructive peer-review process. Please do not hesitate to contact us if there are specific requests from the reviewers that you believe are technically impossible or unlikely to yield a meaningful outcome.</td></tr>
<tr><td>Response</td><td>We appreciate the reviewers' recognition of their interest in our work. We understand and respect the concerns raised regarding the conceptual advance over earlier studies and the strength of novel conclusions.

We recognize the rigorous standards of Nature, and we are grateful for the opportunity to have our work evaluated. We will carefully consider the reviewers' comments and suggestions to address the concerns raised.</td></tr>
</table>

Referee #1 (Remarks to the Author):

REF1-1. Summary of key results	
Summary of key results. This manuscript presents a discussion of the development and analysis of a novel, extensive single-cell transcriptomic atlas from samples obtained from the human dorsolateral cortex. The work is considered "population scale" since samples were obtained from 1,494 unique donors. The significance of the work is that the study is designed to assess transcriptomic vulnerability across several common neurodegenerative and neuropsychiatric diseases: Alzheimer's disease, Parkinson's disease, diffuse Lewy body disease, vascular dementia, tauopathy, frontotemporal dementia, schizophrenia and bipolar disorder. These diseases are heterogeneous in clinical presentation, genetic associations and pathophysiology. Key results include specific biological signatures that are common to the diseases including mRNA splicing and protein localization. The cell-type specific analysis identified a link to excitatory neurons that was prominent when neuropsychiatric symptoms accompany Alzheimer's disease (AD). Notably, some genes with higher inter-individual variability were involved in housekeeping roles in contrast to the fast that these genes are more likely to be conserved. Another finding of note is that the brain vascular system is linked to immune dysfunction in neurodegenerative diseases. In summary, the paper presents a detailed description of the Psych-AD single-cell disease atlas with comprehensive analysis of the data in context of the specific diseases.	
Response	We appreciate the reviewer for the thorough and insightful assessment of our manuscript.

REF1-2. Originality and significance	
Originality and significance. The data presented is unique and novel. While there are single nucleus studies of the various neurodegenerative and neuropsychiatric diseases, this study represents an effort to compile population-scale data and compare transcriptomic variation across the diseases. The significance of the work is two-fold, first to present this data resource to the research community and second to present novel findings that result from analysis of the data across the diseases. The value of the Atlas is substantial for researchers interested in analysis of single nucleus data for neurodegenerative and neuropsychological disorders.	
Response	We appreciate your positive feedback and recognition of the originality and significance of our work. We agree this atlas is a valuable resource for researchers interested in single-cell resolution data for neurodegenerative and neuropsychological disorders.<br><br>We also want to highlight that this study is part of a larger package of papers (see https://psych-ad.org). Among these are studies that delve deeper into specific subject areas such as healthy aging, QTL, TWAS, and disease phenotyping. These complementary studies provide further insights into the complex landscape of human brain disorders.<br><br>We believe that the combined impact of these studies will be substantial, offering a comprehensive understanding of a range of brain disorders and paving the way for new therapeutic strategies.

REF1-3. Data and methodology	
Data and methodology. The data quality and bioinformatics methods utilized are outstanding. Several brain banks and studies were used to construct the large cohort and dataset. The study utilized rigorous approaches to harmonize the clinical, pathological and demographic metadata.	
Response	We appreciate your recognition of the data quality and bioinformatics methods utilized.

REF1-4. Appropriate use of statistics and treatment of uncertainties	
Appropriate use of statistics and treatment of uncertainties. Overall, the statistical analysis presented in this paper is appropriate and rigorous. The statistical model for differential gene expression utilizes a linear mixed model which is a strength. The sample size is “large” and several places in the manuscript note that the study is adequately powered, although the manuscript would be strengthened to better understand effect sizes since as a resource, investigators are likely to use subsets of the dataset in addition to the full dataset.	
Response	We agree that providing further insights into effect sizes would enhance the manuscript's utility, especially for researchers interested in utilizing subsets of our data.<br><br>In response, we performed additional analyses to address power and effect size from a theoretical and then empirical perspective. We have expanded the **Methods** section (see section titled “Statistical power and effect size considerations in single-cell analyses”) to include a detailed discussion of power calculations for both cell type composition and differential expression analyses. Specifically, we now report that, based on theoretical calculations and our observed sample sizes and cell counts, we have >80% power to detect changes in cell type composition, as small as ~0.1%, and differential expression effect sizes of 1 on Cohen’s d scale (**Supplementary Figs. 5a-b**). We further acknowledge the variability in empirical power across cell types and genes due to biological and technical factors, including the number of cells per cluster, read depth, and the number of individuals passing QC filters. These additions also address concerns about potential effect size inflation in low-powered settings due to Winner’s Curse and the observed relationship between the number of differentially expressed genes and mean effect size estimates (**Supplementary Figs. 5c-g**). We believe these additions clarify the scope and limitations of our study and strengthen the interpretation of our findings.
Excerpt	“... To support interpretation and enable future analyses, we evaluated power and effect size variability across cell types (**Methods**, **Supplementary figs. 5a-g**). …”<br><br>“... **Statistical power and effect size considerations in single-cell analyses**<br>To complement the primary analyses presented in the manuscript, we conducted additional power and effect size assessments to guide interpretation of the results and provide context for the sensitivity of our study. These analyses include both theoretical calculations and empirical evaluations designed to quantify the ability to detect changes in cellular composition and gene expression, as well as to highlight variability in power across cell types. Based on theoretical calculations, the statistical power to identify changes in cell type composition increases with the magnitude of the difference and the total number of cells observed. With the sample size and cell count in this study, we have >80% power to identify changes in cell type composition of ~ 0.1% (**Supplementary Fig. 5a**). For differential expression analyses, power increases with sample size, standardized effect size and correlation between measured and true gene expression. Given the sample size in this study and moderated R^2 values between measured and true gene expression, we have >80% power to detect effect sizes of 1 on Cohen’s d scale (**Supplementary Fig. 5b**). In practice, there are many factors affecting statistical power in gene expression studies. In bulk RNA-seq experiments, power is determined by biological signal and sample size in addition to total read count and magnitude of gene expression. In single cell datasets, additional technical factors include the number of

	cells collected for each cell cluster, and the number of reads per cell. These numbers vary widely across cell types, as does the number of individuals with sufficient cell and read counts. As such, empirical statistical power varies across cell types and genes even when testing the case/control analysis for one disease. In addition, the Winner's Curse phenomenon means that effect size estimates that pass a 5% FDR cutoff in low-powered cell types will be substantially overestimated compared to effect sizes in well-powered cell types. In our analysis of AD versus controls, there is wide variation in the number of expressed genes and the number of individuals passing filters based on cell and read count. We observe that the number of DEGs increases with the number of individuals passing filters and the number of expressed genes (**Supplementary Fig. 5c**). Yet the mean of the absolute value of the effect size estimates decreases with numbers of individuals and expressed genes (**Supplementary Fig. 5d**). Indeed, there is a strong negative correlation (Spearman's rho = 0.58) between the number of DEGs and the mean absolute effect size (**Supplementary Fig. 5e**). Examining the estimated effect size in more detail, we see estimated effect size centered at zero for all cell subclasses (**Supplementary Fig. 5f**), but the mean of the absolute estimated effect sizes varies widely for DEGs (**Supplementary Fig. 5g**). "

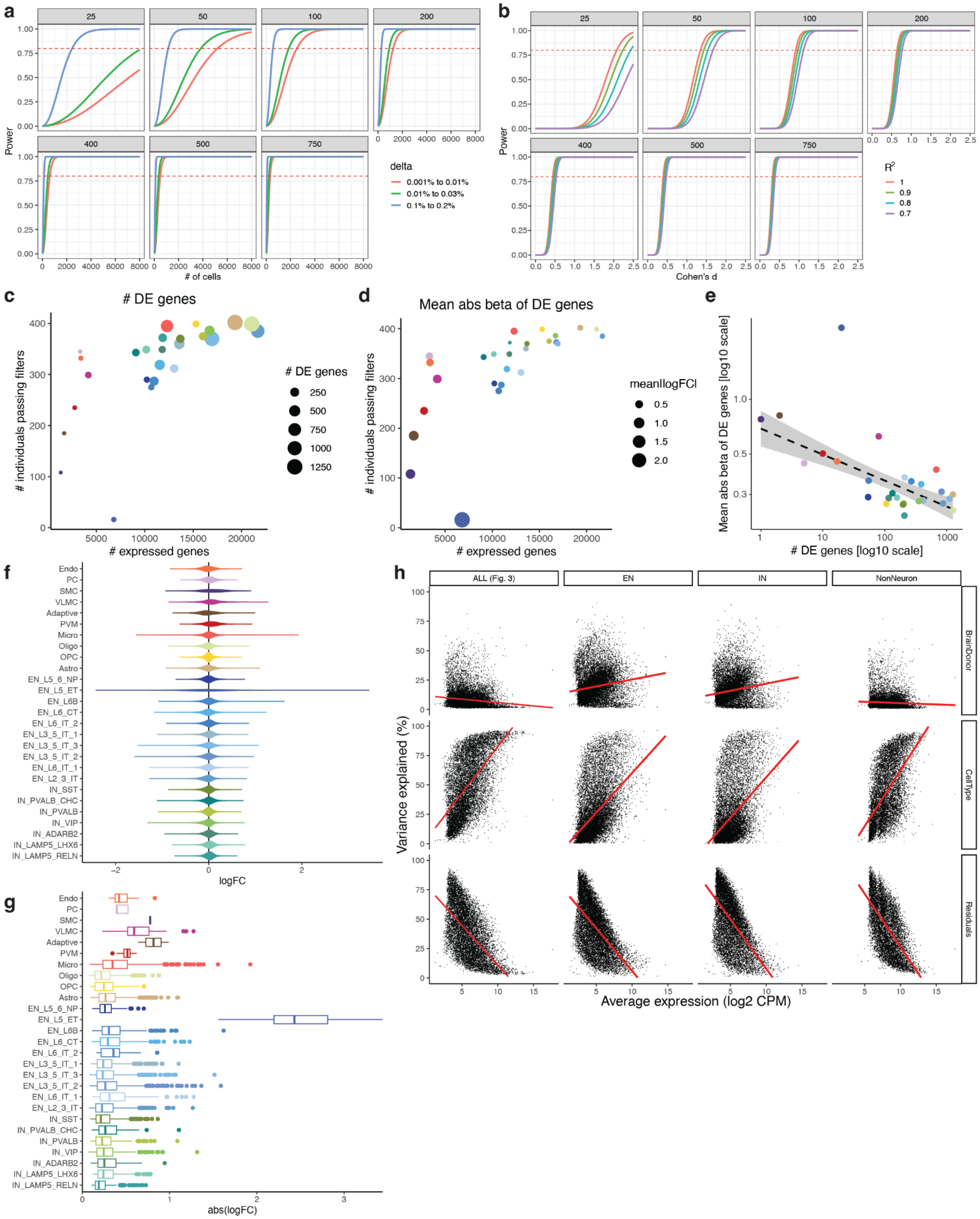

Supplementary Fig. 5. Statistical power and effect size considerations in single-cell analyses. **(a)** Power to identify changes in cellular composition. Total number of cells per individual is shown on the x-axis and change in composition (i.e. delta) is shown by colored lines. Numbers in grey panels indicate sample size in each disease class, i.e. “25” indicates 25 cases and 25 controls. **(b)** Power to identify changes in gene expression. Standardized effect size is shown in Cohen’s d. Power calculations use the approach of Mandric et al.[1] to model the noise in single cell assays using the squared correlation between measured and true gene expression. Numbers in grey panels indicate sample size in each disease class, as in panel a. **(c-d)** Variation in power across cell types for analysis of AD versus controls. Each point represents a cell type and with the number of expressed genes and individuals

	passing filters for cell and read counts. The size of the points indicates **(c)** Number of DEGs and **(d)** Mean absolute effect size of genes that pass FDR 5% cutoff. Each point is a cell subclass colored as in the main text. **(e)** Negative association between number of DEGs and mean absolute value of estimated effect size. Each point is a cell subclass colored as in the main text. **(f-g)** Estimated effect size for AD vs control comparison. **(f)** Estimated effect size for all genes. **(g)** Absolute estimated effect size for DEGs. **(h)** Relationship between the percentage of variation explained by mean expression (log2 counts per million). Rows are stratified by model coefficients; brain donors (BrainDonor) and cell types (CellTypes). Gene expression variance explained by these variables is computed for all subclasses (as shown in Fig. 3a) and three superclasses (as shown in Supplementary Fig. 6j), namely, Excitatory Neurons (EN), Inhibitory Neurons (IN), and Non-Neurons (NonNeuron). For example, variance of superclass "EN" is computed across all EN subclasses. The same is true for "IN". "NonNeuron" comprises all subclasses but EN and IN subclasses.

REF1-5. Clarification of statistical assumptions	
It is unclear, in the section on mediation analysis, if the statistical assumptions for mediation analysis were tested.	
Response	We acknowledge that the statistical assumptions for mediation analysis were not explicitly stated in the original manuscript and have revised the **Methods** section (titled "Causal mediation analysis") accordingly. We believe this strengthens the transparency and rigor of our mediation analysis.<br><br>One key assumption in causal mediation analysis is that there is no unobserved confounding effect between the mediator and the outcome. To ensure this, we carefully selected and included covariates in our model that are known, or suspected, confounders of the mediator-outcome relationship. Furthermore, we ensured that the dependent, independent, and mediator variables were continuous, normally distributed, and linearly related.<br><br>Moreover, we performed additional sensitivity analyses to strengthen the robustness of our mediation findings (see **REF1-6**). To ensure the AD-specificity of the observed mediation, we performed the causal mediation analysis using PRSs derived from other neurological diseases (PD, MS, ALS, SCZ, BD, and ASD). We found that none of these alternative PRSs yielded significant mediation effects (**Supplementary Table 13**), supporting the specificity of our findings to AD.
Excerpt	**Causal mediation analysis**<br>**Methods**. Causal mediation analysis was performed on a subset of 645 individuals with European ancestry in AD contrast (Tier 2, n=696), who have PRS calculations from the latest AD GWAS[2]. Two different R packages were utilized, namely mediation (https://cran.r-project.org/web/packages/mediation/) and psych (https://cran.r-project.org/web/packages/psych/). The results were cross-checked between the two methods (identical within a threshold) to ensure the estimated coefficients, and the mediation effects are statistically robust. **Statistical assumptions**. A key assumption in causal mediation analysis is that there are no unobserved confounding effects between the mediator and the outcome. To address this, we carefully selected and included covariates in our model that are known or suspected to confound the mediator-outcome relationship. Furthermore, we ensured that the dependent, independent, and mediator variables were continuous, normally distributed, and linearly related. For each regression, we used the following covariates:<br><br>Age + Sex + PMI + PC1 + PC2 + PC3 + PC4 + PC5 + PC6 + PC7 + PC8 + PC9 + PC10<br><br>where PC1-10 indicate genotype PCs. For the variation of subclass-level cell type composition, we used CLR-transformed cell count fractions from the crumblr compositional analysis. For bootstrapping, we used 10,000 simulations with 50th percentiles of the treatment variable used as the control condition and 90th percentile of the treatment variable used as the treatment condition, similar to previous publication[3]. **Sensitivity analysis**. We performed additional sensitivity analyses to strengthen the robustness of the mediation analysis. To ensure the AD-

	specificity of the observed mediation, we performed the causal mediation analysis using PRSs derived from other neurological diseases (PD, MS, ALS, SCZ, BD, and ASD). We found that none of these alternative PRSs yielded significant mediation effects (**Supplementary Table 13**), supporting the specificity of our findings to AD.

REF1-6. Use of APOE genotype	
For the differential gene expression analysis using Dreamlet, APOE genotype is not considered as a factor and this decision should either be justified or APOE effects on differential expression could be considered. It is unclear how APOE genotype was handled in the polygenic risk score analysis.	
Response	We thank the reviewer for their insightful comments regarding the handling of APOE genotype in our analyses. We have addressed the reviewer's points in the revised manuscript as follows:<br><br>**1. Regarding differential gene expression analysis using Dreamlet**: We considered including APOE genotype as a covariate in the linear mixed model, but this was not supported by the data. First, performing differential expression analysis based on APOE genotype did not identify any genes as differentially expressed at a study-wide FDR of 5%. Second, we then included APOE genotype as a covariate in the differential expression analysis of AD vs controls. The estimated effect sizes for AD were very similar when comparing models with and without APOE genotype (**Supplementary Fig. 13a**). No genes show a significant difference in estimated effect size between the two models. Based on these empirical results, our analysis excluded APOE genotype in the model. The results from these supplementary analyses are described in the revised **Methods**.<br><br>**2. Regarding PRS calculations**: To clarify the handling of APOE genotype in the polygenic risk score (PRS) analysis, we now have explicitly stated in the **Methods** section that the PRS were calculated by both including and excluding the APOE locus. This decision was motivated by the well-established and substantial impact of APOE on Alzheimer's disease risk[4,5]. By excluding APOE, we aimed to assess the contribution of other genetic risk variants and their potential interplay with APOE. Furthermore, we conducted a sensitivity analysis comparing the PRS generated with and without the APOE locus. We found marginal differences between the two PRS, suggesting that the exclusion of APOE did not significantly alter the overall genetic risk profile (**Supplementary Fig. 13b**).<br><br>**3. Regarding mediation analysis**: We performed additional sensitivity analysis to test the robustness of the mediation analysis. Given the APOE ε4 allele is a strong and well-established risk factor, we compared the mediation analysis results when using PRS with or without the APOE locus (**Supplementary Table 13**). We observed negligible differences when the APOE locus was removed from PRS, emphasizing the robustness of the mediation analysis.
Excerpt	"...<br>**Differential gene expression analysis using Dreamlet**<br>… **Note on APOE genotype**. We considered including the APOE genotype as a covariate in the differential expression analysis of AD, but this was not supported by the data. First, performing differential expression analysis based on the APOE genotype did not identify any genes as differentially expressed at a study-wide FDR of 5%. Second, we then included APOE genotype as a covariate in the differential expression analysis of AD vs controls. The estimated effect sizes for AD were very similar when comparing models with and without APOE genotype (**Supplementary Fig. 13a**). No genes show a significant difference in estimated effect size between the two models. Based on these empirical results, our analysis excluded APOE genotype in the model.<br>…"<br><br>"...<br>**Polygenic risk score calculation**<br>… For AD PRS, two separate scores were calculated – both including and excluding the APOE locus. We conducted a sensitivity analysis comparing the PRS generated with and without the APOE locus. We found negligible

differences between the two PRS, suggesting that the exclusion of APOE did not significantly alter the overall genetic risk profile (**Supplementary Fig. 13b**). The PRS with the APOE locus was used for the primary analysis. …"

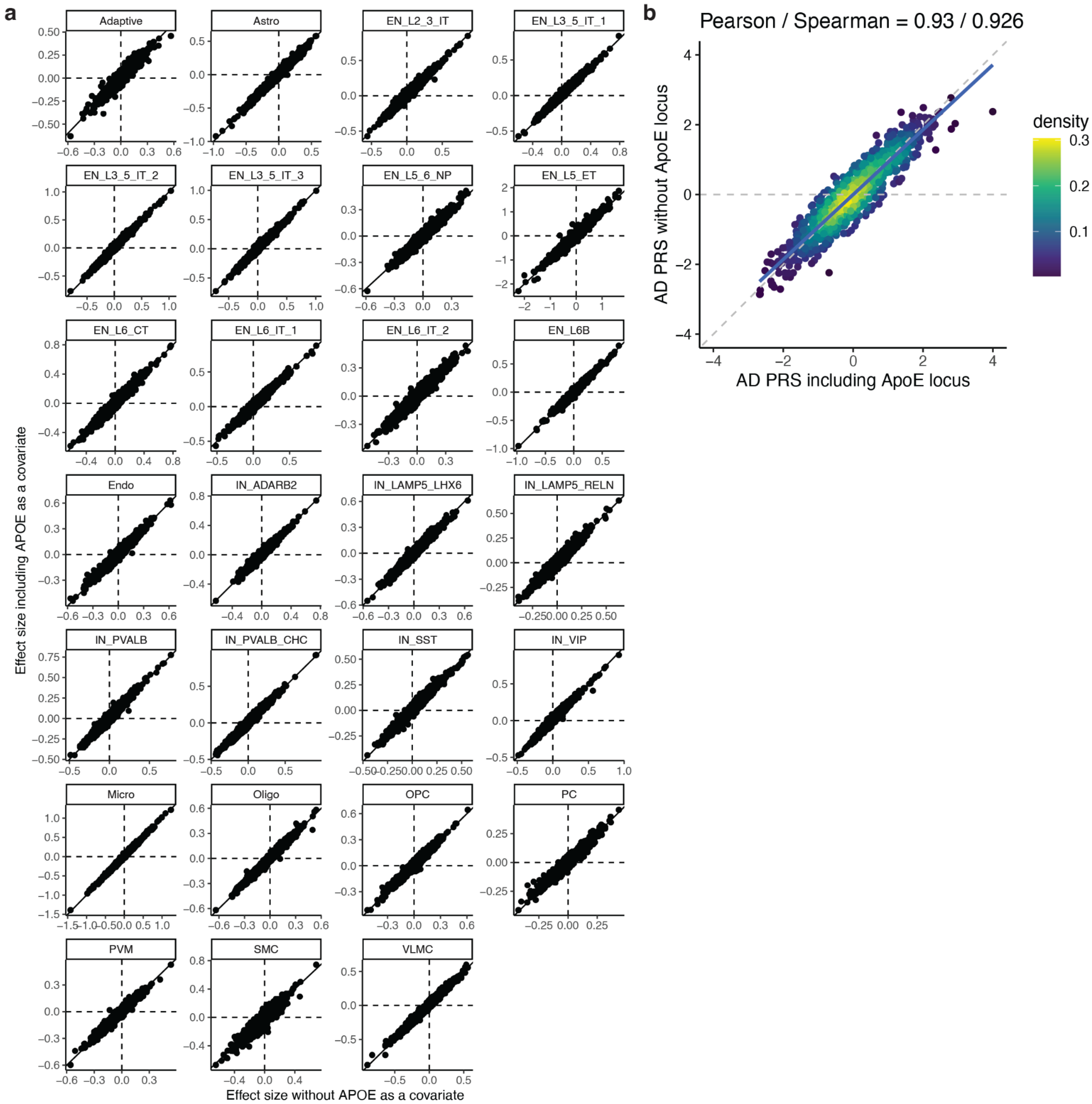

Supplementary Fig. 13. **(a)** Estimated effect sizes from differential expression analysis of AD vs control. Comparison of AD models excluding (x-axis) vs including (y-axis) APOE genotype as a covariate. Each point represents a gene. Both analyses included the following covariates: age, sex, PMI, log(n_genes), percent_mito, mito_genes, ribo_genes, and mito_ribo. **(b)** Correlation between PRS scores calculated including (x-axis) vs excluding (y-axis) APOE locus.

Treatment (IV)	Mediator (MV)	Outcome (DV)	Effects	Estimate	95% CI Lower	95% CI Upper	p-value	Significance	Note
PRS_AD	PLAQUE	Braak	ACME	0.0878	0.05	0.13	<2e-16	***	AD_2022_Bellenguez
PRS_AD	PLAQUE	Braak	ADE	0.0735	0.0296	0.12	0.0014	**	AD_2022_Bellenguez
PRS_AD	PLAQUE	Braak	Total Effect	0.1613	0.1045	0.22	<2e-16	***	AD_2022_Bellenguez
PRS_AD	PLAQUE	Braak	Prop. Mediated	0.5444	0.3633	0.76	<2e-16	***	AD_2022_Bellenguez
PRS_AD_wo_APOE	PLAQUE	Braak	ACME	0.1342	0.0866	0.18	<2e-16	***	AD_2022_Bellenguez without APOE locus
PRS_AD_wo_APOE	PLAQUE	Braak	ADE	0.0907	0.0334	0.15	0.0018	**	AD_2022_Bellenguez without APOE locus
PRS_AD_wo_APOE	PLAQUE	Braak	Total Effect	0.2249	0.1525	0.3	<2e-16	***	AD_2022_Bellenguez without APOE locus
PRS_AD_wo_APOE	PLAQUE	Braak	Prop. Mediated	0.5958	0.4276	0.81	<2e-16	***	AD_2022_Bellenguez without APOE locus
PRS_PD	PLAQUE	Braak	ACME	-0.0418	-0.0897	0.01	0.085	.	PD_2019_Nalls
PRS_PD	PLAQUE	Braak	ADE	0.014	-0.0397	0.07	0.612		PD_2019_Nalls
PRS_PD	PLAQUE	Braak	Total Effect	-0.0278	-0.099	0.04	0.45		PD_2019_Nalls
PRS_PD	PLAQUE	Braak	Prop. Mediated	0.9626	-8.6891	10.14	0.399		PD_2019_Nalls
PRS_ALS	PLAQUE	Braak	ACME	0.021	-0.0198	0.06	0.3		ALS_2021_vanRheenen
PRS_ALS	PLAQUE	Braak	ADE	0.0284	-0.0173	0.07	0.22		ALS_2021_vanRheenen
PRS_ALS	PLAQUE	Braak	Total Effect	0.0495	-0.0105	0.11	0.11		ALS_2021_vanRheenen
PRS_ALS	PLAQUE	Braak	Prop. Mediated	0.4228	-1.6198	2.22	0.28		ALS_2021_vanRheenen
PRS_MS	PLAQUE	Braak	ACME	0.00877	-0.031	0.05	0.68		MS_2019_IMSGC
PRS_MS	PLAQUE	Braak	ADE	-0.02275	-0.06859	0.02	0.33		MS_2019_IMSGC
PRS_MS	PLAQUE	Braak	Total Effect	-0.01399	-0.07371	0.05	0.65		MS_2019_IMSGC
PRS_MS	PLAQUE	Braak	Prop. Mediated	0.21086	-9.73452	8.34	0.78		MS_2019_IMSGC
PRS_SCZ	PLAQUE	Braak	ACME	-0.00548	-0.04663	0.04	0.79		SCZ_2022_Trubetskoy
PRS_SCZ	PLAQUE	Braak	ADE	0.01654	-0.03092	0.06	0.49		SCZ_2022_Trubetskoy
PRS_SCZ	PLAQUE	Braak	Total Effect	0.01106	-0.05093	0.07	0.73		SCZ_2022_Trubetskoy
PRS_SCZ	PLAQUE	Braak	Prop. Mediated	0.31545	-6.80958	7.49	0.66		SCZ_2022_Trubetskoy
PRS_BD	PLAQUE	Braak	ACME	-0.00184	-0.04155	0.04	0.92		BD_2021_Mullins
PRS_BD	PLAQUE	Braak	ADE	0.00309	-0.04126	0.05	0.88		BD_2021_Mullins
PRS_BD	PLAQUE	Braak	Total Effect	0.00124	-0.05802	0.06	0.97		BD_2021_Mullins
PRS_BD	PLAQUE	Braak	Prop. Mediated	0.44031	-5.62087	7.29	0.55		BD_2021_Mullins
PRS_ASD	PLAQUE	Braak	ACME	0.00407	-0.0387	0.05	0.84		ASD_2019_Grove
PRS_ASD	PLAQUE	Braak	ADE	0.02864	-0.0184	0.08	0.23		ASD_2019_Grove
PRS_ASD	PLAQUE	Braak	Total Effect	0.03271	-0.02994	0.1	0.31		ASD_2019_Grove
PRS_ASD	PLAQUE	Braak	Prop. Mediated	0.25062	-4.19067	5.1	0.62		ASD_2019_Grove

Supplementary Table 13. Causal mediation analysis involving PRS, cell type composition, pathology, and dementia status. Estimates of direct and mediating effects and their statistical significance.

REF1-7. Prior study results

Conclusions: robustness, validity, reliability. Considering the size and composition of the dataset, the availability of other datasets for replication is very limited. Where possible, the manuscript puts results in context of prior studies and reports. Where prior study results are sparse and/or present contradictory results, this should be noted.

Response

We appreciate the reviewer's feedback and agree that replicating our findings with existing datasets is crucial.

To address these concerns, we performed a replication of our DEG analysis using two additional AD associated single-cell datasets from the human prefrontal cortex: the ROSMAP[6] and the SEA-AD[7] studies. Overall, we successfully replicated our key findings and observed highly concordant disease signatures across these independent datasets (**Supplementary Figs. 12a-e**). We have updated the manuscript to include these replication results and discuss their implications.

In addition, we compared our compositional variation analyses to two previous studies and found high concordance across different measures of AD pathology (CERAD score, Braak stage and ordinal dementia scale) (**Supplementary Figs. 10a-b**). Furthermore, we replicated our findings using the published SEA-AD MERFISH dataset[7], showing the observed change is robust to technology used (**Supplementary Fig. 10c**). These datasets had differing demographics and tissue handling protocols, further supporting the robustness of our findings across distinct sources and methodologies.

These additional analyses strengthen the robustness of our conclusions and provide further support for the validity and reliability of our findings.

Excerpt

"... DEGs in general had high concordance across different AD pathology variables, consistent with previous reports[6], and the disease signatures were highly concordant with previous studies[6,7] (**Supplementary Figs. 12a-e**). …"

"... The variation in cell type composition reveals distinct patterns associated with AD pathologies compared to normal aging (**Fig. 6a, Supplementary Fig. 9a, Supplementary Table 5**) and were consistent with previous studies[6,7] (**Supplementary Figs. 10a-b**). Furthermore, we replicated our findings using a published MERFISH dataset[7], showing the observed change is robust to technology used (**Supplementary Fig. 10c**). …"

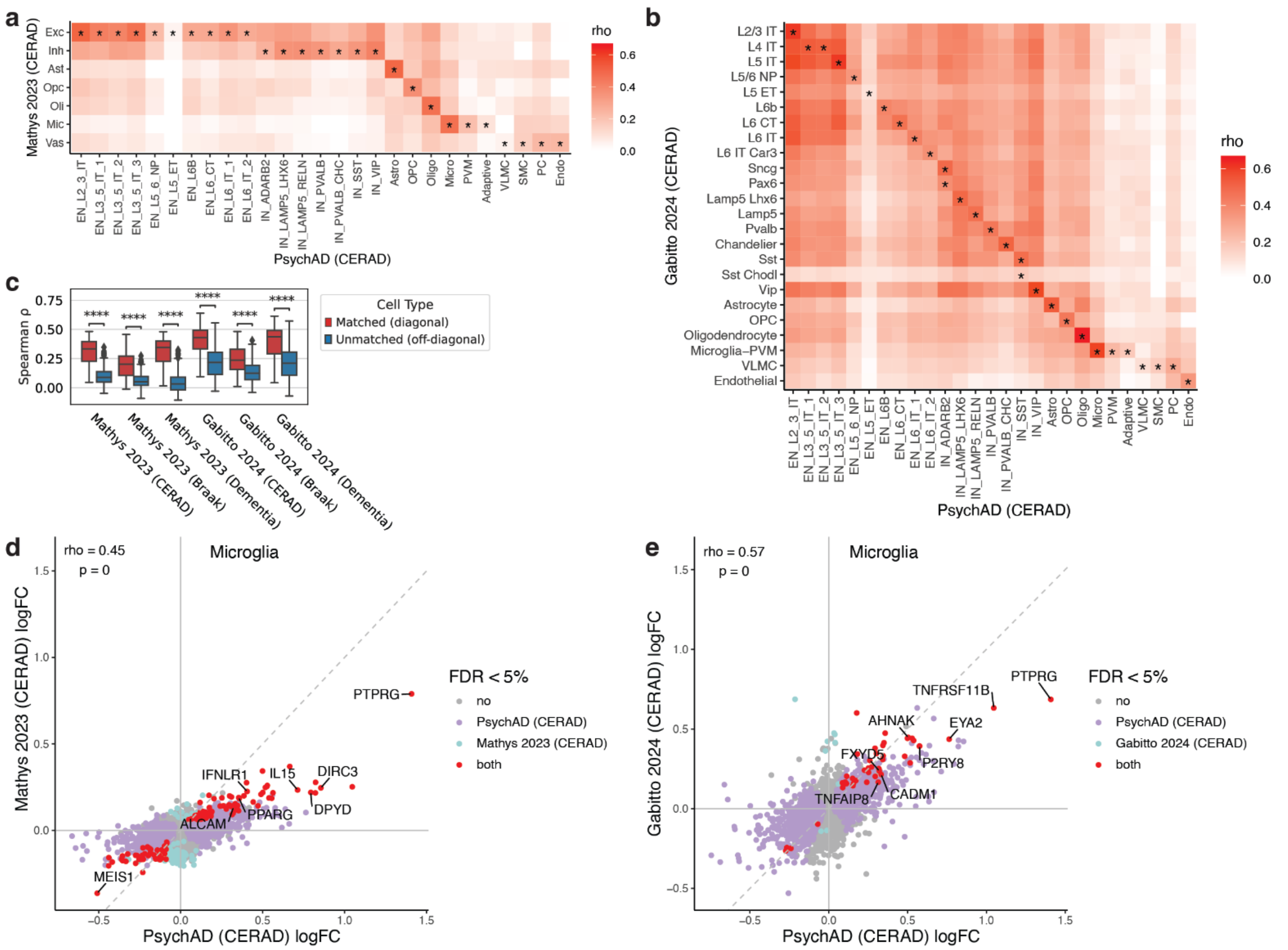

Supplementary Fig. 12. (a) Comparison of differential expression signatures with previous studies – ROSMAP (Mathys et al. 2023)[6] and SEA-AD (Gabitto et al. 2024)[7]. Pairwise Spearman correlation between PsychAD subclass and ROSMAP Major Cell Type. CERAD as a measure of disease severity. Asterisks indicate the best matching cell type based on **Supplementary Table 11**. **(b)** Pairwise Spearman correlation between PsychAD subclass and SEA-AD subclass. CERAD as a measure of disease severity. Asterisks indicate the best matching cell type based on **Supplementary Table 11**. **(c)** Comparison of various disease signatures (CERAD, Braak, Dementia) between matched (diagonal, asterisks) and unmatched (off-diagonal) cell types. Mann–Whitney U test. ****: $p \leq 1.0e\text{-}4$. **(d)** Comparison of differentially expressed genes between PsychAD Microglia and ROSMAP Mic using CERAD as a measure of disease severity. Spearman correlation (rho) shown. **(e)** Comparison of differentially expressed genes between PsychAD Microglia and SEA-AD Microglia-PVM using CERAD as a measure of disease severity. Spearman correlation (rho) shown.

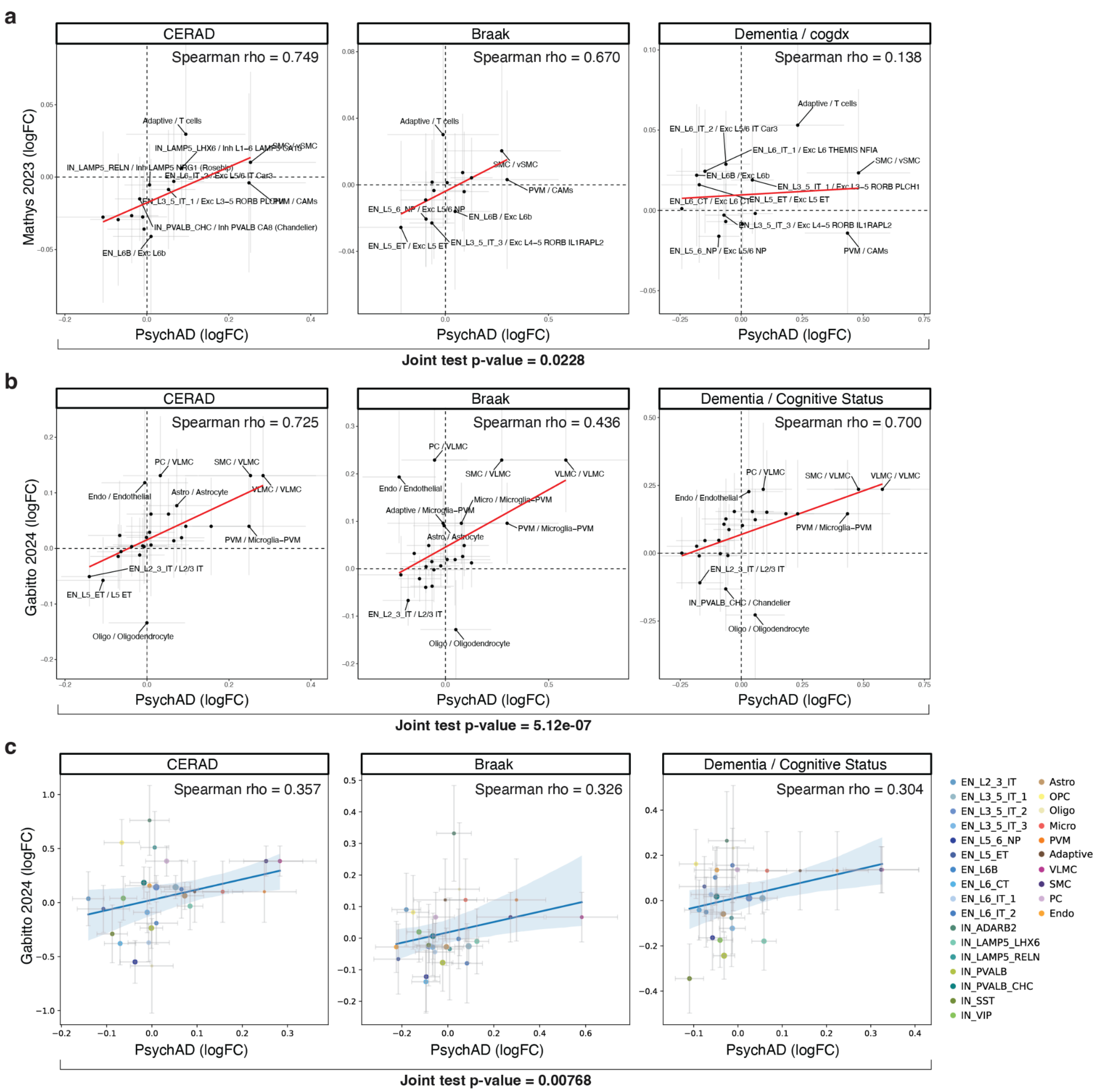

Supplementary Fig. 10. Comparison of compositional variation analysis with previous studies - DLPFC snRNA-seq data from **(a)** ROSMAP (Mathys et al. 2023)[6] and **(b)** SEA-AD (Gabitto et al. 2024)[7]. **(c)** Comparison of compositional variation analysis using the MERFISH spatial transcriptomics dataset included in the SEA-AD study[7]. Spearman correlation between this study (x-axis) and previous studies (y-axis) using 3 different measures of AD pathology (CERAD score, Braak staging, and ordinal Dementia scale). ROSMAP used clinical diagnoses of AD, MCI, and NCI, while SEA-AD used clinical diagnosis of dementia as a measure of cognitive decline. Further details regarding compositional analysis of the MERFISH data and joint statistical testing can be found in the **Methods** section.

REF1-8. Suggested improvements - abbreviation & terminology

Suggested improvements. While the bioinformatics analysis is comprehensive and rigorous, the manuscript needs to be revised so that the results and main conclusions can be accessible and understandable by the broad scientific backgrounds of the readers of the Journal. Both Figures and text must be revised to clarify aspects of the results that may be expressed using terminology,

<table>
<tr><td colspan="2">abbreviations and notations that are specific to single nucleus analysis and therefore understandable by scientists working with this type of data. For example, Figure 5b, the labels such as EN_L3_5_IT_2 are difficult to interpret. For lines 327-328 “Additionally, the loss of EN_L2_3_IT in AD which is not evident in formal aging” - what is the biological interpretation of this specific neuronal subtype? In Figure 3g the label “SRP_Dependent_cotranslational_protein_targeting_to_membrane” is likewise difficult to interpret since SRP is not defined (signal recognition particle).</td></tr>
<tr><td>Response</td><td>We agree that clarity is critical for a broad Nature readership and have taken several steps to address this in the revised manuscript.

First, we have added a "Glossary of Abbreviations" to the end of the revised manuscript to define all terms. Second, we revised the section "Unified processing and hierarchical cellular taxonomy of the human prefrontal cortex uncovers 27 distinct subclasses of cells," describing the cellular taxonomy, to provide more context and explanation. We opted to create a new nomenclature because a clear, consensus reference taxonomy for the dorsolateral prefrontal cortex (DLPFC) region was lacking. Existing studies of the primate PFC, (including, Ma 2022), derived their taxonomy from 600,000 nuclei and have limited sensitivity to detect rare cell types. These studies utilize different nomenclatures, making direct comparison and adoption challenging. Specifically, there is no established consensus on the hierarchical level of annotation. For example:

* ROSMAP (Mathys 2023) divides top-level annotations into "major cell type" and "cell type."
* SEA-AD (Gabitto 2024) uses three levels: "class," "subclass," and "supertype."
* Ma-Sestan (Ma 2022) also uses three levels: "class," "subclass," and "subtype."

This variability in nomenclature and hierarchical organization is further illustrated by considering specific cell types. For instance, the L2/3 IT cell type is designated as a "subclass" in SEA-AD (Gabitto 2024), a "cell type" (Exc L2-3 CBLN2 LINC02306) in ROSMAP (Mathys 2023), and a "subclass" (L2-3 IT) in Ma-Sestan (Ma 2022). Similarly, the IN_SST cell type is a "subclass" (Sst) in SEA-AD (Gabitto 2024), a "cell type" (Inh L3-5 SST MAFB) in ROSMAP (Mathys 2023), and a "subclass" (SST) in Ma-Sestan (Ma 2022). These examples highlight the inconsistencies and lack of standardization in current PFC cell type annotations. We provide a detailed breakdown of cell type nomenclature in Supplementary Table 2 and a comparison of our taxonomy to public datasets in Supplementary Table 11.

Third, we have revised the figure labels and text where necessary. In Figure 3g, we tried not to modify the original name of the GO term (GO:0006614 SRP-dependent cotranslational protein targeting to membrane). However, we agree with the reviewer that this may not be easily interpretable, so we revised the figure labels and explained the meaning of SRP.

We believe these changes, including the glossary and the more detailed explanations in the text, Supplementary Tables, and figure captions, will significantly improve the manuscript's accessibility for a broader readership while maintaining the rigor of our analysis.</td></tr>
<tr><td>Excerpt</td><td>“...
Glossary of Abbreviations
…
EN: Glutamatergic excitatory neuron
EN_L2_3_IT: Layer 2-3 Intratelencephalic Excitatory Neuron
EN_L3_5_IT_1: Layer 3-5 Intratelencephalic Excitatory Neuron 1
EN_L3_5_IT_2: Layer 3-5 Intratelencephalic Excitatory Neuron 2
EN_L3_5_IT_3: Layer 3-5 Intratelencephalic Excitatory Neuron 3
EN_L5_6_NP: Layer 5-6 Near-Projecting Excitatory Neuron
EN_L5_ET: Layer 5 Extratelencephalic Excitatory Neuron
EN_L6B: Layer 6B Excitatory Neuron
EN_L6_CT: Layer 6 Corticothalamic Excitatory Neuron
EN_L6_IT_1: Layer 6 Intratelencephalic Excitatory Neuron 1
EN_L6_IT_2: Layer 6 Intratelencephalic Excitatory Neuron 2
IT: intra-telencephalic</td></tr>
</table>

	ET: extra-telencephalic<br>NP: near projecting<br>CT: corticothalamic<br>IN: GABAergic inhibitory neuron<br>IN_ADARB2: ADARB2 Inhibitory Neuron<br>IN_LAMP5_LHX6: Ivy cell (LAMP5 LHX6 Inhibitory Neuron)<br>IN_LAMP5_RELN: Neurogliaform cell (LAMP5 RELN Inhibitory Neuron)<br>IN_PVALB: Basket Cell (PVALB Inhibitory Neuron)<br>IN_PVALB_CHC: Chandelier Cell (PVALB CHC Inhibitory Neuron)<br>IN_SST: Martinotti and Non-Martinotti cell (SST Inhibitory Neuron)<br>IN_VIP: VIP Inhibitory Neuron<br>Astro: Astrocyte<br>OPC: Oligodendrocyte Progenitor Cell<br>Oligo: Oligodendrocyte<br>Micro: Microglia<br>PVM: Perivascular Macrophage<br>Adaptive: Adaptive Immune Cell<br>VLMC: Vascular Leptomeningeal Cell<br>SMC: Smooth Muscle Cell<br>PC: Pericyte<br>Endo: Endothelial Cell<br>… ”<br><br>“... **Fig. 3. Sources of transcriptomic variation.** … SRP: signal-recognition particle, HSIAO_HOUSEKEEPING_GENES: housekeeping genes identified as expressed across 19 normal tissues[8], HOUNKPE_HOUSEKEEPING_GENES: 1,130 human and mouse housekeeping genes[9].. …”

REF1-9. Suggested improvements - methodology	
Likewise, some of the text is difficult to understand even by bioinformaticians who may not be familiar with specific packages/methods. For example, lines 277-278 “Using Dreamlet followed by Mashr, we performed composite tests to evaluate the specificity of disease effect leading to the identification.” While referring to these specific software in Methods is fine, having this text without context in the main part of the manuscript leads to problems in interpretation for scientists who don’t understand what Dreamlet or Mashr do.	
Response	We appreciate the reviewer’s valuable feedback. We agree that the previous description was overly technical and may not have been fully accessible to the broad readership of Nature. In response, we have revised the **Results** section to better contextualize the analysis of shared versus disease-specific effects and updated the **Methods** section to more clearly explain the technical details.<br><br>Briefly, after identifying differentially expressed genes (DEGs) separately for each disorder and brain tissue source, we devised a multivariate Bayesian approach to assess the extent to which DEGs are shared across disorders or specific to individual conditions. This meta-analysis allowed us to decompose disease effects into shared and disease-specific components using posterior probability estimates.
Excerpt	“...<br>**Cross-disorder variation of gene expression and genetic concordance**<br>Analyzing gene expression signatures across multiple disorders can reveal shared molecular pathways, facilitating improved diagnostics and targeted therapeutics. As such, we sought to assess the extent of sharing across NDDs and NPDs. Using a pseudobulk approach and precision-weighted regression modeling for large-scale single cell datasets, implemented in the dreamlet software[10] (**Methods**), we performed a comprehensive analysis of differentially expressed genes (DEG) across 8 different NDDs and NPDs. After identifying DEGs for each disease contrast and tissue source, we performed a standard frequentist fixed-effect meta-analysis across the 3 tissue sources to define consensus disease trait signatures. We then applied a multivariate Bayesian meta-analysis[10,11] across cell types and disease traits to produce posterior estimates of the effect size and the posterior probability that each effect is non-zero. Using a composite testing approach, we used these results to estimate posterior

	probabilities that each effect is specific to a trait or shared across traits. We then evaluated, in a cell-type-specific manner, cross-disease sharing and decomposed the total disease effects into shared and disease-specific components (**Supplementary Table 4**)."<br><br>"...<br>**Evaluation of shared disease signatures**<br>We modeled the total disease signature for a given condition as the sum of shared and disease-specific DEGs:<br>$\Delta\tau_k = \sum_{i \in G_{\text{shared}}} \Delta\tau_{i,k} + \sum_{j \in G_{\text{distinct}}} \Delta\tau_{j,k}$<br>where $\Delta\tau_k$ denotes the total disease signature for a disease k, G_{shared} denotes a set of genes having cross-disorder effects, and G_{distinct} denotes a set of genes with non-shared (disease-specific) effects. This notation highlights that DEG profiles for each disease arise from contributions of both shared genes (common to all diseases) and distinct genes (specific to each disease).<br>To determine how DEG effects are shared across cell types and disease, we applied a multivariate Bayesian meta-analysis approach using the mashr software[11]. The software uses a Bayesian approach to shrink effect sizes across genes and cell types in order to estimate the posterior effect sizes and posterior probability that an effect has the correct sign. In accompanying work, we have extended this approach to develop a formal statistical test to identify cell type- and disease-specific effects[12]. For this analysis, we conducted composite tests in a cell-type-specific manner, assessing the probability that a gene exhibits a non-zero effect across all eight disease contrasts. Genes with a posterior probability ≥ 0.05 were classified as part of the shared component."

REF1-10. Suggested improvements - biological contexts	
In areas where specific genes are identified with biological interpretation, the genes should be identified by name, for example, there is extensive discussion of ARL17B (lines 214-220) yet this gene is never named.<br><br>On line 205, Chandelier cells are noted, please explain that these are a subset of GABAergic interneurons.<br><br>In some parts of the manuscript, some stated results need to be put in clearer context with prior studies. For example, lines 301-302 – "We observed a positive correlation between genetic and transcriptomic similarities…". While this result is reported with a specific correlation coefficient, it would be helpful to place the result in context of prior studies that examined genetic and transcriptomic similarities and differences.<br><br>The section on - non-linear dynamics of the AD pathological trajectory is interesting but could be placed in a clearer context with the overall design of the study to examine the transcriptome across diseases.	
Response	We agree that providing full gene names and placing results in context with prior studies would improve the readability of the manuscript. In addition to addressing terminology and abbreviations (see **REF1-8**), we have thoroughly revised the manuscript to incorporate full gene names where appropriate and to explicitly state that Chandelier cells are a subset of GABAergic interneurons. Furthermore, we rewrote the section on the heritability-transcriptome concordance to place our results in context with prior studies. Lastly, we concur that incorporating biological context into our discussion of the non-linear dynamics of AD would be beneficial to the reader. To address this point, we have included several sentences (see **Excerpt**) that summarize recent findings regarding the transcriptomic and cellular alterations occurring during AD progression. We have also added more context explaining our design choice rationale for this section, in addition to augmenting the results and analysis. For full details, please see the response **REF2-7**.
Excerpt	"... Notably, we observed that inter-individual differences explained 82.2% of the variation in expression of the ADP Ribosylation Factor Like GTPase 17B (ARL17B) gene (**Fig. 3d**). Several adjacent genes, such as KAT8 Regulatory NSL Complex Subunit 1 (KANSL1) and ARL17A (a paralog of ARL17B), also had high inter-individual variation

	(**Supplementary Fig. 6c**). ARL17B and KANSL1 genes are localized in the disease-associated MAPT locus (17q21.31), are often found as a fusion transcript (KANSL1::ARL17B), frequently undergo polymorphic translocation[13,14] and have been implicated in neurological disorders, including ALS, PD, and MS[15–18]."<br><br>"… Among all cell types, Chandelier cells (IN_PVALB_CHC), a subset of GABAergic interneurons, had the highest concordance with the pairwise trait heritability. …"<br><br>"...<br>**Cross-disorder variation of gene expression and genetic concordance**<br>… By correlating the shared heritability against the average transcriptome similarity across cell types, we obtained a heritability-transcriptome concordance (r_g-ρ_s), which indicated a strong positive correlation (Spearman's ρ=0.604) between genetic and transcriptomic similarities (**Fig. 5f, Supplementary Fig. 8f**). The results show that diseases with higher genetic overlap also tend to have more similar transcriptomic dysregulation, suggesting that shared transcriptomic vulnerabilities are coupled with underlying genetic architecture, an unsurprising result that corroborates previous observations[19–21]."<br><br>"...<br>**Nonlinear dynamics of the AD pathological trajectory**<br>… Recent studies have begun to map the time course of transcriptomic and cellular changes during AD progression. Early events include increased inflammation, astrocyte reactivity, and blood-brain barrier dysfunction, along with heightened vulnerability in select neuronal populations. These are followed by a more dysregulated immune response and broader neuronal loss."

REF1-11. References	
References. Appropriate credit is given for previous work.	
Response	Within the confines of the manuscript, we believe that the references used are appropriate, up to date and accurate.

REF1-12. Limitations of the study	
Clarity and context. As noted in 'suggested improvements' the manuscript requires substantial revision so that the results and main conclusions can be understandable by readers with broad scientific backgrounds. No study limitations are noted and this is a weakness. One important limitation is that all of this analysis and the Atlas itself are for one region of the brain, the dorsolateral frontal cortex. While this fact does not negate the value of the Atlas or the paper, it is a limitation and important caveat for interpretation of the data in context of the diseases considered.	
Response	We agree with the reviewer that limitations and caveats of the study were not discussed sufficiently. We have amended the discussion in the revision (see **Excerpt**).
Excerpt	"While the human brain comprises numerous regions with distinct functions, in this study we limited our analysis to the DLPFC. Future studies that include additional brain regions will provide insights into a broader range of phenotypes and offer a more holistic view of the molecular foundations of brain function in both health and disease. Moreover, more detailed spatial analyses, including whole-cell transcriptomics, could further elucidate the physiological contexts that contribute to disease. Analyzing larger numbers of cells may also uncover rarer disease-associated cell types or subtypes, while integrating additional omics modalities (e.g. proteomics or epigenomics) will provide deeper molecular insights."

Referee #2 (Remarks to the Author):

REF2-1. General remarks - reporting of data processing and analyses	
In this study, Lee et al. curate and analyse single-nuclei transcriptomic profiles of prefrontal cortex from > 1000 individuals affected by several complex brain disorders. They annotate cell types and perform comparative analyses within and between disorders, to identify genes and cell types involved in pathological processes, with a specific focus on Alzheimer's disease.<br><br>Processing and curating such a rich dataset is a substantial effort, and it's likely that many practitioners in single-cell genomics and neuroscience will find this dataset invaluable for further analyses. The integration of evidence from tissue molecular features, human genetics, and clinical data is both timely and relevant, representing a natural and necessary progression for single-cell tissue profiling studies. However, the reporting of data processing and analyses lacks the thoroughness needed to fully understand the dataset. As it stands, this limitation reduces the dataset's potential as a widely accessible resource.	
Response	We appreciate your positive feedback and recognition of the originality and significance of our work. We agree this atlas is a valuable resource for researchers interested in single-cell resolution data for neurodegenerative and neuropsychological disorders.<br><br>We also want to highlight that this study is part of a larger package of papers (see https://psych-ad.org), including analyses that delve deeper into specific subject areas such as healthy aging, QTL, TWAS, and disease phenotyping. These complementary studies provide further insights into the complex landscape of human brain disorders.<br><br>We believe that the combined impact of these studies will be substantial, offering a comprehensive understanding of these conditions and paving the way for new therapeutic strategies.

REF2-2. General remarks - investigation of existing literature	
While I am not an expert in neuropathology and therefore cannot fully assess the novelty of the associations between cell subtypes, genes, and disease, I believe that the study would benefit from a more in-depth investigation of a few prioritized cell types and genes, beyond gene ontology enrichment analyses. Although I recognize that collecting additional experimental data may be unfeasible, there is a wealth of publicly available complementary clinical and genomics data on Alzheimer's Disease (AD). At the very least, the results should be discussed in the context of the extensive literature on single-cell genomics and human genetics of AD to enhance their interpretative value.	
Response	We agree that comparing our findings to existing literature is crucial for contextualizing our results and highlighting novel contributions. We addressed the reviewer's suggestions in the revised manuscript as follows:<br><br>1. We improved the robustness and reliability of our conclusions by replicating our findings using publicly available data. We have now performed a replication of our DEG analysis using two additional single-cell datasets focused on AD in the human prefrontal cortex: the ROSMAP (Mathys et al., 2023[6]) and SEA-AD (Gabitto et al., 2024[7]) studies. Overall, we successfully replicated our key findings and observed highly concordant disease signatures across these independent datasets (**Supplementary Figs. 12a-e**). We have updated the manuscript to include these results and discuss their implications. This additional analysis strengthens the robustness of our conclusions and provides further support for the validity and reliability of our findings.

	2. We compared our mediation analysis to previous findings, particularly with Green et al. (2024)[22]. While our mediation analysis was inspired by previous studies, there are several key methodological and conceptual differences that lead to distinct and, we believe, complementary findings:<br><br>**a. Anchoring Genetic Risk**: Green et al. (2024) utilized the APOE ε4 genotype as their primary anchor for genetic risk. In contrast, our study employed a polygenic risk score (PRS) derived from the latest AD GWAS by Bellenguez et al. (2022). While the APOE ε4 allele remains by far the strongest single genetic risk factor for AD, it captures only a subset of the total heritable risk. Genetic risk is continuous and it has been shown that PRS improves prediction beyond APOE alone by capturing additional polygenic contributions, offering meaningful stratification even among APOE4-negative individuals[4,5].<br><br>**b. Cell Type Specificity and Breadth**: Green et al. (2024) focused on four specific subpopulations: two microglia subtypes (Mic.12 and Mic.13), one astrocyte subtype (Ast.10), and one oligodendrocyte subtype (Oli.7). Our analysis, however, considered a broader range of cell types. We initially included all 14 cell subclasses that showed significant alterations in cell type composition in any of the AD contrasts (FDR < 0.05). From this wider set, we identified four cell subclasses (Microglia, VLMC, IN_SST, and IN_LAMP5_LHX6) exhibiting significant mediation effects (P < 0.05). This broader initial screen allows for the discovery of mediating roles for cell types that might be overlooked when focusing on a predetermined subset.<br><br>Overall, both our study and that of Green et al. (2024) identified Microglia as significant mediators of AD risk. This corroborates the established importance of microglial dysfunction in AD pathogenesis and provides further support for the role of these cells in translating genetic risk into disease phenotypes. However, Green et al. (2024) emphasized the mediating effects of specific glial subtypes (Oligodendrocytes and Astrocytes). While our analysis does not preclude a role for these cell types, our broader approach, combined with the use of a PRS, highlighted the significant mediating effects of vascular cells (VLMC) and two subclasses of interneurons (IN_SST and IN_LAMP5_LHX6). These findings complement, rather than contradict, the findings of Green et al., suggesting that the increased power of PsychAD cohorts can reveal distinct, yet potentially interacting, cell-types that mediate AD risk.
Excerpt	"... In general, DEGs had high concordance across different AD pathology variables, consistent with previous reports[6], and the disease signatures were highly concordant with previous studies[6,7] (**Supplementary Figs. 12a-e**). …"

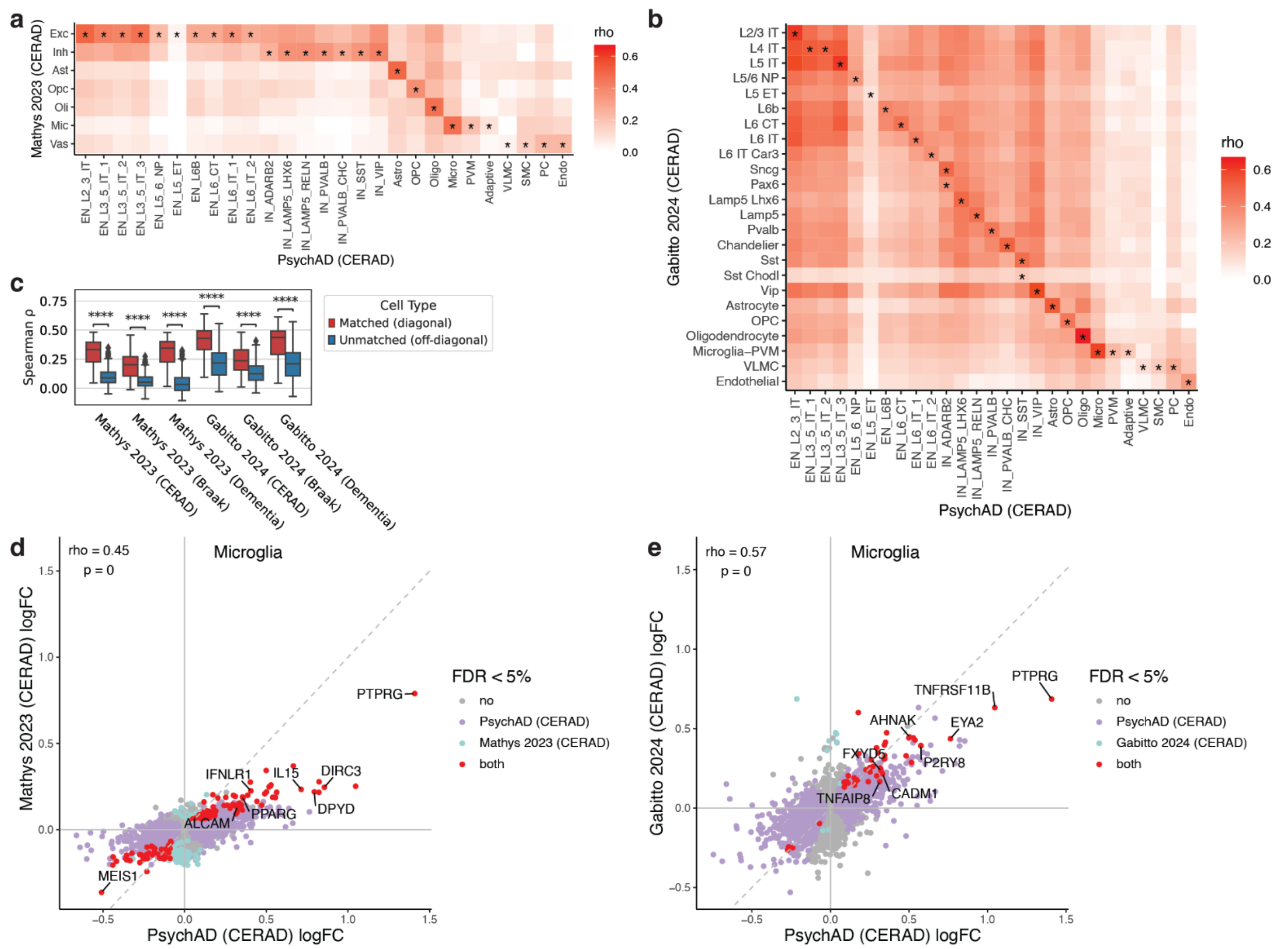

Supplementary Fig. 12. (a) Comparison of differential expression signatures with previous studies – ROSMAP (Mathys et al. 2023)[6] and SEA-AD (Gabitto et al. 2024)[7]. Pairwise Spearman correlation between PsychAD subclass and ROSMAP Major Cell Type. CERAD as a measure of disease severity. Asterisks indicate the best matching cell type based on **Supplementary Table 11**. **(b)** Pairwise Spearman correlation between PsychAD subclass and SEA-AD subclass. CERAD as a measure of disease severity. Asterisks indicate the best matching cell type based on **Supplementary Table 11**. **(c)** Comparison of various disease signatures (CERAD, Braak, Dementia) between matched (diagonal, asterisks) and unmatched (off-diagonal) cell types. Mann–Whitney U test. ****: $p \leq 1.0e\text{-}4$. **(d)** Comparison of differentially expressed genes between PsychAD Microglia and ROSMAP Mic using CERAD as a measure of disease severity. Spearman correlation (rho) shown. **(e)** Comparison of differentially expressed genes between PsychAD Microglia and SEA-AD Microglia-PVM using CERAD as a measure of disease severity. Spearman correlation (rho) shown.

REF2-3. Presentation of the dataset

The curated dataset presented in this study has significant potential to be highly impactful. However, the presentation of dataset features and patient metadata feels somewhat synthetic; many details are either missing or difficult to parse, and the methods section for snRNA-seq preprocessing lacks sufficient detail. Here are some specific suggestions:

- In Figure 1a: The circular visualization may not be the most effective choice. The section of the circle dedicated to each cohort is not proportional to the cohort size, which can be confusing. I suggest using a multi-panel linear barplot instead. One panel could display barplots showing percentages (including the number of donors for each category), while another could show the age distribution. This would also make it easier to label the y-axis of the female/male plot, which is currently missing. Additionally, the annotation "SNP" is unclear; consider labeling the y-axis as "% available genotype data."

- The outer bar of figure 1a denotes the number of diagnoses, but providing clear numbers for how many individuals with each diagnosis are present in the cohort would be very helpful. This information is partially shown in Figure 1c but only for a subset of the cohort. What is the rationale for excluding certain samples?
- Figure 1e: The figure could be more informative with better legend descriptions. What distances were used to construct the dendrogram? What is the relationship between the Venn diagrams and the dendrogram, aside from the labels? Consider splitting this into two panels with adequate descriptions in the legend. To facilitate comparison, replacing the Venn diagram with a colored UpSet plot (as in Figure 1c), with bars colored by sex, could be more effective.

- Including more plots from the quality control and preprocessing analyses described in the methods would be beneficial. For example, line 611 mentions that some donors were excluded for low counts, but Supplementary Figure 1h-i only shows the number of cells per sample/donor in aggregate. Could this be broken down by donor, showing which were excluded and how they compare to the rest of the cohort?
- Line 608: “We checked for possible contamination of ambient RNA” - how was this check performed?
- Can the description of the batch correction steps be expanded? Which covariates were used for batch correction? Adding a supplementary figure showing that no donor/cohort-specific cell subtypes remain after integration would be valuable.
- The methods section only describes clustering but does not explain the procedure used for cell type annotation. Was this done through manual inspection of marker genes? Please specify the set of markers used, provide references, and refer to relevant figures (e.g., Figure 2d). Including key marker genes used for annotation in Supplementary Table 2 would greatly enhance the utility of this resource.

- Unfortunately, the methodological details mentioned above are difficult to ascertain from inspecting the code repository. The steps also do not match between the methods section and the code. For instance, the preprocessing code includes a linear regression step (see https://github.com/DiseaseNeuroGenomics/PsychADxD/blob/a8143ffa1ad967619732aa6cf342df4b10786c7d/2_taxonomy/PsychAD_pegasus_end2end_2_pass.py#L64) that is not described in the methods. Furthermore, it appears that batch correction with Harmony is performed before, not after, doublet detection. Please ensure that the methods section accurately reflects the analysis steps outlined in the code.

Response

We thank the reviewer for the valuable feedback regarding the presentation of dataset features and patient metadata, as well as the suggestions for improving figures.

1. Regarding Fig. 1a - the presentation of dataset features and patient metadata: In response, we have revised **Fig. 1** as well as added a new **Supplementary Fig. 2**, which contains additional plots to provide a more comprehensive and intuitive overview of the dataset. Specifically, we summarized donor metadata across brain sources using bar plots, boxplots, violin plots, and scatter plots. These visualizations highlight distributions of diagnosis by brain source (**Supplementary Fig. 2b**) and sex (**Supplementary Fig. 2c**), distributions of age by diagnosis (**Supplementary Fig. 2d**), brain source (**Supplementary Fig. 2e**), and sex (**Supplementary Fig. 2f**), distributions of genetic ancestry by brain source (**Supplementary Fig. 2g**) and sex (**Supplementary Fig. 2h**), and availability of genotypes by brain sources (**Supplementary Fig. 2i**), making the full scope of demographic and clinical data readily accessible. We also revised the representation of genotypic data availability by updating the y-axis label to "Genotyped - Yes/No".

2. Regarding Fig. 1c - rationale for excluding certain samples: We further clarified our rationale for sample exclusion in the revised manuscript. We added a sentence to both the main text and the figure legend of **Fig. 1c** to make it more explicit (see **Excerpt**). This clarification helps define the rationale for subset selection and reinforces the robustness of the cross-disorder analysis. In addition, we provide more comprehensive summary of all diagnoses identified within the PsychAD cohort, along with the classification criteria used by each brain bank, in the Table S1 of PsychAD data descriptor manuscript[23].

3. Regarding Fig. 1e - replacing the Venn diagram with the UpSet plot: In the original manuscript, we provided the UpSet plot with bars colored by sex as **Supplementary Fig. 1b**. However, we agree with the reviewer that the UpSet plot would be more effective than the Venn diagram in **Fig. 1e**. In the revised manuscript, we replaced the Venn diagram with a colored UpSet plot, as suggested (**Fig. 1e**).

	This new visualization provides a clearer and more detailed representation of the overlaps between diagnostic groups, with bars colored by sex to facilitate direct comparison across groups.<br><br>**4. Regarding QC and cell type annotation**: To address the reviewer's comments on quality control and preprocessing, we have now included a **Supplementary Table 12** detailing the QC statistics (median gene and UMI counts, median percent mitochondrial genes, and cell counts), separated by 2,924 single-cell libraries utilized, before and after removal of low-quality cells. This allows for a clearer understanding of the impact of our QC pipeline. We also improved the **Methods** section to further clarify the processing steps we took to analyze the snRNA-seq data. To directly answer the reviewer's question, we checked for possible contamination from ambient RNA using CellBender[24]. We also addressed the reviewer's comment regarding the batch correction steps. More specifically, we performed harmony batch correction before and after the doublet removal step. First, to prepare input for the doublet inference step, we performed the first pass Leiden clustering analysis on the whole dataset. After doublets were removed, we then performed the second pass Leiden clustering analysis as part of the iterative clustering procedure. In fact, the batch correction procedure was applied on every iteration of clustering analysis where we performed the highly variable gene selection after correcting for tissue source bias, regressing out technical effects of UMI counts, mitochondrial percentage, and the cell cycle after PCA, and harmony correction based on sequencing batch. The resulting three-level hierarchical cellular taxonomy was free of donor or tissue source specific bias. **Supplementary Figs. 3a-b** illustrate that the compositions of subclass or subtype level annotations were stable across tissue sources. We also improved the description of the procedure used for cell type annotation (see **Methods** section titled "Defining cellular taxonomy using iterative clustering"). In short, instead of relying on a set of marker genes, the resulting hierarchical clusters were aggregated into pseudobulk samples, and then we calculated cluster-wise Pearson correlation coefficients using the annotations from existing human DLPFC[25] and M1[26] annotations. Cell type annotations were assigned based on a combination of high correlation and cell type specificity, with the results summarized in **Supplementary Table 11**.<br><br>**5. Regarding the code repository**: Finally, we improved the code repository by restructuring the scripts into five processing steps and adding more documentation (see **REF2-12**). They are publicly available and accessible at: https://github.com/DiseaseNeuroGenomics/PsychADxD/tree/main/2_taxonomy. These extensive updates, including text, extended data figures, supplementary figures, and tables, aim to address all of the reviewer's concerns, improving the manuscript's clarity, consistency, and utility for the broader research community. We thank the reviewers again for their insightful comments, which have been instrumental in enhancing the quality of this work.
Excerpt	"... First, we performed cross-disorder analyses (**Fig. 1c**). To ensure analytical power and interpretability, we focused on eight well-represented disorders with clear pathological definitions and sufficient donor representation, including balanced numbers of cases and controls. In detail, using a subset of 1,160 donors, aged ≥ 17, with minimal comorbidities, we targeted six neurodegenerative diseases (NDDs; AD, DLBD, Vas, Tau, FTD, and PD) and two neuropsychiatric diseases (NPDs; SCZ and BD). To estimate the sharing of transcriptomic vulnerability, we compared donors affected with NDDs and NPDs against the baseline of 319 neurotypical controls. …"

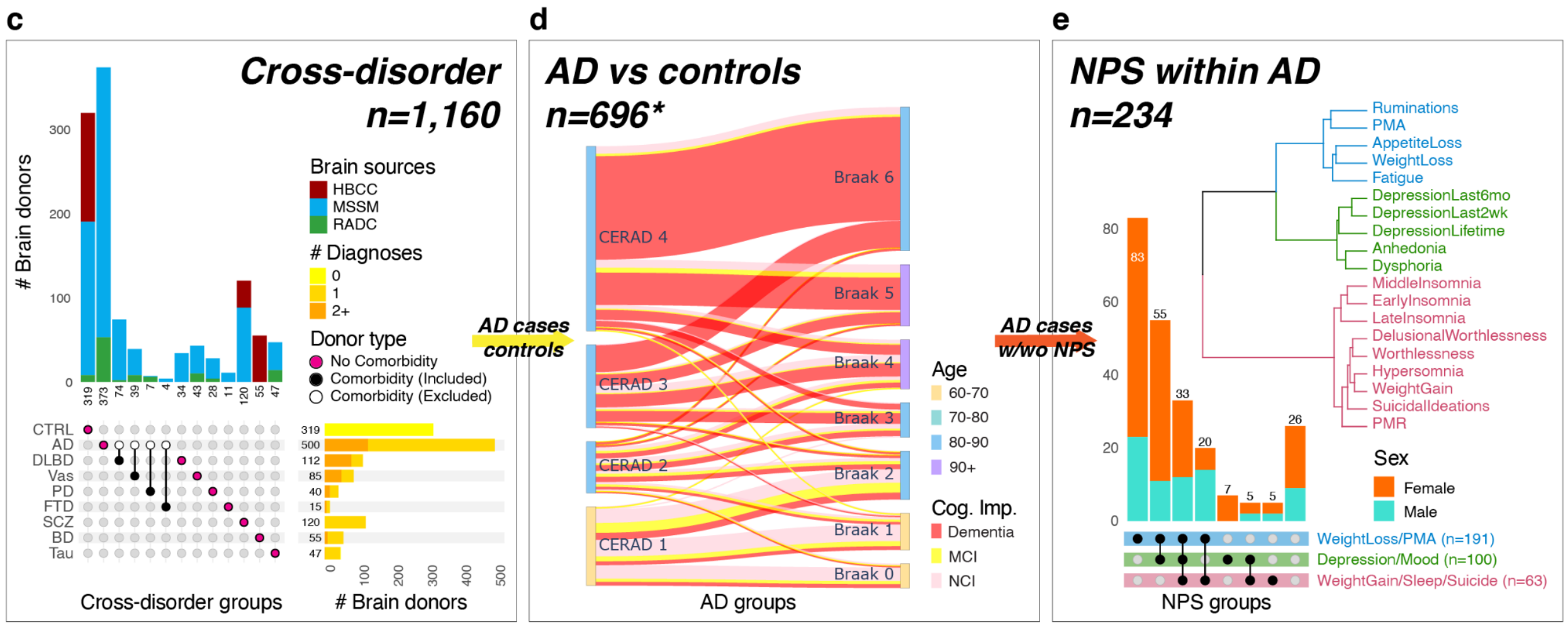

*54 donors with incomplete pathology or cognitive status omitted

Fig. 1. Overview of the PsychAD cohort and study design. … **(c)** A subset of the PsychAD cohort (n=1,160 donors) focused on cross-disorder contrasts. This subset includes a group of neurotypical controls (n=319), and donors diagnosed with one of eight NDD and NPDs. The bar plot shows the total number of brain donors per cross-disorder group, stratified by brain sources (top) or by the number of diagnoses per donor (right). The UpSet plot (bottom left) illustrates unique and overlapping disorder phenotypes, highlighting patterns of comorbidity. For AD contrasts, comorbid cases were not considered. For non-AD contrasts, comorbid cases were included to ensure sufficient statistical power for the analysis. Due to the inclusion of both comorbid and non-comorbid cases, there is an overlap in donor counts across disorder groups. As a result, the total number of donors represented in the plot may exceed the actual number of unique donors, reflecting instances where the same donor appears in multiple diagnostic intersections. **(d)** A subset of the PsychAD cohort (n=696) focused on AD phenotype contrasts. Comparison of different measures of AD severity, including neuritic plaque density (CERAD), NFT pathology (Braak), and cognitive impairments. *n=54 donors with incomplete pathology or cognitive status were omitted from the visualization. **(e)** A subset of the AD cases (n=234) was used to characterize co-occurrence of NPS with AD. Columns represent the intersection of NPS categories and vertical bars indicate the number of donors in each intersection. Horizontal bars represent the total number of donors within each individual NPS category: Depression/Mood (n=100) - depression and mood changes, WeightLoss/PMA (n=191) - weight loss and psychomotor agitation (PMA), WeightGain/Sleep/Suicide (n=63) - weight gain, sleep disturbances, suicidal ideation, guilt, and psychomotor retardation (PMR), and No NPS - representing AD donors without any recorded NPS. Donor proportions are further stratified by sex.

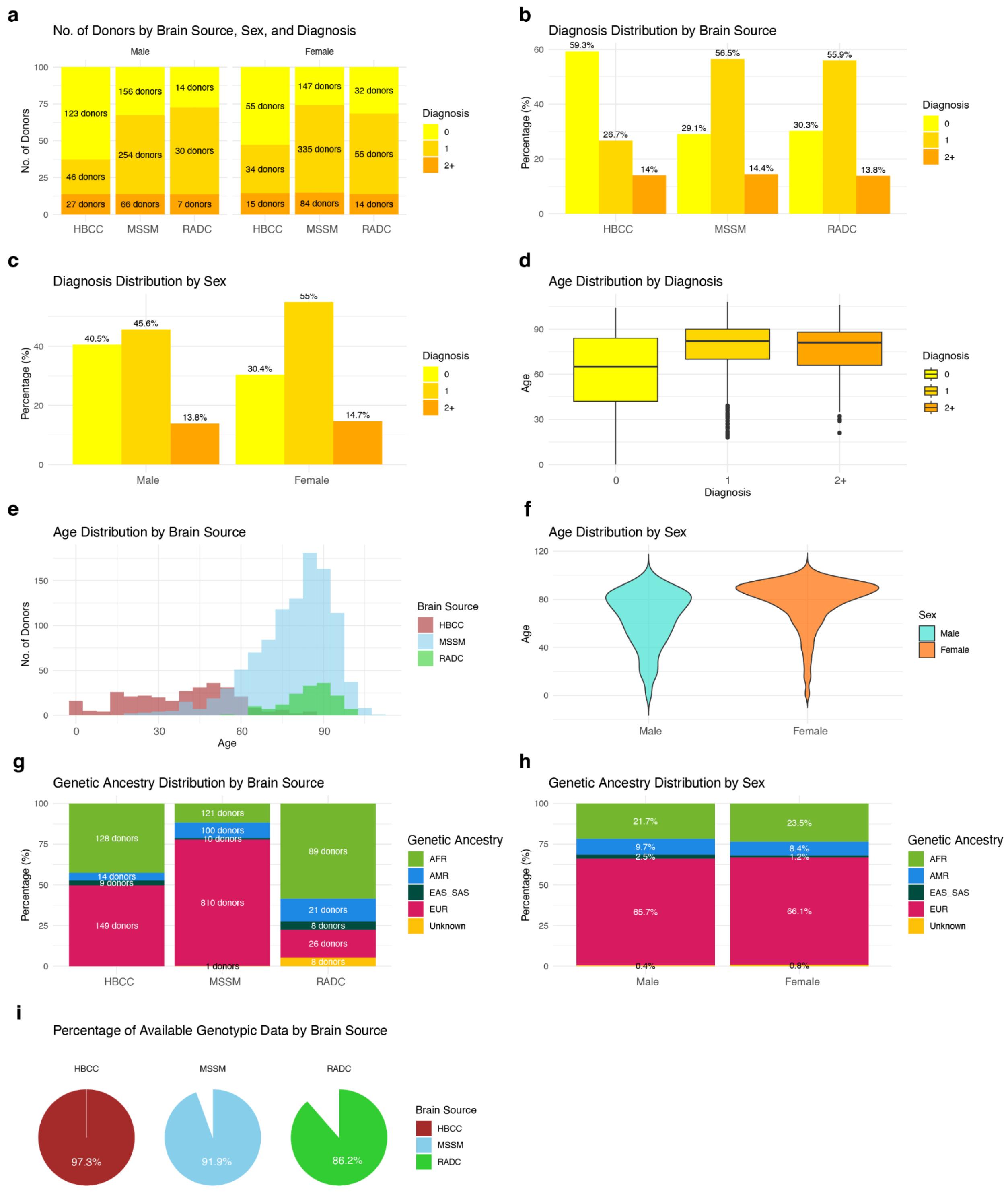

a
No. of Donors by Brain Source, Sex, and Diagnosis
Male
Female
No. of Donors
123 donors
156 donors
14 donors
55 donors
147 donors
32 donors
46 donors
254 donors
30 donors
34 donors
335 donors
55 donors
27 donors
66 donors
7 donors
15 donors
84 donors
14 donors
HBCC
MSSM
RADC
Diagnosis
0
1
2+
b
Diagnosis Distribution by Brain Source
Percentage (%)
59.3%
26.7%
14%
29.1%
56.5%
14.4%
30.3%
55.9%
13.8%
c
Diagnosis Distribution by Sex
40.5%
45.6%
13.8%
30.4%
55%
14.7%
Male
Female
d
Age Distribution by Diagnosis
Age
Diagnosis
e
Age Distribution by Brain Source
No. of Donors
Age
Brain Source
HBCC
MSSM
RADC
f
Age Distribution by Sex
Sex
Male
Female
g
Genetic Ancestry Distribution by Brain Source
128 donors
14 donors
9 donors
149 donors
121 donors
100 donors
10 donors
810 donors
1 donors
89 donors
21 donors
8 donors
26 donors
8 donors
Genetic Ancestry
AFR
AMR
EAS_SAS
EUR
Unknown
h
Genetic Ancestry Distribution by Sex
21.7%
9.7%
2.5%
65.7%
0.4%
23.5%
8.4%
1.2%
66.1%
0.8%
i
Percentage of Available Genotypic Data by Brain Source
97.3%
91.9%
86.2%

	**Supplementary Fig. 2**. Comprehensive demographic and extended overview of the PsychAD cohort. This figure provides a detailed breakdown of the demographic, diagnostic, and genotypic characteristics of donors from three brain sources (HBCC, MSSM, and RADC). **(a)** Bar plots showing the number of donors by brain source, stratified by sex (male and female) and diagnosis (0: no diagnosis, 1: single diagnosis, 2+: multiple diagnoses). **(b)** Percentage distribution of donors by brain source, stratified by number of diagnoses (0, 1, and 2+). **(c)** Percentage distribution of diagnoses (0, 1, and 2+) by sex (male and female), highlighting diagnostic differences between sexes. **(d)** Box plots of age distribution by diagnosis category (0, 1, and 2+), illustrating the relationship between age and diagnostic burden. **(e)** Histogram of age distribution by brain source, showing the variation in donor ages for HBCC, MSSM, and RADC cohorts. **(f)** Age distribution by sex (male and female) across all donors. **(g)** Distribution of genetic ancestry across the three brain sources; AFR (African), AMR (Admixed American), EAS_SAS (East and South Asian), EUR (European), and Unknown. The number of donors in each category is indicated. **(h)** Distribution of genetic ancestry by sex (male and female), with percentages displayed for each genetic ancestry category. **(i)** Percentage of donors with available genotypic data per brain source.<br><br>**Defining cellular taxonomy using iterative clustering**<br>**Hierarchical cellular taxonomy**. Cellular taxonomy was defined using a divide-and-conquer strategy. From the full dataset containing over 6 million nuclei, 8 major cell classes were defined using the following steps: 6,000 highly variable genes (HVGs) were selected from mean and dispersions trends[27] using the default parameters (min_mean=0.0125, max_mean=3, min_disp=0.5) and brain source as a batch variable after manually excluding sex and mitochondrial chromosomes. We used the k-nearest-neighbor (kNN) graph calculated on the basis of harmony-corrected PCA embedding space to cluster nuclei of the same cell type using Leiden[28] clustering algorithms. We used UMAP[29] to visualize the resulting clusters. From the class-level clusters, we subsetted the data by each class. Re-calculating HVGs among cells in the same class allowed us to re-focus on a feature space that is more relevant for the same class of cells. A kNN graph was then calculated on the basis of the harmony-corrected PCA of the selected HVGs. Leiden-clustering was used to annotate 27 subclass-level annotations. We iterated the same HVG-kNN-Leiden clustering for all 27 subclasses yielding 67 subtypes of human brain cells. After obtaining annotations in three-levels of hierarchy, the resulting clusters were aggregated into pseudobulk and cluster-wise Pearson correlation coefficient was calculated using existing human DLPFC[25] and M1[26] annotations. We matched the annotations based on both high correlation and specificity of the cell type. The final cellular taxonomy was compared to the following snRNA-seq datasets: PFC from ROSMAP cohort[6] and PFC (Brodmann area 9) from SEA-AD cohort[7] (**Supplementary Table 11**).

REF2-4. Gene expression variance analysis	
(2) The variance analysis in figure 3 is potentially very useful to understand the features of the dataset. However, the observation that variable genes are mostly housekeeping genes makes me slightly concerned that the model is biased towards ubiquitously highly expressed genes.<br><br>Can the authors show the relationship between model coefficients and the mean expression across datapoints (MA plot)? Could the authors provide an MA plot to illustrate the relationship between model coefficients and mean gene expression across datapoints? It may be beneficial to recalibrate the analysis to account for expected gene variability across tissues and populations (see related work: https://www.biorxiv.org/content/10.1101/2024.04.10.588830v1).<br><br>Another concern is that variability is driven by compositional differences (described in figure 4). Additionally, having asserted that the main driver of gene expression are cell type differences, would this analysis be more informative if repeated for each cell type? See Ramirez Flores et al. (https://elifesciences.org/articles/93161) for a related approach.	
Response	We appreciate the reviewer’s concern regarding potential bias toward ubiquitously expressed housekeeping genes in the VariancePartition (VP) analysis. While it is true that highly expressed genes may exhibit higher absolute variance, our analysis does not prioritize genes solely based on variance

	magnitude. Instead, we focus on the relative contribution of biological and technical covariates to gene expression variability.<br><br>**1. MA plot for VP analysis**: In differential expression analysis, an MA plot shows the relationship between estimated log fold change (logFC) and expression magnitude. While the logFC should be centered around zero for all expression levels, lowly expressed genes have higher variation in logFC and so have high mean absolute logFC values. Here we consider the variation partitioning analysis examining expression variation across subclasses within each subclass and show the variance explained by donor cell type and residuals for each gene as a function of mean expression in terms of log2 counts per million (**Supplementary Fig. 5h**). In VP analysis, genes with higher expression have more reads so they have less random noise, are measured more precisely and thus have higher biological signals. We observed increasing gene expression correlates with increased variation explained across donor and cell type and less explained by residuals across all cell classes. This is a technical rather than a biological effect.<br><br>**2. Recalibrating differential gene expression**: We appreciate the reference to recent work by Rentzsch et al.[30] and agree with the principle of accounting for expected gene variability. The recalibration analysis designed to account for gene expression variability of Rentzsch et al. is an interesting idea. They develop a method that accounts for "relative change in comparison to natural dosage variation for each gene" in order to address the limitations of log fold changes and p-values. Indeed, this has a similar motivation as VP analysis. They estimate parameters from their model using bulk tissue RNA-seq from GTEx and provide gene-level statistics for 49 tissues. They also provide gene-level statistics averaged across all tissues. We agree that recalibration is an intriguing idea, but we are concerned that existing statistics were computed from bulk tissue from GTEx and do not represent our gene expression from single nucleus brain data very well.<br><br>Applying the recalibration method to the differential expression results from AD using the best matching tissue, Brain - Frontal Cortex (BA9), gives recalibrated log fold changes that have a 0.944 - 0.967 Spearman correlation with the original (**Figure below**). Given this, the recalibration has only a modest effect on the differential expression results. While this might match some of the biology of common cell types like oligodendrocytes, low frequency cell types are not well captured by this approach.

	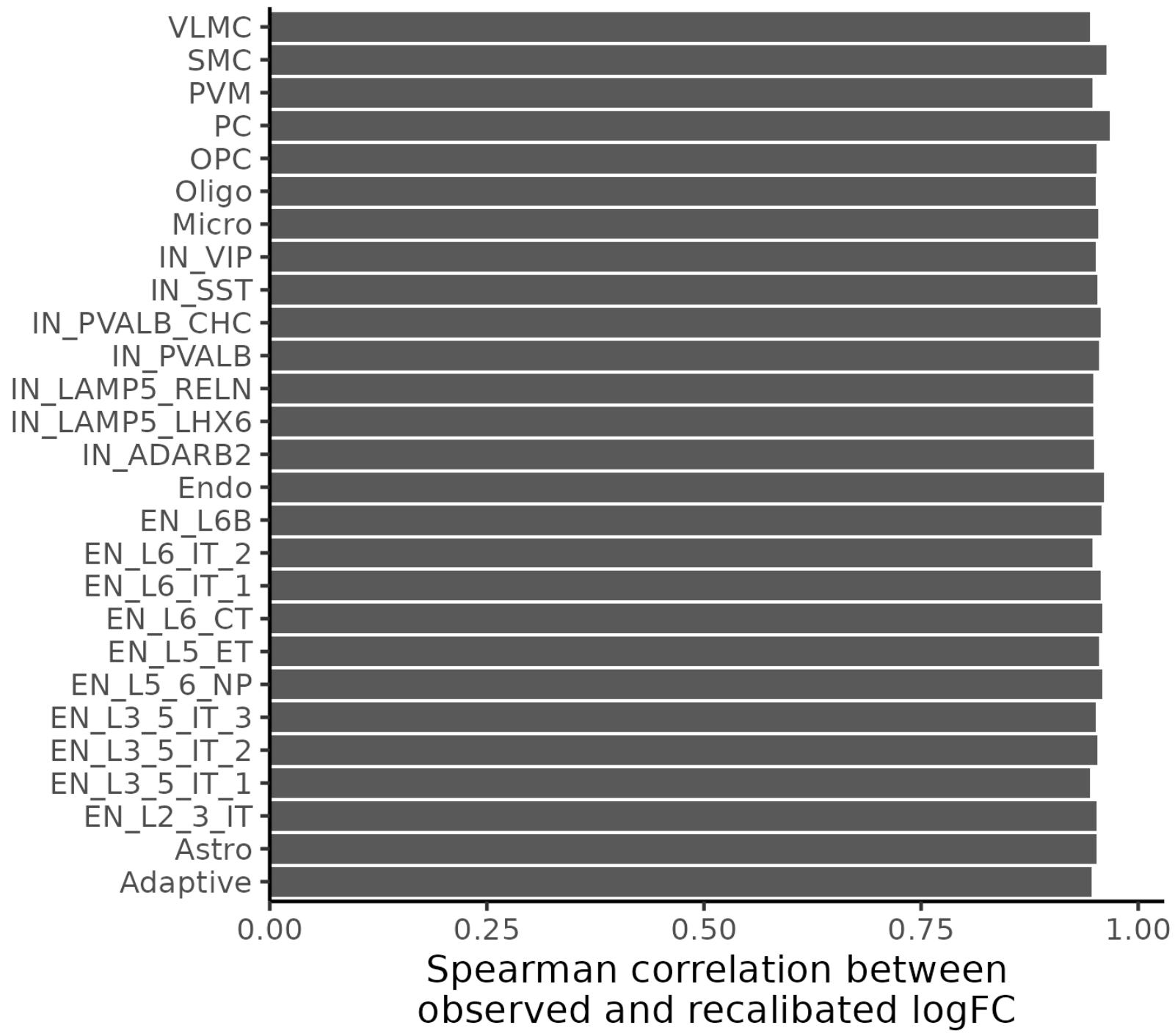 <br>**3. Repeating VP analysis per cell type (Ramirez Flores et al., eLife 2024)**: By modeling variance within specific cell classes (e.g. IN, EN, Non-Neuron), we reduce the confounding impact of across-cell-type composition, focusing instead on within-cell-type transcriptional variation. We note that this approach is more aligned with the reviewer's suggestion and improves interpretability of covariate-specific effects. To address this concern more directly, we performed a stratified VP analysis within stacked assays for individual cell classes (e.g. EN, IN, and Non-Neuron) (**Supplementary Figs. 6j,k**, see our response to **REF3-5** for more details). This design reduces the confounding impact of cross-cell-type expression levels and ensures that comparisons are made within more transcriptionally homogeneous populations. As a result, the updated analysis reveals covariate-specific variance patterns that are not driven merely by overall expression level but by biologically relevant sources of variability within each cell class.

Excerpt

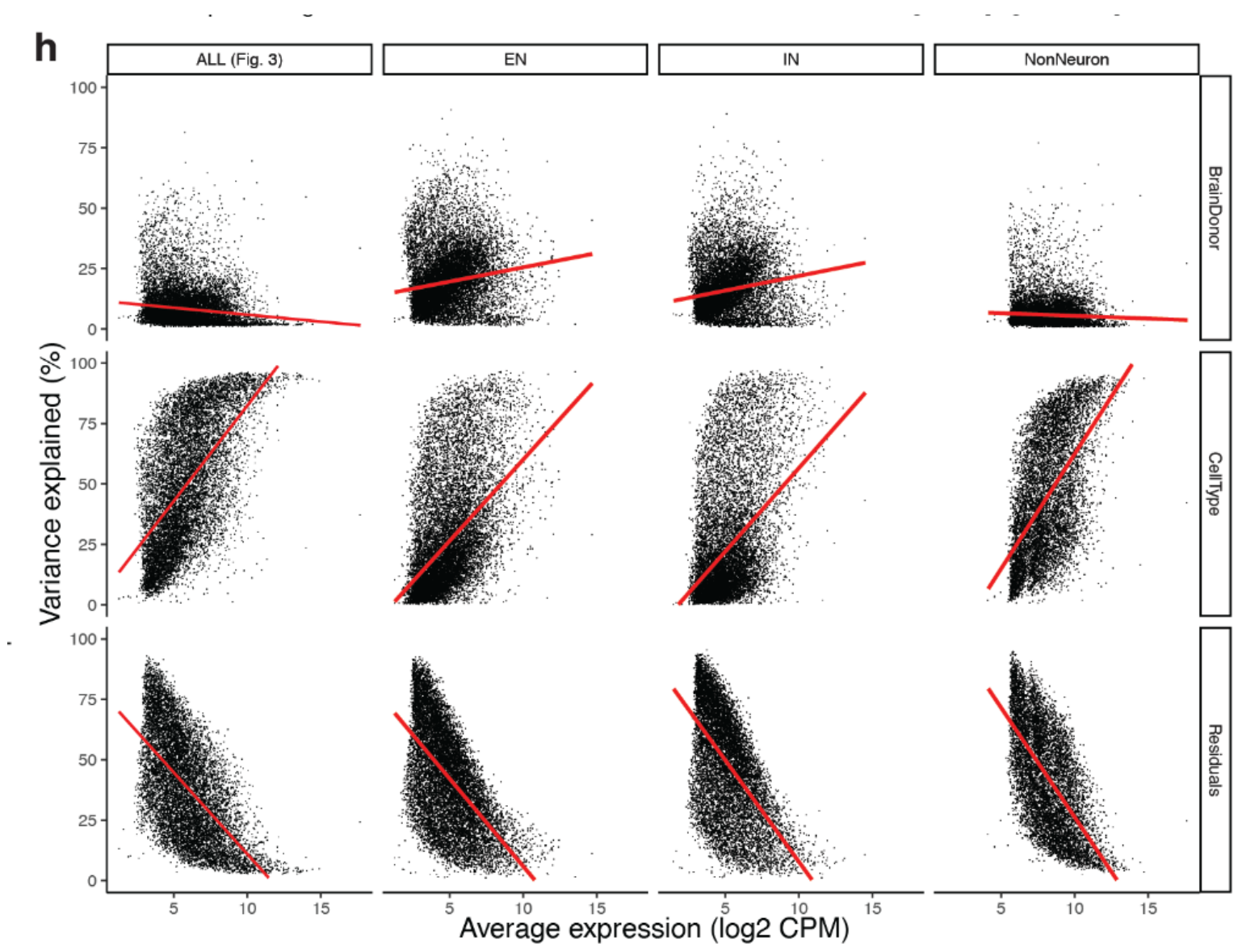

Supplementary Fig. 5. … **(h)** Relationship between the percentage of variation explained by mean expression (log2 counts per million). Rows are stratified by model coefficients; brain donors (BrainDonor) and cell types (CellTypes). Gene expression variance explained by these variables is computed for all subclasses (as shown in Fig. 3a) and three superclasses (as shown in Supplementary Fig. 6j), namely, Excitatory Neurons (EN), Inhibitory Neurons (IN), and Non-Neurons (NonNeuron). For example, variance of superclass "EN" is computed across all EN subclasses. The same is true for "IN". "NonNeuron" comprises all subclasses but EN and IN subclasses.

Variance partition analysis of gene expression

… We performed sensitivity analyses to ensure that performing variance partition analysis across all subclasses does not obscure the cell-type-specific signals. We performed variance partition analyses within more homogeneous cell populations by stacking cell type-specific assays into three superclasses, namely, EN, IN, and Non-Neuron, similar to previous approach by BICCN exploring inter-individual variation of human brain[31]. This setup enables better resolution of potentially meaningful covariates within a cell class while still adjusting for sample- and technical-level confounders. Overall, we observed highly concordant variance partition results between variance partition performed separately in each superclass and variance partition performed with all subclasses. The dominant source of variance remained the same as the CellType followed by the BrainDonor (**Supplementary Figs. 6j**). Spearman correlation of estimated variance between two models were high across all covariates including BrainDonor, Age, Sex, Ancestry, and Diagnosis (**Supplementary Figs. 6k**).

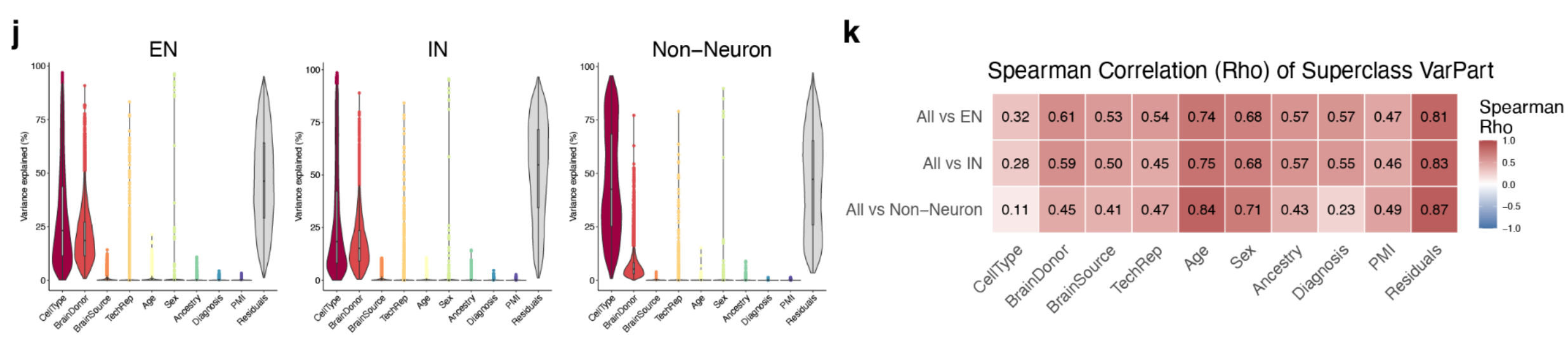

Supplementary Fig. 6. … **(j)** Variance partition analysis within three superclasses, namely, Excitatory Neurons (EN), Inhibitory Neurons (IN), and Non-Neurons (Non-Neuron). **(k)** Sensitivity analyses showing the robustness of variance explained estimates. Spearman correlation of variance explained estimates between all subclasses model (All) vs. EN, IN, and Non-Neuron superclass models. …

REF2-5. Compositional analysis	
(3) The compositional analysis in Figure 4 raises some questions. While Figure 4a suggests significant changes in cell type composition between patients with different disorders, Supplementary Figure 4b shows that only a small fraction of variance is explained by these disorders. Are the correlations in Figure 4a adjusted for compositional bias, or could they be showing artifactual anti-correlations? It might be clearer to either show correlation estimates using the composition estimated by Crumblr or remove the correlations to avoid confusion. Additionally, it would be helpful to include the raw data for some of the cell types highlighted in the compositional analysis in the supplementary materials e.g. boxplots showing the fraction of VLMC cells across samples from donors with NDDs and NPDs could provide insight into the magnitude of these changes. Finally, are these compositional changes expected based on what is known about these disorders? Have they been reported before, and if so, what mechanisms have been proposed?	
Response	We thank the reviewer for this feedback. Interpreting results relating to differences in cellular composition based on disease status is challenging due to the nature of compositional data. Single nucleus RNA-seq captures the relative, but not absolute, abundance of each cell type. Moreover, for a given sample the cell fractions must sum to 100% by definition. These limitations mean that, for example, an increase in the composition of one cell type will cause the observed composition of the remaining cell types to decrease. The centered log ratio (CLR) transform we used in the crumblr method[32] is one way to account for the compositional bias in these data, although we acknowledge that all compositional analyses have this issue to some degree. We then performed statistical testing on CLR transformed count ratio data using precision-weighted linear mixed models incorporating random effects for complex study designs. As a result, we describe changes in cell subclass frequency, but don't claim that they are causal. To summarize our approach, we estimated changes in cell type composition driven by disease for each brain source, after regressing out the effects of age and sex. For diseases represented by two brain sources, we performed fixed effect meta-analysis, as described in **Methods**.<br><br>With that said, we would like to clarify that the variance partition results are shown in relative scale (**Supplementary Fig. 7b**; formerly Supplementary Fig. 4b), and they do not accurately represent the effect sizes or significance of covariates. Furthermore, **Fig. 4a** suggests changes in cell type composition clusters based on category of disorders (by NDDs or NPDs). Overall, we observed a significant difference in cellular composition associated with disease status. However, it explains a small fraction of variance, indicating that they may not have been uncovered in a more modestly sized study.<br><br>To further demonstrate the robustness of our approach, we replicated the compositional variation analysis using two independent snRNA-seq datasets (**Supplementary Figs. 10a-b**), using AD as a disease contrast where there was a good match of cases and controls between cohorts. We also performed compositional variation analysis on the MERFISH dataset included in the recent SEA-AD publication [7] - using an orthogonal experimental method demonstrating that the compositional variation is observed independent of experimental approach (**Supplementary Fig. 10c**). These datasets had differing demographics and tissue handling protocols, further supporting the robustness of our findings across distinct sources and methodologies.<br><br>In response to the reviewer's suggestion, we added boxplots showing the fraction of VLMC cells across donors with NDDs and NPDs, providing insights into the magnitude of these changes (**Supplementary Figs. 7e-f**)

Excerpt

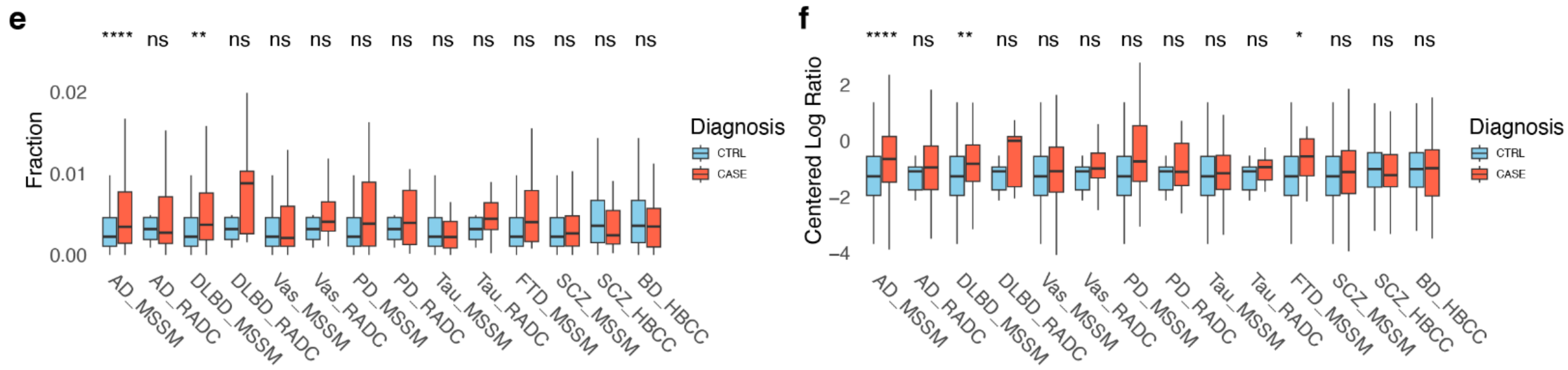

Supplementary Fig. 7. Cross-disorder variation of cell type composition comparing eight different NDDs and NPDs against common neurotypical controls. … **(e-f)** Compositional variation of VLMC subclass for eight different diseases and brain sources. T-test with p-value <= 0.05 *, <= 0.01 **, <= 0.001 ***, <= 0.0001 ****. Using **(e)** fraction or **(f)** centered log ratio (CLR).

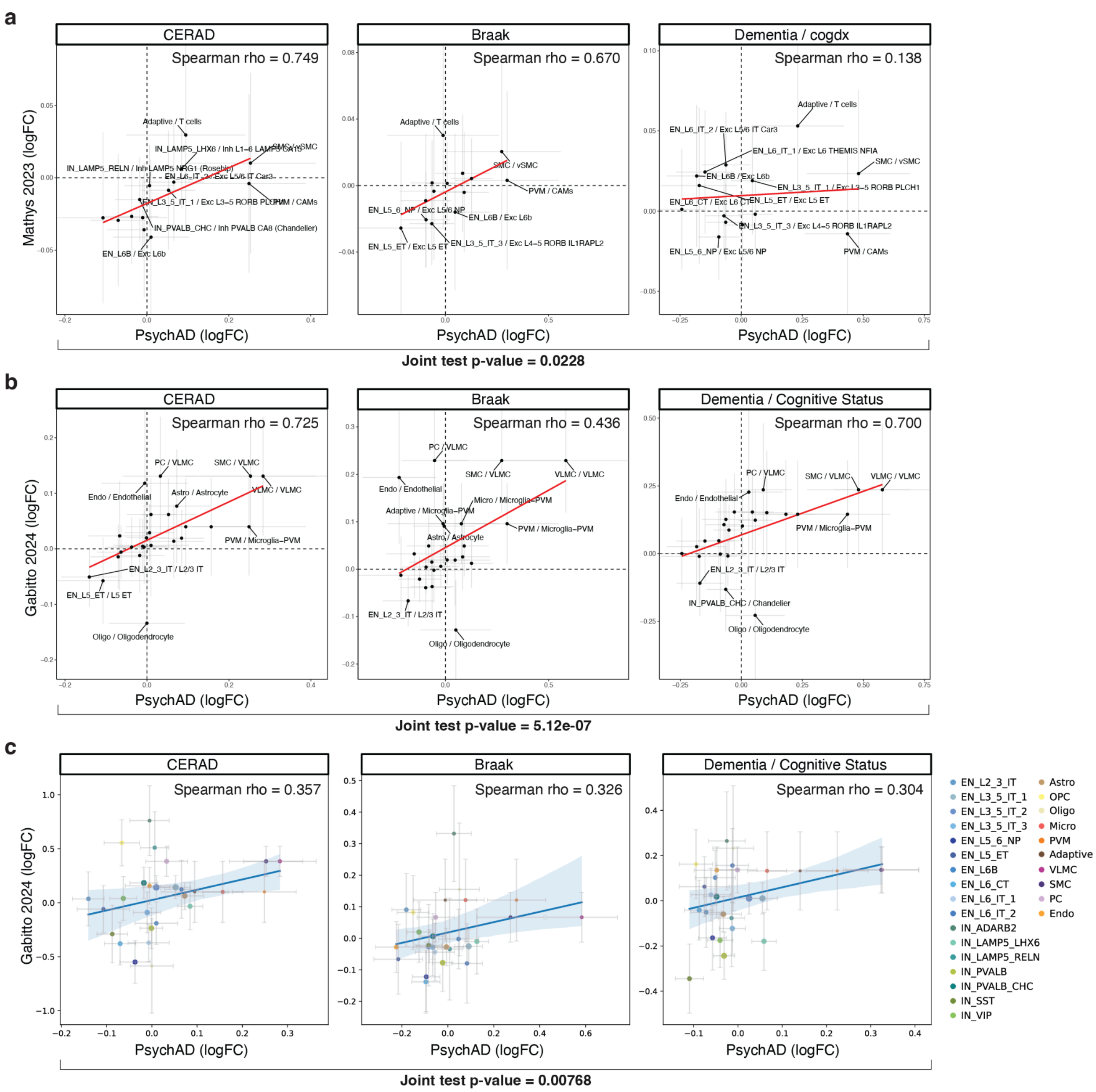

	**Supplementary Fig. 10**. Comparison of compositional variation analysis with previous studies - DLPFC snRNA-seq data from **(a)** ROSMAP (Mathys et al. 2023)[6] and **(b)** SEA-AD (Gabitto et al. 2024)[7]. **(c)** Comparison of compositional variation analysis using the MERFISH spatial transcriptomics dataset included in the SEA-AD study[7]. Spearman correlation between this study (x-axis) and previous studies (y-axis) using 3 different measures of AD pathology (CERAD score, Braak staging, and ordinal Dementia scale). ROSMAP used clinical diagnoses of AD, MCI, and NCI, while SEA-AD used clinical diagnosis of dementia as a measure of cognitive decline. Further details regarding compositional analysis of the MERFISH data and joint statistical testing can be found in the **Methods** section.

REF2-6. Mediation analysis	
(4) The robustness of the mediation analysis (figure 6) and its conclusions are challenging to assess. How sensitive is the model to changes in the included cell types? Would the conclusions hold if polygenic risk scores (PRS) for a different disease were used? Could the authors also clarify how this model aligns with other analyses in the paper? The mediation analysis indicates that polygenic risk influences plaque and tau progression, which then impacts microglia abundance. However, the scDRS analysis in Figure 2f shows that microglial cells over-express genes linked to AD-implicated variants. Doesn't this suggest that polygenic risk might be mediated by microglial cells?	
Response	We thank the reviewer for their insightful comments regarding the mediation analysis. We have incorporated their suggestions and present the following revisions and clarifications.<br><br>1. We performed additional sensitivity analyses to address the robustness of our mediation findings. First, given that the APOE ε4 allele is a strong, well-established AD risk factor, we tested its specific impact on the mediation analysis. We recalculated the PRS after excluding the APOE locus and repeated the mediation analysis (**Supplementary Table 13**). The differences were negligible, demonstrating that the PRS and mediation results are robust to the influence of APOE. Second, to ensure the AD-specificity of our observations, we performed the causal mediation analysis using PRSs derived from other neurological diseases (PD, MS, ALS, SCZ, BD, and ASD). None of these alternative PRSs yielded significant mediation effects (**Supplementary Table 13**), supporting the specificity of our findings to AD.<br><br>2. We acknowledge the reviewer's point about the strong scDRS scores for Microglia and PVM and their expected role in mediating polygenic risk. While Microglia did show evidence of mediating effects, it was not statistically significant ($P > 0.05$). The direct effect of PRS on AD pathology (Plaque, Braak, or dementia) was considerably stronger than the indirect effect mediated through changes in Microglia proportions. It is crucial to note that our analysis focused on the mediation effect through changes in cell type composition. This does not preclude other mechanisms by which Microglia might mediate AD risk (e.g. changes in gene expression or activation state within the microglia population), which were not directly assessed in this mediation analysis.
Excerpt	"...

Treatment (IV)	Mediator (MV)	Outcome (DV)	Effects	Estimate	95% CI Lower	95% CI Upper	p-value	Significance	Note
PRS_AD	PLAQUE	Braak	ACME	0.0878	0.05	0.13	<2e-16	***	AD_2022_Bellenguez
PRS_AD	PLAQUE	Braak	ADE	0.0735	0.0296	0.12	0.0014	**	AD_2022_Bellenguez
PRS_AD	PLAQUE	Braak	Total Effect	0.1613	0.1045	0.22	<2e-16	***	AD_2022_Bellenguez
PRS_AD	PLAQUE	Braak	Prop. Mediated	0.5444	0.3633	0.76	<2e-16	***	AD_2022_Bellenguez
PRS_AD_wo_APOE	PLAQUE	Braak	ACME	0.1342	0.0866	0.18	<2e-16	***	AD_2022_Bellenguez without APOE locus
PRS_AD_wo_APOE	PLAQUE	Braak	ADE	0.0907	0.0334	0.15	0.0018	**	AD_2022_Bellenguez without APOE locus
PRS_AD_wo_APOE	PLAQUE	Braak	Total Effect	0.2249	0.1525	0.3	<2e-16	***	AD_2022_Bellenguez without APOE locus
PRS_AD_wo_APOE	PLAQUE	Braak	Prop. Mediated	0.5958	0.4276	0.81	<2e-16	***	AD_2022_Bellenguez without APOE locus
PRS_PD	PLAQUE	Braak	ACME	-0.0418	-0.0897	0.01	0.085	.	PD_2019_Nalls
PRS_PD	PLAQUE	Braak	ADE	0.014	-0.0397	0.07	0.612		PD_2019_Nalls
PRS_PD	PLAQUE	Braak	Total Effect	-0.0278	-0.099	0.04	0.45		PD_2019_Nalls
PRS_PD	PLAQUE	Braak	Prop. Mediated	0.9626	-8.6891	10.14	0.399		PD_2019_Nalls
PRS_ALS	PLAQUE	Braak	ACME	0.021	-0.0198	0.06	0.3		ALS_2021_vanRheenen
PRS_ALS	PLAQUE	Braak	ADE	0.0284	-0.0173	0.07	0.22		ALS_2021_vanRheenen
PRS_ALS	PLAQUE	Braak	Total Effect	0.0495	-0.0105	0.11	0.11		ALS_2021_vanRheenen
PRS_ALS	PLAQUE	Braak	Prop. Mediated	0.4228	-1.6198	2.22	0.28		ALS_2021_vanRheenen
PRS_MS	PLAQUE	Braak	ACME	0.00877	-0.031	0.05	0.68		MS_2019_IMSGC
PRS_MS	PLAQUE	Braak	ADE	-0.02275	-0.06859	0.02	0.33		MS_2019_IMSGC
PRS_MS	PLAQUE	Braak	Total Effect	-0.01399	-0.07371	0.05	0.65		MS_2019_IMSGC
PRS_MS	PLAQUE	Braak	Prop. Mediated	0.21086	-9.73452	8.34	0.78		MS_2019_IMSGC
PRS_SCZ	PLAQUE	Braak	ACME	-0.00548	-0.04663	0.04	0.79		SCZ_2022_Trubetskoy
PRS_SCZ	PLAQUE	Braak	ADE	0.01654	-0.03092	0.06	0.49		SCZ_2022_Trubetskoy
PRS_SCZ	PLAQUE	Braak	Total Effect	0.01106	-0.05093	0.07	0.73		SCZ_2022_Trubetskoy
PRS_SCZ	PLAQUE	Braak	Prop. Mediated	0.31545	-6.80958	7.49	0.66		SCZ_2022_Trubetskoy
PRS_BD	PLAQUE	Braak	ACME	-0.00184	-0.04155	0.04	0.92		BD_2021_Mullins
PRS_BD	PLAQUE	Braak	ADE	0.00309	-0.04126	0.05	0.88		BD_2021_Mullins
PRS_BD	PLAQUE	Braak	Total Effect	0.00124	-0.05802	0.06	0.97		BD_2021_Mullins
PRS_BD	PLAQUE	Braak	Prop. Mediated	0.44031	-5.62087	7.29	0.55		BD_2021_Mullins
PRS_ASD	PLAQUE	Braak	ACME	0.00407	-0.0387	0.05	0.84		ASD_2019_Grove
PRS_ASD	PLAQUE	Braak	ADE	0.02864	-0.0184	0.08	0.23		ASD_2019_Grove
PRS_ASD	PLAQUE	Braak	Total Effect	0.03271	-0.02994	0.1	0.31		ASD_2019_Grove
PRS_ASD	PLAQUE	Braak	Prop. Mediated	0.25062	-4.19067	5.1	0.62		ASD_2019_Grove

Supplementary Table 13. Causal mediation analysis involving PRS, cell type composition, pathology, and dementia status. Estimates of direct and mediating effects and their statistical significance.

…"

REF2-7. Nonlinear dynamics of AD pathology

(5) Regarding the section on nonlinear dynamics of AD pathology, it's unclear what the proposed VAE-based approach brings to the table. The motivation to employ this modelling strategy is abstractly described in the method section, but there is no data showing that this approach identifies different genes than what you would get from linear regression or common trajectory inference methods. Additionally, it's not entirely clear why the normalized ordinal labels from Braak staging couldn't be used to identify clusters of genes with similar dynamic patterns. If the primary concern is de-correlating tau and dementia, a multi-factorial linear model with an interaction term might be a more straightforward and less error-prone approach. Without calibration and sanity check analyses, it's difficult to assess whether the identified patterns are genuine or the result of over-fitting and over-smoothing.

Response

We fully agree with the reviewer that we did not clearly justify our use of neural network models to perform trajectory inference and omitted several important controls. Below, we address why we chose not to use 1) existing trajectory inference methods, 2) linear regression models, or 3) a multi-factorial approach. The decision to adopt a neural network approach was partly motivated to disentangle changes in gene expression associated with disease burden from those associated with dementia, allowing us to calculate a dementia resilience score; we've therefore added 4) new visualization to better illustrate how certain pathways are protective against dementia, whereas others are damaging. We also present new controls regarding 5) possible over-smoothing, and present 6) new results on the rich gene expression dynamics underlying the immune response during disease progression.

1. Comparison with existing trajectory inference methods: We agree with the reviewer that we did not adequately describe why we chose to perform trajectory inference using our neural network models over existing trajectory inference methods. While existing methods such as Monocle3, Palantir, etc.,

	have been used to successfully describe developmental trajectories, we felt they were less well suited to modelling disease trajectories.<br><br>We have added a section in the **Methods** detailing our rationale for not using traditional trajectory inference methods (see **Excerpt 1a**). We compare our method to two traditional approaches, Monocle3 and Palantir, highlighting a greater correlation between the true Braak values and our network model predictions (added as **Supplementary Fig. 27** and shown below as **Excerpt 1b).**<br><br>**2. Comparison with linear regression model**: As pointed out by the reviewer, we did not state whether our approach identifies different genes/pathways than linear regression. We now perform this comparison for the Immune cell class, describing our approach in the new Methods section **Comparison to partial least squares (PLS) regression**, with the results presented in **Supplementary Fig. 15** (shown below as **Excerpt 2a**).<br><br>**Supplementary Fig. 15** demonstrates that while linear models achieve reasonable accuracy for Braak stage predictions (and less so for dementia), their predictions for Braak and dementia are highly correlated. Disentangling these effects is crucial, as models that fail to do so may overlook genes and pathways with opposing expression patterns. Notably, for pathways exhibiting such opposing trends—including key immune response pathways such as macrophage activation, antigen presentation, and T-helper I immune response —the PLS model suggests a significantly weaker response.<br><br>We've also added a section in the Main text (and shown below as **Excerpt 2b**) emphasizing the importance of decorrelating Braak and dementia model predictions.<br><br>Methods might exist to 1) improve the PLS dementia classification accuracy while at the same time 2) decorrelate Braak and dementia model predictions. Thus, we are not claiming that linear models are inherently incapable of modelling gene expression dynamics. However, it is not obvious to us how to improve PLS model predictions on these two points such that they match our network model predictions.<br><br>**3. Possible use of a multi-factorial linear model**: We believe that a linear multi-factorial model with an interaction (with the form $gene\ expression = \alpha \times Braak + \beta \times Dementia + \gamma \times Braak \times Dementia$) would only work under a narrow set of conditions. First, it assumes that gene expression varies linearly with Braak, which we argue here is not always the case. Second, if a gene is associated with cognitive resilience (or decline) in only the early or later stages of the disease, then model accuracy would suffer, and interpreting the model coefficients would be challenging. Given these limitations, we felt that training neural networks to disentangle Braak and dementia, for which there are established methods (Idrissi et al, 2021), would be a more effective way to isolate the effects of disease progression and dementia on gene expression.<br><br>**4. Visualizing protective and damaging pathways**: The decision to adopt a neural network approach was partly motivated by the need to disentangle gene expression changes associated with overall disease burden from those specifically linked to dementia symptoms. This distinction enabled us to calculate a dementia resilience score that captures how individual expression profiles deviate from expected cognitive outcomes given disease severity. We have added new visualizations to better illustrate how certain pathways may be protective against dementia, while others contribute to cognitive decline (**Supplementary Fig. 21** and **Excerpt 4a**). In this new figure, we show the pathway enrichment (Zenith z-score) as a function of disease pseudotime (predicted Braak stage, x-axis) and predicted dementia (y-axis). For damaging pathways, such as the synaptic pathways of inhibitory neurons, we observe that, at a fixed disease pseudotime, pathway enrichment tends to increase with higher predicted dementia (**Supplementary Fig. 21b**). For immune pathways, which are protective, it is the opposite: at a fixed disease pseudotime, pathway enrichment tends to decrease with higher predicted dementia (**Supplementary Fig. 21d**).

	**5. Concern about overfitting and over-smoothing**: Lastly, we agree with the reviewer that one should always be concerned if the results might be affected by any form of overfitting or over-smoothing. Regarding the former, all analysis was performed using cross-validated model predictions, in which donors in the training set were distinct from donors in the test set. While mentioned in the methods, we did not state this in the main text. We now explicitly state this in the main text when describing Figure 7 (**Excerpt 5a**).<br><br>We admit we did not consider how potential over-smoothing might affect our results. Thus, we now compare the changes in gene expression during the early and late disease states, and the early and late resilience scores, between smoothed and unsmoothed gene trajectories. We describe this comparison in the new **Methods** section **Pathway enrichment without smoothing**, and the results are shown **Supplementary Fig. 28** (shown below as **Excerpt 5b**). The results demonstrate that pathway enrichment scores using unsmoothed trajectories are highly similar to those obtained using smoothed trajectories.<br><br>**6. Further exploring the complex gene expression dynamics**: Lastly, we attempted to expand our analysis to better demonstrate the rich, nonlinear dynamics of gene expression. We've added **Supplementary Fig. 25** (shown below as **Excerpt 6b**) which shows the change in gene expression for key pathways using a sliding window across the disease pseudotime axis. We present results for pathways that significantly changed during disease progression, and either (1) significantly enriched for Alzheimer's disease (AD) risk genes, or (2) were among the key immune and metabolic pathways highlighted previously in **Fig. 7e** (details provided in **Methods**). This analysis suggests two distinct phases of the immune response: an initial innate response (e.g. macrophage activation, TLR2, etc.), followed by a more adaptive/monocyte-based response. This second stage is characterized by an increase in cytokine production, and further upregulation of several key pathways involved in lipid homeostasis and efflux. These results highlight the nonlinear dynamics of the immune response, underlying the importance of examining how key pathways shift at different stages of the disease.
Excerpt	**1a.**<br>**Methods**, rationale for not using existing trajectory inference methods: "Most trajectory inference tools rely on identifying continuous transitions within the dominant sources of variation in the data. However, as described in **Fig. 3a**, the variance in gene expression attributable to disease state is dwarfed by variance introduced by inter-donor heterogeneity, sex, brain source, and other technical or biological confounders. When disease related variance is not the principal axis of variation, these tools may construct trajectories that primarily reflect confounding factors rather than true disease progression. To address this limitation, we trained neural network models in a supervised manner that directly estimates the state of disease progression from single-cell gene expression data. Unlike unsupervised trajectory inference methods, our approach explicitly models the relationship between gene expression and disease labels (e.g. Braak stage and dementia status), allowing us to assign each cell a continuous disease progression score. This enables more accurate isolation and sensitive detection of disease-related signals, even when they account for a relatively small fraction of the overall variance.<br><br>**1b.**

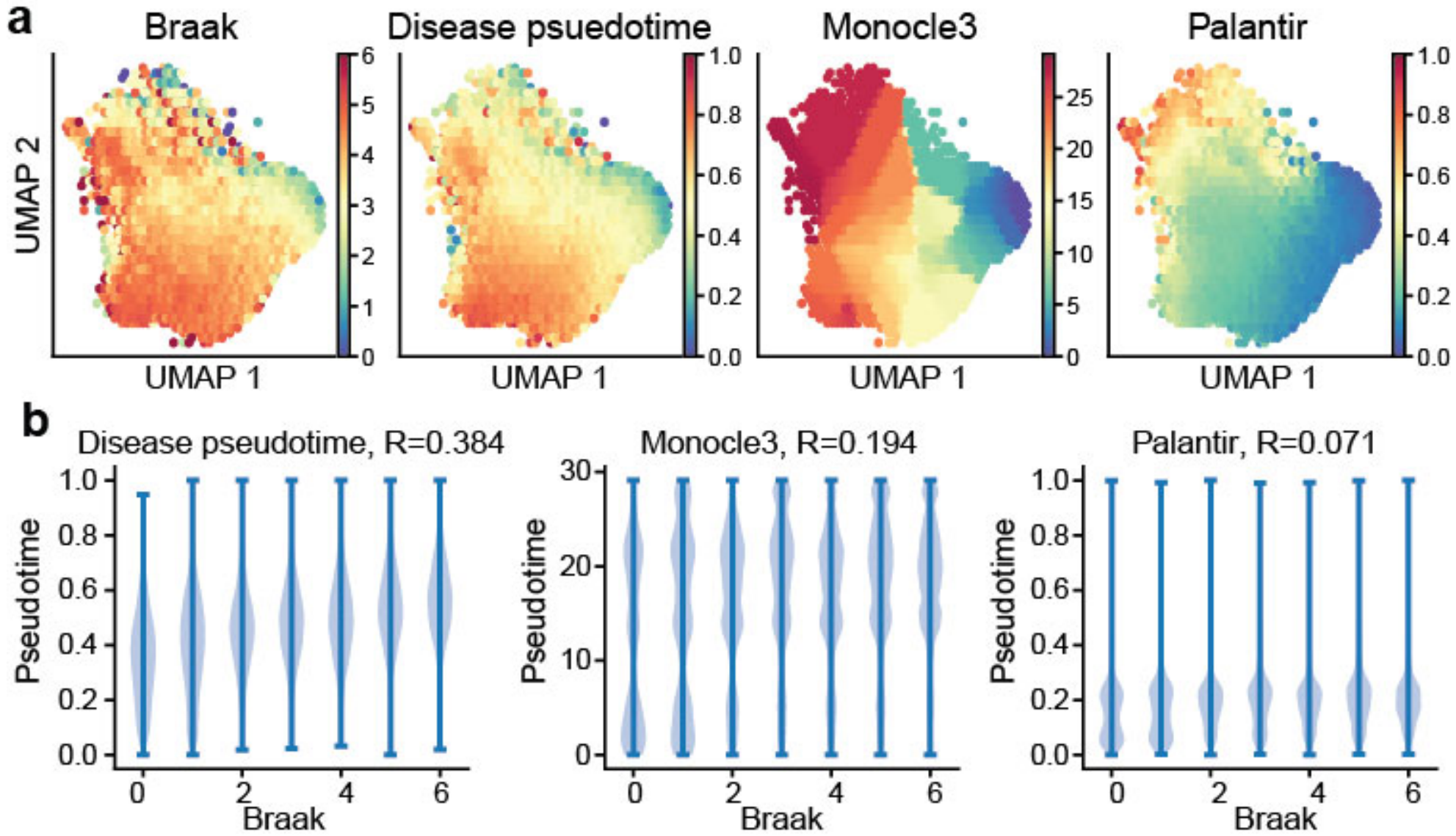

Supplementary Fig. 27 Comparing model predictions to existing single cell trajectory inference methods. **(a)** UMAP projections of the microglia cell subtype with the color at each location indicating the mean (from left to right) actual Braak values, the disease pseudotime neural network model predictions, Monocle3 and Palantir pseudotime values. **(b)** Distribution of (from left to right) the disease pseudotime neural network model predictions, Monocle3 pseudotime values and Palantir pseudotime values. The Pearson correlation coefficient is shown for each method.

2a.

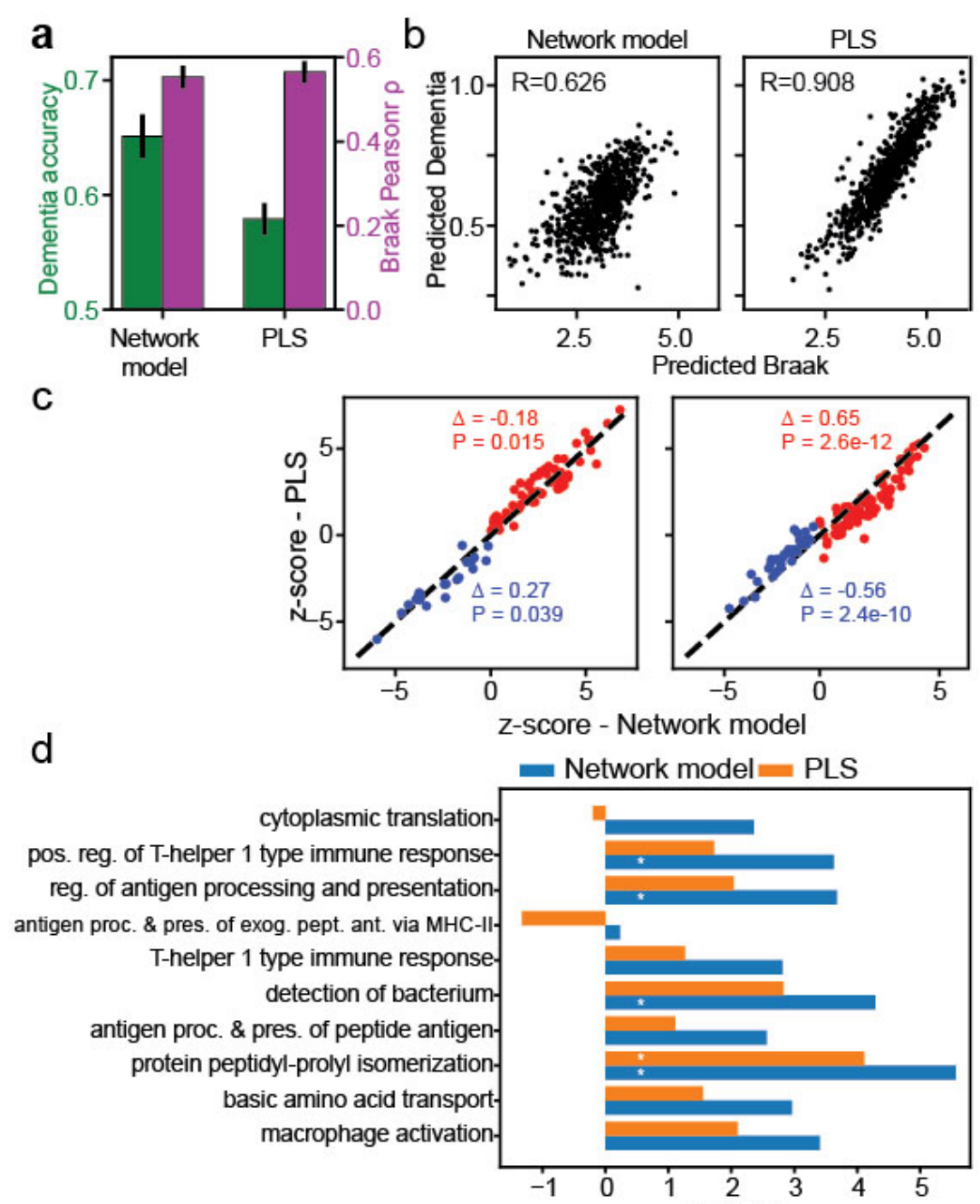

Supplementary Fig. 15 Comparing model predictions to PLS regression. **(a)** Dementia classification accuracy (green) and Pearson correlation between actual and predicted Braak values (magenta) for our network model and PLS regression. Results for the Immune cell class shown. **(b)** Donor-averaged Braak predictions (x-axis) and dementia predictions (y-axis) for our network model (left) and PLS regression (right). The Pearson correlation is shown for both models. **(c)** Pathway enrichment Zenith z-score for our network model (x-axis) and PLS (y-axis). The left panel shows pathways with similar trends between Braak and dementia (i.e. expression increases with Braak and is associated with greater dementia or decreases with Braak and is associated with less dementia). The right panel shows pathways with diverging trends (i.e. expression increases with Braak and is associated with less dementia or decreases with Braak and is associated with greater dementia). Red (blue) dots indicate whether the z-

score was positive (negative). The difference and Wilcoxon p-value comparing the network model and PLS z-scores is shown. Results are shown for changes in the early disease state. All significant pathways (FDR < 0.05 for either model, either for changes in disease (i.e. Braak) or resilience), are included. **(d)** The top ten pathways with the greatest difference between the z-scores from our network model (blue) and the PLS model (orange). White asterisks indicate whether the pathway was significant (FDR < 0.05).

2b.

Main text, describing Figure 7: Because tau proteinopathy and dementia are correlated, models that do not disentangle their effects may miss genes with opposing expression patterns. For instance, if a gene's expression rises with tau but falls with dementia, these effects could cancel one another out, obscuring significant changes."

4a.

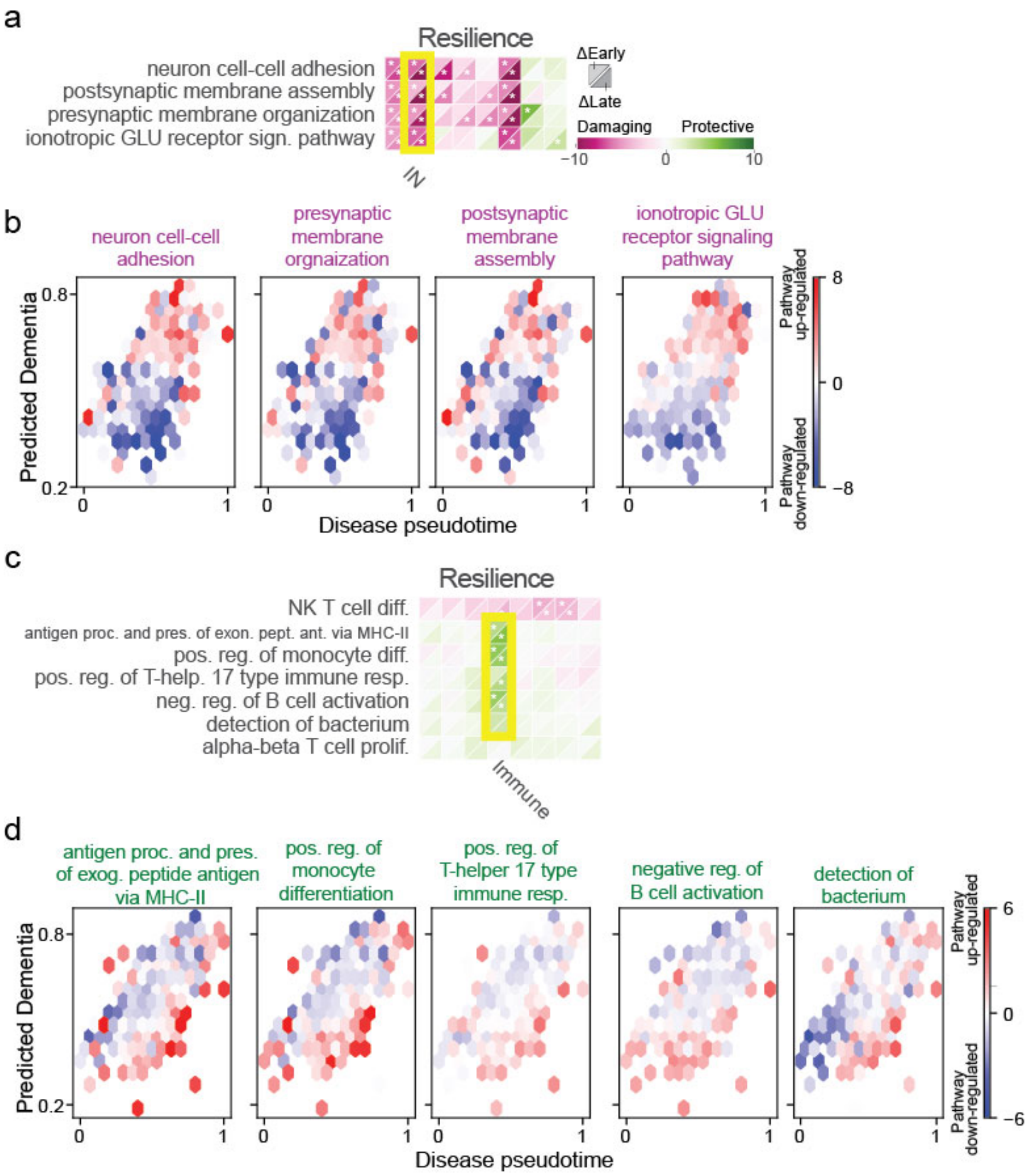

Supplementary Fig. 21. Visualization of protective and damaging pathways. (a) Extracted from Fig 7e., highlighting damaging synaptic pathways associated with the IN cell class. **(b)** Hexbin plots showing the mean pathway enrichment scores of four different synaptic pathways as a function of predicted Braak stage (x-axis) and predicted dementia status (y-axis). The color of each hexagon indicates the mean pathway enrichment from donors with similar disease pseudotime and predicted dementia scores. Enrichment of four synaptic pathways as a function of the disease pseudotime (x-axis) and the predicted Dementia score (y-axis). The color at each location indicates the mean pathway enrichment (Zenith z-score). **(c)** Same as (a), except highlighting protective pathways of the Immune cell class. **(d)** Same as (b), except for the protective immune pathways.

5a.
Main text, describing Figure 7: "... with predictions generated on cross-validated data, using non-overlapping donors for training and testing."

5b.

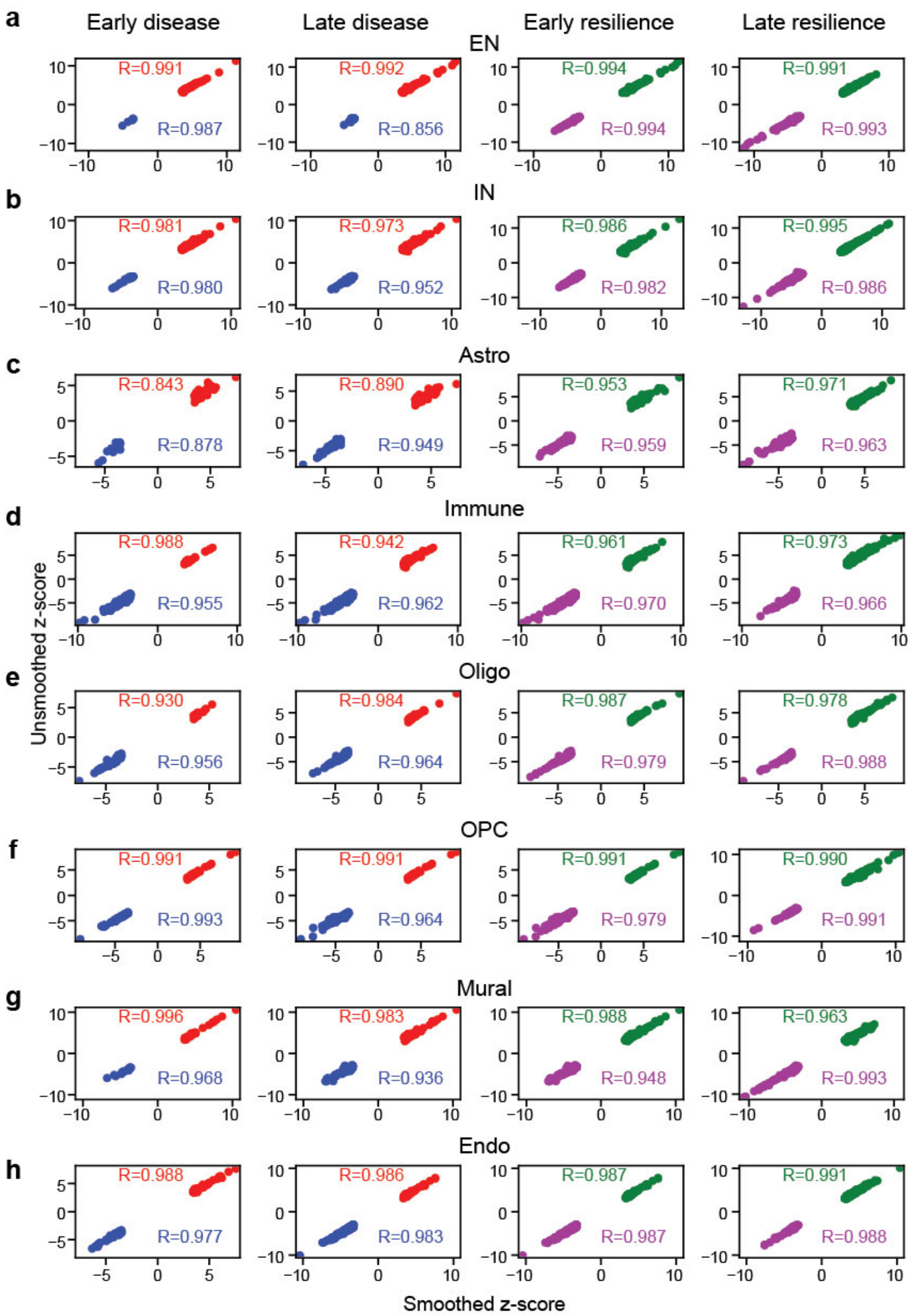

Supplementary Fig. 28 Comparing smoothed and unsmoothed approaches to measuring changes in gene expression and resilience. Each dot represents a GO BP pathway, in which the x-axis indicates the Zenith z-score using the smoothed approach, and the y-axis indicates the z-score using the unsmoothed approach (see Methods). Only pathways for which the Zenith FDR calculated using the smoothed approach was below 0.05 were included. The columns, from left-to-right, show the early and late changes in gene expression with disease pseudotime, and the early and late resilience scores. For disease pseudotime, red circles indicate increasing pathways, and blue circles indicate decreasing pathways. For resilience, green circles indicate protective pathways, and magenta circles indicate damaging pathways. The Pearson correlation (R), measured separately for increasing and decreasing, or protective and damaging, pathways is shown in each panel. Each row shows the results for one cell class.

6a.
Main text, describing Fig. 7: "To further resolve the immune response at a finer time resolution, we performed pathway enrichment using a sliding window across disease pseudotime (**Supplementary Fig. 25).** Our results suggest two distinct immune phases[33]: an initial innate response, followed by a more adaptive and monocyte-driven response, along with increased cytokine production. Lipid homeostasis and efflux pathways, many involving known AD risk genes, increased throughout disease progression, particularly in later stages (**Supplementary Figs. 25&26b)**, supporting the hypothesis that microglia develop a lipid-droplet accumulation phenotype that may exacerbate disease progression[34–36]."

6b.

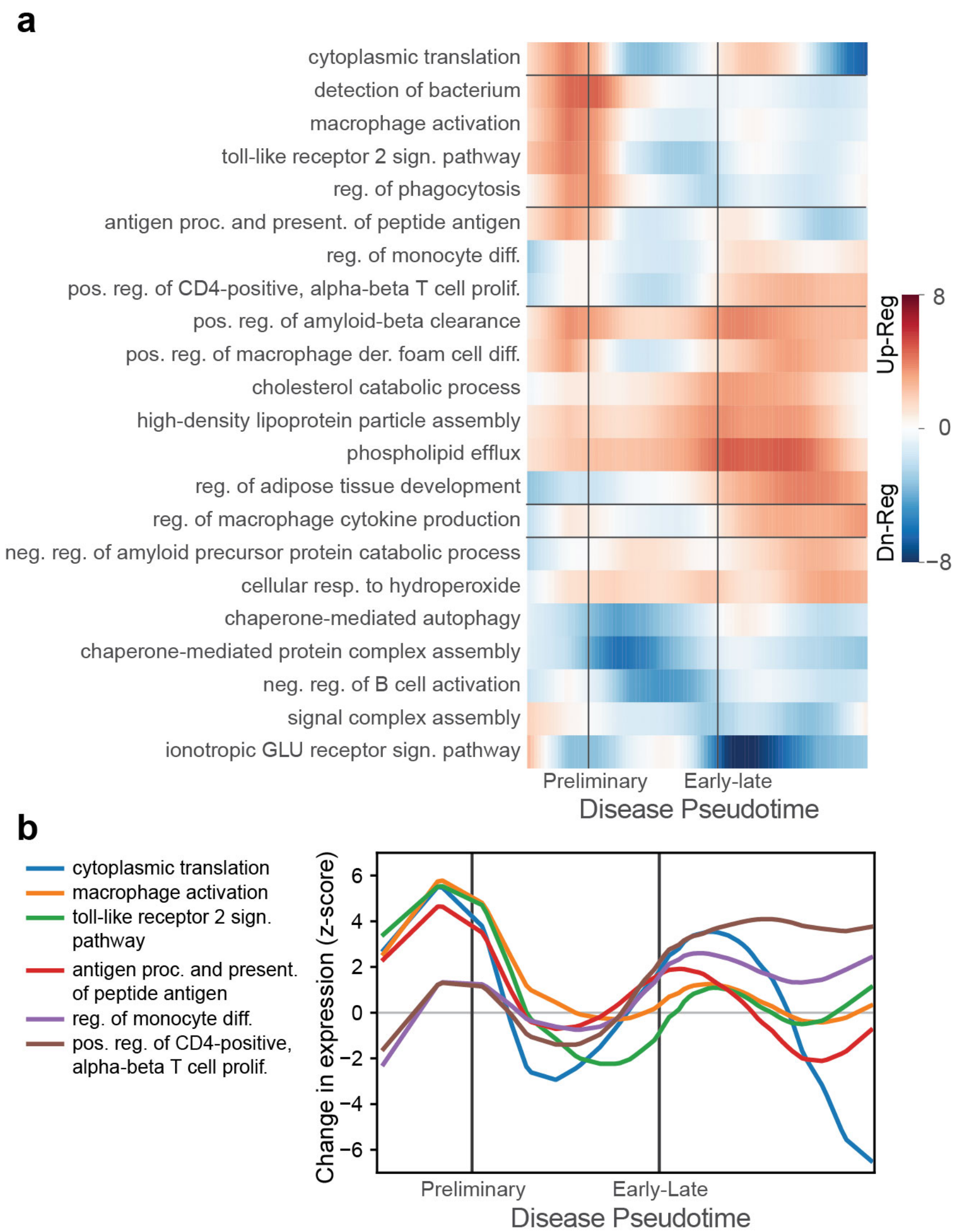

Supplementary Fig. 25. Dynamics of the immune response using pathways enriched in risk genes. **(a)** Change in pathway gene expression calculated using a sliding window across disease pseudotime. Hue indicates Zenith z-score. Vertical lines (indicated by preliminary and early-late) are the transition points identified in Fig. 7c. **(b)** Similar to (a), but only showing a subset of the pathways that significantly changed during disease progression, and are either (1) significantly enriched for Alzheimer's disease (AD) risk genes, or (2) among the key immune and metabolic pathways highlighted previously in Fig. 7e (details provided in **Methods**). The biphasic response suggests two distinct phases of the immune response.

REF2-8. Shared heritability

(6) In figure 5f the authors report that disease pairs with similar differential expression effects across cell types also have a higher shared heritability. To ensure that this association is robust, the authors should adjust the correlation analysis in figure 5f to propagate uncertainties from the shared heritability and transcriptome similarity (which seem rather large from fig. 5d-e). Having validated this association, is this expected? Is this reported or investigated in similar studies comparing genomics and transcriptomics data (e.g. PsychENCODE https://www.nature.com/articles/nn.4156)?

Response	We appreciate your valuable feedback. We addressed the reviewer's concern regarding **Fig. 5f** by revising the correlation analysis to include uncertainties from both shared heritability and transcriptome similarity using a bootstrapping approach, which is now presented in the figure with confidence intervals.<br><br>To further validate the association, we conducted a bootstrap analysis that consistently reproduced the observed results, demonstrating robustness. The association between estimated transcriptome similarity and shared heritability values was estimated using an approach to model the uncertainty in these estimates. Synthetic values were drawn from normal distribution centered at each estimate with the corresponding standard error. The Spearman correlation between the sampled transcriptome similarity and shared heritability was computed for each of 10,000 synthetic datasets (**Supplementary Fig. 8f**). Using a normal approximation of the rho statistic, we can reject the null hypothesis that rho is less than zero with p-value 1.27e-4.<br><br>Additionally, we included reference to relevant literature such as the PsychENCODE project and elaborated on the biological plausibility of the association, clarifying how similar differential expression patterns across cell types can reflect shared underlying genetic factors. We believe these revisions significantly strengthen the analysis and provide a more robust validation of the reported association.

Excerpt

"... By correlating the shared heritability against the average transcriptome similarity across cell types, we obtained a heritability-transcriptome concordance (r_g-ρ_s), which indicated a strong positive correlation (Spearman's ρ=0.604) between genetic and transcriptomic similarities (**Fig. 5f, Supplementary Fig. 8f**). The results show that diseases with higher genetic overlap also tend to have more similar transcriptomic dysregulation, suggesting that shared transcriptomic vulnerabilities are coupled with underlying genetic architecture, an unsurprising result that corroborates previous observations[19–21]. ..."

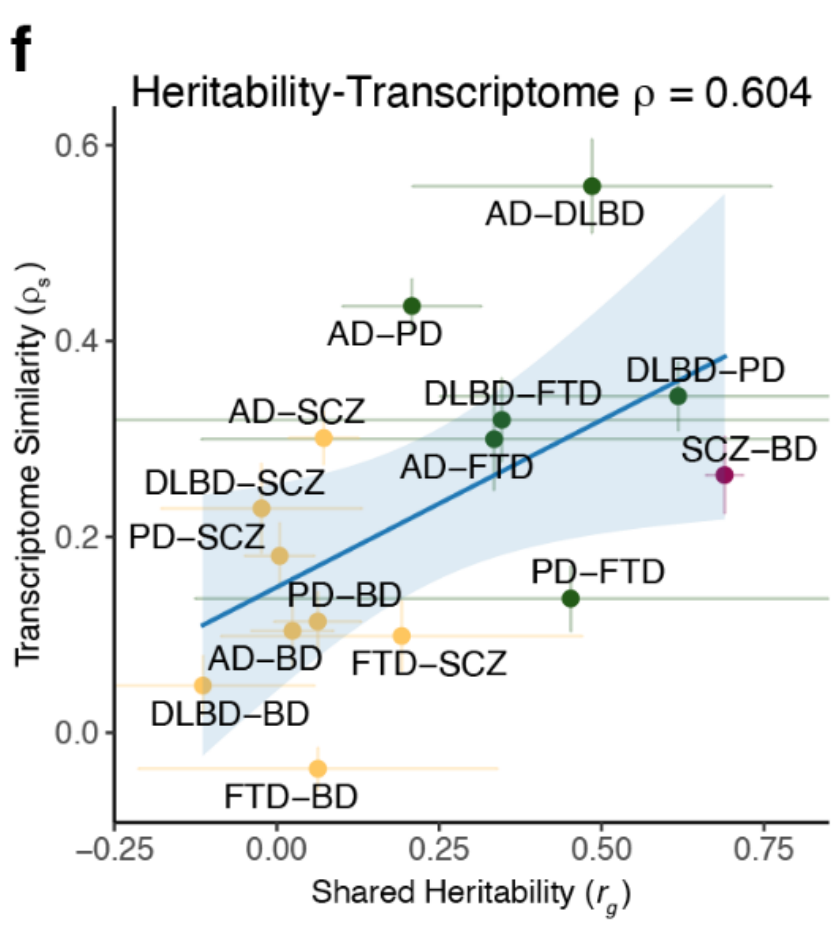

Fig. 5. **Cross-disorder variation of gene expression.** … **(f)** Correlation between shared heritability (r_g) and mean transcriptome similarity (ρ_s). Spearman's correlation coefficient. Error bars indicate 95% confidence intervals.

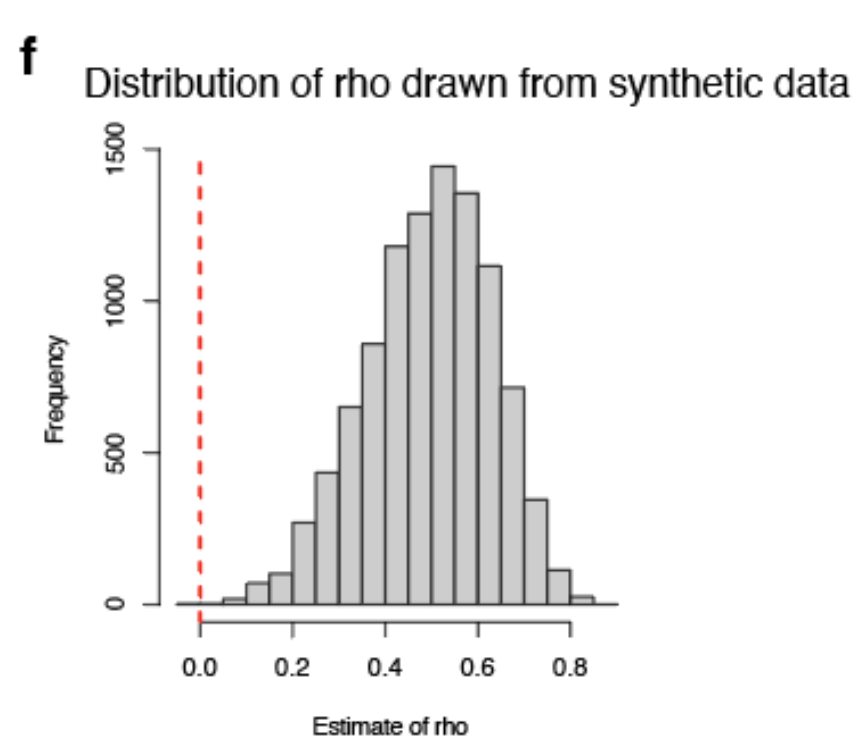

Supplementary Fig. 8. Cross-disorder gene expression signature. … **(f)** Distribution of the Spearman correlation coefficients from 10,000 synthetic datasets. Synthetic values were drawn from normal distribution centered at each

	estimate with the corresponding standard error. The Spearman correlation between the sampled transcriptome similarity and shared heritability was computed for each of 10,000 synthetic datasets. Using a normal approximation of the rho statistic, we can reject the null hypothesis that rho is less than zero with p-value 1.27e-4.

REF2-9. Omnigenic model, core genes, and burden tests

(7) Throughout the text, the authors reference the omnigenic model of inheritance to interpret their findings, but this connection feels somewhat speculative without further supporting analysis. The authors suggest that DE genes shared between diseases may capture common effects on peripheral genes (i.e., those closer to disease-relevant genetic variants) and propose that 'discounting cross-disease effects could facilitate the identification of core disease-relevant functional genes.' While plausible, this hypothesis isn't further tested in the current study. A counter-argument could be that any gene found to be significantly differentially expressed when the disease is already manifest should be considered several steps downstream of the causal genetic variants. To test whether disease-specific genes can be considered "core" rather than broadly upregulated genes, I recommend using data from burden tests (e.g., https://app.genebass.org/), where the signal for rare coding variants affecting the same genes is aggregated. Genes with a high burden of rare variants tend to be more enriched in functional annotations and are often considered better proxies for 'core' genes (see https://www.sciencedirect.com/science/article/pii/S0092867417306293 and https://www.nature.com/articles/s41586-022-05684-z).

Response

We would like to thank the reviewer for this excellent suggestion. In the revised manuscript, we performed additional analyses to confirm our hypothesis that discounting the cross-disease effects could facilitate the identification of core disease-relevant functional genes. In summary, we demonstrate that genes with disease-specific effects have a higher burden of rare variants than genes with cross-disease shared effects (**Supplementary Fig. 8e**). This observation further supports the proposed omnigenic model of complex traits[37] and aligns with previous findings that disease-relevant core genes tend to be associated with rare loss-of-function mutations[38].

Excerpt

"...

Cross-disorder variation of gene expression and genetic concordance

… Genes with disease-specific effects exhibited a higher burden of rare variants compared to those with shared effects (**Supplementary Fig. 8e**), suggesting that disease-specific components more precisely reflect core disease biology[38]. To facilitate the identification of core disease-relevant functional genes, we discounted genes with shared effects from the overall DEG expression profiles. …"

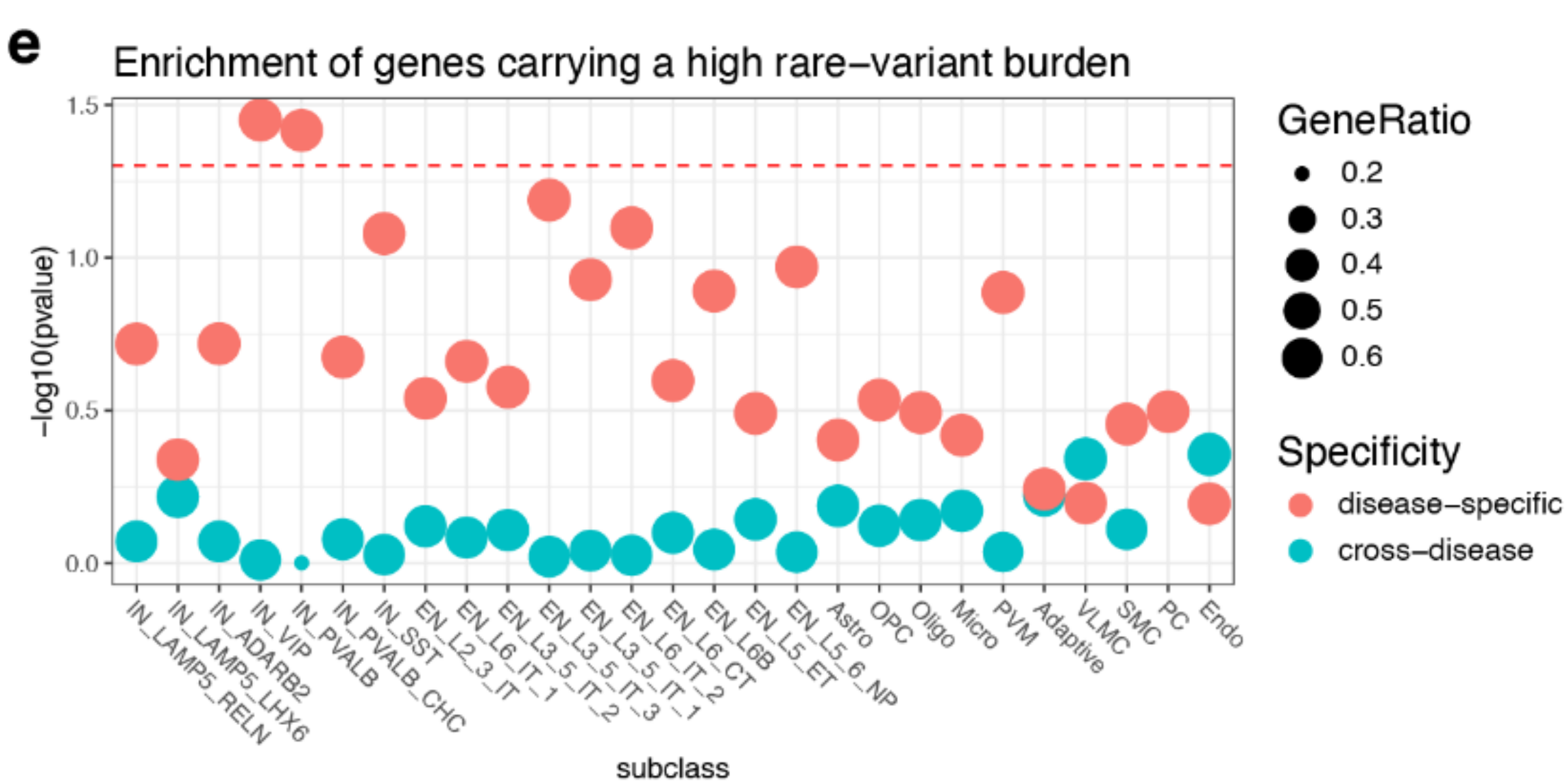

Supplementary Fig. 8. … **(e)** Enrichment of genes carrying a high rare-variant burden. Hypergeometric tests were performed on genes with cross-disorder shared effects and genes with disease-specific effects for each subclass. Rare-variant association results were downloaded from Genebase[39] (https://app.genebass.org/). For gene-based

	burden testing, we utilized the results from the Sequence Kernel Association Test - Optimal (SKAT-O), which utilized both mean and variance of the variant effects. Only predicted loss-of-function (LoF) variants for rare-variant association testing were considered. We defined a high rare-variant burden as genes with genome-wide significance (SKAT-O p-value < 2.5 x 10-7). GeneRatio is the fraction of genes-of-interest (either shared or disease-specific genes) with a high rare-variant burden. …

<table>
<tr><td colspan="2">REF2-10. Details on figure legends and main texts</td></tr>
<tr><td colspan="2">(8) In general, the breadth of the analyses performed in this study require a more detailed description to be fully grasped. This would not only facilitate the interpretation of results but also make the analyses easier to replicate in similar cohorts, ultimately enhancing the impact of this work. Here are some specific suggestions:
- Many figure legends, particularly in the main figures, are brief and do not adequately describe the plots. It’s important to clearly state what is on the x-axis and y-axis, what the colors indicate, and which data points were included or excluded.
- The design and setup of some multifactorial comparative analyses are not described in the methods section (or at least, I couldn’t locate their descriptions). For example, the analyses in Figure 6a and Figure 6d lack detailed explanations. I recommend dedicating a specific subparagraph to each of these analyses in the methods section, as this would make the results easier to evaluate.
- Figure 2e: the legend for Figure 2e should specify what is being enriched - marker genes, perhaps? Additionally, how are these marker genes defined?
- Where possible, be specific when reporting quantities, or refer to figures showing the data. For example:
Line 94: ‘roughly equal numbers’ - how many?
Line 98: “over 30% of donors” - how many?
S. Fig. 1 legend: 6M - how many cells?
Line 611: “by removing donors with very low nuclei counts” - how many donors?
Line 166: what do the authors mean by “relatively concordant”?</td></tr>
<tr><td>Response</td><td>Thank you for highlighting the difficulty in locating certain analytical details. We agree that more detailed technical descriptions are essential for fully understanding the analyses presented in this study. In response, we have revised the Methods section to include explicit figure associations. For instance, the reviewer noted that descriptions for Fig. 6a and Fig. 6d were hard to locate; these analyses were previously described under “Compositional variation analysis using Crumblr.” We have now clarified this by annotating the relevant section with references to Figs. 4a–c and 6a–c.

Additionally, we have revised all figure legends to clearly describe the plots—specifying the x- and y-axes, color encodings, and which data points were included or excluded. For example, the legend for Fig. 2e now clarifies the selection criteria for marker genes. Specifically, we identified cell-type-specific marker genes through differential expression analysis between one subclass and all others, retaining genes with statistically significant FDR values (i.e. https://diseaseneurogenomics.github.io/dreamlet/reference/dreamletCompareClusters.html).

Lastly, we addressed all other comments in the revised manuscript. This includes reporting exact numerical values where applicable and avoiding vague qualifiers.</td></tr>
<tr><td>Excerpt</td><td>“... We applied the Crumblr method (https://diseaseneurogenomics.github.io/crumblr) for testing the variation of cell type composition[40], as reflected in Figs. 4a-c, 6a-c. …”

“... To account for the scale of these data, complex study designs with repeated measures, and low read count per cell, we applied Dreamlet for differential expression analysis, which applies a pseudobulk approach, as reflected in Figs. 5a-c, 6d-e. …”</td></tr>
</table>

	"... **(e)** Functional enrichment of cellular subclasses using Gene Ontology Biological Process (GO BP). **Cell-type-specific genes were selected for each subclass using differential gene expression analysis, comparing one versus the rest of subclass-level clusters, and filtering for genes with FDR ≤ 0.05**. …"<br><br>"... roughly equal numbers of males **(n=723)** and females **(n=771)** …The cohort covers a diverse genetic background and over 30% of the donors **(n=509)** were of non-European (EUR) ancestry. …"<br><br>"... **Supplementary Fig. 1**. … **(c)** Distribution of gene counts by UMI counts after QC of **6,320,459** nuclei. …"<br><br>"... Lastly, QC was applied at the donor-level by removing any sample **represented by < 50 nuclei**. …"<br><br>"... The cellular taxonomy was consistent and well represented across all three brain sources (**Supplementary Figs. 3a-b**). Our most granular-level subtype annotation was robust and invariant to donor and technical variables (**Supplementary Fig. 3c**). …"

REF2-11. Minor comments	
Minor comments<br><br>(1) The term 'transcriptomic vulnerability' is unclear and not defined.<br>(2) Line 527: Unclear what is reference 102, I expected to find a paper describing the method for assigning genetic ancestry.<br>(3) Line 622: 'mitochondrial chromosomes and MT' - typo?<br>(4) Why are there 2 sections of the methods describing assignment of genetic ancestries from genotype data? Line 526-530 and line 711-720? Which one was used on which data?<br>(5) Why is suppl. Fig. 4a called CCA? What quantities are being correlated? Cell type proportions?<br>(6) The VAE models for dynamics seem to be trained on a very low number of epochs, at least compared to how many epochs are generally needed for an scVI model to converge. Can you show that the models have reached convergence?<br>(7) Avoid using qualifiers (e.g., "rigorous" line 605, "properly" in line 928).<br>(8) Make figure text larger where possible (fig. 1a, c-e, suppl. Fig. 1j, figure 4a).<br>(9) Is the Xenium data shared? Could the authors add at least one supplementary figure on Xenium dataset QC?	
Response	We appreciate the reviewer's attention to detail. We corrected errors and clarified the abbreviations and terms in the revised manuscript.<br><br>1. We rewrote the definition of transcriptomic vulnerability for clarity (**Excerpt 1**). Shared transcriptomic vulnerability refers to the common patterns of gene expression perturbations observed across different brain diseases. By examining cross-disease atlases, we tried to identify these shared patterns, which may have implications for early treatment and the development of effective therapeutics.<br><br>2. It refers to a data descriptor paper (Fullard, J. F. et al. Population-scale cross-disorder atlas of the human prefrontal cortex at single-cell resolution. Sci. Data), which is a part of the PsychAD package (https://psych-ad.org). We fixed this in revision by adding a brief description of how genetic ancestry assignments were performed (**Excerpt 2**).<br><br>3. Thanks for pointing out this error, which has now been fixed in the revised manuscript (**Excerpt 3**).<br><br>4. Thanks for pointing out this error. We consolidated the two sections describing genetic ancestry assignments into **Methods** section titled "Collection and harmonization of clinical, pathological, and demographic metadata" (**Excerpt 2**).

5. CCA refers to Canonical Correlation Analysis, and we clarified this in the revised manuscript (**Excerpt 4**). To assess the degree to which they co-vary and contain the same information, we are evaluating absolute correlation values of all pairs of donor-level clinical and demographic variables.

6. We agree that the number of training epochs used was relatively small for an scVI-style VAE. We note that gene reconstruction is the primary goal for most studies that use the VAE, whereas our goal was to predict donor level variables from the latent space. With the VAE, we noticed that validation accuracy began to saturate after ~4 epochs (depending on the cell class) and, more importantly, the correlation between the Braak and dementia model predictions started to increase significantly after ~4 epochs. Since we were interested in disentangling the effect of Braak stage and dementia status on gene expression, we terminated model training at this point.

We initially decided to use the scVI-style VAE architecture as the gene reconstruction helped regularize model training and improve prediction accuracy, even though the reconstructed gene output was not used for our analysis. However, after further experimentation, we decided to replace the VAE architecture with a simpler feedforward MLP with two hidden layers (described in the methods).
With appropriate training methods (e.g. replacing the Adam optimizer with vanilla SGD), the feedforward model architecture generated predictions with similar accuracy, slightly lower correlations between Braak and dementia, and greatly simplified model size and training. Using this approach, the number of training epochs is 20 for cell classes except Endo, Mural and OPC, 30 epochs for OPC which required extra training time before convergence, and 100 epochs for the (much smaller) vascular classes.

We've added the training curves showing the Braak and dementia validation accuracy, as well as the correlation between the Braak and dementia model predictions, as **Supplementary Fig. 29**.

7. We fixed this in the revision and avoided using unnecessary qualifiers.

8. We made the text bigger, as requested (**Figs. 1a, 1c-e, 4a, Supplementary Fig. 1j**).

9. Xenium data will be available via Zenodo (https://doi.org/10.5281/zenodo.14606776). The data in this repository is under a publication embargo. Reviewer access is available from https://zenodo.org/records/14606776?token=eyJhbGciOiJIUzUxMiJ9.eyJpZCI6ImZlN2I2YTgwLTNlNDItNDdjNS1hODIzLTNkMTZmOGY4N2ZjZiIsImRhdGEiOnt9LCJyYW5kb20iOiJhMTFlMzMxNWRmZjdhZjI1YTAxOWUxYjUzOGRmNjU0ZiJ9.jEGHTm0kcnFgic8zYoetmb5e2BmyqU8j5J-0aM86pFFUOQnCzCMfsmAK8DgeK2XEP_O1Md6BlxfnLQtWdfl0jQ. We also improved the description on how the processing, QC, and annotation were performed along with additional supplementary figures (**Methods, Supplementary Figs. 4a-d**).

Excerpt

Excerpt 1. Regarding transcriptomic vulnerability:
"... We uncover shared transcriptomic vulnerability across different brain diseases and, in so doing, reveal specific transcriptional patterns and regulatory drivers underpinning each trait. By characterizing putative disease-driving molecular changes across multiple traits, we differentiate shared and novel disease- and cell-type-specific associations. Concordance between heritability estimates and transcriptomic similarity identifies shared genetic factors underlying cross-disorder traits. Deep phenotyping of AD trajectories using Tau pathology and clinical dementia status suggests a potential link between immune and brain vasculature dysfunctions. …"

Excerpt 2. Regarding genetic ancestry assignments:
"… We described the process for assigning genetic ancestry in a prior data descriptor paper[23]. In brief, we leveraged quadratic discriminant analysis (QDA) to infer genetic ancestry by training our model using data from the 1000 Genomes Project. We utilized 10-fold stratified cross validation to optimize the regularization parameter within QDA[41] as well as forward selection to identify the optimal number of principal components for genetic ancestry assignments. For samples without genotypic data, we utilized race/ethnicity as a proxy for inferring genetic ancestry. We emphasize that while genetic ancestry is a distinct concept from the social constructs of race and ethnicity[42], we leveraged the correlated race/ethnicity variables as proxies to retain those samples in the analyses. Values for superpopulations included: EAS, SAS, AFR, AMR, EUR, and EAS_SAS, where the category "EAS_SAS" was assigned to samples with unavailable genotypes with an "Asian" value for race/ethnicity, which can potentially correspond to both EAS and SAS."

Excerpt 3. Typo:
"... manually excluding sex and mitochondrial chromosomes. …"

Excerpt 4. Regarding CCA:
"... Canonical Correlation Analysis (CCA) matrix showing the relationships between various clinical and demographic variables for covariate selection. …"

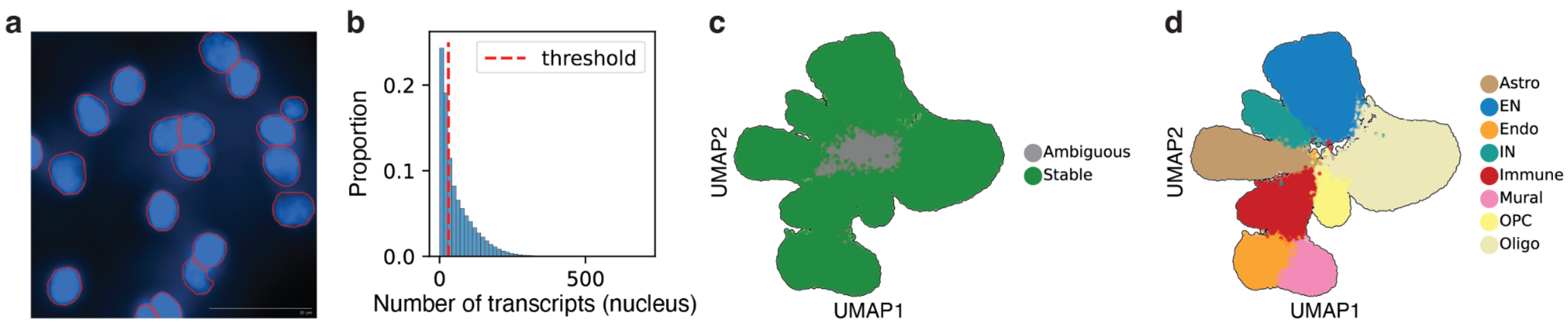

Supplementary Fig. 4. Validation of cellular taxonomy using spatial transcriptomics. **(a)** Processing and QC of Xenium in situ spatial transcriptomics data. Example image to demonstrate nuclei segmentation. The DAPI signal is shown in blue, and boundaries of segmented nuclei are shown as red outlines. **(b)** Distribution of transcript counts per nucleus across all samples. The chosen threshold (30 counts) is shown as a red dotted line for reference. **(c)** UMAP of all Xenium nuclei containing >30 transcripts. Clusters were assigned "stable" or "ambiguous" labels based on how many of their nuclei were mapped to the same class using scANVI (**Methods**). **(d)** The same UMAP as (c), after removing ambiguous clusters and nuclei (mismatch between nuclei cluster and scANVI labels). Nuclei are colored based on their assigned class. …

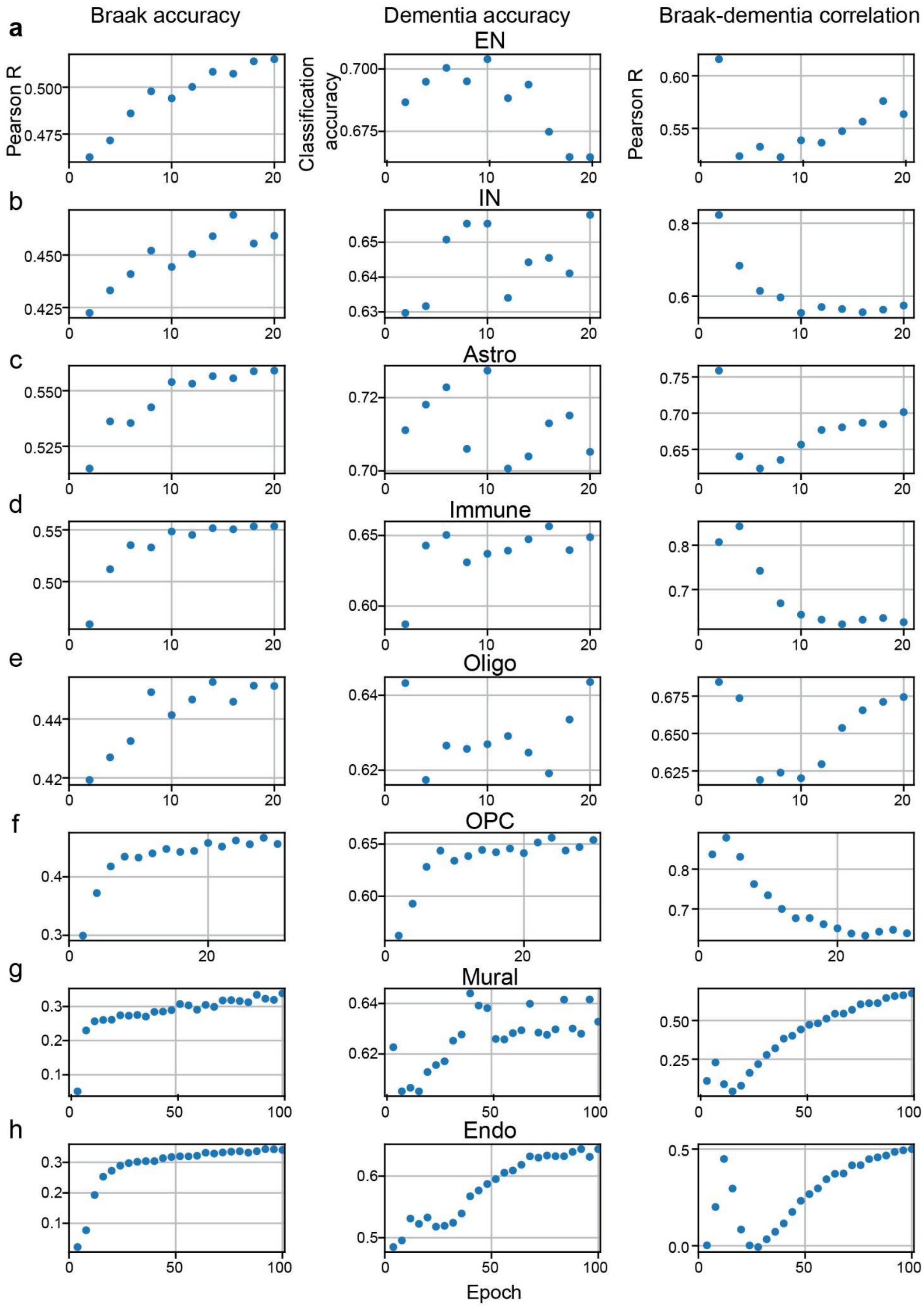

Supplementary Fig. 29. Neural network model training curves across the cell classes. (a-h) Training curves for each cell class. The left panel shows the cross-validated Pearson correlation between actual and predicted Braak values. Predictions are averaged at the donor-level. The middle panel shows the dementia classification accuracy, and the right panel shows the correlation between the donor-averaged Braak and dementia model predictions. The x-axis shows the number of training epochs.

REF2-12. Remarks on code availability	
The code repository could benefit from improvements to ensure FAIRness (Findability, Accessibility, Interoperability, and Reusability). As mentioned earlier, I wasn't able to find code for all the analyses described in the study, and in some cases, the procedures outlined in the methods section do not match the code. Here are some specific suggestions for improvement:<br><br>(1) At a minimum, please ensure that each code subfolder includes a README file that indicates the purpose of each script, as done in https://github.com/DiseaseNeuroGenomics/PsychADxD/tree/main/7_trajectory<br>(2) Several scripts refer to a custom module pge loaded from a local library (/path/to/leetools/pegasus/). Can this module be added to the repo for reproducibility?<br>(3) Please ensure that all analyses presented in the manuscript have a corresponding script or subfolder in the repository. For instance, I couldn't locate the scDRS analysis or the analyses shown in Figures 5d-g.<br>(4) While I was able to download and inspect the snRNA-seq datasets via cellxgene and access other datasets (although not all, e.g. Xenium data), expanding the "Dataset" section of the repository README would be beneficial. This section could include pointers to various types of datasets spread across platforms (e.g., raw snRNA-seq data, processed h5ad files, genotype data, patient metadata, DE analysis results, Xenium data). This would likely reduce the number of inquiries the maintainers receive asking where to find specific pieces of data.	
Response	We thank the reviewer for their thorough and constructive feedback regarding the FAIRness of our code repository. We have made significant revisions to address these points and improve the findability, accessibility, interoperability, and reusability of our code. Here's a point-by-point response to the reviewer's comments:<br><br>- We updated README files in each code subfolder, detailing, as suggested, the purpose of each script and providing usage examples(https://github.com/DiseaseNeuroGenomics/PsychADxD/tree/main/2_taxonomy).<br>- A collection of custom functions (i.e., /path/to/leetools/pegasus), which was adopted from https://github.com/leelaboratory/leetools/blob/main/pegasus/pge.py, has been added to GitHub repo (https://github.com/DiseaseNeuroGenomics/PsychADxD/blob/main/2_taxonomy/pge.py).<br>- We have now added the code for the scDRS analysis and other genetic heritability analyses presented in Figures 5d-g (LD Score Regression (LDSC)) to the repository (https://github.com/DiseaseNeuroGenomics/PsychADxD/tree/main/8_genetics).<br>- We have also added QC and iterative clustering scripts (https://github.com/DiseaseNeuroGenomics/PsychADxD/tree/main/2_taxonomy).<br>- As suggested, we have added a "Dataset" section to the repository's README file. This section now provides pointers to the datasets used in the study. Due to a data release embargo, we have temporarily removed the direct link to the cellxgene resource. We will ensure this link is updated with the public URL as soon as the manuscript is published and the data becomes publicly available.

Excerpt	

2_taxonomy: preprocessing and hierarchical cellular taxonomy of the snRNA-seq data

Requirements

A collection of custom pegasus functions was adopted from https://github.com/leelaboratory/leetools/blob/main/pegasus/pge.py

Step 1 - Preprocessing

Performs QCs at gene-level, cell-level, and library-level to filter out low-quality cells using pegasus. Performs shifted log-normalization and signature score required in subsequent processing.

Example Usage

```
python PsychAD_pegasus_end2end_1_qc.py --input <Input h5ad file> --output <Output h5ad file>
```

Step 2 - Highly variable feature

Highly variable feature selection. scanpy's `highly_variable_genes` function was extended to select from protein-coding genes in autosome.

Example Usage

```
PsychAD_pegasus_end2end_2_hvf.py --input <Input h5ad file> --output <Output h5ad file> --flavor cell_ranger --n_top_(
```

Step 3 - First pass clustering

First pass clustering analysis using pegasus. This step is required to prepare for the doublet removal step.

Example Usage

```
python PsychAD_pegasus_end2end_3_pass.py --input <Input h5ad file> --output <Output h5ad file> --n_pcs 50 --batch po(
```

Step 4 - Doublet removal

Removal of doublets using scrublet.

Example Usage

```
python PsychAD_pegasus_end2end_4_scrublet.py --input <Input h5ad file> --output <Output h5ad file>
```

Step 5 - Iterative clustering

Iterative clustering to define the hierarchical structure of clustering.

Example Usage

```
python PsychAD_pegasus_end2end_5_iclust.py --input <Input h5ad file> --output <Output h5ad file>
```

Referee #3 (Remarks to the Author):

REF3-1. Overall assessment of the manuscript	
Lee et al. have produced an impressively large snRNA-seq dataset from the human prefrontal cortex and analyzed it to identify patterns of differential expression across disease states. Overall, the enormous size of the data resource makes this an important resource, and researchers studying any of the diseases included in the atlas are likely to find valuable information embedded in the supplementary tables. However, the biological insights described in the main text do not provide substantial novel insights for two reasons. The cross-disorder analyses are rather superficial and may be confounded by a wide range of technical and biological factors. The AD-specific analyses are somewhat more detailed. The descriptions of disease trajectories and the dynamics of differential gene expression (Fig. 7) are quite interesting. However, the findings largely recapitulate those of previous snRNA-seq studies of AD and ADRDs without providing any new mechanistic understanding beyond what has already been shown. In addition, some details are missing that aid in the evaluation of the resource's quality and in the interpretation of the results.	
Response	We acknowledge and respect the concerns raised regarding the conceptual advance over earlier studies and the strength of novel conclusions. We'd like to point out that this study is part of a larger package of papers (see https://psych-ad.org), including studies with different focuses that delve deeper into specific subject areas such as healthy aging, QTL, TWAS, and disease phenotyping. These complementary manuscripts provide further insights into the complex landscape of human brain disorders. Importantly, this study integrates single-nucleus data from 1,494 brains across 8 neurodegenerative and neuropsychiatric conditions, making it the largest harmonized disease atlas of its kind. This scale enabled robust, multi-level cellular taxonomy and unprecedented statistical power to characterize both shared and disease-specific transcriptomic changes.<br><br>In this revision, we validated our compositional variation and gene expression variation findings by replicating results using two independent published datasets[6,7]. We also validated the compositional change using a published MERFISH dataset[7], showing the observed change is robust to technology used. We also strengthened the cross-disorder analyses by removing shared transcriptomic effects and focusing on disease-specific signatures. These core signatures were found to be enriched for rare variant burden (**Supplementary Fig. 8e**), supporting the idea that genes with disease-specific effects may represent stronger mechanistic candidates.<br><br>Beyond descriptive comparisons, we introduced a decorrelated modeling framework to disentangle tau pathology from cognitive decline. This allowed us to identify nonlinear, temporally ordered gene programs across cell types, highlighting early and late phases of immune activation, lipid dysregulation, and synaptic compensation (**Fig. 7**). To our knowledge, this level of trajectory resolution, including dementia resilience modeling, has not been reported at this scale in prior AD single-cell studies.<br><br>We also implemented a causal mediation analysis that connects polygenic risk to plaque and tangle pathology and downstream cognitive impairment via changes in specific cell subclasses. These models identified immune and vascular mediators of pathology, offering mechanistic hypotheses for future functional validation.<br><br>Finally, we incorporated detailed neuropsychiatric symptom annotation, enabling associations between deep-layer excitatory neuron subclasses and weight loss in AD, a connection not previously explored in other large-scale studies.<br><br>We believe that the combined impact of these studies will be substantial, offering a comprehensive understanding of these conditions and paving the way for new therapeutic strategies.

REF3-2. QC metrics

1. Fig. S1. This is a massive dataset. The strategy for multiplexing and demultiplexing samples, including replicate samples for each donor, is well constructed. Overall, the quality metrics look good. It would be helpful to see the QC metrics separated out by batch and disease state, here or in a supplementary table.

Response

We agree that further breakdown of the QC metrics is valuable. As suggested, we have now included a **Supplementary Table 12** detailing the QC statistics (median gene and UMI counts, median percent mitochondrial genes, and cell counts), separated by 2,924 single-cell libraries utilized, before and after the removal of low-quality cells. This allows for a clearer understanding of the impact of our QC pipeline. Furthermore, we have expanded the Methods section to explicitly describe the rigorous three-step QC criteria applied at the gene, cell, and library levels. Finally, the relevant scripts used in this analysis are publicly available and accessible at: https://github.com/DiseaseNeuroGenomics/PsychADxD/tree/main/2_taxonomy.

Excerpt

"... **QC**. We applied a rigorous three-step QC process to filter out low quality nuclei for subsequent downstream analyses. First, QC was applied at the cell-level. Poor-quality nuclei were detected by thresholding based on UMI counts, gene counts, and mitochondrial content. The QC thresholds were defined from log-transformed gene and UMI counts. Three median absolute deviations (MAD) were used as the lower limit. This resulted in 986 for genes and 1,179 for UMI counts as lower bounds. We used a hard cutoff of 15,000 genes and 200,000 UMI counts as upper limits. Any cells with mitochondrial genes greater than 1% were filtered out. We checked for possible contamination from ambient RNA using CellBender[24]. We also checked for cells with high fractions of reads that mapped to non-mRNAs, like rRNA, sRNA, and pseudogenes, as well as known confounding features such as lncRNA MALAT1. Second, QC was applied at the feature-level by removing those that were not robustly expressed in at least 0.05% of nuclei. Lastly, QC was applied at the donor-level by removing any sample represented by < 50 nuclei. Finally, we excluded donors with low genotype concordance and sex discrepancies. Detailed QC statistics (median gene and UMI counts, median percent mitochondrial genes, and cell counts), separated by 2,924 single-cell libraries utilized, before and after the removal of low-quality cells are available in **Supplementary Table 12**. …"

Library	kept	median_n_genes	median_n_umis	median_percent_mito	filt	total	median_n_genes_before	median_n_umis_before	median_percent_mito_before
**Donor_0001-1**	3089	3368	9010	0.19470567	93	3182	3356	8930	0.20319362
**Donor_0001-2**	3281	3761	10693	0.20889285	130	3411	3733	10566	0.21818937
**Donor_0002-1**	2360	2609	6218.5	0.07513537	41	2401	2606	6198	0.077745385
**Donor_0002-2**	2968	2533.5	5630	0.08097639	64	3032	2524	5594	0.0835795
**Donor_0003-1**	4955	3400	7538	0.073823676	51	5006	3375	7460	0.07492897
**Donor_0003-2**	4901	3336	7314	0.07716421	93	4994	3305.5	7156	0.078943595
**Donor_0004-1**	4957	3014	7124	0.096339114	43	5000	3013	7106	0.09751529
**Donor_0004-2**	5114	2965.5	6908	0.09499	43	5157	2965	6899	0.095744684
**Donor_0005-1**	483	2559	5647	0.46781436	347	830	2406.5	5107	0.8412919
**Donor_0005-2**	509	2702	5917	0.46372974	237	746	2574	5501	0.7014957
**Donor_0006-1**	3648	2596	5395.5	0.22527874	301	3949	2602	5415	0.24505183
**Donor_0006-2**	3691	2857	6225	0.22477521	309	4000	2847	6208.5	0.24306372
**Donor_0007-1**	3855	4977	13625	0.09967625	85	3940	4855	13131	0.10311271
**Donor_0007-2**	3806	5035	14057.5	0.0947568	108	3914	4831.5	13448.5	0.09828773
**Donor_0008-1**	3091	2732	6060	0.18298261	73	3164	2717.5	5992.5	0.18799938
**Donor_0008-2**	3041	2850	6490	0.1736312	93	3134	2824	6402.5	0.17881148
**Donor_0009-1**	3208	3180.5	7354.5	0.069482654	16	3224	3163	7297	0.07004912
**Donor_0009-2**	2980	3245	7775.5	0.06710793	12	2992	3242	7746	0.06732495
**Donor_0010-1**	2722	3650.5	11442	0.22299865	396	3118	3641.5	11259	0.27220422
**Donor_0010-2**	2829	3945	12982	0.22603236	306	3135	3883	12488	0.25944442

Supplementary Table 12. The QC statistics of median gene and UMI counts, median percent mitochondrial genes, and cell counts, separated by 2,924 single-cell libraries utilized, before and after the removal of low-quality cells.

REF3-3. Cell type annotations

2. Cell type annotations (starting at line 136): The authors note that they used the Ma 2022 (citation 6) and BICCN 2021 (citation 7) papers, but it is difficult to see how these were used from the description of the methods and the code on github. Many of the

	subtypes differ in nomenclature from those papers. If the authors are establishing a new cell-type nomenclature, they should explain their reasoning for diverging from the nomenclature in Ma 2022, which also studies the PFC.
Response	We thank the reviewer for this important comment regarding cell type annotation. To provide greater clarity, we have modified the description of our cell type annotation methodology in the **Methods** section and updated the code on GitHub. We also added text to explain the need to create a new 3-level hierarchical cellular taxonomy and have included an additional resource to match our cellular taxonomy with other large-scale published snRNA-seq studies (**Supplementary Table 11**).<br><br>Briefly, after quality control and log-normalization, we performed a first-pass Leiden clustering on the entire dataset. This initial clustering served as input for our doublet inference step. Following doublet removal, we employed iterative clustering across three hierarchical levels. The resulting clusters were aggregated into pseudobulk samples. We then calculated cluster-wise Pearson correlation coefficients using the annotations from both Ma 2022[25] and BICCN 2021[26]. Cell type annotations were assigned based on a combination of high correlation and cell type specificity, with the results summarized in **Supplementary Table 11**.<br><br>We opted to create a new nomenclature because a clear, consensus reference taxonomy for the dorsolateral prefrontal cortex (DLPFC) region was lacking. Existing studies of the primate PFC, including Ma 2022, derived their taxonomy from smaller numbers of nuclei and, thus, have limitations with regard to their sensitivity to detect rare cell types. In addition, these studies utilize different nomenclatures, making direct comparison and adoption challenging. Specifically, there is no established consensus on the hierarchical level of annotation. For example:<br><br>* ROSMAP (Mathys 2023[6]) divides top-level annotations into "major cell type" and "cell type."<br>* SEA-AD (Gabitto 2024[7]) uses three levels: "class," "subclass," and "supertype."<br>* Ma-Sestan (Ma 2022[25]) also uses three levels: "class," "subclass," and "subtype."<br><br>This variability in nomenclature and hierarchical organization is further illustrated by considering specific cell types. For instance, the L2/3 IT cell type is designated as a "subclass" in SEA-AD (Gabitto 2024), a "cell type" (Exc L2-3 CBLN2 LINC02306) in ROSMAP (Mathys 2023), and a "subclass" (L2-3 IT) in Ma-Sestan (Ma 2022). Similarly, the IN_SST cell type is a "subclass" (Sst) in SEA-AD (Gabitto 2024), a "cell type" (Inh L3-5 SST MAFB) in ROSMAP (Mathys 2023), and a "subclass" (SST) in Ma-Sestan (Ma 2022). These examples highlight the inconsistencies and lack of standardization in current PFC cell type annotations.<br><br>Therefore, rather than directly adopting potentially inconsistent nomenclature, we aimed to create a new, internally consistent nomenclature that reflects the specific cell types identified in our DLPFC dataset. We believe this approach provides greater clarity and avoids potential misinterpretations arising from the use of existing, but non-standardized, nomenclatures.<br><br>Our class and subclass level annotation closely match Ma 2022. At the subtype level, we observed greater discrepancy with markers identified from other studies. We ultimately found that our single-cell transcriptome data has more power to distinguish rare cell types and their marker genes. Despite the use of this new nomenclature, our cellular taxonomy closely matches published datasets with a mean Pearson correlation coefficient of approximately 0.75 (compared to SEA-AD, Gabitto 2024).
Excerpt	**Defining cellular taxonomy using iterative clustering**<br>**Hierarchical cellular taxonomy**. Cellular taxonomy was defined using a divide-and-conquer strategy. From the full dataset containing over 6 million nuclei, 8 major cell classes were defined using the following steps: 6,000 highly variable genes (HVGs) were selected from mean and dispersions trends[27] using the default parameters (min_mean=0.0125, max_mean=3, min_disp=0.5) and brain source as a batch variable after manually excluding sex and mitochondrial chromosomes. We used the k-nearest-neighbor (kNN) graph calculated on the basis of harmony-corrected PCA embedding space to cluster nuclei of the same cell type using Leiden[28] clustering algorithms. We

used UMAP[29] to visualize the resulting clusters. From the class-level clusters, we subsetted the data by each class. Re-calculating HVGs among cells in the same class allowed us to re-focus on a feature space that is more relevant for the same class of cells. A kNN graph was then calculated on the basis of the harmony-corrected PCA of the selected HVGs. Leiden-clustering was used to annotate 27 subclass-level annotations. We iterated the same HVG-kNN-Leiden clustering for all 27 subclasses yielding 67 subtypes of human brain cells. After obtaining annotations in three-levels of hierarchy, the resulting clusters were aggregated into pseudobulk and cluster-wise Pearson correlation coefficient was calculated using existing human DLPFC[25] and M1[26] annotations. We matched the annotations based on both high correlation and specificity of the cell type. The final cellular taxonomy was compared to the following snRNA-seq datasets: PFC from ROSMAP cohort[6] and PFC (Brodmann area 9) from SEA-AD cohort[7] (**Supplementary Table 11**). …

PsychAD taxonomy	annotation level	SEA-AD (Gabitto 2024) subclass	SEA-AD (Gabitto 2024) subclass max Pearson corr	SEA-AD (Gabitto 2024) supertype	SEA-AD (Gabitto 2024) supertype max Pearson corr	ROSMAP (Mathys 2023 major cell type	ROSMAP (Mathys 2023 major cell type max Pearson corr	ROSMAP (Mathys 2023 cell type	ROSMAP (Mathys 2023 cell type max Pearson corr	Ma-Sestan (Ma 2022) subclass	Ma-Sestan (Ma 2022) subclass max Pearson corr	Ma-Sestan (Ma 2022) subtype	Ma-Sestan (Ma 2022) subtype max Pearson corr
EN_L2_3_IT	subclass	L2/3 IT	0.9187288212	L2/3 IT_6,L2/3 IT_1,L2/3	0.8611772721	Exc	0.7717483474	Exc L2-3 CBLN2 LINC02	0.9172254164	L2-3 IT	0.8725840403	L2-3 CUX2 ACVR1C TH	0.8046401429
EN_L3_5_IT_1	subclass	L4 IT	0.8158918528	L4 IT_4	0.8810472557	Exc	0.6622488901	Exc L3-5 RORB PLCH1	0.909090236	L3-5 IT-1	0.7324995101	L3-5 RORB PCBP3 IL1R	0.767696478
EN_L3_5_IT_2	subclass	L4 IT	0.8291418979	L4 IT_2,L5 IT_1,L5 IT_3	0.8750498059	Exc	0.7213865133	Exc L4-5 RORB GABRG	0.9187347817	L3-5 IT-2	0.8693074558	L3-5 RORB GABRG1 KC	0.8065500544
EN_L3_5_IT_3	subclass	L5 IT	0.8959538072	L5 IT_2,L4 IT_1,L5 IT_5,	0.8507641797	Exc	0.7401590327	Exc L4-5 RORB IL1RAP	0.9007103521	L3-5 IT-3	0.8648960962	L3-5 RORB MKX DCC,L	0.7522985044
EN_L5_6_NP	subclass	L5/6 NP	0.9145852941	L5/6 NP_2,L5/6 NP_1,L5	0.8749730696	Exc	0.4341197832	Exc L5/6 NP	0.9007561907	L5-6 NP	0.83685155	L5-6 FEZF2 NXPH2 CDH	0.7595088045
EN_L5_ET	subclass	L5 ET	0.7265062878	L5 ET_2	0.7029362929	Exc	0.4354458422	Exc L5 ET	0.6741477044	L5 ET	0.631834104	L5 FEZF2 BCL11B POU	0.6174767666
EN_L6B	subclass	L6b	0.8500651765	L6b_6,L6b_1,L6b_2,L6b	0.7984820883	Exc	0.5597979258	Exc L6b	0.8603038306	L6B	0.8342116517	L6 FEZF2 NPFFR2 KCN	0.7542275073
EN_L6_CT	subclass	L6 CT	0.898810144	L6 CT_1,L6 CT_2,L6 CT	0.8350324711	Exc	0.5933707096	Exc L6 CT	0.8945312035	L6 CT	0.8355778965	L6 FEZF2 SYT6 PTPRT,	0.7426814055
EN_L6_IT_1	subclass	L6 IT	0.9098830697	L6 IT_2,L6 IT_1,L5 IT_7	0.8831111638	Exc	0.7151890216	Exc L6 THEMIS NFIA	0.8780584942	L6 IT-1	0.8595272792	L6 OPRK1 THEMIS RGS	0.8254555818
EN_L6_IT_2	subclass	L6 IT Car3	0.8957187821	L6 IT Car3_3,L6 IT Car3	0.8706138304	Exc	0.5443088585	Exc L5/6 IT Car3	0.8765137034	L6 IT-2	0.8295278577	L6 OPRK1 SMYD1 KCN	0.7439007504
IN_ADARB2	subclass	Sncg,Pax6	0.7916130687	Lamp5_5,Lamp5_6,Pax6	0.6779338755	Inh	0.5874842287	Inh PTPRK FAM19A1,Inh	0.7489585106	ADARB2 KCNG1	0.7771300079	InN ADARB2 FAM19A1,I	0.6183164533
IN_LAMP5_LHX6	subclass	Lamp5 Lhx6	0.9021993061	Lamp5_Lhx6_1	0.8746819368	Inh	0.5669116108	Inh L1-6 LAMP5 CA13	0.8905215134	LAMP5 LHX6	0.7752169422	InN LAMP5 LHX6 TAC1,	0.7065330997
IN_LAMP5_RELN	subclass	Lamp5	0.8704028684	Lamp5_2,Lamp5_1,Lam	0.8466160021	Inh	0.489139614	Inh LAMP5 NRG1 (Rose	0.8957379443	LAMP5 RELN	0.8133243353	InN LAMP5 BMP7,InN L	0.7927525983
IN_PVALB	subclass	Pvalb	0.9263062318	Pvalb_2,Pvalb_6,Pvalb_	0.8670196042	Inh	0.6269203326	Inh PVALB HTR4,Inh GP	0.8889459755	PVALB	0.8777941672	InN PVALB ANOS1,InN	0.7950429635
IN_PVALB_CHC	subclass	Chandelier	0.893270175	Chandelier_1,Chandelier	0.8693547749	Inh	0.525996677	Inh PVALB CA8 (Chande	0.8716531309	PVALB ChC	0.7957038942	InN PVALB PDE3A,InN	0.7273340271
IN_SST	subclass	Sst,Sst Chodl	0.8835103807	Sst_3,Sst Chodl_1,Sst_1	0.7459501606	Inh	0.6652509188	Inh L3-5 SST MAFB,Inh	0.8118387333	SST,SST HGF,SST NPY	0.8413625503	InN SST TRPC7,InN SS	0.6829729597
IN_VIP	subclass	Vip	0.9081041439	Vip_12,Vip_15,Vip_4,Vip	0.7503065862	Inh	0.619090261	Inh VIP CLSTN2,Inh AL	0.8091660067	VIP	0.8560988165	InN VIP HS3ST3B1,InN	0.6941960572
Astro	subclass	Astrocyte	0.941960136	Astro_2,Astro_1,Astro_6	0.9247545956	Ast	0.8708978562	Ast GRM3,Ast DPP10,As	0.9210120156	Astro	0.9024143001	Astro AQP4 SLC1A2,Ast	0.8733404618
OPC	subclass	OPC	0.9165489687	OPC_2,OPC_1,OPC_2_	0.9077318788	Opc	0.818570085	OPC DOCK5,Opc GRIA4	0.8989758515	OPC	0.8625147801	OPC PDGFRA PCDH15,	0.8424732612
Oligo	subclass	Oligodendrocyte	0.9471532343	Oligo_2,Oligo_1,Oligo_4	0.9061543538	Oli	0.8943853615	Oli OPALIN,Oli RASGRF	0.9306266642	Oligo	0.8984764384	Oligo MOG OPALIN,Olig	0.8867157111
Micro	subclass	Microglia-PVM	0.933464529	Micro-PVM_2,Micro-PVM	0.9431161957	Mic	0.8750903614	Mic P2RY12,Mic DUSP1	0.9205525592	Micro	0.8716353724	Micro P2RY12 APBB1IP,	0.8531886838
PVM	subclass	Microglia-PVM	0.8004772944	Micro-PVM_1,Monocyte	0.8024480424	Mic	0.7480330851	CAMs	0.8042346208	Micro	0.6953629214	Macro F13A1 COLEC12,	0.7407988262
Adaptive	subclass	Microglia-PVM	0.5192119724	Lymphocyte	0.7596051787	Mic	0.472210012	T cells	0.7455249921	Immune	0.612156929	T SKAP1 CD247,B EBF1	0.6940775816
VLMC	subclass	VLMC	0.574201537	VLMC_1	0.7169092979	Vas	0.5200110149	Fib1,Fib2,Fib3	0.6859019018	VLMC	0.6562826098	VLMC COL1A2 SLC13A	0.5998101471
SMC	subclass	VLMC	0.5346194194	Pericyte_1	0.5084966195	Vas	0.4684040835	vSMC	0.5497551139	SMC	0.5036573682	SMC ACTA2 CNN1	0.5024970327
PC	subclass	VLMC	0.8021404483	Pericyte_1,SMC-SEAAD	0.8536162061	Vas	0.628928542	Per1,Per2	0.8112394489	PC	0.7110466179	PC P2RY14 GRM8,SMC	0.6914495179
Endo	subclass	Endothelial	0.8437066858	Endo_2	0.8774086393	Vas	0.6721570177	capEndo,aEndo,aSMC,v	0.8672182435	Endo,RB	0.8101485547	Endo CLDN5 SLC7A5,E	0.8088990719

Supplementary Table 11. Comparison of PsychAD cellular taxonomy to published snRNA-seq datasets (SEA-AD Gabitto 2024 and ROSMAP Mathys 2023).

REF3-4. Compositional analysis using taxonomic hierarchy

3. Given the cell type proportion differences observed across ages and diagnoses (and to a proportional extent brain banks), it is important to identify in the course of iterative clustering what is driving each cluster split. How do the authors ensure that subclustering truly identifies subtypes vs. altered cell states due to age, diagnosis, collection site, etc.? This seems well-defined at the subclass level, but less so at the finer subtype resolution. A subset of this information is provided in the plots for the distributions of cell types across brain banks and in the spatial transcriptomics data. However, this information is not presented in a sufficiently comprehensive fashion to thoroughly evaluate the quality of the sub-clustering. Were spatial transcriptomics data informative at the subtype level for INs and ENs? A further possibility would be to add metrics such as LISI to systematically evaluate the fine-level sub-types across variables.

Response

We thank the reviewer for this valuable suggestion. In response, we performed additional analyses to systematically evaluate the robustness of our finer-level subtype annotation.

In specific, we performed the LISI (Local Inverse Simpson's Index) analysis[43] as suggested to evaluate the influence of age, diagnosis, and brain source on our most granular-level subtype annotation, providing a quantitative assessment of mixing across covariates during subclustering (**Methods**). Overall, we found our subtype annotation was more robust and invariant to donor and technical variables than other granular-level annotations from previous publications (**Supplementary Fig. 3c**).

We further evaluated whether EN and IN subtypes were discernible using our Xenium spatial transcriptomics data. To this end, we performed additional label transfers at the subtype-level using scANVI[44]. We found that the subtypes were well-resolved in the spatial data, based on a comparison of pseudobulk-level transcriptional similarity between the predicted subtypes from the Xenium and snRNA-seq from the RADC cohort (**Supplementary Figs. 4g-h**). Furthermore, we found that EN subtypes exhibited finer layer structures within those defined by EN subclasses (**Supplementary Fig. 4j**), and a

subset of IN subtypes showed distinct layer-specific enrichment (**Supplementary Fig. 4f**). For example, all of IN_SST subtypes had distinct layer-specific enrichment patterns (**Supplementary Fig. 4k**).

Excerpt

"… The cellular taxonomy was consistent and well represented across all three brain sources (**Supplementary Figs. 3a-b**). Our most granular-level subtype annotation was robust and invariant to donor and technical variables (**Supplementary Fig. 3c**). …"

" …

Defining cellular taxonomy using iterative clustering

… **Evaluation of cellular taxonomy**. To ensure that subsequent clustering truly identifies biological subtypes that are not confounded by technical variables as brain sources or donor variables such as age and sex, we evaluated the quality of our most granular subtype-level cell annotations with other published datasets[6,7]. To quantify the separability of cell groupings across datasets, we followed the approach of previous work where Local Inverse Simpson's Index (LISI) was used to quantify the contribution of biological and technical factors to transcriptional variation[43]. We computed the LISI scores using various covariates, including cell type, donor, age, tissue source, and sex. LISI measures the degree of local label mixing in low-dimensional space and is sensitive to the number of distinct clusters present in a dataset. To ensure comparability across datasets, we applied LISI using a harmonized set of metadata categories and calculated scores using the compute_lisi function with a perplexity of 30 and nn_eps = 0, based on embeddings derived from a randomly sampled subset of cells. Since datasets with more granular annotations (i.e. greater numbers of clusters) tend to yield higher LISI values due to increased opportunities for label separation in local neighborhoods, we normalized the mean LISI by dividing it by the natural logarithm of the number of clusters (adjusted LISI = mean LISI / log(n_clusters)). This log-based normalization compresses the scale of the cluster count, helping to avoid over-penalizing datasets with finer annotations while still accounting for granularity-related bias. The resulting adjusted LISI scores allow for more equitable comparisons of separability across datasets with varying label resolution. …"

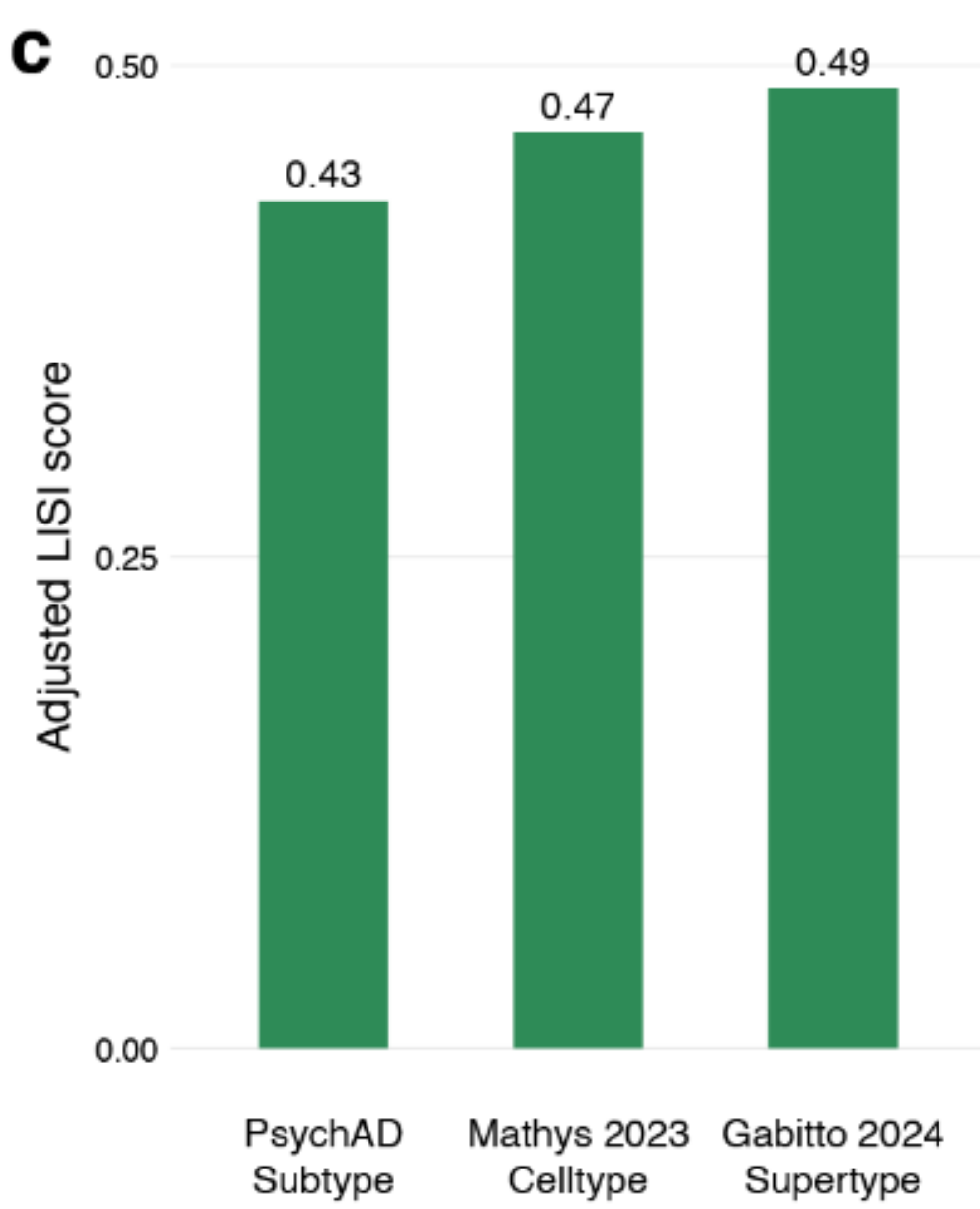

Supplementary Fig. 3. … **(c)** Evaluation of PsychAD subtype-level annotation using two independent published datasets. Barplot showing the adjusted Local Inverse Simpson's Index (LISI) scores[43] for PsychAD subtype, ROSMAP[6] celltype, and SEA-AD[7] supertype annotations. LISI quantifies the local label separability of cell groupings based on annotated cell types or subtypes. To account for the inflation of LISI values in datasets with more fine-grained annotations, mean LISI was normalized by the natural logarithm of the number of clusters (adjusted LISI = mean LISI divided by log of the number of clusters). Lower adjusted LISI scores indicate greater local mixing relative to annotation granularity, suggesting better integration or less artificial separation. PsychAD subtype exhibited the lowest adjusted LISI, followed by ROSMAP celltype and SEA-AD supertype. …

"... Notably, we observed SST+ inhibitory neuron subtypes had distinct layer-specific enrichment patterns (**Supplementary Figs. 4f,k**)."

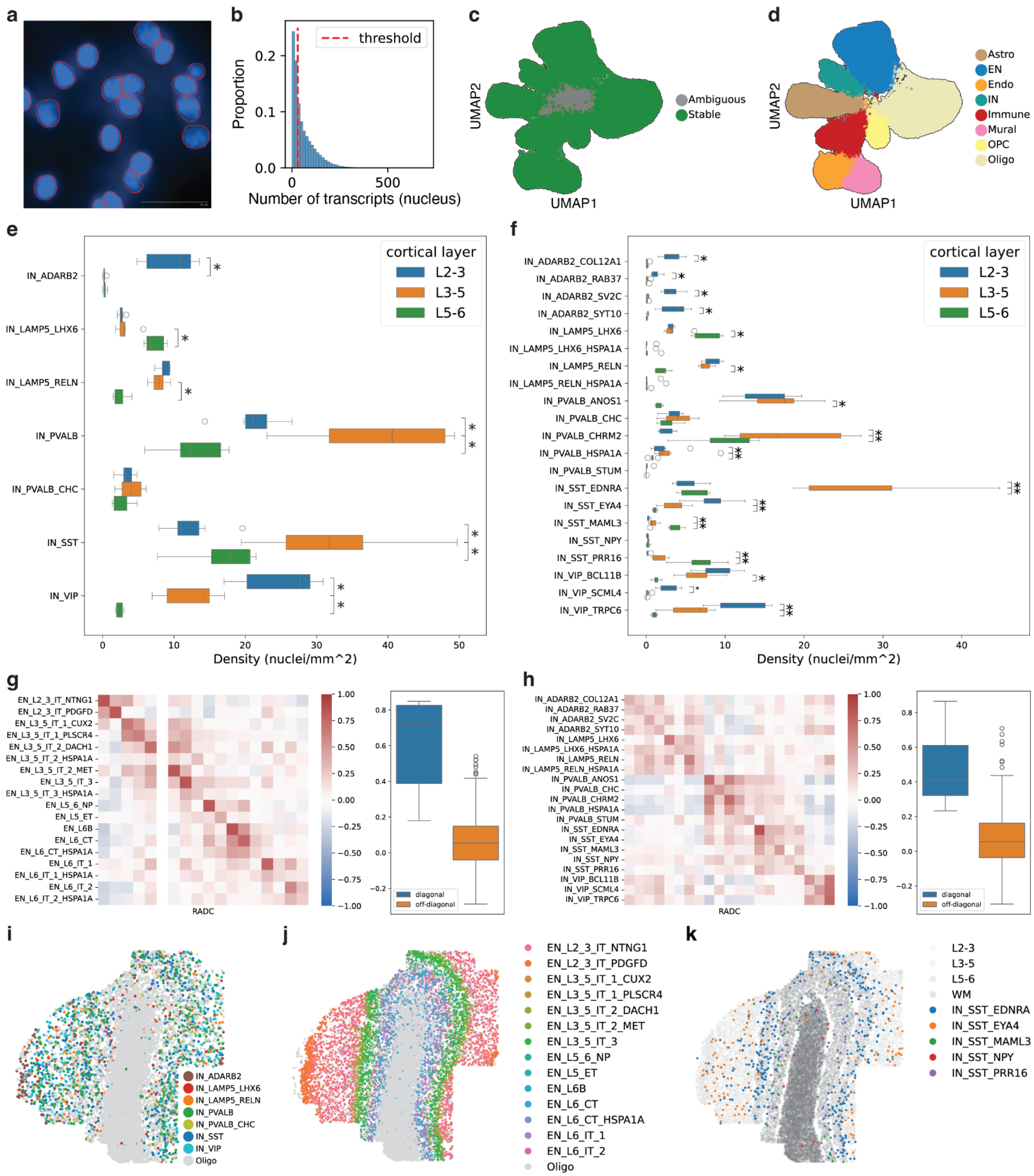

	**Supplementary Fig. 4**. Validation of cellular taxonomy using spatial transcriptomics. **(a)-(d)** Processing and QC of Xenium in situ spatial transcriptomics data. **(a)** Example image to demonstrate nuclei segmentation. The DAPI signal is shown in blue, and boundaries of segmented nuclei are shown as red outlines. **(b)** Distribution of transcript counts per nucleus across all samples. The chosen threshold (30 counts) is shown as a red dotted line for reference. **(c)** UMAP of all Xenium nuclei containing >30 transcripts. Clusters were assigned "stable" or "ambiguous" labels based on how many of their nuclei were mapped to the same class using scANVI (**Methods**). **(d)** The same UMAP as (c), after removing ambiguous clusters and nuclei (mismatch between nuclei cluster and scANVI labels). Nuclei are colored based on their assigned class. **(e)** Comparison of nuclei density of IN subclasses by cortical layers (**Methods**). Note that y axes are in units of 1/μm^2 scaled with a factor of 1e-5 (equivalent to 10 nuclei per mm^2). Paired-sample Wilcoxon rank sum testing between adjacent cortical layers. Asterisk denotes p-value < 0.05. **(f)** Comparison of nuclei density of IN subtypes by cortical layer for IN subtypes, similarly to the subclass-level analysis in (e). **(g-h)** Concordance of snRNA-seq and Xenium **(g)** EN and **(h)** IN subtypes. Pearson correlation between each pair of subtypes between Xenium (rows) and the RADC cohort snRNA-seq (columns) at pseudo-bulk level (see **Methods**). Adjacent box-plots compare the diagonal and off-diagonal elements. **(i)** Example spatial distribution of IN subclasses. A single tissue section measured with Xenium in situ spatial transcriptomics data. **(j)** Example spatial distribution of EN subtypes. **(k)** Example spatial distribution for SST-expressing IN subtypes. Coarse cortical layer structure is shown in greyscale.

REF3-5. Variance partition analysis at each cell type	
4. Figure 3. Applying variancePartition analysis across cell types does not seem to have produced any useful results. Not surprisingly, most of the variation is across cell types. However, variancePartition does not seem to have pulled out interesting cell type markers. Rather, the top genes merely describe differences between major cell classes that are very well known. variancePartition results for other variables seem essentially uninterpretable. The most interesting genes are those that show highly cell type-specific expression patterns, as well as associations with disease, age, or other variables. But the variancePartition analysis as constructed is not designed to pull out that kind of effect. It is possible that a variancePartition analysis performed separately in each cell type could produce more informative and interesting results.	
Response	We would like to clarify that VariancePartition (VP) analysis was not optimized for identifying marker genes, nor was that its intended purpose in our workflow. Rather, it serves to assess which covariates are most influential in driving expression variability, which can guide downstream modeling or prioritization of biological hypotheses. For identifying and quantifying disease- and age-effects on cell types or genes, we performed more specific quantitative analyses modeling after complex study design and various covariates using dreamlet and crumblr.<br><br>However, we agree that running VP across all cell types may obscure cell type-specific signals, particularly those of biological interest such as associations with diagnosis, age, or other covariates. To address this, we performed additional sensitivity analyses to show that having more cell types does not obscure the results. In summary, we found that running cell-type-specific VP analysis does not necessarily add more biological insights. Instead, we argue that performing VP analysis across all subclasses adds more power to discover a comprehensive source of expression variation.<br><br>In detail, we performed VP analyses within more homogeneous cell populations by stacking cell type-specific assays into three superclasses, namely, EN, IN, and Non-Neuron, similar to previous approach by BICCN exploring inter-individual variation of human brain[31]. This setup enables better resolution of potentially meaningful covariates within a cell class while still adjusting for sample- and technical-level confounders.<br><br>Overall, we observed highly concordant VP results between VP performed separately in each superclass and VP performed with all subclasses. The dominant source of variance remained the same as the CellType followed by the BrainDonor (**Supplementary Fig. 6j**). Spearman correlation of estimated

	variance between two models were high across all covariates including BrainDonor, Age, Sex, Ancestry, and Diagnosis (**Supplementary Fig. 6k**).
Excerpt	**Variance partition analysis of gene expression**<br>… We performed sensitivity analyses to ensure that performing variance partition analysis across all subclasses does not obscure the cell-type-specific signals. We performed variance partition analyses within more homogeneous cell populations by stacking cell type-specific assays into three superclasses, namely, EN, IN, and Non-Neuron, similar to previous approach by BICCN exploring inter-individual variation of human brain[31]. This setup enables better resolution of potentially meaningful covariates within a cell class while still adjusting for sample- and technical-level confounders. Overall, we observed highly concordant variance partition results between variance partition performed separately in each superclass and variance partition performed with all subclasses. The dominant source of variance remained the same as the CellType followed by the BrainDonor (**Supplementary Fig. 6j**). Spearman correlation of estimated variance between two models were high across all covariates including BrainDonor, Age, Sex, Ancestry, and Diagnosis (**Supplementary Fig. 6k**).<br>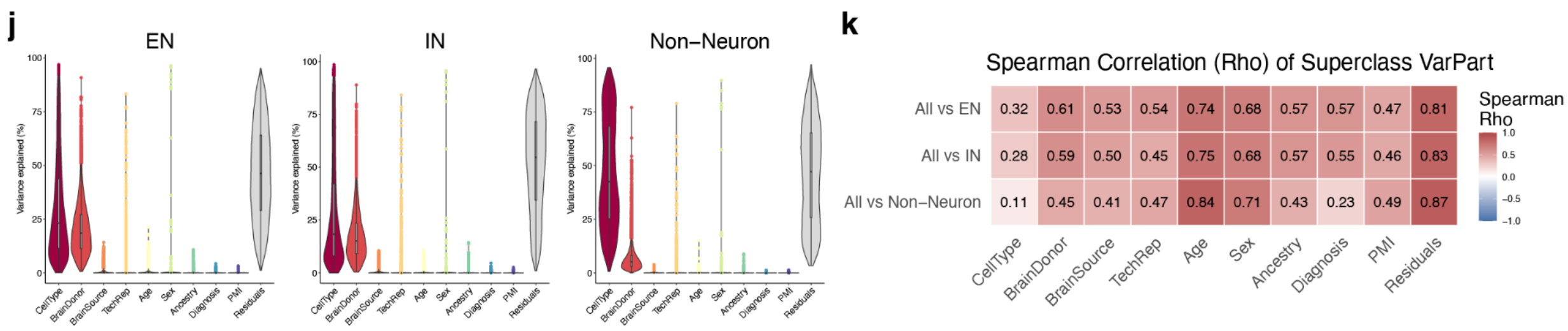<br><br>**Supplementary Fig. 6**. … **(j)** Variance partition analysis within three superclasses, namely, Excitatory Neurons (EN), Inhibitory Neurons (IN), and Non-Neurons (Non-Neuron). **(k)** Sensitivity analyses showing the robustness of variance explained estimates. Spearman correlation of variance explained estimates between all subclasses model (All) vs. EN, IN, and Non-Neuron superclass models. …

REF3-6. Comparison of compositional analysis with previous studies	
5. A number of analyses (esp. in Figs 4 and 6) compare cell type proportions between disease states, however from Supp. Fig. 2 and Supp Fig. 4 it appears that cell type proportions are different between brain banks. Cell type composition is highly sensitive to subtle variation tissue preservation, dissection, processing of nuclei, etc. In addition, cell type composition is known to change throughout aging, which is also variable across the tissue sources and disease states. These technical challenges make analyses of cell type composition very sensitive and possibly challenging to interpret. Notably, cell type composition has been extensively studied in both neuropsychiatric and neurodegenerative disorders by classical immunostaining approaches. It is concerning that some of the patterns identified in the current analysis do not line up with those previous findings. For instance, Pvalb-expression interneurons have reproducibly been shown to be reduced in SCZ, but at the subtype level they appear to be increased in Supp. Fig 4d. It would be important to clearly show how confounding variables contribute to these analyses. Even if these variables are included as covariate there may be artifacts. The authors should research and cite the previous literature and acknowledge both similarities and discrepancies with other techniques. There is also a need for replication in independent cohorts, especially ones where the cases and controls are better matched by age, source, and other confounding variables.	
Response	We thank the reviewer for this feedback. Interpreting results relating to differences in cellular composition based on disease status is challenging due to the nature of compositional data. Single nucleus RNA-seq captures the relative, but not absolute, abundance of each cell type. Moreover, for a given sample the cell fractions must sum to 100% by definition. These limitations mean that, for example, an increase in the composition of one cell type will cause the observed composition of the remaining cell types to decrease. The centered log ratio (CLR) transform we used in the crumblr method[32] is one way to account for the compositional bias in these data, although we acknowledge that all compositional analyses have this issue to some degree. We then performed statistical testing on CLR transformed count ratio data using precision-weighted linear mixed models incorporating random effects

	for complex study designs. We estimated changes in cell type composition driven by disease after regressing out the effects of age and sex. We also accounted for sequencing batch effects and inter-site variability by performing analyses separately within each brain source, followed by fixed-effect meta-analysis for diseases represented by two brain sources. In addition, we applied batch-aware normalization and downstream corrections, as detailed in the **Methods**, to reduce potential artifacts from technical variation. As a result, we describe changes in cell subclass frequency, but don't claim that they are causal.<br><br>Regarding the specific example about the Parvalbumin (PVALB) expressing interneurons in SCZ, the observed differences may be attributed to age differences between prior studies and this one. Additionally, differences in disease chronicity—particularly the lower or undefined chronicity in earlier studies—are also likely contributing factors. While the reduction in markers like PVALB protein is a very robust and consistent finding[45–50], several studies have reported that the actual number or density of PVALB+ interneurons is not significantly reduced in the prefrontal cortex (PFC)[50,51]. Furthermore, a meta-analysis of parvalbumin interneurons in schizophrenia discovered significant variability between studies and discussed the methodological limitations[52]. Detecting PVALB+ neurons often relies on immunostaining for the PVALB protein itself. If PVALB protein expression is significantly reduced (as it is in SCZ), it can make the neuron harder to detect, potentially leading to an underestimation of cell number unless researchers use very sensitive methods or complementary markers. We also note that our single-nucleus approach allowed for subtype-level resolution, revealing changes in specific subclasses of PVALB+ interneurons. This granularity may uncover patterns not captured in bulk or immunohistochemistry-based studies.<br><br>Despite the inherent limitations of cell type composition analysis, we agree that replicating our findings with existing datasets is crucial. To address these concerns, we performed a replication of our compositional variation analyses using two additional AD associated single-cell datasets from the human prefrontal cortex: the ROSMAP[6] and the SEA-AD[7] studies. Overall, we found high concordance across different measures of AD pathology (CERAD score, Braak stage and ordinal dementia scale) (**Supplementary Figs. 10a-b**). Furthermore, we replicated our findings using a published MERFISH dataset[7], showing the observed change is robust to technology used (**Supplementary Fig. 10c**). These datasets had differing demographics and tissue handling protocols, further supporting the robustness of our findings across distinct sources and methodologies. We have updated the manuscript to include these replication results and discuss their implications.<br><br>These additional analyses strengthen the robustness of our conclusions and provide further support for the validity and reliability of our findings.
Excerpt	"... The variation in cell type composition reveals distinct patterns associated with AD pathologies compared to normal aging (**Fig. 6a, Supplementary Fig. 9a, Supplementary Table 5**) and were consistent with previous studies[6,7] (**Supplementary Figs. 10a-b**). Furthermore, we replicated our findings using a published MERFISH dataset[7], showing the observed change is robust to technology used (**Supplementary Fig. 10c**)."

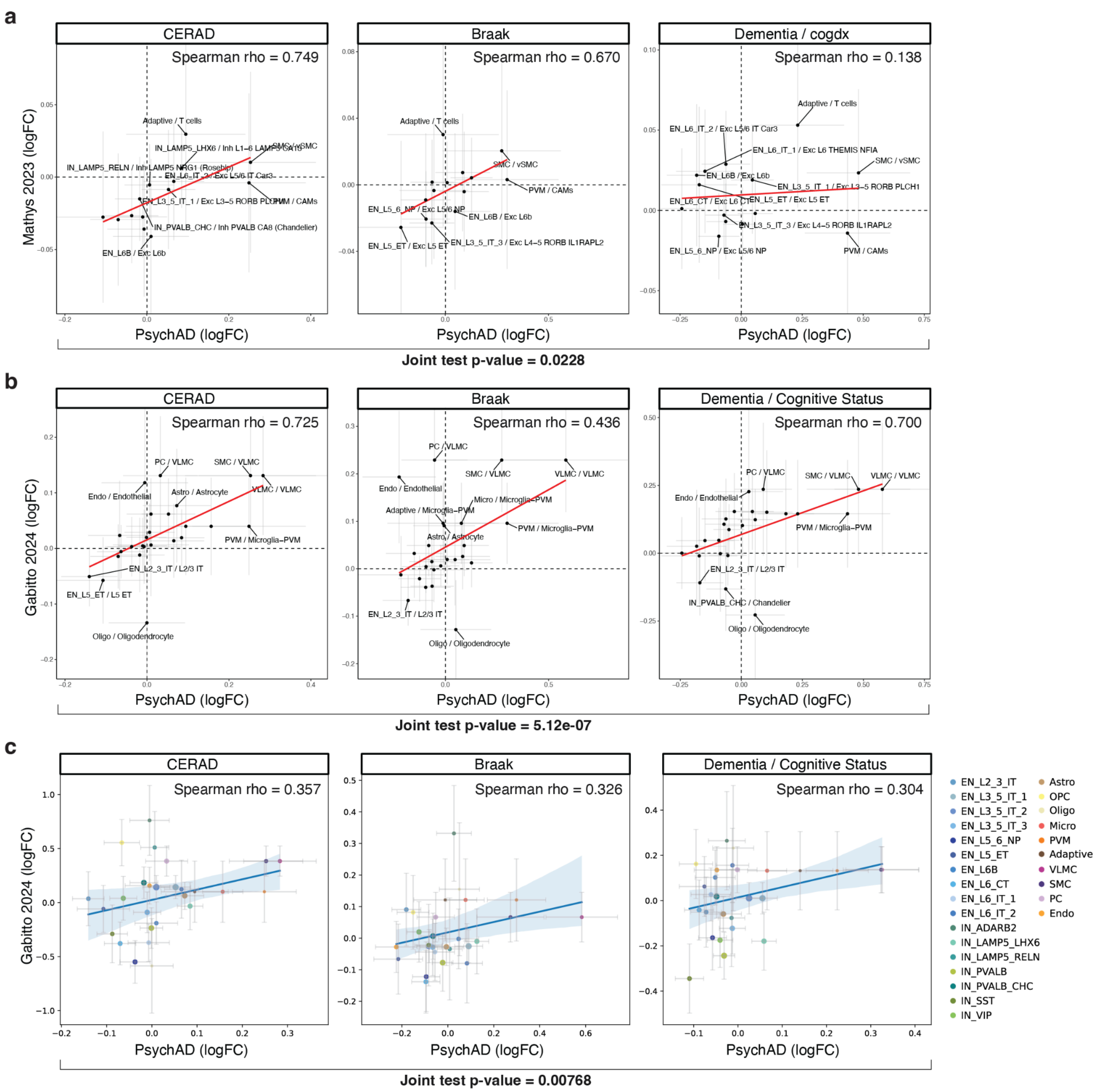

Supplementary Fig. 10. Comparison of compositional variation analysis with previous studies - DLPFC snRNA-seq data from **(a)** ROSMAP (Mathys et al. 2023)[6] and **(b)** SEA-AD (Gabitto et al. 2024)[7]. **(c)** Comparison of compositional variation analysis using the MERFISH spatial transcriptomics dataset included in the SEA-AD study[7]. Spearman correlation between this study (x-axis) and previous studies (y-axis) using 3 different measures of AD pathology (CERAD score, Braak staging, and ordinal Dementia scale). ROSMAP used clinical diagnoses of AD, MCI, and NCI, while SEA-AD used clinical diagnosis of dementia as a measure of cognitive decline. Further details regarding compositional analysis of the MERFISH data and joint statistical testing can be found in the **Methods** section.

REF3-7. Shared gene expression signature

6. Line 274. The analysis of differential expression with Dreamlet seems well done. However, I found it very difficult to interpret the shared and disease-specific signatures from Mashr (in the first half of Fig 5 and Supp Fig 5) because the authors do not provide information about the individual genes that contribute to these patterns. Is it possible to provide information about the gene loading

in each of the signatures and to show expression patterns for the individual genes that comprise them? It would be easy for these patterns (especially shared signatures) to arise from technical issues as well as disease processes.

Response

We thank the reviewer for their positive feedback on the differential expression analysis. To address the concern raised, we have updated **Supplementary Table 4** to include the posterior probability that each gene exhibits a cross-disorder shared effect, in a cell-type-specific manner. These posterior probabilities serve as a proxy for gene loadings, indicating the extent to which gene expression changes are shared across disorders.

To further support the robustness of our shared signatures, we conducted a pairwise correlation analysis of disease effects using either shared or disease-specific genes (**Supplementary Fig. 8a**). The results demonstrate that the shared signatures effectively capture common molecular features across traits, as evidenced by higher Spearman correlations among diseases.

Excerpt

"... Using a composite testing approach, we used these results to estimate posterior probabilities that each effect is specific to a trait or shared across traits. We then evaluated, in a cell-type-specific manner, cross-disease sharing and decomposed the total disease effects into shared and disease-specific components (**Supplementary Table 4**). We demonstrate that cross-disorder signatures have a higher degree of disease-effect sharing (**Supplementary Fig. 8a**) and encompass crucial transcriptional processes, such as mRNA splicing and processing, as well as protein localization to mitochondria (**Fig. 5a, Supplementary Fig. 8b**)."

Supplementary Table 4. Differentially expressed genes across 8 cross-disorder traits. Columns named crossDis_PP and crossDis_cat indicate posterior probability of each gene having cross-disorder shared effect and category of shared or non-shared used for the analysis.

a

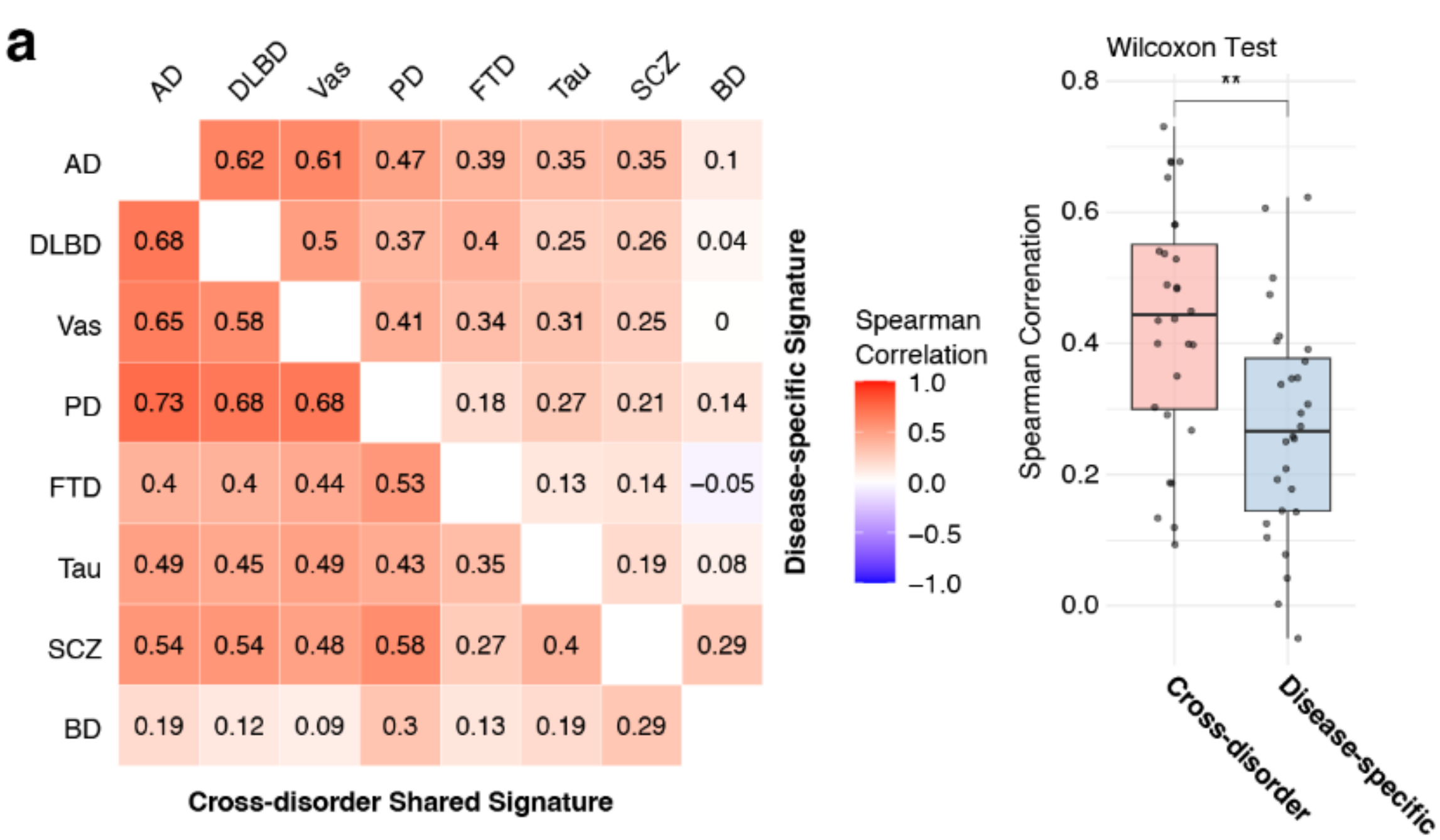

Supplementary Fig. 8. Cross-disorder shared and disease-specific gene expression signature. **(a)** Heatmap of pairwise Spearman correlation between diseases using cross-disorder shared genes (squares below the diagonal) and disease-specific genes (squares above the diagonal). Wilcoxon rank-sum test between two gene groups. …

REF3-8. Comparison of compositional analysis to previous literature	
7. Line 321 / Fig. 6a. Cell type composition has been extensively studied in AD across stages of disease. The authors' findings seem reasonably consistent with some of this prior knowledge (e.g., dysfunction of vascular smooth muscle cells). However, it is not clear what this analysis adds beyond what has been extensively shown by others using techniques that are probably more reliable for understanding cell type proportions. At the very least, the authors should cite the prior literature. For findings that may be more novel such as a potential vulnerability of EN subclasses specifically in AD patients experiencing weight loss it would be important to validate this finding by some independent techniques, or at least in independent cohorts with transcriptomic data.	
Response	We appreciate the reviewer's insightful feedback. We would like to highlight that a key strength of our study lies in the cross-disease comparison of cellular composition, which remains largely unexplored in the current literature.<br><br>In response to the comment, we extended our compositional variation analyses to include two additional AD-associated snRNA-seq datasets from the human prefrontal cortex: the ROSMAP[6] and the SEA-AD[7] studies and referenced relevant prior studies where appropriate (see **REF3-6**).<br><br>Additionally, we investigated the potential vulnerability of deep-layer excitatory neuron (EN) subclasses in AD patients experiencing psychomotor agitation (PMA) and weight loss. Although we were unable to find any other snRNA-seq datasets with detailed neuropsychiatric symptom annotations (including weight loss), we were able to obtain and leverage the body mass index (BMI) data for the RADC cohort. Using this information, we created a binary weight-loss indicator and performed compositional analyses in strictly defined AD cases. These analyses revealed a consistent enrichment of deep-layer excitatory neurons in AD patients with weight loss (**Supplementary Figs. 9c-d**), further supporting our original findings.
Excerpt	"... To evaluate the cell types associated with NPS prevalence, we applied compositional variation analysis to three categories of NPS based on co-occurrence estimates. Notably, using age-matched groups of AD patients with or without NPS (**Supplementary Fig. 9b**), we found AD patients experiencing weight loss and PMA have an increased ratio of excitatory neurons, especially deep layer neurons in L5-6 (**Fig. 6a, Supplementary Figs. 9a,c-d**). Our results corroborate previous findings[53] that changes in deeper PFC layers (L5-6) are associated with certain types of NPS."

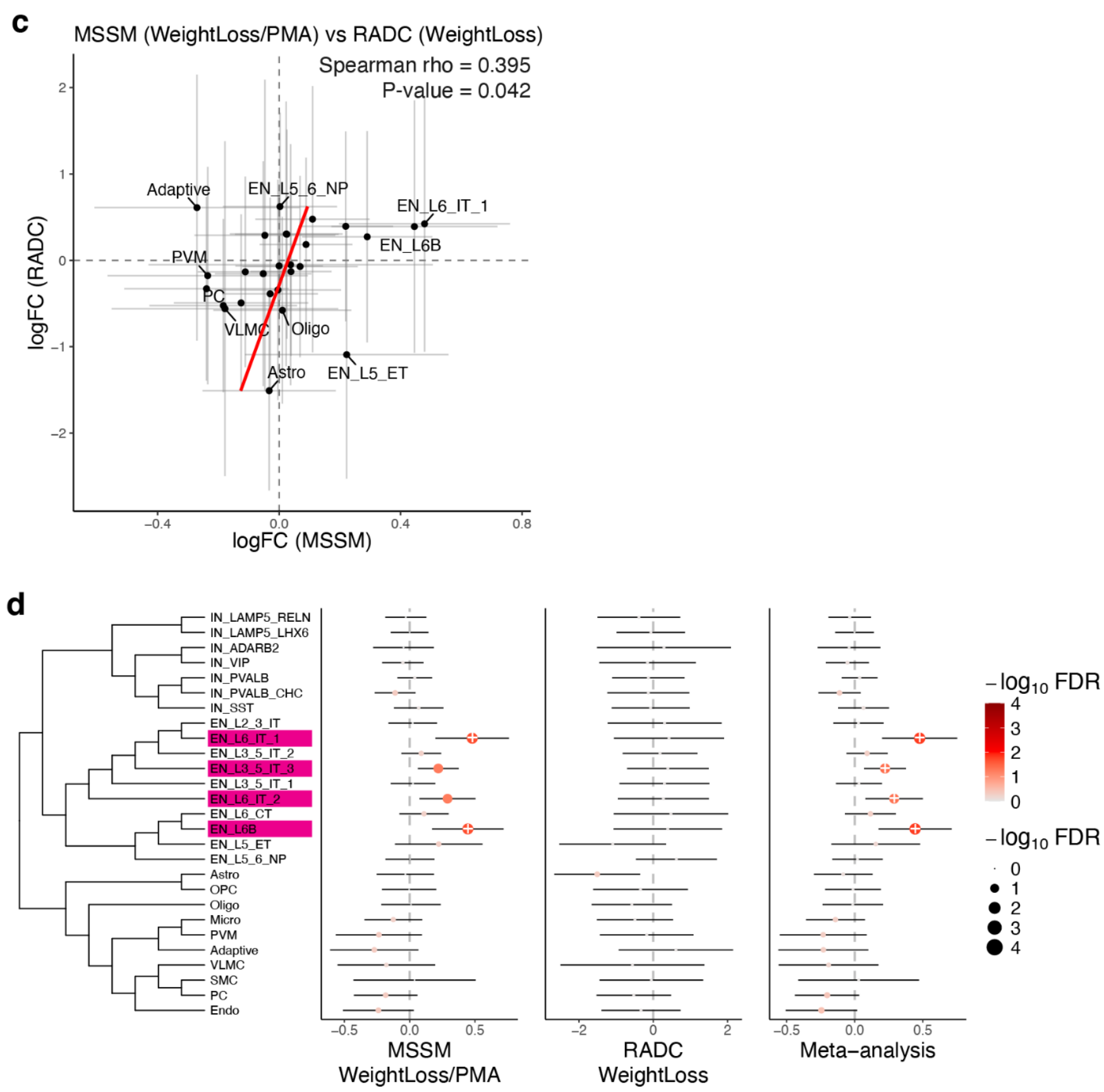

Supplementary Fig. 9. Compositional variation across AD neuropathology. **… (c)** Comparison of compositional variation between MSSM and RADC for WeightLoss phenotype. Due to lack of exact WeightLoss/PMA phenotype in RADC, WeightLoss was binarized from body mass index (BMI) within AD cases. **(d)** Fixed-effect meta-analysis between MSSM WeightLoss/PMA and RADC WeightLoss.

REF3-9. Mediation analysis

8. Mediation models are interesting, but as noted by the authors this analysis largely overlaps previous analyses. To what extent do the mediation analyses here corroborate or disagree with previous findings? My impression, unfortunately, is that previous attempts were more nuanced and provided greater insight into the disease processes. How do the mediation pathways described here compare to alternate pathways to a AD-related and aging-related states as modeled by Green et al. 2024 in Nature?

Response

We appreciate the reviewer's insightful comments and positive feedback regarding the mediation analysis. We acknowledge the reviewer's point that this analysis builds upon previous work, and we agree that comparing our findings to existing literature, particularly Green et al. (2024), is crucial for contextualizing our results and highlighting novel contributions. While our mediation analysis was inspired by previous studies, there are several key methodological and conceptual differences that lead to distinct, and we believe, complementary findings:

1. Anchoring Genetic Risk: Green et al. (2024) utilized the APOE ε4 genotype as their primary anchor for genetic risk. In contrast, our study employed a polygenic risk score (PRS) derived from the latest AD GWAS by Bellenguez et al. (2022). While the APOE ε4 allele remains by far the strongest single genetic risk factor for AD, it captures only a subset of the total heritable risk. Genetic risk is continuous and it has been shown that PRS improves prediction beyond APOE alone by capturing additional polygenic contributions, offering meaningful stratification even among APOE4-negative individuals[4,5].

We performed additional sensitivity analyses involving AD PRS with or without APOE locus and using PRS from other neurological diseases (see **REF1-6** for extended analyses). We observed negligible differences when APOE locus was removed from PRS, reassuring the robustness of the analysis (**Supplementary Table 13**). Furthermore, we observed non-significant mediation effects when PRS from different neurological diseases were used in the causal mediation analysis.

2. Cell Type Specificity and Breadth: Green et al. (2024) focused on four specific subpopulations: two microglia subtypes (Mic.12 and Mic.13), one astrocyte subtype (Ast.10), and one oligodendrocyte subtype (Oli.7). Our analysis, however, considered a broader range of cell types. We initially included all 14 cell subclasses that showed significant alterations in cell type composition in any of the AD contrasts ($FDR < 0.05$). From this wider set, we identified four cell subclasses (Microglia, VLMC, IN_SST, and IN_LAMP5_LHX6) exhibiting significant mediation effects ($P < 0.05$). This broader initial screen allows for the discovery of mediating roles for cell types that might be overlooked when focusing on a predetermined subset.

Overall, both our study and Green et al. (2024) identify Microglia as a significant mediator of AD risk. This corroborates the established importance of microglial dysfunction in AD pathogenesis and provides further support for the role of these cells in translating genetic risk into disease phenotypes. However, Green et al. (2024) emphasized the mediating effects of specific glial subtypes (Oligodendrocytes and Astrocytes). While our analysis does not preclude a role for these cell types, our broader approach, combined with the use of a PRS, highlighted the significant mediating effects of vascular cells (VLMC) and two subclasses of interneurons (IN_SST and IN_LAMP5_LHX6). These findings complement, rather than contradict, the findings of Green et al., suggesting that the increased power of PsychAD cohorts can reveal distinct, yet potentially interacting, cell types mediating AD risk.

Excerpt

"...

Treatment (IV)	Mediator (MV)	Outcome (DV)	Effects	Estimate	95% CI Lower	95% CI Upper	p-value	Significance	Note
PRS_AD	PLAQUE	Braak	ACME	0.0878	0.05	0.13	<2e-16	***	AD_2022_Bellenguez
PRS_AD	PLAQUE	Braak	ADE	0.0735	0.0296	0.12	0.0014	**	AD_2022_Bellenguez
PRS_AD	PLAQUE	Braak	Total Effect	0.1613	0.1045	0.22	<2e-16	***	AD_2022_Bellenguez
PRS_AD	PLAQUE	Braak	Prop. Mediated	0.5444	0.3633	0.76	<2e-16	***	AD_2022_Bellenguez
PRS_AD_wo_APOE	PLAQUE	Braak	ACME	0.1342	0.0866	0.18	<2e-16	***	AD_2022_Bellenguez without APOE locus
PRS_AD_wo_APOE	PLAQUE	Braak	ADE	0.0907	0.0334	0.15	0.0018	**	AD_2022_Bellenguez without APOE locus
PRS_AD_wo_APOE	PLAQUE	Braak	Total Effect	0.2249	0.1525	0.3	<2e-16	***	AD_2022_Bellenguez without APOE locus
PRS_AD_wo_APOE	PLAQUE	Braak	Prop. Mediated	0.5958	0.4276	0.81	<2e-16	***	AD_2022_Bellenguez without APOE locus
PRS_PD	PLAQUE	Braak	ACME	-0.0418	-0.0897	0.01	0.085	.	PD_2019_Nalls
PRS_PD	PLAQUE	Braak	ADE	0.014	-0.0397	0.07	0.612		PD_2019_Nalls
PRS_PD	PLAQUE	Braak	Total Effect	-0.0278	-0.099	0.04	0.45		PD_2019_Nalls
PRS_PD	PLAQUE	Braak	Prop. Mediated	0.9626	-8.6891	10.14	0.399		PD_2019_Nalls
PRS_ALS	PLAQUE	Braak	ACME	0.021	-0.0198	0.06	0.3		ALS_2021_vanRheenen
PRS_ALS	PLAQUE	Braak	ADE	0.0284	-0.0173	0.07	0.22		ALS_2021_vanRheenen
PRS_ALS	PLAQUE	Braak	Total Effect	0.0495	-0.0105	0.11	0.11		ALS_2021_vanRheenen
PRS_ALS	PLAQUE	Braak	Prop. Mediated	0.4228	-1.6198	2.22	0.28		ALS_2021_vanRheenen
PRS_MS	PLAQUE	Braak	ACME	0.00877	-0.031	0.05	0.68		MS_2019_IMSGC
PRS_MS	PLAQUE	Braak	ADE	-0.02275	-0.06859	0.02	0.33		MS_2019_IMSGC
PRS_MS	PLAQUE	Braak	Total Effect	-0.01399	-0.07371	0.05	0.65		MS_2019_IMSGC
PRS_MS	PLAQUE	Braak	Prop. Mediated	0.21086	-9.73452	8.34	0.78		MS_2019_IMSGC
PRS_SCZ	PLAQUE	Braak	ACME	-0.00548	-0.04663	0.04	0.79		SCZ_2022_Trubetskoy
PRS_SCZ	PLAQUE	Braak	ADE	0.01654	-0.03092	0.06	0.49		SCZ_2022_Trubetskoy
PRS_SCZ	PLAQUE	Braak	Total Effect	0.01106	-0.05093	0.07	0.73		SCZ_2022_Trubetskoy
PRS_SCZ	PLAQUE	Braak	Prop. Mediated	0.31545	-6.80958	7.49	0.66		SCZ_2022_Trubetskoy
PRS_BD	PLAQUE	Braak	ACME	-0.00184	-0.04155	0.04	0.92		BD_2021_Mullins
PRS_BD	PLAQUE	Braak	ADE	0.00309	-0.04126	0.05	0.88		BD_2021_Mullins
PRS_BD	PLAQUE	Braak	Total Effect	0.00124	-0.05802	0.06	0.97		BD_2021_Mullins
PRS_BD	PLAQUE	Braak	Prop. Mediated	0.44031	-5.62087	7.29	0.55		BD_2021_Mullins
PRS_ASD	PLAQUE	Braak	ACME	0.00407	-0.0387	0.05	0.84		ASD_2019_Grove
PRS_ASD	PLAQUE	Braak	ADE	0.02864	-0.0184	0.08	0.23		ASD_2019_Grove
PRS_ASD	PLAQUE	Braak	Total Effect	0.03271	-0.02994	0.1	0.31		ASD_2019_Grove
PRS_ASD	PLAQUE	Braak	Prop. Mediated	0.25062	-4.19067	5.1	0.62		ASD_2019_Grove

Supplementary Table 13. Causal mediation analysis involving PRS, cell type composition, pathology, and dementia status. Estimates of direct and mediating effects and their statistical significance.

…"

REF3-10. Comparison of cell type-specific DEGs in AD with previous studies	
9. Line 360. Descriptions of cell type-specific DEGs in AD are extremely superficial and add little to what is already known. For instance, the transcriptional states of microglia in AD have been extensively studied, and it is known that distinct microglial states contribute to AD in different ways at different stages of the disease. For instance, lysosomal/DAM/TREM2 microglia appear to be enriched at early stages and may be protective, whereas pro-inflammatory subtypes with neurotoxic effects prevail later. Yet the authors merely state that there were many DEGs in microglia, give the names of a few that overlap previous studies, and do not at all grapple with the known complexities (line 367). Similarly, a statement that the DEGs in neurons were enriched for synaptic functions is too vague to be informative (line 370).	
Response	We thank the reviewer for highlighting the need for a more nuanced interpretation of the cell type-specific findings in AD, particularly with respect to microglia and neuronal transcriptional states.<br><br>In response, we have expanded our nonlinear trajectory analysis (**Fig. 7**) and discussion to better contextualize our findings within existing hypotheses of AD pathogenesis and to uncover novel disease triggers. Specifically, we have further analyzed the development of the microglia lipid droplet phenotype (see **Excerpt 1a**) and increasing lipid homeostasis stress (see **Excerpt 1b**), detailed the two distinct phases of the immune response (see **Excerpt 1c**), and introduced new findings on a key pathway regulating tau phosphorylation that selectively decreases in neurons (see **Excerpt 1d**). Additionally, as in the original manuscript, we continue to reference how our pathway enrichment analysis across cell classes (**Fig. 7e**) aligns with and expands upon findings in the existing literature.<br><br>To further strengthen the robustness and reliability of our findings, we performed an independent replication of our differentially expressed gene (DEG) analysis using two additional single-cell datasets derived from the human prefrontal cortex in AD: the ROSMAP study[6] and the SEA-AD study[7]. These analyses supported the key disease-associated expression changes reported in the main dataset and bolster confidence in the reproducibility of our observations (**Supplementary Figs. 12a-e**, more details in **REF1-7**, see **Excerpt 2**).
Excerpt	1. Results section titled **Nonlinear dynamics of the AD pathological trajectory**:<br><br>a. "In contrast, several early increasing genes, such as ACSL1, DPYD, and CD163 were recently implicated in a pathogenic lipid-droplet accumulation phenotype in APOE4/4 AD patients (another implicated gene, NAMPT, ranked 134th; **Supplementary Fig. 26a** and **Supplementary Table 9**)[36]."<br><br>b. "Lipid homeostasis and efflux pathways, many involving known AD risk genes, increased throughout disease progression, particularly in later stages (**Supplementary Figs. 25-26b)**, supporting the hypothesis that microglia develop a lipid-droplet accumulation phenotype that may exacerbate disease progression[34–36]"<br><br>c. "To further resolve the immune response at a finer time resolution, we performed pathway enrichment using a sliding window across disease pseudotime (**Supplementary Fig. 25).** Our results suggest two distinct immune phases[33]: an initial innate response, followed by a more adaptive and monocyte-driven response, along with increased cytokine production."<br><br>d. "One particularly notable example is the *negative regulation of peptidyl-threonine phosphorylation* (**Supplementary Figs. 22-23**), which has been implicated in tau phosphorylation[54]. Mean expression of this pathway selectively decreases early in both neural cell types and includes the risk gene *SPRED2*[55]"<br><br>2. Results section titled **Transcriptomic variation with AD pathology**:<br><br>"... In general, DEGs had high concordance across different AD pathology variables, consistent with previous reports[6], and the disease signatures were highly concordant with previous studies[6,7] (**Supplementary Figs. 12a-e**). ..."

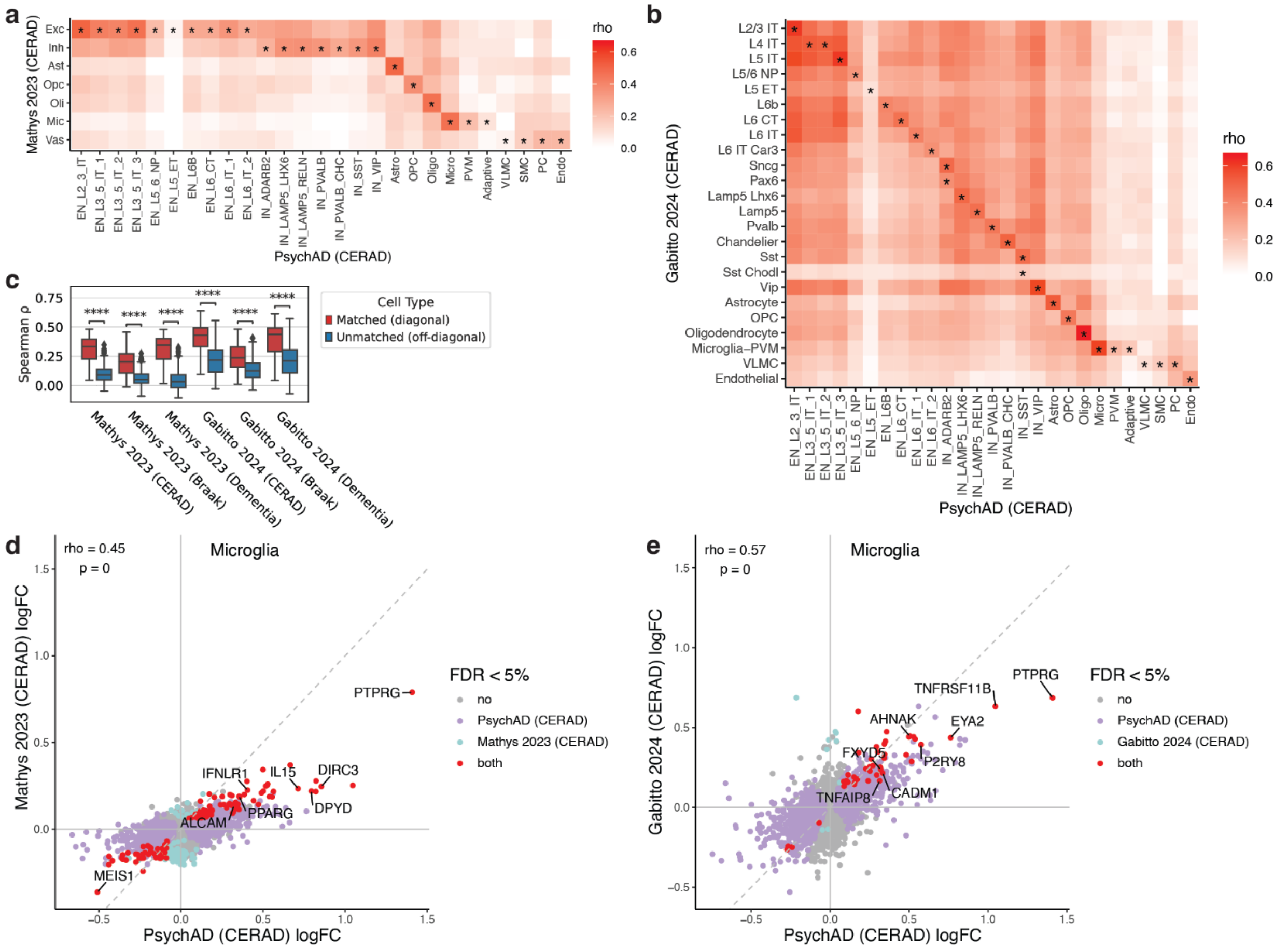

Supplementary Fig. 12. (a) Comparison of differential expression signatures with previous studies – ROSMAP (Mathys et al. 2023)[6] and SEA-AD (Gabitto et al. 2024)[7]. Pairwise Spearman correlation between PsychAD subclass and ROSMAP Major Cell Type. CERAD as a measure of disease severity. Asterisks indicate the best matching cell type based on **Supplementary Table 11**. **(b)** Pairwise Spearman correlation between PsychAD subclass and SEA-AD subclass. CERAD as a measure of disease severity. Asterisks indicate the best matching cell type based on **Supplementary Table 11**. **(c)** Comparison of various disease signatures (CERAD, Braak, Dementia) between matched (diagonal, asterisks) and unmatched (off-diagonal) cell types. Mann–Whitney U test. ****: p ≤ 1.0e-4. **(d)** Comparison of differentially expressed genes between PsychAD Microglia and ROSMAP Mic using CERAD as a measure of disease severity. Spearman correlation (rho) shown. **(e)** Comparison of differentially expressed genes between PsychAD Microglia and SEA-AD Microglia-PVM using CERAD as a measure of disease severity. Spearman correlation (rho) shown.

REF3-11. Reproducibility in other snRNA-seq datasets

10. There are no replication analyses to determine whether the patterns described in this cohort are reproducible in other snRNA-seq datasets, despite the fact that large cohorts are available for this purpose.

Response	In response, we have now performed a replication of our differentially expressed gene (see **REF3-10, Supplementary Figs. 12a-e**) and compositional variation analyses (see **REF3-6, Supplementary Figs.**

	**10a-c**) using two additional single-cell datasets focused on AD in the human prefrontal cortex: the ROSMAP study[6] and the SEA-AD study[7]. Overall, we successfully replicated our key findings and observed highly concordant disease signatures across these independent datasets. We have updated the manuscript to include these replication results and discuss their implications.
Excerpt	See **REF3-6, REF3-10**

REF3-12. Validation experiments	
11. There are no validation experiments using orthogonal techniques to verify predictions. As noted above, this may be especially important for cell type proportions, which can be estimated from standard techniques and are not always reliably detected from single-cell data.	
Response	We appreciate your insightful feedback and fully agree on the importance of using orthogonal approaches to validate our findings.<br><br>To strengthen the compositional analysis, we conducted RNAscope validation on brain tissue from 5 AD cases and 5 neurotypical controls (see **Methods**). We selected markers from three cell subclasses predicted to show opposing trends - CUX2 for L2-3 IT excitatory neurons, SST for SST^+ inhibitory interneurons, and BMP5 for VLMCs. The results confirmed a decline in $CUX2^+$ and SST^+ neurons and an increase in $BMP5^+$ VLMCs in AD brains (**Supplementary Figs. 9e–f**), supporting our single-nucleus transcriptomic findings.<br><br>Furthermore, as part of replication analyses of our compositional variation results (see **REF3-6, Supplementary Figs. 10a-c**), we additionally performed compositional variation analysis using the MERFISH dataset included in the recent SEA-AD publication[7] - a dataset produced by an independent group of researchers, using an orthogonal experimental method. We pre-processed the data to take into account the matching of the 2 studies' annotations, chose 3 AD-associated clinical traits that were annotated both in this external data and in our study (CERAD, Braak, and cognitive impairment), and performed compositional variation analysis with relation to each trait using crumblr (see **Methods**). A summary of the results for each trait, as well as a joint concordance test for all traits across the 2 studies, can be found in **Supplementary Fig. 10c**.
Excerpt	"... Notably, changes more pronounced in AD, such as the increase in $LAMP5^+LHX6^+$ inhibitory neurons (IN_LAMP5_LHX6) and the loss of SST^+ inhibitory neurons (IN_SST) with higher CERAD scores, indicates a potential association between this neuronal subtype and Aβ pathology. Additionally, the loss of L2-3 IT neurons in AD (based on case-control and CERAD comparisons), which is not evident in normal aging, suggests a specific vulnerability for this neuronal subtype to AD. We then experimentally validated the compositional shifts using RNAscope (**Methods**), confirming the decline of L2-3 IT excitatory and SST^+ inhibitory neurons and the prevalence of VLMCs in AD (**Supplementary Figs. 9e-f**). Taken together, our findings highlight the cell-type-specificity of AD vulnerability."

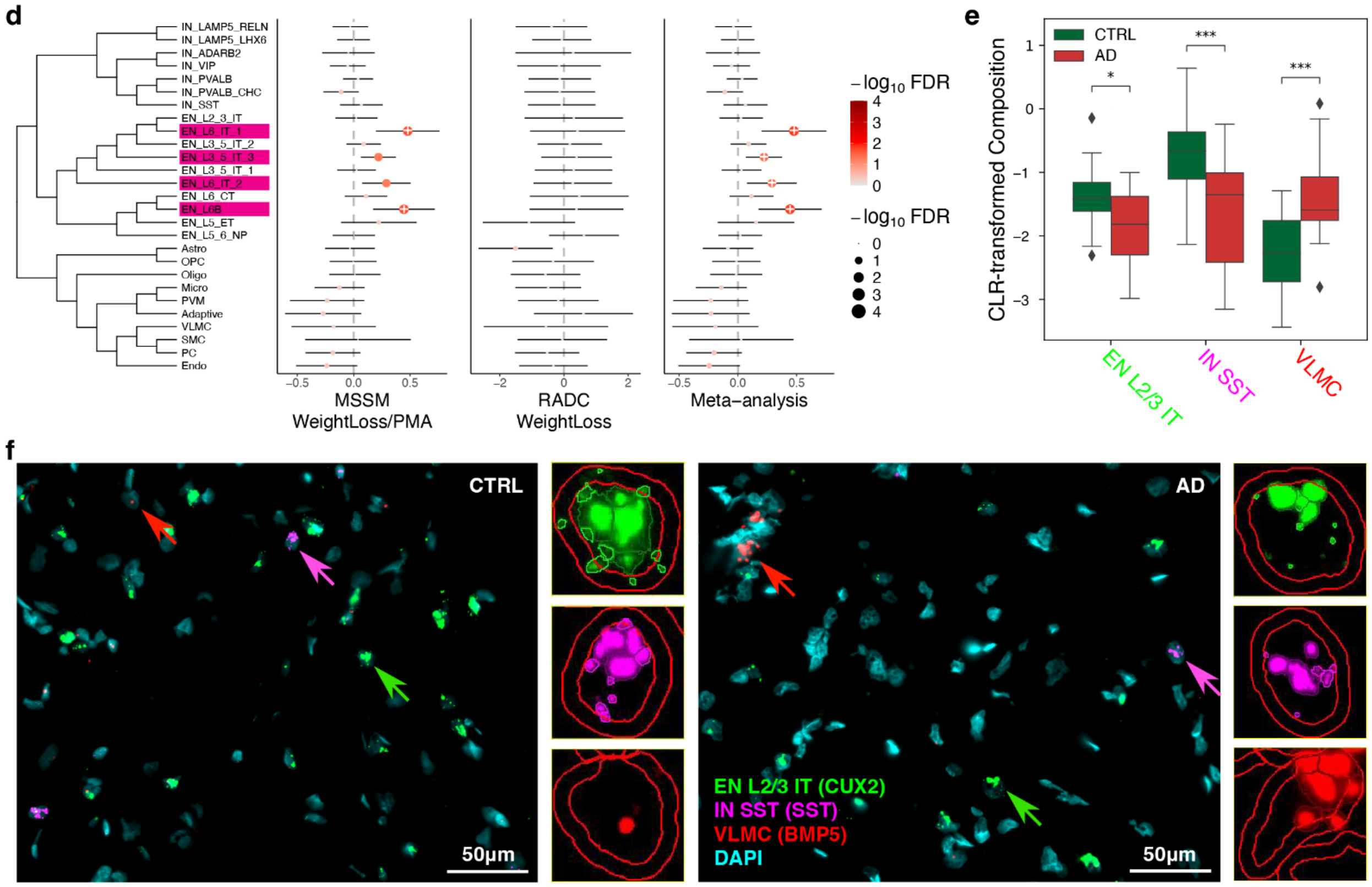

Supplementary Fig. 9. Compositional variation across AD neuropathology. **… (e)** Validation of compositional changes using RNAscope (see **Methods**). Cell counts were quantified based on specific markers - CUX2 (L2-3 IT neurons), SST (somatostatin interneurons), and BMP5 (VLMCs). Cell fractions were transformed using the centered log-ratio (CLR) method. Statistical differences between AD cases and controls (n = 10) were assessed using the Mann–Whitney–Wilcoxon test with p-value <= 0.05 *, <= 0.01 **, <= 0.001 ***. **(f)** Representative immunofluorescence images comparing AD and neurotypical control dissections.

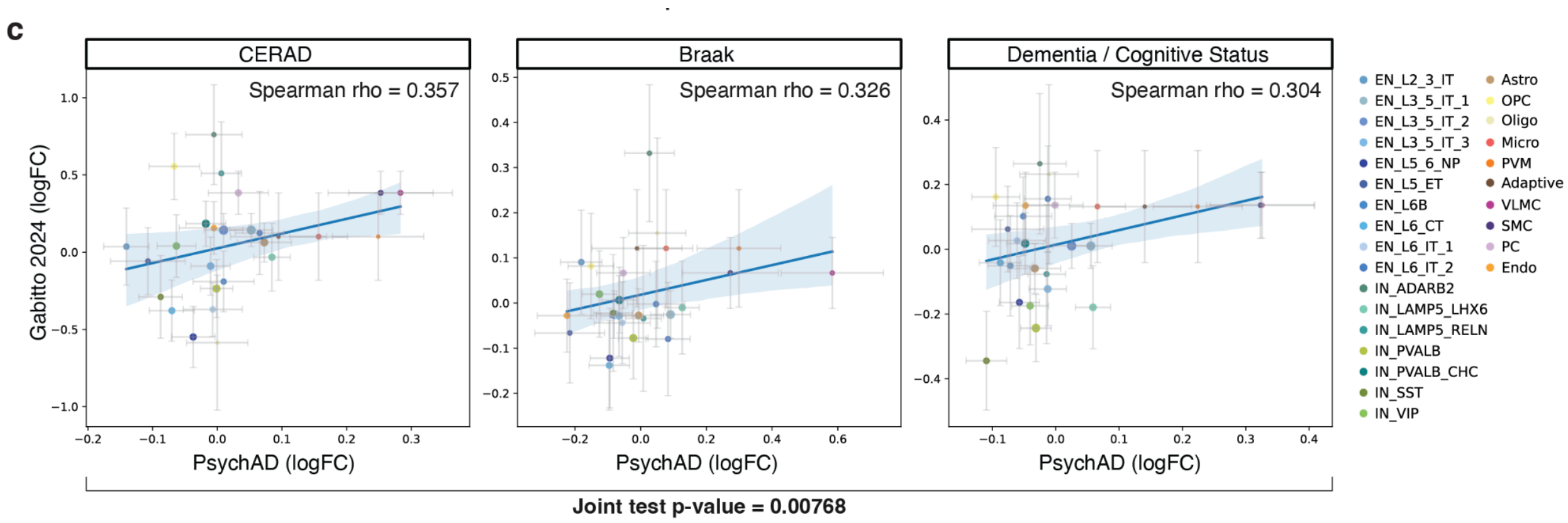

Supplementary Fig. 10. Comparison of compositional variation analysis with previous studies - DLPFC snRNA-seq data … **(c)** Comparison of compositional variation analysis using the MERFISH spatial transcriptomics dataset included in the SEA-AD study[7]. Spearman correlation between this study (x-axis) and previous studies (y-axis) using 3 different measures of AD pathology (CERAD score, Braak staging, and ordinal Dementia scale). ROSMAP used clinical diagnoses of AD, MCI, and NCI, while SEA-AD used clinical diagnosis of dementia as a measure of cognitive decline. Further details regarding compositional analysis of the MERFISH data and joint statistical testing can be found in the **Methods** section.

Validation of compositional variation using RNAscope

Tissue selection. We selected 5 AD and 5 neurotypical donors, matched for age and gender, and their DLPFC tissue blocks were obtained from the Mount Sinai Neuropathology Brain Bank and Research CoRE. **Probe design**.

We focused on 4 subclasses that showed robust variation in AD (Fig. 6a). Cell type markers were selected based on gene expression level and specificity (Fig. 2d, Supplementary Fig. 3i). The following probes were used: CUX2 (RNAscope™ Probe Hs-CUX2, Cat. # 425581) for EN_L2_3_IT, SST (RNAscope™ Probe Hs-SST-C2, Cat. # 310591-C2) for IN_SST, BMP5 (RNAscope™ Probe Hs-BMP5-C3, Cat. # 472461-C3) for VLMC, and MYOCD (RNAscope™ Probe Hs-MYOCD-C4, Cat. #416211-C4) and CASQ2 (RNAscope™ Probe Hs-CASQ2-C3, Cat. # 447111-C3) for SMC. **Cryosectioning**. Before sectioning, frozen brain tissue blocks were allowed to equilibrate to the cryostat (CryoStar NX50, Thermo Fisher) chamber temperature. Tissue blocks were mounted to chuck with OCT (Tissue-Tek® O.C.T. Compound, 4583, Sakura Finetek USA). After trimming, ROIs with high quality and intact grey and white matter were selected and scored with razor blades. Sections were cut at 16 μm thickness and collected onto the slides by gently touching the sections with the slides and two sections were collected per donor. Sections were further adhered to the slides by drying at room temperature before sealed in slide mailers (Fisherbrand 5-Place Slide Mailer, Fisher) and stored in -80 °C until RNAscope assay. **RNAscope assay and True Black treatment pipeline**. Sample preparation was carried out with ACD RNAscope Multiplex Fluorescent v2 for Fresh Frozen tissues protocol (UM 323100/Rev B) with an extended C2 TSA dye incubation of 45 minutes (determined based on pilot experiment testing). Sections were fixed in freshly made 10% Formalin (Formaldehyde solution, F8775-25mL, Millipore Sigma) in 1X PBS at 4°C for 1 hour, after which sections were rinsed twice with 1X PBS. Sections were dehydrated by placing the slides into a series of diluted ethanol and finally 100% ethanol. Before the creation of hydrophobic barriers, slides were air dried, and thick hydrophobic barriers were drawn around each section with the hydrophobic barrier pen (ImmEDG Hydrophobic Barrier Pen, NC9545623, Vector Laboratories). After the barriers were dried, hydrogen peroxide was applied to the sections and incubated for 10 min at room temperature, following the removal of the hydrogen peroxide, sections were washed twice with distilled water. Sections were then treated with Protease IV for 30 min at room temperature and washed with distilled water. To hybridize the gene specific probes, sections were incubated with diluted and mixed probes for 2 HRS at 40°C, then the sections were washed twice with 1X Wash Buffer 2 MIN at room temperature. Sections were either stored in 5X SSC overnight or immediately processed with AMP hybridization. During AMP hybridization, sections were incubated sequentially with AMP1, AMP2, and AMP3 for 30 minutes each at 40°C, with two washes using 1X Wash Buffer for 2 minutes between each incubation. Following the AMP steps, fluorescent signal development was performed by incubating with HRP-C1 for 15 minutes at 40°C. After two 2-minute washes with 1X Wash Buffer, a diluted fluorophore was added and incubated for 30 minutes at 40°C, followed by two 2-minute washes with 1X Wash Buffer. After the washes, HRP blocker is applied for 15 minutes at 40°C, followed by two final 2-minute washes with 1X Wash Buffer. The same procedure was repeated for HRP-C2 and HRP-C3, each with distinct fluorophores. Nuclei were counterstained with DAPI for 30 seconds, and washed with 1X Wash Buffer. After the RNAscope assay, samples underwent a TrueBlack (20X TrueBlack reagent, 23007, biotium) treatment following the biotium TrueBlack Lipofuscin Autofluorescence Quencher Protocol 2: Post-treatment with TrueBlack protocol (PSF006). Buffers on the sections were carefully removed, and 1X TrueBlack in 70% ethanol was applied to completely cover the tissue sections. After 30 seconds of incubation, sections were washed with PBS for three times. Coverslip mounting was performed with ProLong Gold Antifade Mountant (P36930, Thermo Fisher). **Imaging**. RNAscope results were acquired with EVOS M7000 at 40X (OLY XAPO 40X NA0.95/WD0.18, AMEP4907, Thermo Fisher). For each section, 2-3 ROIs were captured, the selected ROIs covered the entire grey matter and approximately two-thirds of the white matter, with good tissue quality and well represented the whole section. In total four channels were imaged for nuclei and the C1-3 of the RNAscope signal, with DAPI, GFP, RFP, and CY5 fluorophore cubes. At 40X, the ROIs contain 100–200 single field of views, which were captured with the scan area function of the EVOS M7000. The scan was performed with serpentine horizontal scan pattern and quick scan model with less overlapping areas. Auto focus on every field of the DAPI channel was chosen to gain proper focus. Single field images were saved as 16-bit TIF files. **Image processing and result analysis**. Individual images were stitched into the whole ROIs and four channel images of the same ROI were stacked together with in house Fiji imageJ macro using the Grid/Collection Stitching Plugin. For the DAPI channel, Grid: snake by rows function was used, and compute_overlap and subpixel_accuracy options were chosen. After the DAPI channel images were stitched, the resulting '`TileConfiguration.registered.txt`' files were used as guidance to stitch images of the other channels to make the resulting ROI images having the same dimension. Whole ROI images of four channels were stacked with "`Images to Stack`" and "`Stack to Hyperstack`" functions of Fiji imageJ. To calculate the gene expression level at single cell level, all stacked whole ROI images were further analyzed in quPATH, by counting the number of RNAscope punctate signals inside segmented cell borders. quPATH annotation was drawn to cover the whole ROI whilst avoiding areas of poor tissue quality or high fluorescent background. The cells in the ROIs were segmented based on DAPI nuclei signals with the cell detection function of the quPATH. Afterward, RNAscope punctate signals were detected by the subcellular detection function of the quPATH. Results were saved as csv files with the `saveDetectionMeasurements` function of the quPATH. To compare the difference of cell composition

	between the AD and control donors, an in-house python script was developed to quantify the cells of a certain cell type. The threshold of a positive cell is determined by evaluating the distribution of the cell and also by inspecting the images. Cell type was determined based on the number of clusters detected for each channel (2 for CUX2, 0 for SST and BMP5). We observed a higher sensitivity for CUX2, so we used a higher minimum threshold to compensate. For each sample, cell counts and fraction was calculated. Cell fractions were transformed using the centered log-ratio (CLR) method, which divides each part of the composition by the geometric mean of all parts and then takes the logarithm of the resulting ratio. Statistical differences between AD cases and controls (n = 10) were assessed using the Mann–Whitney–Wilcoxon test.

REF3-13. Discussion	
12. The Discussion is very minimal. A missed opportunity to more fully elucidate some of the connections of this work to previous studies.	
Response	In response, we have amended the discussion in the revision to elaborate our findings with previous studies and discuss the limitations and caveats of the study (see **Excerpt**).
Excerpt	"... Among our findings is the observation that the brain vascular system is intricately linked to immune dysfunction in AD. In general, we saw a relative increase in vascular cell types in most NDDs (**Supplementary Figs. 6e,f**) and demonstrate that levels of VLMCs are particularly elevated in AD among individuals with more severe cognitive impairment (**Figs. 6a,c**). It has previously been shown that the meningeal lymphatic system plays multiple roles in the brain, including waste removal[56–58] and the adaptive immune response[59,60]. Given the role of VLMCs in these processes[61–64], their contribution to disease warrants further investigation.<br>Our analysis of the pathological trajectory of AD aligns with various proposed hypotheses[65–70], particularly for pathways affected in the earliest stages of the disease. Neuronal, immune, and vascular cells exhibit distinct vulnerabilities, with shared alterations across correlated pathways. In neurons, we observed a decrease in metabolic functions (cyclic nucleotide catabolic process, cytoplasmic translation, and ATP synthesis) that have been closely linked to synaptic dysfunction and cognitive decline[65]. On the other hand, the immune response appears to be generally protective, where an early innate immune activation[66] followed by an adaptive immune response[71–73] was associated with resilience to dementia. In particular, monocyte differentiation appeared to play a protective role during both early and late disease stages, consistent with the idea that monocyte-derived macrophages serve as reinforcements when the immune system is under sustained stress[74]. The one exception was the response of immune, mural, and endothelial cell classes to IL-17, which was damaging in the early stages of AD. IL-17 has been associated with cognitive decline[75,76] and disruption of the blood-brain barrier[77], and blocking IL-17 function has been shown to mitigate cognitive defects in murine models of AD[75,76]. In addition, chaperones have been closely associated with a number of disease related processes, including tau misfolding and aggregation, and neurotoxicity[67–70,78,79], however, their precise roles in AD pathogenesis remains unclear. Our results suggest that chaperones can play opposing roles depending on the cellular context - damaging for glia, immune and endothelial cells but possibly protective for neurons. Lastly, upregulation of endothelial cell stress, differentiation and adhesion/junction pathways were associated with cognitive decline, consistent with the idea that blood-brain barrier dysfunction[80], a known hallmark of dementia, may trigger compensatory increases in these pathways.<br>Taken together, these insights deepen our understanding of AD pathogenesis and implicate cellular responses that warrant further investigation. While the human brain comprises numerous regions with distinct functions, in this study we limited our analysis to the DLPFC. Future studies that include additional brain regions will provide insights into a broader range of phenotypes and offer a more holistic view of the molecular foundations of brain function in both health and disease. Moreover, more detailed spatial analyses, including whole-cell transcriptomics, could further elucidate the physiological contexts that contribute to disease. Analyzing larger numbers of cells may also uncover rarer disease-associated cell types or subtypes, while integrating additional omics modalities (e.g. proteomics or epigenomics) will provide deeper molecular insights. Overall, the PsychAD single-cell disease atlas serves as a unique and foundational resource to further our understanding of population-level disease-associated transcriptomic variation in the human brain."

REF3-14. Deeper evaluation of findings with previous literature	
13. Overall, the manuscript is surprisingly superficial and lacks deep insights into the pathology of the diseases (even for AD). Given the breadth of the datasets, this represents a missed opportunity. The manuscript as presented represents primarily a high-level analysis that basically notes similarities among neurodegenerative and neuropsychiatric, but the richness of the dataset should allow the authors to elucidate these relationships in much more nuanced ways. For instance, they could reconstruct cell-type specific gene regulatory programs that are disease specific, and provide insight into targeted therapies that can be employed in specific disease contexts - ideally in an age/ or disease stage specific manner. There are many connections to the existing/previous literature that are not drawn out. A deeper evaluation of the prior knowledge could aid in producing a more unique analysis.	
Response	We thank the reviewer for this insightful comment and agree that our dataset holds potential for many in-depth mechanistic analyses. Our primary goal for this capstone manuscript was to develop a harmonized, large-scale single-nucleus transcriptomic atlas across multiple neurodegenerative and neuropsychiatric diseases. Rather than exhaust every analytical direction, we prioritized establishing a foundational framework with robust cross-disease contrasts, disease-specific patterns, and scalable methodologies to enable future studies. We'd like to point out that this study is part of a larger package of papers (see https://psych-ad.org), including studies with different focuses that delve deeper into specific subject areas. We believe that this study, in combination with forthcoming disease-focused companion manuscripts, will collectively provide both a comprehensive foundation and rich, disease-specific insights to guide future discovery and therapeutic development.<br><br>That said, we respectfully note that the manuscript does go beyond high-level observations. For example, we used precision-weighted linear mixed models (via Dreamlet) and multivariate adaptive shrinkage (mash) to extract disease-specific transcriptomic signatures after discounting shared effects. We also examined their genetic concordance using cross-trait heritability estimates, demonstrating how shared genetic architecture aligns with transcriptional overlap among specific disease pairs. These analyses allowed us to dissect disease mechanisms across cell types at multiple layers of granularity.<br><br>We also incorporated causal mediation modeling to infer pathways from polygenic risk to cognitive decline via intermediate phenotypes such as amyloid plaques, tau pathology, and cell-type-specific shifts. This revealed mechanistic links involving microglia, SST+ interneurons, and VLMCs in AD progression. Further, our trajectory modeling strategy enabled the decomposition of gene expression programs into early versus late changes and resilience-associated versus damaging signatures across cell classes.<br><br>Finally, we have revised the manuscript to better contextualize our findings in the prior literature. Specifically, we highlight the role of IL-17 signaling in early vascular damage in AD, and the contribution of lipid-droplet-accumulating microglia to late-stage dysfunction. We also expanded our pathway analysis using GWAS-informed modules, further linking transcriptomic trajectories with disease risk.

REF3-15. Error bars	
Additional questions and comments:<br><br>14. What are the error bars in Fig. 5e?	
Response	Thank you for raising this point. To enhance clarity, we have revised the figure legend to make the figure more accessible and informative to the readers.

	**Fig. 5e** illustrates the ranking of pairwise transcriptome similarity between two traits utilizing disease-specific gene signatures defined from **Fig. 5b**. To clarify, we calculated the Spearman correlation between two traits for each subclass. Each dot in the figure represents one such correlation. The boxplot summarizes the distribution of these correlations across all 27 subclasses, allowing for an assessment of the overall similarity trends.
Excerpt	**e** 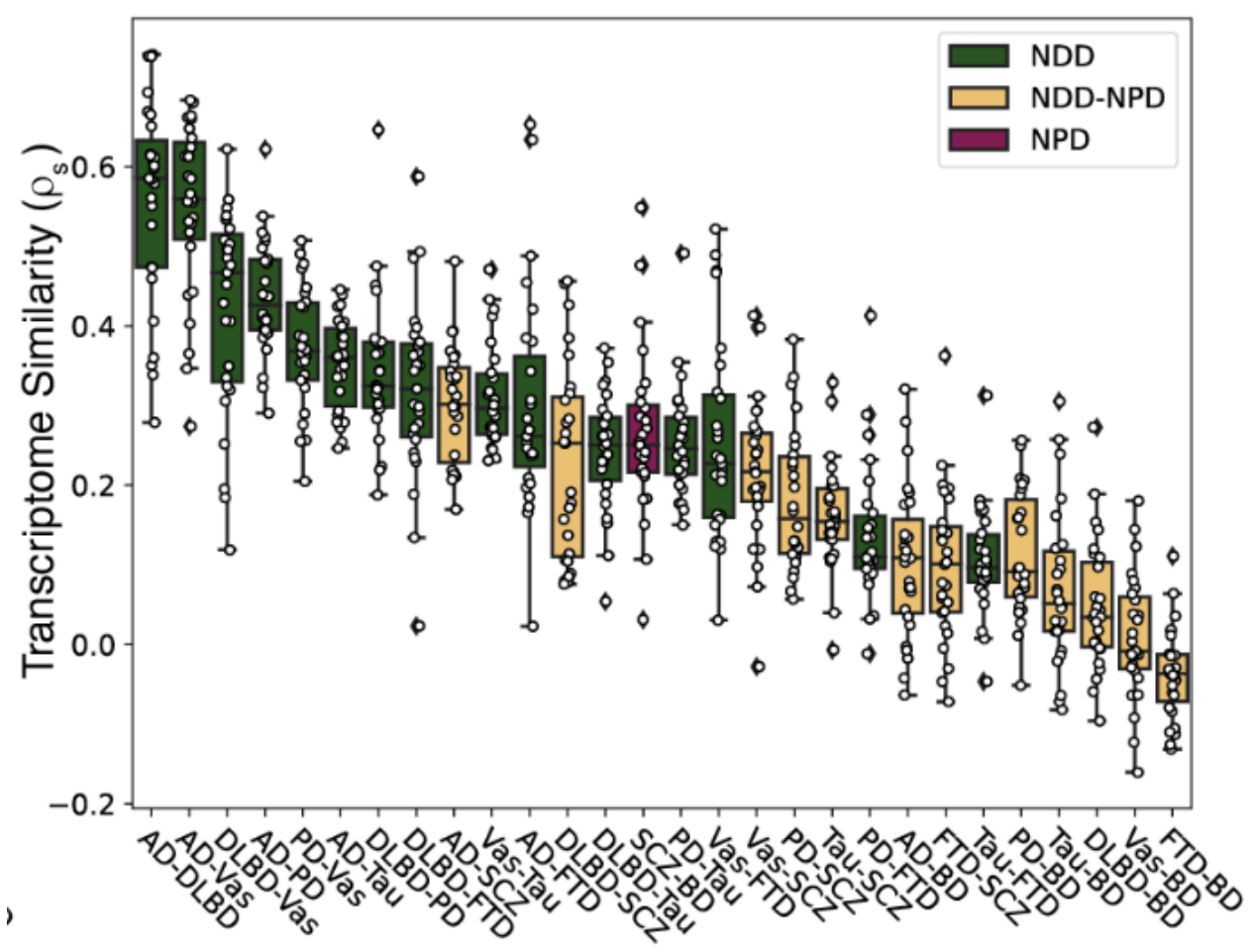 **Fig. 5**. **Cross-disorder variation of gene expression.** … **(e)** Transcriptome similarity (ρ_s) measured by Spearman correlation. Each dot represents the correlation between two traits within a specific subclass. The boxplot summarizes the distribution of these correlations across all 27 subclasses.

REF3-16. Clarification on meta-analysis
15. Line 812. It appears that the meta-analysis across cohorts was performed using the absolute value of the fold change. How does the model account for the possibility that a gene may be differentially expressed in opposite directions across the two samples?

Response	The meta-analysis was performed using the estimated log fold change and the standard error. However, we acknowledge that the original text was not clear. We improved the **Methods** section in the revision.
Excerpt	**Meta-Analysis (between brain sources):**<br>We conducted a meta-analysis to integrate results from different brain banks for the same disorder. Data tables from multiple brain banks were combined into a single list for each disorder and annotated with their respective sources. The `meta_analysis()` function in `dreamlet` was used to perform the meta-analysis, which involved combining data tables into a single data frame, grouping the data by assay. The standard error was computed from the estimated log fold change and moderated t-statistics according to `se = logFC / t`. The meta-analysis was performed using the `rma()` function from the `metafor` package with a fixed effects model. P-values were adjusted using the False Discovery Rate (FDR) method, and the negative log10 of the FDR values were calculated. This method was applied to datasets of AD, DLBD, Vas, PD, Tau, FTD, SCZ, and BD.

REF3-17. Typo	
16. Line 870. I think this should be abs(logFC) > 0.5 & FDR < 0.05	
Response	We appreciate the reviewer for catching this error. It was indeed a typo and we corrected it in the revision.
Excerpt	"... Genes were selected by applying the following criteria: logFC ≥ 0.5, FDR < 0.05. …"

REF3-18. Correlation between genetic constraint and donor variance	
17. Based on the low R value presented in Sup Fig. 3h, the correlation between genetic constraint and variance due to donor, while significant does not appear to fully explain gene expression variability between donors. A more informative analysis would be identifying cell-type specific genes that vary, and further correspond with diagnosis.	
Response	Similar to our response in **REF3-5**, we would like to clarify that VariancePartition (VP) analysis was not optimized for identifying cell-type-specific disease-effects, nor was that its intended purpose in our workflow. Rather, it serves to assess which covariates are most influential in driving expression variability, which can guide downstream modeling or prioritization of biological hypotheses. For identifying and quantifying disease-effects on cell types or genes, we performed more specific quantitative analyses modeling after complex study design and various covariates using dreamlet and crumblr.<br><br>With that said, we acknowledge that the Spearman correlation coefficient between variance explained by BrainDonor and genetic constraint metric (LOEUF) was moderate. To address, we performed additional sensitivity analyses to ensure our conclusions about the relationship between genetic constraints and donor variation are robust. We performed VP analyses within more homogeneous cell populations by stacking cell type-specific assays into three superclasses, namely, EN, IN, and Non-Neuron, similar to previous approach by BICCN exploring inter-individual variation of human brain[31]. Overall, we found consistent results using EN, IN, and Non-Neuron superclasses that genes with lower donor variation tend to have higher genetic constraints (**Fig. REF3-18**, shown below). On the other hand, genes with high inter-individual variability were enriched with basal cellular functions (i.e. housekeeping genes, RNA metabolism and, in particular, cellular response to heat stress), corroborating previous findings[81,82] (**Fig. 3g**, **Supplementary Figs. 6a,m**).<br><br>However, we noticed the relationship between donor variation and genetic constraints are non-linear and might be more nuanced than we originally reported. As a result, we decided to remove the supplementary correlation plot and our claim about the "inverse correlation" in this revision. Instead, we made it clear that we are making two independent claims - the enrichment of constrained genes with genes of low donor variation (**Fig. 3f, Supplementary Fig. 6l**) and the functional enrichment of basal cellular functions with genes of high donor variation (**Fig. 3g, Supplementary Figs. 6a,m**).<br><br>To facilitate the interpretation of variance partition results with functional insights, we added functional enrichment analyses of top 10% of highly variable genes for each covariate (**Supplementary Fig. 6a**). We additionally tested enrichment of constrained genes for all covariates using GeneBayes[83] S_{het} metrics (**Supplementary Fig. 6l**), in addition to the gnomAD LOEUF metric. Finally, we show functional enrichment of low and high donor variation genes along constrained genes defined by gnomAD[84] pLoF (LOEUF < 0.6) or GeneBayes[83] (S_{het} > 1e-3) (**Supplementary Fig. 6m**).

EN:

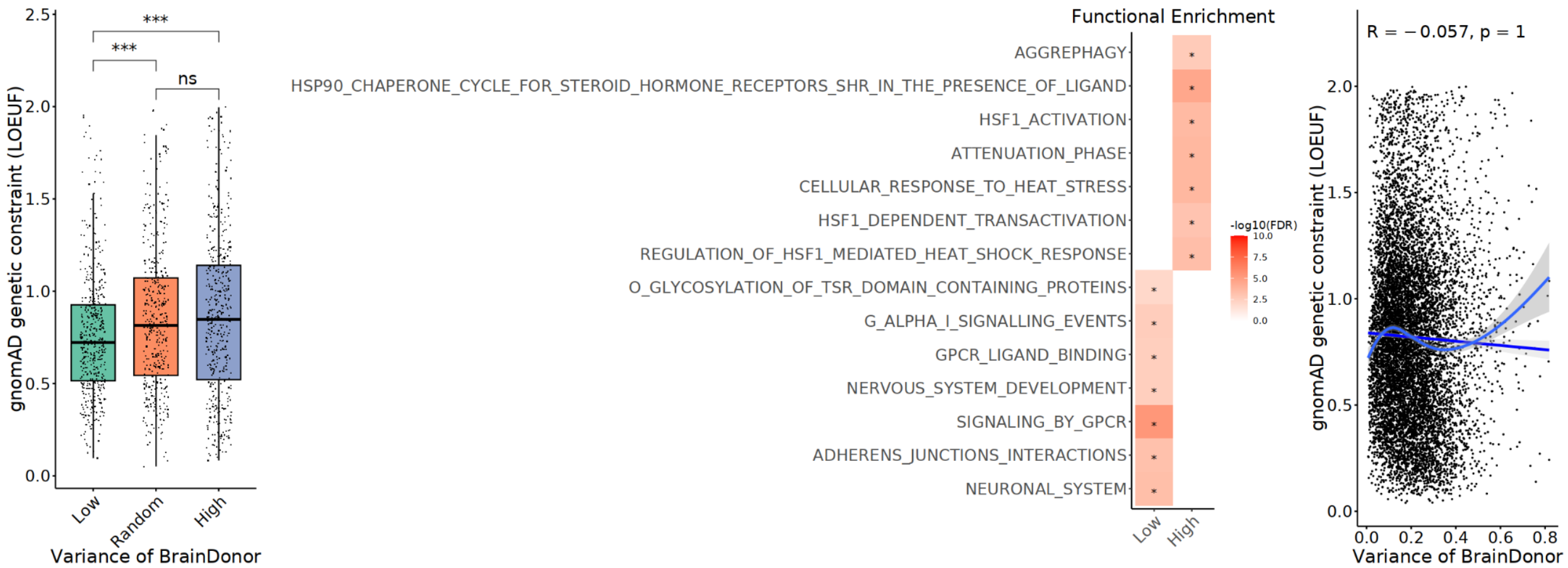

IN:

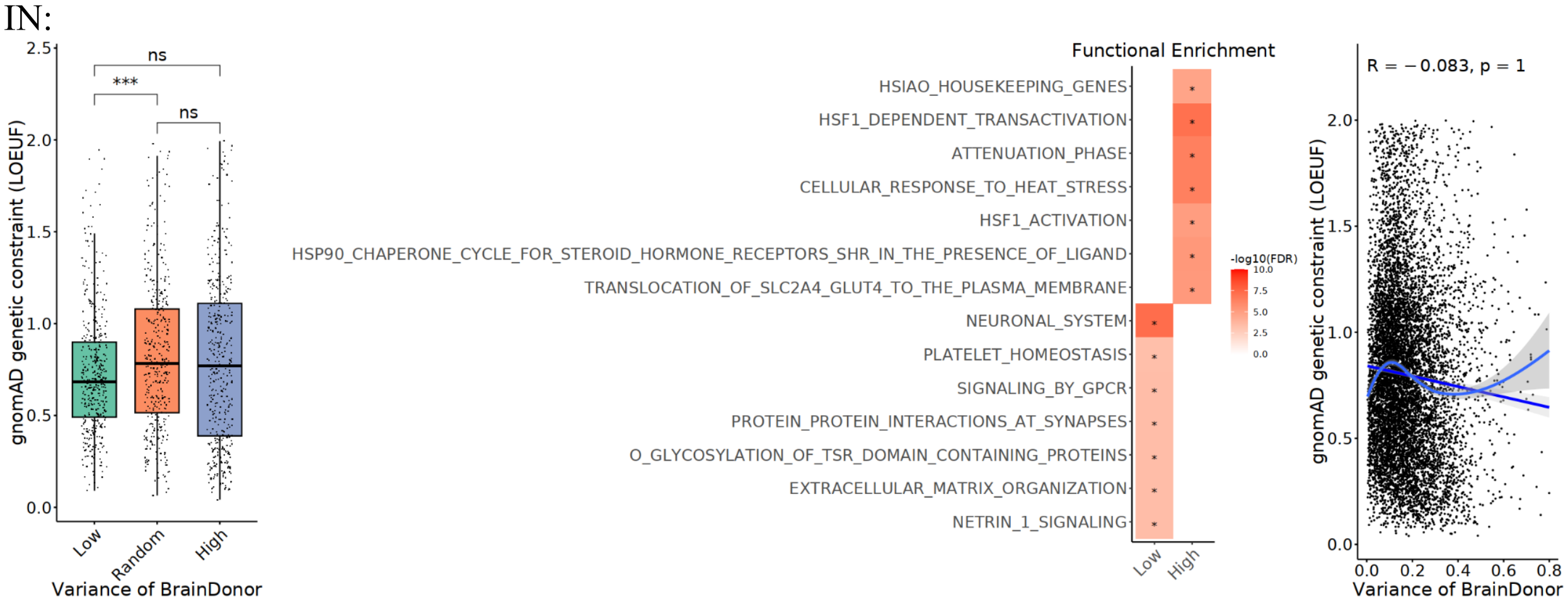

Non-Neuron:

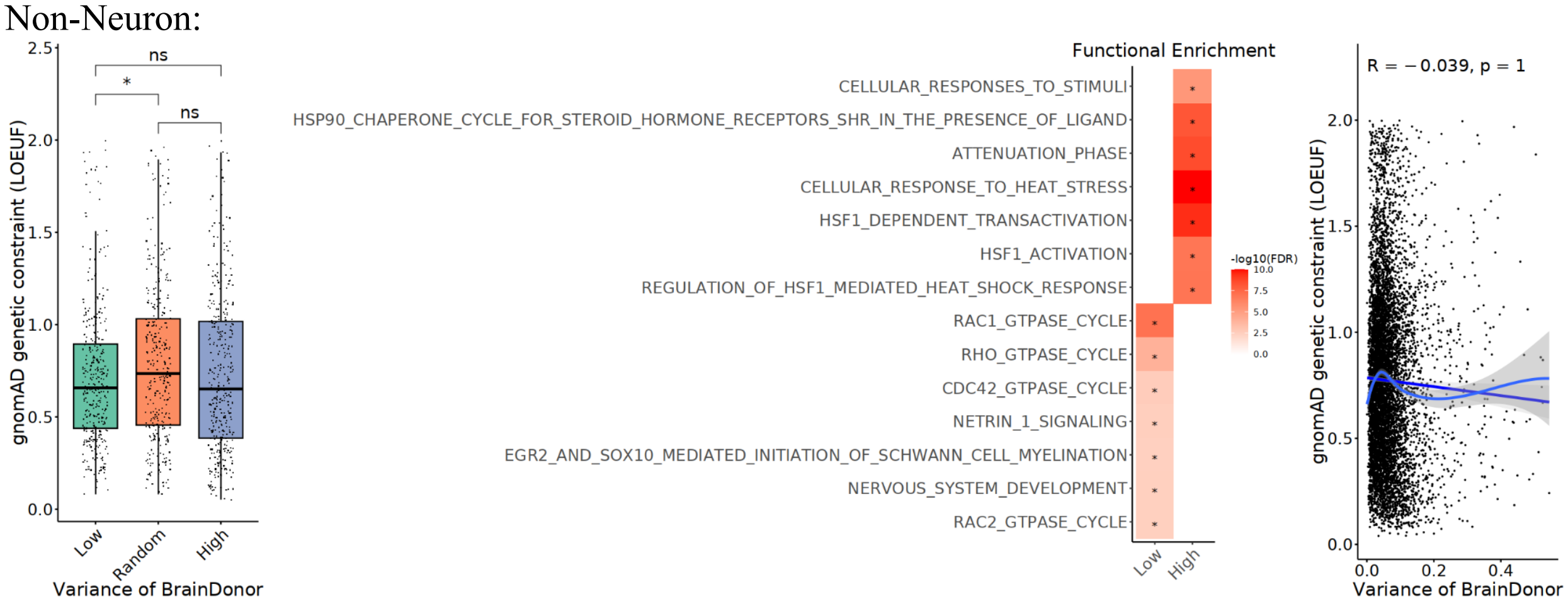

Fig. REF3-18. **(left)** Genetic constraints analyses based on variance partition estimates within three superclasses, namely, Excitatory Neurons (EN), Inhibitory Neurons (IN), and Non-Neurons (Non-Neuron). Genetic constraints of gene groups measured by LOEUF score (upper bound of 90% confidence interval for o/e ratio for high confidence predicted loss-of-function (pLoF) variants; lower values indicate higher genetic constraints). "Low" indicates genes in the bottom 5% quantile of the brain donor variance. "High" indicates genes in the top 5% quantile of the brain donor variance. "Random" indicates random 5% of genes that are in neither the "Low" nor "High" category. **(middle)** Functional enrichment for genes with high or low inter-individual variation across three superclasses. HSIAO_HOUSEKEEPING_GENES: housekeeping genes identified as expressed across 19 normal

tissues[8], HOUNKPE_HOUSEKEEPING_GENES: 1,130 human and mouse housekeeping genes[9]. **(right)** Spearman correlation test between variance explained by inter-individual variation and LOEUF conservation score across three superclasses.

Excerpt

"... Genes with lower inter-individual variation had higher genetic constraints, as measured by the gnomAD[84] Loss-of-function Observed/Expected Upper-bound Fraction (LOEUF) score or GeneBayes[83] constraint metric (S_{het}) (**Fig. 3f, Supplementary Fig. 6l**). These conserved genes were functionally implicated in nervous system development and synaptic functions, indicating gene program in neuronal development is conserved across individuals (**Supplementary Fig. 6m**). On the other hand, genes with high inter-individual variability were enriched with basal cellular functions (i.e. housekeeping genes, RNA metabolism and, in particular, cellular response to heat stress), corroborating previous findings[81,82] (**Fig. 3g, Supplementary Figs. 6a,m**)."

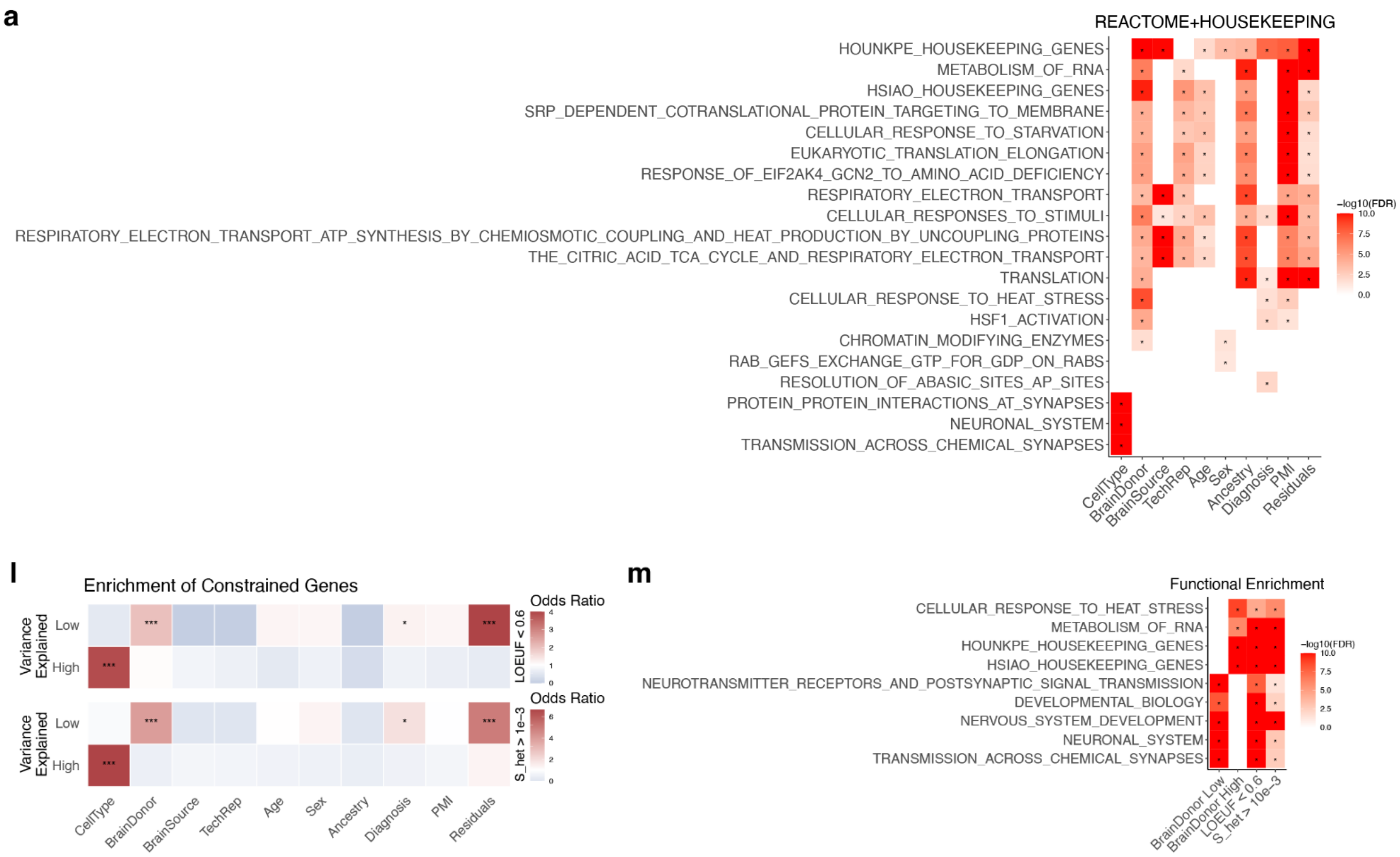

Supplementary Fig. 6. **(a)** Functional enrichment of top variable genes identified by variance partition analysis. For each covariate, genes in the top 10% quantile were selected for functional enrichment using Reactome[85] and housekeeping gene sets from previous literature. HSIAO_HOUSEKEEPING_GENES: housekeeping genes identified as expressed across 19 normal tissues[8], HOUNKPE_HOUSEKEEPING_GENES: 1,130 human and mouse housekeeping genes[9]. … **(l)** Enrichment of constrained genes measured by either the gnomAD[84] Loss-of-function Observed/Expected Upper-bound Fraction (LOEUF) score < 0.6 or GeneBayes[83] S_{het} > 1e-3. Hypergeometric test with p-value <= 0.05 *, <= 0.01 **, <= 0.001 ***. **(m)** Functional enrichment of genes with low donor variation (bottom 10% quantile; BrainDonor Low), high donor variation (top 10% quantile; BrainDonor High), and constrained genes defined by gnomAD pLoF (LOEUF < 0.6) or GeneBayes[83] (S_{het} > 1e-3).

REF3-19. MAPT haplotypes

18. The authors have convincingly shown that the H1 and H2 MAPT haplotypes impacts expression of ARL17B (Fig 3e). Given the predictive power of this locus and potential importance in PD progression, could this analysis be expanded to identify other cell-type specific gene expression changes?

Response

We appreciate the reviewer's suggestion. In response, we expanded the H1 haplotype analysis, which revealed several additional genes implicated in neurodegeneration (**Supplementary Fig. 6i**). These findings are detailed in the **Excerpt**, and we have further elaborated on their potential significance in the revised manuscript.

Excerpt

"... The H1 haplotype exhibited *cis*-regulatory effects on several genes near the MAPT locus that are relevant to neurodegeneration[86] (**Supplementary Fig. 6i**). Specifically, H1 haplotypes in neurons and astrocytes were associated with increased expression of Leucine Rich Repeat Containing 37A genes (LRRC37A2/A3). LRRC37A proteins have been shown to interact with α-synuclein and co-localize with Lewy bodies in PD brains[87]. Another gene upregulated by the H1 haplotype was Pleckstrin Homology Domain-Containing Family M Member 1 (PLEKHM1), which has been linked to PD susceptibility by GWAS[88,89]. The H1 haplotype also demonstrated interchromosomal effects, notably on phosphodiesterase 8B (PDE8B), a gene implicated in movement disorders[90–93]. Furthermore, heterogeneous nuclear ribonucleoprotein K (HNRNPK), a gene involved in synaptic plasticity regulation[94], was downregulated by the H1 haplotype. …"

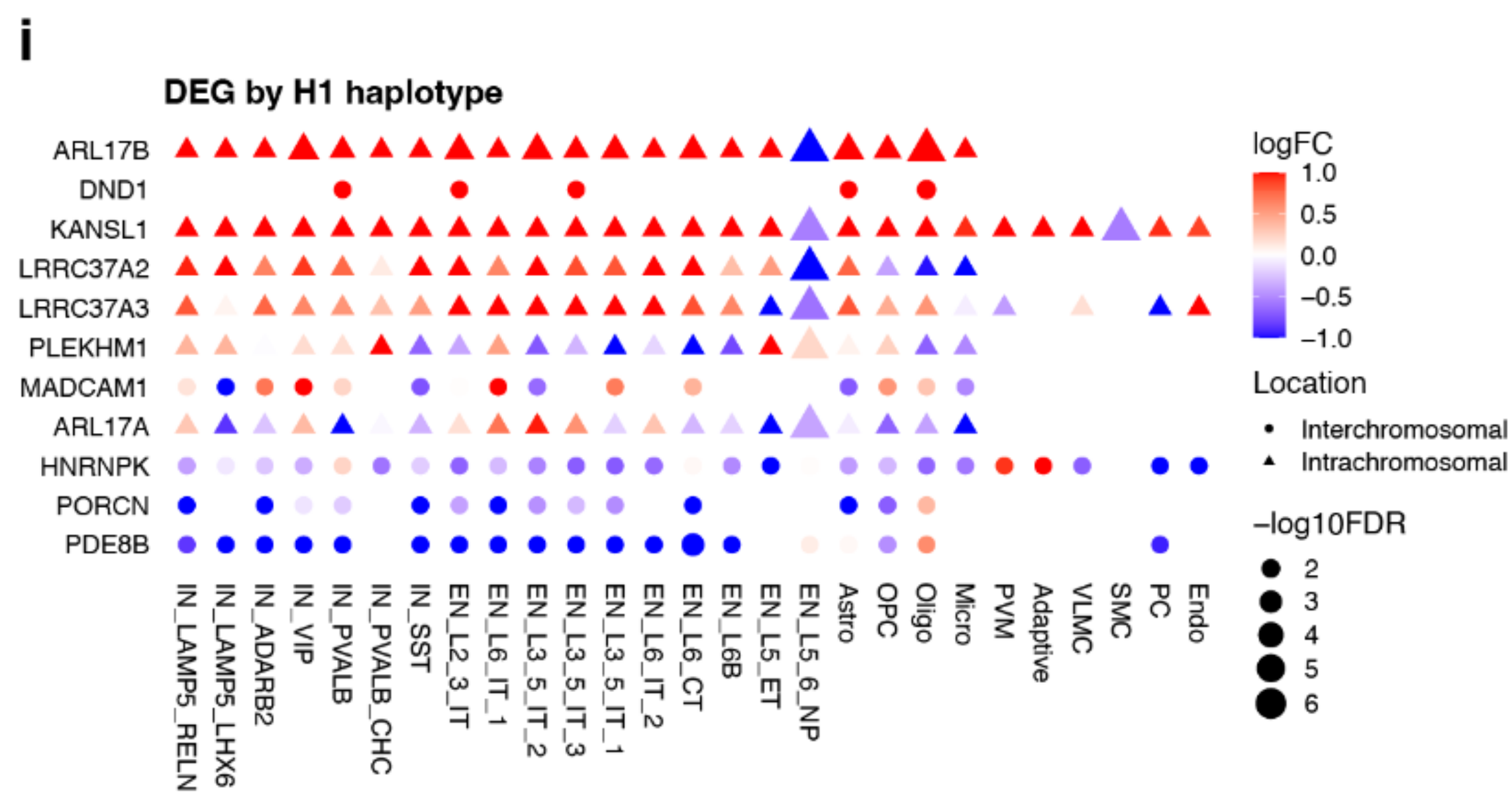

Supplementary Fig. 6. … **(i)** Differentially expressed genes by dosage of H1 allele using additive effects. Intrachromosomal effects are genes in the same chromosome as MAPT locus (17q21). Otherwise, interchromosomal effects. All genes shown have FDR < 0.05.

REF3-20. Supplementary Table 1

19. Please add a column in sup table 1 to indicate which individuals were used for each subset in fig 1 (1160, 696, 234). After reviewing the criteria for selection detailed in the methods and sorting Sup Table 1 by these criteria, I could not recreate the groups described in Fig 1.

Response

We appreciate your comments on **Supplementary Table 1**. We acknowledge it was not immediately clear which donors were included for our three-tier analyses. We added the following variables to indicate individuals included in **Fig. 1c-e**:

- Tier1_crossDis and Tier1_crossDis_dx
- Tier2_AD and Tier2_AD_dx
- Tier3_NPS and Tier3_NPS_dx

Excerpt

Supplementary Table 1. Clinical and technical metadata of 1,494 donors in the PsychAD cohort. Metadata includes binary contrasts and continuous variables used in the study. Columns named crossDis_AD, crossDis_SCZ,

	crossDis_DLBD, crossDis_Vas, crossDis_BD, crossDis_Tau, crossDis_PD, and crossDis_FTD describe donors used in cross-disorder analysis depicted in Fig. 1c as binary contrasts. The value 1 indicates cases while 0 indicates disease-free controls used for the contrast. Columns named Tier1_crossDis, Tier2_AD, and Tier3_NPS indicate individuals included in 3-tier analyses.

REF3-21. Presentation of age	
20. Ages - sup table 1 goes up to 89+, fig 1a goes to 99+, and text mentions a 108 year old. Please be consistent. Presenting actual ages in sup table 1 would be preferable unless there are privacy concerns.	
Response	We understand that the inconsistency in reporting ages over 89 can be confusing. Under the Privacy Rule, the NIH mandates the removal of protected health information of all human subjects, including the reporting of all ages over 89 (https://privacyruleandresearch.nih.gov/healthservicesprivacy.asp). In **Fig. 1a**, we are showing the age distribution and using 99+ on the axis tick could be misleading. We revised it to 99 instead of 99+.
Excerpt	**a** 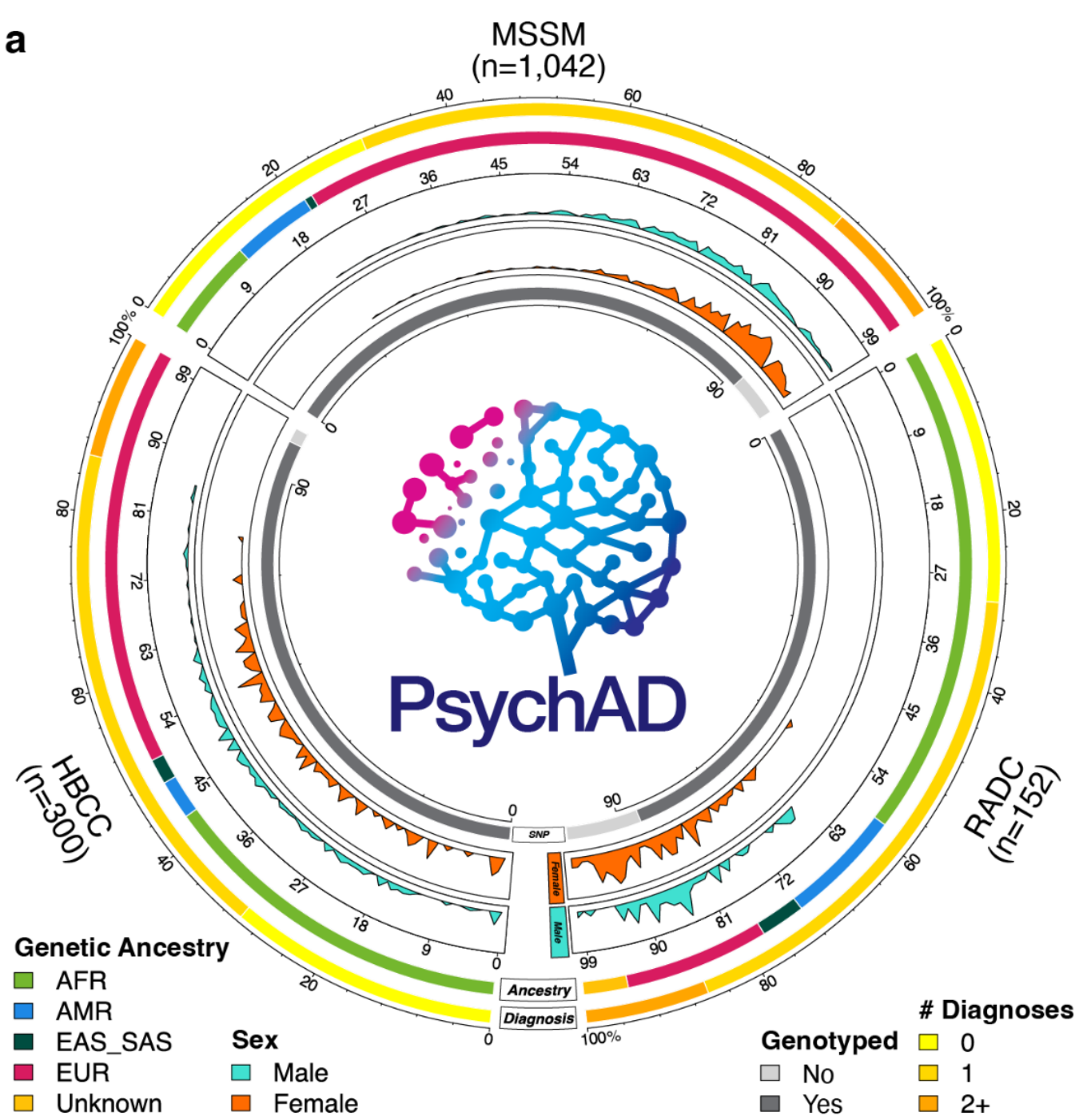 **Fig. 1. Overview of the PsychAD cohort and study design. (a)** Composition of the PsychAD cohort, comprising 1,494 donors from three tissue sources: MSSM (n=1,042), HBCC (n=300), and RADC (n=152). The circular plot details donor demographics, including genetic ancestry (AFR: African, AMR: Ad Mixed American, EAS: East Asian, SAS: South Asian, EUR: European, and Unknown), sex distribution (male and female), and percentage of available genotype data. The number of diagnoses per donor is represented by color-coded bars, distinguishing individuals with zero, one, or multiple NDD and NPD diagnoses. Age at death is illustrated in radial histograms, showing variability across tissue sources. The age distribution is binned and capped at 99+ years. …

REF3-22. Supplementary Table 1	
21. For sup table 1, please add "Cross-disorder groups" depicted in Fig1c	
Response	Thank you for your valuable feedback. **Supplementary Table 1** already contains the "cross-disorder group" information described in **Fig. 1c**. They are described in columns crossDis_AD, crossDis_SCZ, crossDis_DLBD, crossDis_Vas, crossDis_BD, crossDis_Tau, crossDis_PD, and crossDis_FTD as binary contrasts. "1" indicates case donors and "0" indicates disease-free control donors. However, we feel that this was not clear enough. In the revision, we clarified this by updating the descriptions to Supplementary Tables.
Excerpt	**Supplementary Table 1**. Clinical and technical metadata of 1,494 donors in the PsychAD cohort. Metadata includes binary contrasts and continuous variables used in the study. Columns named crossDis_AD, crossDis_SCZ, crossDis_DLBD, crossDis_Vas, crossDis_BD, crossDis_Tau, crossDis_PD, and crossDis_FTD describe donors used in cross-disorder analysis depicted in Fig. 1c as binary contrasts. The value 1 indicates cases while 0 indicates disease-free controls used for the contrast. Columns named Tier1_crossDis, Tier2_AD, and Tier3_NPS indicate individuals included in 3-tier analyses.

REF3-23. Laminar distribution of PVALB neurons	
22. The authors' statement that IN subclasses being randomly distributed (line 159) is counter to existing literature - for example PVALB neuron subtypes have layer-specific enrichments (PMC10660579)	
Response	Thank you for pointing out this oversight in the original text. While it was not our intention to claim inhibitory neurons are uniformly distributed across all cortical layers, we agree that the original writing could easily be interpreted that way.<br><br>We modified the relevant passage (see **Excerpt**) to more clearly reflect what is found in the data. In addition, we performed additional analysis to investigate the distribution of IN subclasses across cortical layers (see **REF3-4**). While the degree of granularity we could achieve was limited, we found non-uniform distributions of IN nuclei densities, generally agreeing with previous literature on this topic. These results are included in **Supplementary Fig. 4e**, and a detailed description of the additional analysis can be found in the **Methods** section of the paper (under the "Spatial validation of cellular taxonomy" section, sub-sections "Definition of rough laminar layers within tissue" and "Density estimation and comparison for IN subclasses").
Excerpt	"... Consistent with previous reports[25,95,96], IN subclasses did not display the same degree of laminar organization as EN (**Supplementary Fig. 4i**). A more careful examination of the spatial density of nuclei from each subclass within coarsely defined laminar regions (see **Methods**) revealed that IN distribution is not uniform across cortical layers. For example, we observed a preference for the ADARB2$^+$ and VIP$^+$ interneurons for outer cortical layers, while both PVALB$^+$ interneuron subclasses were distributed across layers L2-6 with less prominent bias (**Supplementary Figs. 4e-f**). Notably, we observed SST$^+$ inhibitory neuron subtypes had distinct layer-specific enrichment patterns (**Supplementary Figs. 4f,k**)."

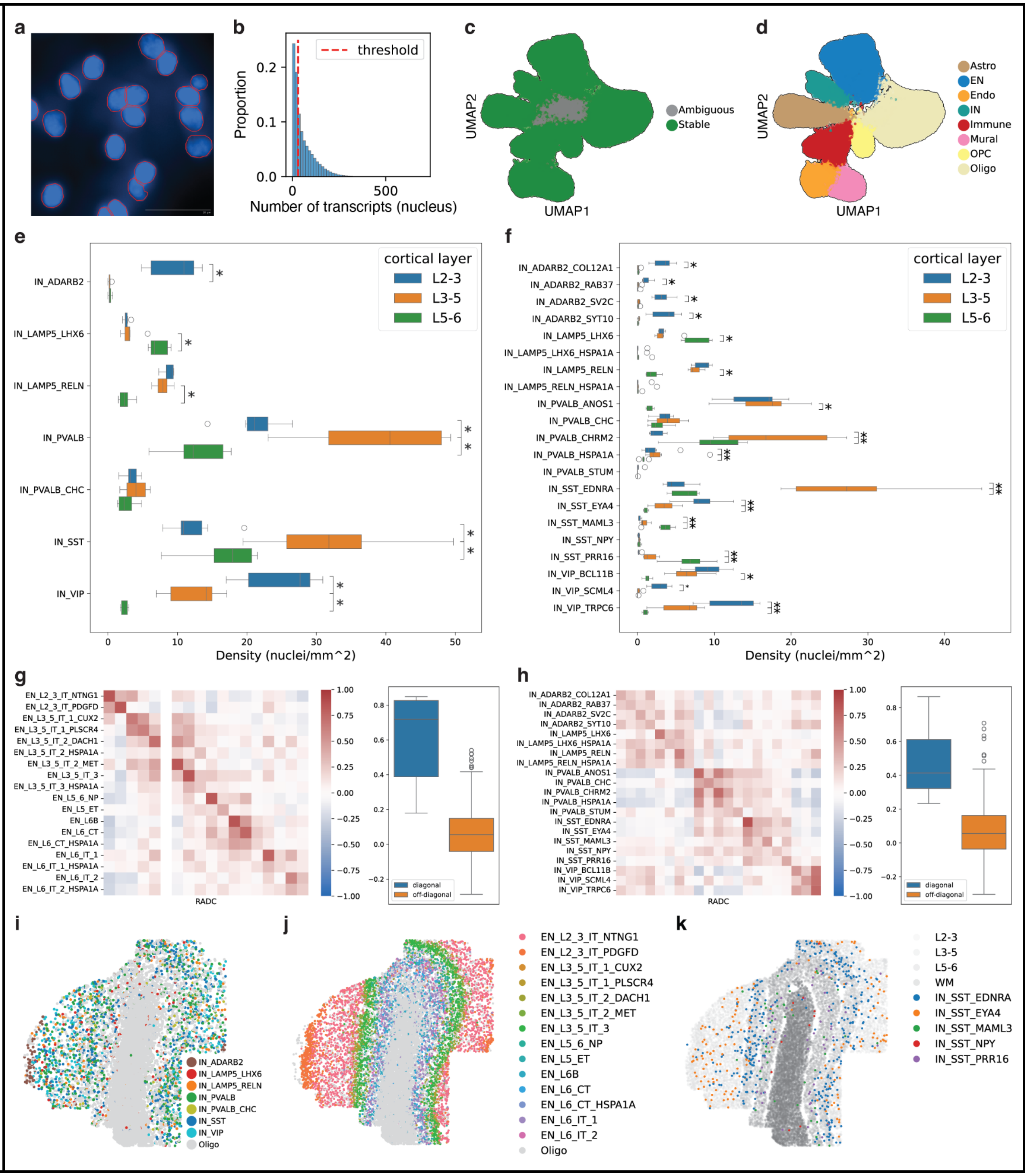

a
b
threshold
Proportion
Number of transcripts (nucleus)
c
Ambiguous
Stable
UMAP1
UMAP2
d
Astro
EN
Endo
IN
Immune
Mural
OPC
Oligo
e
cortical layer
L2-3
L3-5
L5-6
IN_ADARB2
IN_LAMP5_LHX6
IN_LAMP5_RELN
IN_PVALB
IN_PVALB_CHC
IN_SST
IN_VIP
Density (nuclei/mm^2)
f
IN_ADARB2_COL12A1
IN_ADARB2_RAB37
IN_ADARB2_SV2C
IN_ADARB2_SYT10
IN_LAMP5_LHX6
IN_LAMP5_LHX6_HSPA1A
IN_LAMP5_RELN
IN_LAMP5_RELN_HSPA1A
IN_PVALB_ANOS1
IN_PVALB_CHC
IN_PVALB_CHRM2
IN_PVALB_HSPA1A
IN_PVALB_STUM
IN_SST_EDNRA
IN_SST_EYA4
IN_SST_MAML3
IN_SST_NPY
IN_SST_PRR16
IN_VIP_BCL11B
IN_VIP_SCML4
IN_VIP_TRPC6
Density (nuclei/mm^2)
g
RADC
diagonal
off-diagonal
h
RADC
diagonal
off-diagonal
i
IN_ADARB2
IN_LAMP5_LHX6
IN_LAMP5_RELN
IN_PVALB
IN_PVALB_CHC
IN_SST
IN_VIP
Oligo
j
EN_L2_3_IT_NTNG1
EN_L2_3_IT_PDGFD
EN_L3_5_IT_1_CUX2
EN_L3_5_IT_1_PLSCR4
EN_L3_5_IT_2_DACH1
EN_L3_5_IT_2_MET
EN_L3_5_IT_3
EN_L5_6_NP
EN_L5_ET
EN_L6B
EN_L6_CT
EN_L6_CT_HSPA1A
EN_L6_IT_1
EN_L6_IT_2
Oligo
k
L2-3
L3-5
L5-6
WM
IN_SST_EDNRA
IN_SST_EYA4
IN_SST_MAML3
IN_SST_NPY
IN_SST_PRR16

	**Supplementary Fig. 4**. Validation of cellular taxonomy using spatial transcriptomics. **(a)-(d)** Processing and QC of Xenium in situ spatial transcriptomics data. **(a)** Example image to demonstrate nuclei segmentation. The DAPI signal is shown in blue, and boundaries of segmented nuclei are shown as red outlines. **(b)** Distribution of transcript counts per nucleus across all samples. The chosen threshold (30 counts) is shown as a red dotted line for reference. **(c)** UMAP of all Xenium nuclei containing >30 transcripts. Clusters were assigned "stable" or "ambiguous" labels based on how many of their nuclei were mapped to the same class using scANVI (**Methods**). **(d)** The same UMAP as (c), after removing ambiguous clusters and nuclei (mismatch between nuclei cluster and scANVI labels). Nuclei are colored based on their assigned class. **(e)** Comparison of nuclei density of IN subclasses by cortical layers (**Methods**). Note that y axes are in units of $1/\mu m^2$ scaled with a factor of 1e-5 (equivalent to 10 nuclei per mm^2). Paired-sample Wilcoxon rank sum testing between adjacent cortical layers. Asterisk denotes p-value < 0.05. **(f)** Comparison of nuclei density of IN subtypes by cortical layer for IN subtypes, similarly to the subclass-level analysis in (e). **(g-h)** Concordance of snRNA-seq and Xenium **(g)** EN and **(h)** IN subtypes. Pearson correlation between each pair of subtypes between Xenium (rows) and the RADC cohort snRNA-seq (columns) at pseudo-bulk level (see **Methods**). Adjacent box-plots compare the diagonal and off-diagonal elements. **(i)** Example spatial distribution of IN subclasses. A single tissue section measured with Xenium in situ spatial transcriptomics data. **(j)** Example spatial distribution of EN subtypes. **(k)** Example spatial distribution for SST-expressing IN subtypes. Coarse cortical layer structure is shown in greyscale.

REF3-24. Remarks on code availability	
The GitHub archive includes a vast number of scripts used in the analysis. I did not have time to go through all of the scripts, but the ones I looked at appeared well-written and sufficiently documented to make the analysis reproducible.	
Response	We appreciate your positive feedback. We further improved our code repository with more annotations and instructions on using our scripts to replicate the analyses (see **REF2-12**).

Reference

1. Mandric, I. *et al.* Optimized design of single-cell RNA sequencing experiments for cell-type-specific eQTL analysis. *Nat. Commun.* **11**, 5504 (2020).
2. Bellenguez, C. *et al.* New insights into the genetic etiology of Alzheimer's disease and related dementias. *Nat. Genet.* **54**, 412–436 (2022).
3. Sealock, J. M. *et al.* Use of the PsycheMERGE network to investigate the association between depression polygenic scores and white blood cell count. *JAMA Psychiatry* **78**, 1365–1374 (2021).
4. Escott-Price, V., Myers, A., Huentelman, M., Shoai, M. & Hardy, J. Polygenic risk score analysis of Alzheimer's disease in cases without APOE4 or APOE2 alleles. *J. Prev. Alzheimers Dis.* **6**, 16–19 (2019).
5. Logue, M. W. *et al.* Use of an Alzheimer's disease polygenic risk score to identify mild cognitive impairment in adults in their 50s. *Mol. Psychiatry* **24**, 421–430 (2019).
6. Mathys, H. *et al.* Single-cell atlas reveals correlates of high cognitive function, dementia, and resilience to Alzheimer's disease pathology. *Cell* **186**, 4365–4385.e27 (2023).
7. Gabitto, M. I. *et al.* Integrated multimodal cell atlas of Alzheimer's disease. *Nat. Neurosci.* **27**, 2366–2383 (2024).
8. Hsiao, L. L. *et al.* A compendium of gene expression in normal human tissues. *Physiol. Genomics* **7**, 97–104 (2001).
9. Hounkpe, B. W., Chenou, F., de Lima, F. & De Paula, E. V. HRT Atlas v1.0 database: redefining human and mouse housekeeping genes and candidate reference transcripts by mining massive RNA-seq datasets. *Nucleic Acids Res.* **49**, D947–D955 (2021).
10. Hoffman, G. E. *et al.* Efficient differential expression analysis of large-scale single cell transcriptomics data using dreamlet. *bioRxiv* (2023) doi:10.1101/2023.03.17.533005.
11. Urbut, S. M., Wang, G., Carbonetto, P. & Stephens, M. Flexible statistical methods for estimating and testing effects in genomic studies with multiple conditions. *Nat. Genet.* **51**, 187–195 (2019).
12. Zeng, B. *et al.* Single-nucleus atlas of cell-type specific genetic regulation in the human brain. *medRxiv* (2024) doi:10.1101/2024.11.02.24316590.
13. Boettger, L. M., Handsaker, R. E., Zody, M. C. & McCarroll, S. A. Structural haplotypes and recent

evolution of the human 17q21.31 region. *Nat. Genet.* **44**, 881–885 (2012).

14. Wade-Martins, R. Genetics: The MAPT locus-a genetic paradigm in disease susceptibility. *Nat. Rev. Neurol.* **8**, 477–478 (2012).
15. Tian, Y. *et al.* Shared Genetics and Comorbid Genes of Amyotrophic Lateral Sclerosis and Parkinson's Disease. *Mov. Disord.* **38**, 1813–1821 (2023).
16. Català-Senent, J. F. *et al.* A deep transcriptome meta-analysis reveals sex differences in multiple sclerosis. *Neurobiol. Dis.* **181**, 106113 (2023).
17. Cooper, Y. A. *et al.* Functional regulatory variants implicate distinct transcriptional networks in dementia. *Science* **377**, eabi8654 (2022).
18. Vandrovcova, J. *et al.* Association of MAPT haplotype-tagging SNPs with sporadic Parkinson's disease. *Neurobiol. Aging* **30**, 1477–1482 (2009).
19. Gandal, M. J. *et al.* Shared molecular neuropathology across major psychiatric disorders parallels polygenic overlap. *Science* **359**, 693–697 (2018).
20. Sadeghi, I. *et al.* Brain transcriptomic profiling reveals common alterations across neurodegenerative and psychiatric disorders. *Comput. Struct. Biotechnol. J.* **20**, 4549–4561 (2022).
21. Voineagu, I. *et al.* Transcriptomic analysis of autistic brain reveals convergent molecular pathology. *Nature* **474**, 380–384 (2011).
22. Green, G. S. *et al.* Cellular communities reveal trajectories of brain ageing and Alzheimer's disease. *Nature* **633**, 634–645 (2024).
23. Fullard, J. F. *et al.* Population-scale cross-disorder atlas of the human prefrontal cortex at single-cell resolution. *Sci. Data* (Under Revision).
24. Fleming, S. J. *et al.* Unsupervised removal of systematic background noise from droplet-based single-cell experiments using CellBender. *Nat. Methods* **20**, 1323–1335 (2023).
25. Ma, S. *et al.* Molecular and cellular evolution of the primate dorsolateral prefrontal cortex. *Science* **377**, eabo7257 (2022).
26. BRAIN Initiative Cell Census Network (BICCN). A multimodal cell census and atlas of the mammalian primary motor cortex. *Nature* **598**, 86–102 (2021).

27. Zheng, G. X. Y. *et al.* Massively parallel digital transcriptional profiling of single cells. *Nat. Commun.* **8**, 14049 (2017).

28. Traag, V. A., Waltman, L. & van Eck, N. J. From Louvain to Leiden: guaranteeing well-connected communities. *Sci. Rep.* **9**, 5233 (2019).

29. McInnes, L., Healy, J. & Melville, J. UMAP: Uniform Manifold Approximation and Projection for Dimension Reduction. *arXiv [stat.ML]* (2018).

30. Rentzsch, P., Kollotzek, A., Mohammadi, P. & Lappalainen, T. Recalibrating differential gene expression by genetic dosage variance prioritizes functionally relevant genes. *Genomics* (2024).

31. Johansen, N. *et al.* Interindividual variation in human cortical cell type abundance and expression. *Science* **382**, eadf2359 (2023).

32. Hoffman, G. E. & Roussos, P. Fast, flexible analysis of differences in cellular composition with crumblr. *Res. Sq.* (2025) doi:10.21203/rs.3.rs-5921338/v1.

33. Fan, Z., Brooks, D. J., Okello, A. & Edison, P. An early and late peak in microglial activation in Alzheimer's disease trajectory. *Brain* **140**, 792–803 (2017).

34. Marschallinger, J. *et al.* Lipid-droplet-accumulating microglia represent a dysfunctional and proinflammatory state in the aging brain. *Nat. Neurosci.* **23**, 194–208 (2020).

35. Claes, C. *et al.* Plaque-associated human microglia accumulate lipid droplets in a chimeric model of Alzheimer's disease. *Mol. Neurodegener.* **16**, 50 (2021).

36. Haney, M. S. *et al.* APOE4/4 is linked to damaging lipid droplets in Alzheimer's disease microglia. *Nature* **628**, 154–161 (2024).

37. Boyle, E. A., Li, Y. I. & Pritchard, J. K. An expanded view of complex traits: From polygenic to omnigenic. *Cell* **169**, 1177–1186 (2017).

38. Weiner, D. J. *et al.* Polygenic architecture of rare coding variation across 394,783 exomes. *Nature* **614**, 492–499 (2023).

39. Karczewski, K. J. *et al.* Systematic single-variant and gene-based association testing of thousands of phenotypes in 394,841 UK Biobank exomes. *Cell Genom.* **2**, 100168 (2022).

40. Hoffman, G. E. & Roussos, P. *Count Ratio Uncertainty Modeling Based Linear Regression*. (2024).

doi:10.5281/zenodo.12752107.

41. Pedregosa, F. *et al.* Scikit-learn: Machine Learning in Python. *J. Mach. Learn. Res.* **12**, 2825–2830 (2011).
42. Borrell, L. N. *et al.* Race and Genetic Ancestry in Medicine - A Time for Reckoning with Racism. *N. Engl. J. Med.* **384**, 474–480 (2021).
43. Korsunsky, I. *et al.* Fast, sensitive and accurate integration of single-cell data with Harmony. *Nat. Methods* **16**, 1289–1296 (2019).
44. Xu, C. *et al.* Probabilistic harmonization and annotation of single-cell transcriptomics data with deep generative models. *Mol. Syst. Biol.* **17**, e9620 (2021).
45. Hashimoto, T. *et al.* Alterations in GABA-related transcriptome in the dorsolateral prefrontal cortex of subjects with schizophrenia. *Mol. Psychiatry* **13**, 147–161 (2008).
46. Curley, A. A. *et al.* Cortical deficits of glutamic acid decarboxylase 67 expression in schizophrenia: clinical, protein, and cell type-specific features. *Am. J. Psychiatry* **168**, 921–929 (2011).
47. Arion, D. *et al.* Transcriptome alterations in prefrontal pyramidal cells distinguish schizophrenia from bipolar and major depressive disorders. *Biol. Psychiatry* **82**, 594–600 (2017).
48. Hashimoto, T. *et al.* Gene expression deficits in a subclass of GABA neurons in the prefrontal cortex of subjects with schizophrenia. *J. Neurosci.* **23**, 6315–6326 (2003).
49. Chung, D. W. *et al.* Dysregulated ErbB4 splicing in schizophrenia: Selective effects on parvalbumin expression. *Am. J. Psychiatry* **173**, 60–68 (2016).
50. Chung, D. W., Fish, K. N. & Lewis, D. A. Pathological basis for deficient excitatory drive to cortical parvalbumin interneurons in schizophrenia. *Am. J. Psychiatry* **173**, 1131–1139 (2016).
51. Enwright, J. F. *et al.* Reduced labeling of parvalbumin neurons and perineuronal nets in the dorsolateral prefrontal cortex of subjects with schizophrenia. *Neuropsychopharmacology* **41**, 2206–2214 (2016).
52. Kaar, S. J., Angelescu, I., Marques, T. R. & Howes, O. D. Pre-frontal parvalbumin interneurons in schizophrenia: a meta-analysis of post-mortem studies. *J. Neural Transm. (Vienna)* **126**, 1637–1651 (2019).
53. Bhattacherjee, A. *et al.* Cell type-specific transcriptional programs in mouse prefrontal cortex during adolescence and addiction. *Nat. Commun.* **10**, 4169 (2019).

54. Stefanoska, K. *et al.* Alzheimer's disease: Ablating single master site abolishes tau hyperphosphorylation. *Science Advances* (2022) doi:10.1126/sciadv.abl8809.

55. Schwartzentruber, J. *et al.* Genome-wide meta-analysis, fine-mapping and integrative prioritization implicate new Alzheimer's disease risk genes. *Nature Genetics* **53**, 392–402 (2021).

56. Louveau, A. *et al.* Structural and functional features of central nervous system lymphatic vessels. *Nature* **523**, 337–341 (2015).

57. Ahn, J. H. *et al.* Meningeal lymphatic vessels at the skull base drain cerebrospinal fluid. *Nature* **572**, 62–66 (2019).

58. Li, X. *et al.* Meningeal lymphatic vessels mediate neurotropic viral drainage from the central nervous system. *Nat. Neurosci.* **25**, 577–587 (2022).

59. Yang, A. C. *et al.* A human brain vascular atlas reveals diverse mediators of Alzheimer's risk. *Nature* **603**, 885–892 (2022).

60. Fitzpatrick, Z. *et al.* Venous-plexus-associated lymphoid hubs support meningeal humoral immunity. *Nature* **628**, 612–619 (2024).

61. Da Mesquita, S. *et al.* Meningeal lymphatics affect microglia responses and anti-Aβ immunotherapy. *Nature* **593**, 255–260 (2021).

62. Louveau, A. *et al.* CNS lymphatic drainage and neuroinflammation are regulated by meningeal lymphatic vasculature. *Nat. Neurosci.* **21**, 1380–1391 (2018).

63. Da Mesquita, S., Fu, Z. & Kipnis, J. The Meningeal Lymphatic System: A New Player in Neurophysiology. *Neuron* **100**, 375–388 (2018).

64. Da Mesquita, S. *et al.* Functional aspects of meningeal lymphatics in ageing and Alzheimer's disease. *Nature* **560**, 185–191 (2018).

65. Butterfield, D. A. & Halliwell, B. Oxidative stress, dysfunctional glucose metabolism and Alzheimer disease. *Nat. Rev. Neurosci.* **20**, 148–160 (2019).

66. Long, J. M. & Holtzman, D. M. Alzheimer Disease: An Update on Pathobiology and Treatment Strategies. *Cell* **179**, 312–339 (2019).

67. Ryder, B. D., Wydorski, P. M., Hou, Z. & Joachimiak, L. A. Chaperoning shape-shifting tau in disease.

Trends Biochem. Sci. **47**, 301–313 (2022).

68. Mok, S.-A. *et al.* Mapping interactions with the chaperone network reveals factors that protect against tau aggregation. *Nat. Struct. Mol. Biol.* **25**, 384–393 (2018).

69. Koren, J., 3rd *et al.* Chaperone signalling complexes in Alzheimer's disease. *J. Cell. Mol. Med.* **13**, 619–630 (2009).

70. Saha, I. *et al.* The AAA+ chaperone VCP disaggregates Tau fibrils and generates aggregate seeds in a cellular system. *Nat. Commun.* **14**, 560 (2023).

71. Marsh, S. E. *et al.* The adaptive immune system restrains Alzheimer's disease pathogenesis by modulating microglial function. *Proceedings of the National Academy of Sciences* **113**, E1316–E1325 (2016).

72. Grayson, J. M. *et al.* T cell exhaustion is associated with cognitive status and amyloid accumulation in Alzheimer's disease. *Sci. Rep.* **13**, 15779 (2023).

73. Su, W. *et al.* CXCR6 orchestrates brain CD8+ T cell residency and limits mouse Alzheimer's disease pathology. *Nat. Immunol.* **24**, 1735–1747 (2023).

74. Abellanas, M. A., Purnapatre, M., Burgaletto, C. & Schwartz, M. Monocyte-derived macrophages act as reinforcements when microglia fall short in Alzheimer's disease. *Nat Neurosci* **28**, 436–445 (2025).

75. Brigas, H. C. *et al.* IL-17 triggers the onset of cognitive and synaptic deficits in early stages of Alzheimer's disease. *Cell Rep.* **36**, 109574 (2021).

76. Rosenzweig, N. *et al.* Sex-dependent APOE4 neutrophil-microglia interactions drive cognitive impairment in Alzheimer's disease. *Nat. Med.* (2024) doi:10.1038/s41591-024-03122-3.

77. Cipollini, V., Anrather, J., Orzi, F. & Iadecola, C. Th17 and Cognitive Impairment: Possible Mechanisms of Action. *Front. Neuroanat.* **13**, 95 (2019).

78. Criado-Marrero, M. *et al.* Hsp90 co-chaperones, FKBP52 and Aha1, promote tau pathogenesis in aged wild-type mice. *Acta Neuropathol Commun* **9**, 65 (2021).

79. Gorantla, N. V. & Chinnathambi, S. Tau Protein Squired by Molecular Chaperones During Alzheimer's Disease. *J. Mol. Neurosci.* **66**, 356–368 (2018).

80. Galea, I. The blood-brain barrier in systemic infection and inflammation. *Cell. Mol. Immunol.* **18**, 2489–

2501 (2021).

81. Jovanovic, M. *et al.* Immunogenetics. Dynamic profiling of the protein life cycle in response to pathogens. *Science* **347**, 1259038 (2015).

82. Emani, P. S. *et al.* Single-cell genomics and regulatory networks for 388 human brains. *Science* **384**, eadi5199 (2024).

83. Zeng, T., Spence, J. P., Mostafavi, H. & Pritchard, J. K. Bayesian estimation of gene constraint from an evolutionary model with gene features. *Nat. Genet.* **56**, 1632–1643 (2024).

84. Karczewski, K. J. *et al.* The mutational constraint spectrum quantified from variation in 141,456 humans. *Nature* **581**, 434–443 (2020).

85. Milacic, M. *et al.* The reactome pathway knowledgebase 2024. *Nucleic Acids Res.* **52**, D672–D678 (2024).

86. Koks, S., Pfaff, A. L., Bubb, V. J. & Quinn, J. P. Transcript variants of genes involved in neurodegeneration are differentially regulated by the APOE and MAPT haplotypes. *Genes (Basel)* **12**, 423 (2021).

87. Bowles, K. R. *et al.* 17q21.31 sub-haplotypes underlying H1-associated risk for Parkinson's disease are associated with LRRC37A/2 expression in astrocytes. *Mol. Neurodegener.* **17**, 48 (2022).

88. Edwards, T. L. *et al.* Genome-wide association study confirms SNPs in SNCA and the MAPT region as common risk factors for Parkinson disease. *Ann. Hum. Genet.* **74**, 97–109 (2010).

89. Hill-Burns, E. M. *et al.* Identification of a novel Parkinson's disease locus via stratified genome-wide association study. *BMC Genomics* **15**, 118 (2014).

90. Erro, R., Mencacci, N. E. & Bhatia, K. P. The emerging role of phosphodiesterases in movement disorders. *Mov. Disord.* **36**, 2225–2243 (2021).

91. Appenzeller, S. *et al.* Autosomal-dominant striatal degeneration is caused by a mutation in the phosphodiesterase 8B gene. *Am. J. Hum. Genet.* **86**, 83–87 (2010).

92. Azuma, R. *et al.* A novel mutation of PDE8B Gene in a Japanese family with autosomal-dominant striatal degeneration: JAPANESE ADSD FAMILY. *Mov. Disord.* **30**, 1964–1967 (2015).

93. Barsottini, O. G. P. *et al.* Familial striatal degeneration: New mutation and neuroimaging clues. *Neurology* **85**, 1816–1818 (2015).

94. Folci, A. *et al.* Loss of hnRNP K impairs synaptic plasticity in hippocampal neurons. *J. Neurosci.* **34**, 9088–9095 (2014).

95. Markram, H. *et al.* Interneurons of the neocortical inhibitory system. *Nat. Rev. Neurosci.* **5**, 793–807 (2004).

96. Druga, R., Salaj, M. & Al-Redouan, A. Parvalbumin - positive neurons in the neocortex: A review. *Physiol. Res.* **72**, S173–S191 (2023).

Referee #1:

REF1-1. Remarks to the Author	
The revision is extremely comprehensive and addressed the concerns that I described in detail in the original review.	
Response	N/A

REF1-2. Remarks on code availability	
The code is comprehensive and well documented. There is a README file that provides general instructions for installing the code.	
Response	N/A

Referee #2:

REF2-1. Definition of transcriptomic vulnerability	
The authors have substantially expanded the manuscript, providing improved rationale and analytical details that make the datasets and findings more accessible. While many of the main results recapitulate previous findings, this work will serve as a valuable resource for the community studying neurological and psychiatric disease. I have a few minor presentation issues should be addressed to improve clarity and interpretation:<br><br>1) The term "transcriptomic vulnerability" still requires clearer definition. I think the statement in the reviewer comments ("Shared transcriptomic vulnerability refers to the common patterns of gene expression perturbations observed across different brain diseases.") is a clearer description than any of the text in the manuscript.	
Response	We agree with the reviewer for this suggestion. We updated the **Main Text** as suggested.
Excerpt	"... We identify shared transcriptomic vulnerability, defined as overlapping patterns of gene expression changes across diseases, which point to common pathogenic mechanisms. These shared signatures provide insights into potential molecular mechanisms of early intervention that go beyond traditional disease boundaries. Furthermore, we find that shared transcriptomic vulnerability between disorders is concordant with genetic co-heritability, suggesting that common genetic risk factors partly drive shared gene expression changes."

REF2-2. Inclusion of variance explained in the main text	
2) The relationship between variance explained and baseline expression level is explored in Supplementary Figure 5h, but not referenced in the main text. This technical effect should be acknowledged in the main text, as it substantially affects interpretation of Figure 3b. The current framing that the analysis "prioritized several drivers of expression variation" is misleading since the approach is inherently biased toward highly expressed genes.	
Response	We thank the reviewer for the suggestion. We rephrased the analysis in the **Main Text** and acknowledged the relationship between variance explained and baseline expression level in the **Methods**.
Excerpt	"... We prioritized genes with the highest expression variation across variables (**Fig. 3b, Supplementary Figs. 6b-f**)."<br><br>"... To better interpret the variance partitioning analysis, we examined the relationship between variance explained and baseline gene expression across subclasses. Specifically, we plotted the proportion of variance attributed to donor, cell type, and residual components as a function of mean expression ($\log_2$ counts per million) (**Supplementary Fig. 5h**). Genes with higher expression showed greater variance explained by donor and cell type, and lower residual variance, reflecting a technical effect where higher read counts yield more precise estimates and thus bias the analysis toward highly expressed genes."

REF2-3. Limitation of the analysis
3) While interesting, the association between expression patterns and loss-of-function burden shows overall weak correlation significant in only two cell types (Supplementary Figure 8). This limitation should be acknowledged in the main text alongside the presentation of these results.

Response	We would like to clarify that **Supplementary Fig. 8e** shows an enrichment of rare-variant burden, not a correlation. However, we agree that two subclasses with statistically significant enrichments should be acknowledged. We have updated it in the **Main Text**.
Excerpt	"... Genes with disease-specific effects exhibited a higher burden of rare variants compared to those with shared effects (**Supplementary Fig. 8e**), with statistically significant enrichments observed in VIP$^+$ and PVALB$^+$ interneurons. While this supports the relevance of disease-specific components to core disease biology[1], the signal was limited to a subset of cell types."

REF2-4. Cross-disorder variation in gene expression and genetic concordance	
4) The expanded section on "cross-disorder variation in gene expression and genetic concordance" would benefit from additional discussion of implications. Key questions merit consideration: What insights does concordance provide? Should future studies focus on shared or distinct DE signatures? Why are cell types with higher concordance more relevant? These findings warrant an additional section in the discussion, in relation to previous neuro-genetics studies.	
Response	We updated the **Discussion** to clarify the implication of the concordance between shared transcriptomic vulnerability and genetic co-heritability.
Excerpt	"... The concordance between shared differential expression patterns and genetic co-heritability suggests that overlapping transcriptomic signatures reflect core biological processes influenced by pleiotropic risk variants. This concordance provides insight into molecular pathways that may be causally linked to disease etiology across disorders. Cell types with higher concordance, including deep-layer excitatory neurons and PVALB$^+$ Chandelier cells, likely represent critical sites of genetic risk convergence and may warrant prioritization in mechanistic studies. While disease-specific DE signatures remain important for understanding disorder-specific pathophysiology, shared signatures offer a window into fundamental processes contributing to comorbidity and pleiotropy."

REF2-5. Disease trajectory	
5) Regarding REF2-7: the comparison with PLS regression presented in suppl. Fig. 15 is not a fair comparison, since the linear model is not jointly trained on Dementia and Braak staging like the NN model is. It is to be expected that the two factors remain correlated. This comparison makes sense against a multi-factorial model but not a univariate model. At the very least, I would add to the text (in Methods) the arguments presented in the reviewer response under section "Possible use of a multi-factorial linear model"	
Response	We apologize for any confusion, as the PLS model was indeed jointly trained on both Braak and Dementia. As the reviewer suggested, we now state this explicitly in the **Methods**. However, there exists other possible reasons why dementia classification was lower than the neural network model. For example, training PLS regression on an inherently binary classification problem (for dementia) is not ideal. We now state this explicitly as a possible reason why PLS dementia accuracy was lower than the neural network model (see **Excerpt**).
Excerpt	In the Methods section describing PLS:<br><br>"... As with our neural network models, the PLS model was jointly trained to predict Braak and dementia across 20 train/test splits, … In contrast, the dementia classification accuracy was significantly lower ($P < 0.001$, bootstrap). However, we note that training a regression model on a binary classification task is not ideal, and improvements to this approach are possible."

REF2-6. Remarks on code availability	
While the code repository is substantially improved, I could still catch instances where I wasn't able to locate the code used for a specific analysis (e.g. comparison between NN and PLS model for disease pseudotime described above). I recommend that the authors to make sure the code repository is complete, ideally also including the code used to generate raw figures for all main and supplementary figures in the paper. Complete code availability, including scripts for generating all figures, would better serve the research community and reduce post-publication clarification requests.	
Response	Yes, we agree with the reviewer that our code repository was not fully up to date. We made sure the code repository is complete and added instructions where necessary. Specifically for the disease trajectory analysis, we have updated and cleaned up our code to fully document our analysis and figure generation (including the comparison between the PLS and neural network models). All analysis performed for the paper is found in PsychADxD/7_trajectory/analysis.py, and the worksheet showing how the figures for the paper were generated is found in PsychADxD/7_trajectory/generate_figures.ipynb.
Excerpt	https://github.com/DiseaseNeuroGenomics/PsychADxD

Referee #3:

REF3-1. Remarks to the Author	
The authors have thoroughly addressed my concerns. The revised manuscript is very impressive, and the study overall represents a major accomplishment and an important resource for the field. Congratulations!	
Response	N/A

REF3-2. Remarks on code availability	
The code repository Is comprehensive. During their revisions, the authors have made the code repository significantly more accessible and usable.	
Response	N/A

Reference

1. Weiner, D. J. *et al.* Polygenic architecture of rare coding variation across 394,783 exomes. *Nature* **614**, 492–499 (2023).
