# Supplementary material for: Single-cell atlas of transcriptomic vulnerability across brain disorders
Source: Nature. 2026 Sep 23;657(8133):988–1002. doi: 10.1038/s41586-025-09573-z (PMC13600946; doi:10.1038/s41586-025-09573-z)
Supplement: Supplementary file 3 [file 41586_2025_9573_MOESM1_ESM.zip › 41586_2025_9573_MOESM1_ESM/2024-07-15352B-s1/PsychAD_Supplementary_F10_v1.pdf]

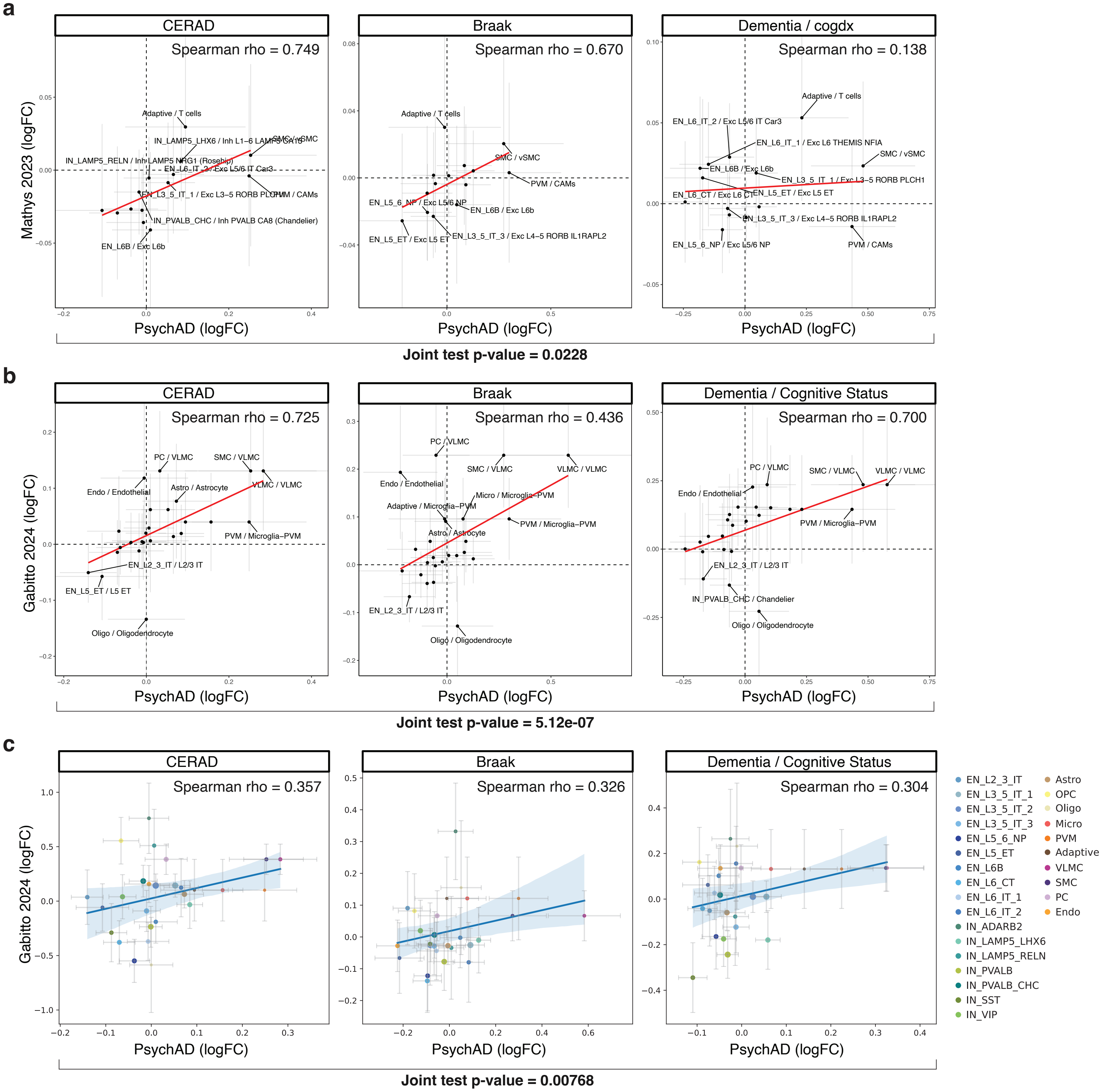

a
CERAD
Spearman rho = 0.749
Braak
Spearman rho = 0.670
Dementia / cogdx
Spearman rho = 0.138
Mathys 2023 (logFC)
PsychAD (logFC)
Adaptive / T cells
IN_LAMP5_LHX6 / Inh L1−6 LAMP5 CA13
SMC / vSMC
IN_LAMP5_RELN / Inh LAMP5 NRG1 (Rosehip)
EN_L6_IT_2 / Exc L5/6 IT Car3
EN_L3_5_IT_1 / Exc L3−5 RORB PLCH1
PVM / CAMs
IN_PVALB_CHC / Inh PVALB CA8 (Chandelier)
EN_L6B / Exc L6b
EN_L5_6_NP / Exc L5/6 NP
EN_L5_ET / Exc L5 ET
EN_L3_5_IT_3 / Exc L4−5 RORB IL1RAPL2
EN_L6_IT_1 / Exc L6 THEMIS NFIA
EN_L6_CT / Exc L6 CT
Joint test p-value = 0.0228
b
CERAD
Spearman rho = 0.725
Braak
Spearman rho = 0.436
Dementia / Cognitive Status
Spearman rho = 0.700
Gabitto 2024 (logFC)
PsychAD (logFC)
PC / VLMC
SMC / VLMC
VLMC / VLMC
Endo / Endothelial
Astro / Astrocyte
PVM / Microglia−PVM
Micro / Microglia−PVM
Adaptive / Microglia−PVM
EN_L2_3_IT / L2/3 IT
EN_L5_ET / L5 ET
Oligo / Oligodendrocyte
IN_PVALB_CHC / Chandelier
Joint test p-value = 5.12e-07
c
CERAD
Spearman rho = 0.357
Braak
Spearman rho = 0.326
Dementia / Cognitive Status
Spearman rho = 0.304
Gabitto 2024 (logFC)
PsychAD (logFC)
EN_L2_3_IT
EN_L3_5_IT_1
EN_L3_5_IT_2
EN_L3_5_IT_3
EN_L5_6_NP
EN_L5_ET
EN_L6B
EN_L6_CT
EN_L6_IT_1
EN_L6_IT_2
IN_ADARB2
IN_LAMP5_LHX6
IN_LAMP5_RELN
IN_PVALB
IN_PVALB_CHC
IN_SST
IN_VIP
Astro
OPC
Oligo
Micro
PVM
Adaptive
VLMC
SMC
PC
Endo
Joint test p-value = 0.00768
