# Supplementary material for: Single-cell atlas of transcriptomic vulnerability across brain disorders
Source: Nature. 2026 Sep 23;657(8133):988–1002. doi: 10.1038/s41586-025-09573-z (PMC13600946; doi:10.1038/s41586-025-09573-z)
Supplement: Supplementary file 3 [file 41586_2025_9573_MOESM1_ESM.zip › 41586_2025_9573_MOESM1_ESM/2024-07-15352B-s1/PsychAD_Supplementary_F11_v1.pdf]

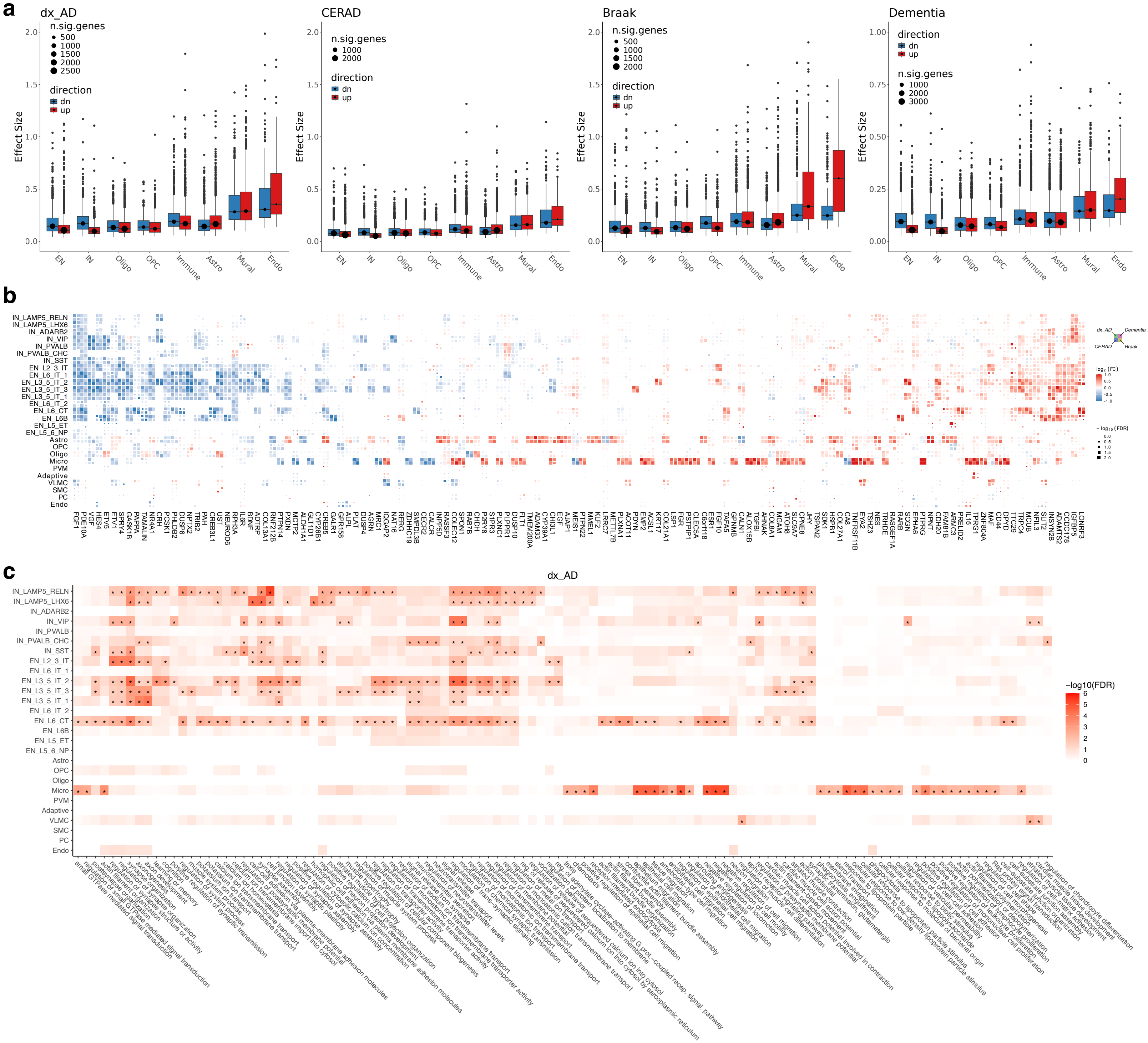

a
dx_AD
CERAD
Braak
Dementia
n.sig.genes
500
1000
1500
2000
2500
3000
direction
dn
up
Effect Size
EN
IN
Oligo
OPC
Immune
Astro
Mural
Endo
b
IN_LAMP5_RELN
IN_LAMP5_LHX6
IN_ADARB2
IN_VIP
IN_PVALB
IN_PVALB_CHC
IN_SST
EN_L2_3_IT
EN_L6_IT_1
EN_L3_5_IT_2
EN_L3_5_IT_3
EN_L3_5_IT_1
EN_L6_IT_2
EN_L6_CT
EN_L6B
EN_L5_ET
EN_L5_6_NP
Astro
OPC
Oligo
Micro
PVM
Adaptive
VLMC
SMC
PC
Endo
dx_AD
Dementia
CERAD
Braak
log2 (FC)
−log10 (FDR)
c
dx_AD
−log10(FDR)
