# Supplementary material for: Single-cell atlas of transcriptomic vulnerability across brain disorders
Source: Nature. 2026 Sep 23;657(8133):988–1002. doi: 10.1038/s41586-025-09573-z (PMC13600946; doi:10.1038/s41586-025-09573-z)
Supplement: Supplementary file 3 [file 41586_2025_9573_MOESM1_ESM.zip › 41586_2025_9573_MOESM1_ESM/2024-07-15352B-s1/PsychAD_Supplementary_F12_v1.pdf]

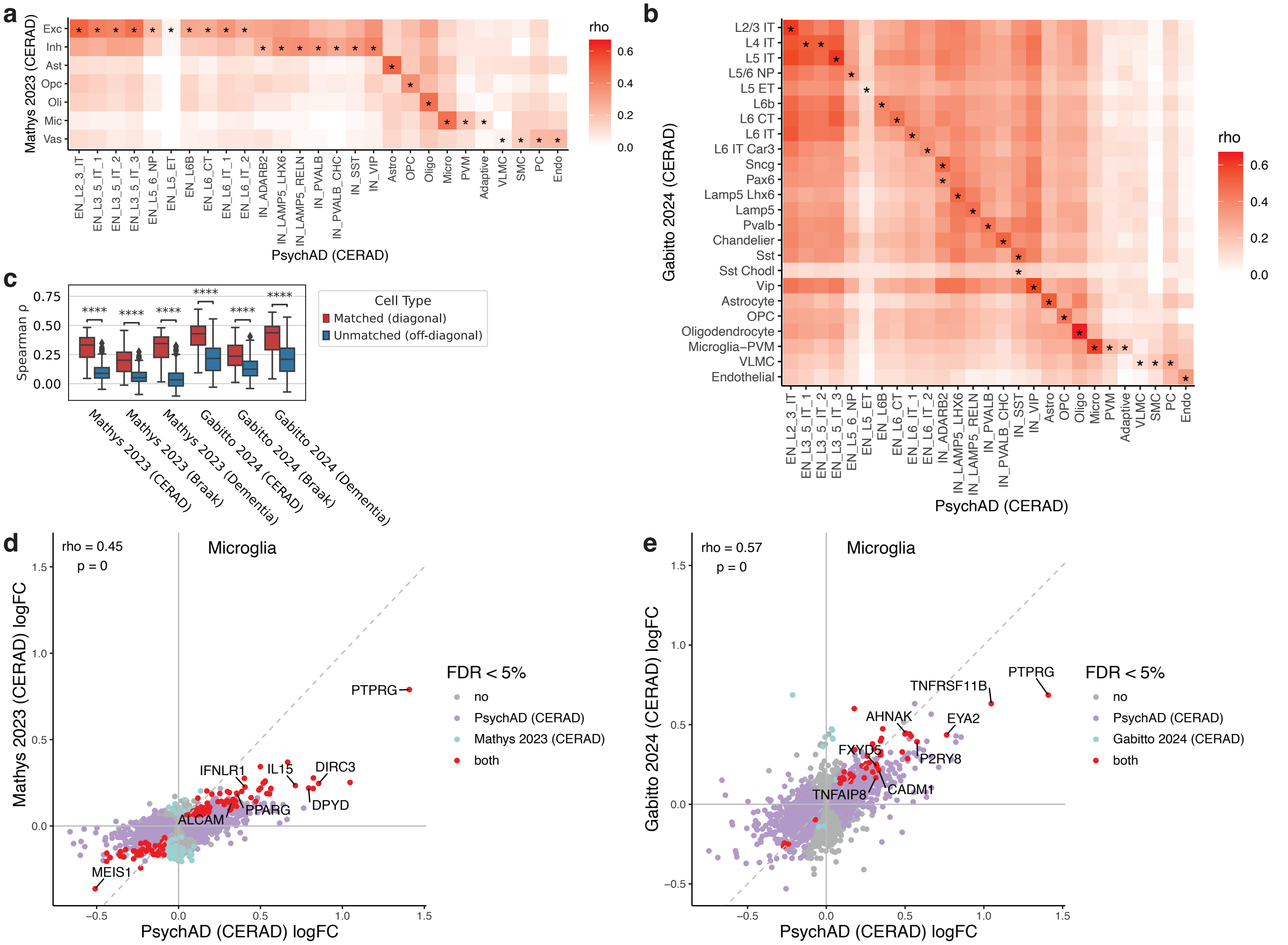
a
b
c
d
e
Mathys 2023 (CERAD)
PsychAD (CERAD)
Gabitto 2024 (CERAD)
rho
Spearman ρ
Cell Type
Matched (diagonal)
Unmatched (off-diagonal)
Mathys 2023 (CERAD)
Mathys 2023 (Braak)
Mathys 2023 (Dementia)
Gabitto 2024 (CERAD)
Gabitto 2024 (Braak)
Gabitto 2024 (Dementia)
rho = 0.45
p = 0
Microglia
Mathys 2023 (CERAD) logFC
PsychAD (CERAD) logFC
FDR < 5%
no
PsychAD (CERAD)
Mathys 2023 (CERAD)
both
PTPRG
IFNLR1
IL15
DIRC3
PPARG
DPYD
ALCAM
MEIS1
rho = 0.57
p = 0
Microglia
Gabitto 2024 (CERAD) logFC
PsychAD (CERAD) logFC
FDR < 5%
no
PsychAD (CERAD)
Gabitto 2024 (CERAD)
both
TNFRSF11B
PTPRG
AHNAK
EYA2
FXYD5
P2RY8
TNFAIP8
CADM1
