# Supplementary material for: Single-cell atlas of transcriptomic vulnerability across brain disorders
Source: Nature. 2026 Sep 23;657(8133):988–1002. doi: 10.1038/s41586-025-09573-z (PMC13600946; doi:10.1038/s41586-025-09573-z)
Supplement: Supplementary file 3 [file 41586_2025_9573_MOESM1_ESM.zip › 41586_2025_9573_MOESM1_ESM/2024-07-15352B-s1/PsychAD_Supplementary_F13_v1.pdf]

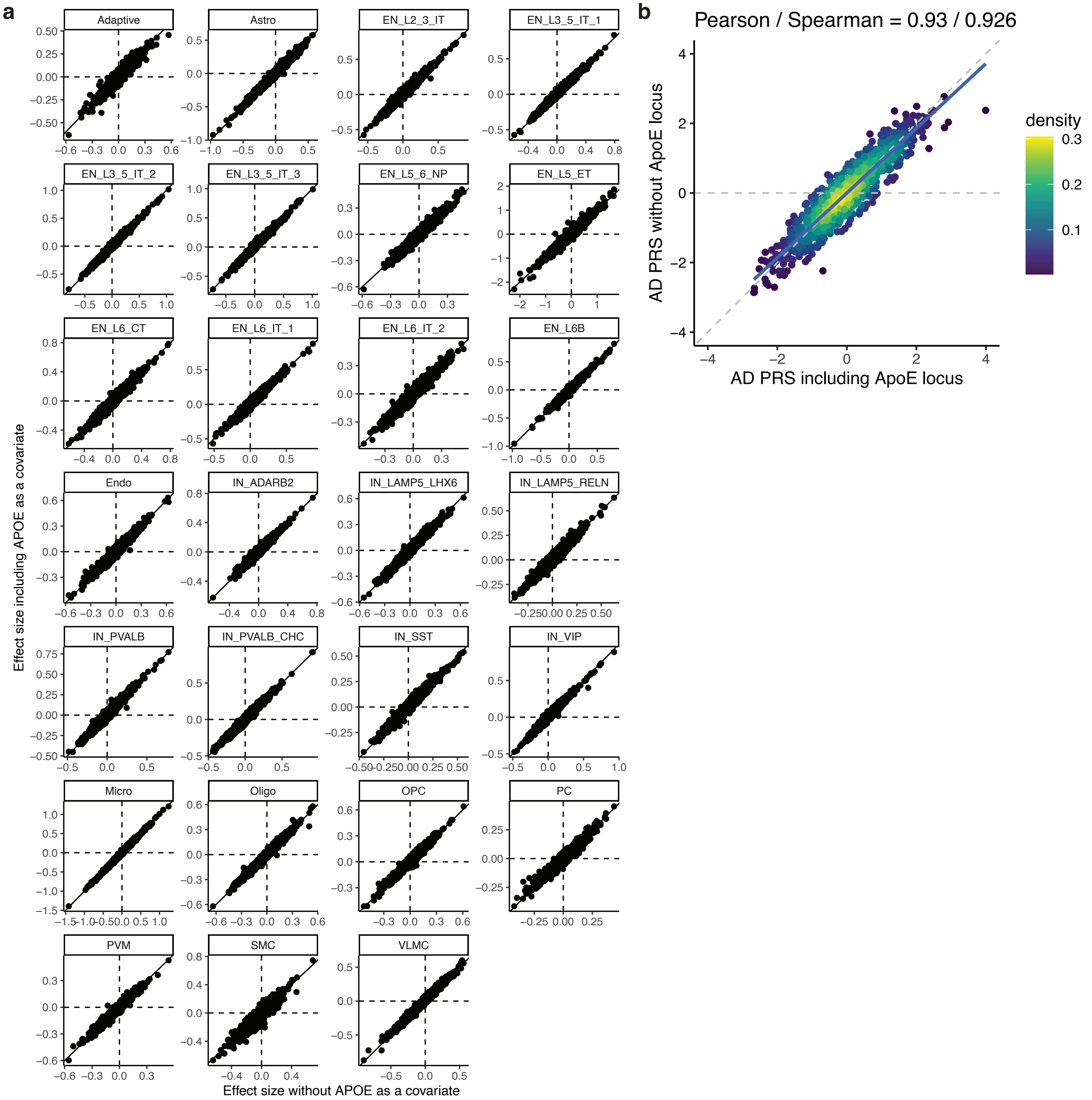

a
Adaptive
Astro
EN_L2_3_IT
EN_L3_5_IT_1
EN_L3_5_IT_2
EN_L3_5_IT_3
EN_L5_6_NP
EN_L5_ET
EN_L6_CT
EN_L6_IT_1
EN_L6_IT_2
EN_L6B
Endo
IN_ADARB2
IN_LAMP5_LHX6
IN_LAMP5_RELN
IN_PVALB
IN_PVALB_CHC
IN_SST
IN_VIP
Micro
Oligo
OPC
PC
PVM
SMC
VLMC
Effect size including APOE as a covariate
Effect size without APOE as a covariate
b
Pearson / Spearman = 0.93 / 0.926
AD PRS without ApoE locus
AD PRS including ApoE locus
density
0.3
0.2
0.1
