# Supplementary material for: Single-cell atlas of transcriptomic vulnerability across brain disorders
Source: Nature. 2026 Sep 23;657(8133):988–1002. doi: 10.1038/s41586-025-09573-z (PMC13600946; doi:10.1038/s41586-025-09573-z)
Supplement: Supplementary file 3 [file 41586_2025_9573_MOESM1_ESM.zip › 41586_2025_9573_MOESM1_ESM/2024-07-15352B-s1/PsychAD_Supplementary_F14_v1.pdf]

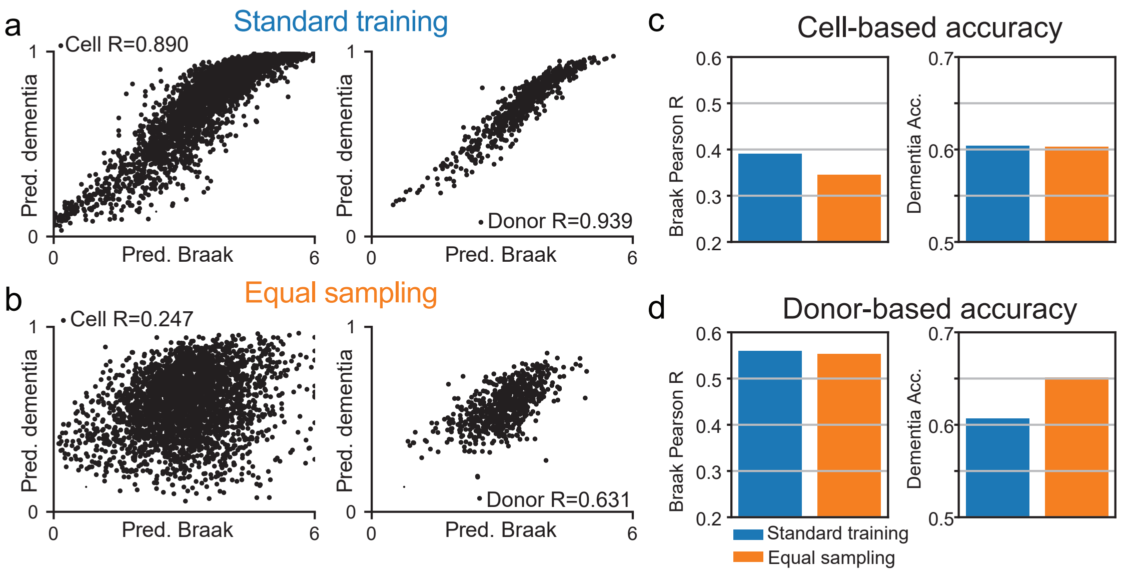
a
Standard training
Cell R=0.890
Pred. dementia
Pred. Braak
Donor R=0.939
b
Equal sampling
Cell R=0.247
Donor R=0.631
c
Cell-based accuracy
Braak Pearson R
Dementia Acc.
d
Donor-based accuracy
Standard training
Equal sampling
