# Supplementary material for: Single-cell atlas of transcriptomic vulnerability across brain disorders
Source: Nature. 2026 Sep 23;657(8133):988–1002. doi: 10.1038/s41586-025-09573-z (PMC13600946; doi:10.1038/s41586-025-09573-z)
Supplement: Supplementary file 3 [file 41586_2025_9573_MOESM1_ESM.zip › 41586_2025_9573_MOESM1_ESM/2024-07-15352B-s1/PsychAD_Supplementary_F15_v1.pdf]

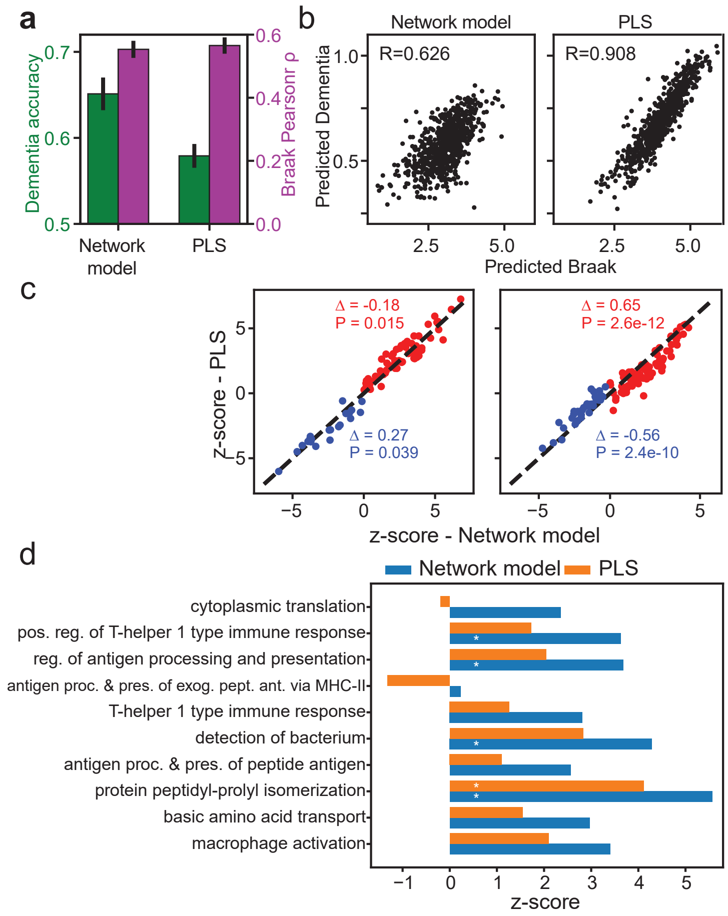
a
Dementia accuracy
Braak Pearsonr ρ
0.5
0.6
0.7
0.0
0.2
0.4
0.6
Network model
PLS
b
Network model
PLS
R=0.626
R=0.908
Predicted Dementia
1.0
0.5
2.5
5.0
Predicted Braak
c
Δ = -0.18
P = 0.015
Δ = 0.27
P = 0.039
Δ = 0.65
P = 2.6e-12
Δ = -0.56
P = 2.4e-10
z-score - PLS
z-score - Network model
d
Network model
PLS
cytoplasmic translation
pos. reg. of T-helper 1 type immune response
reg. of antigen processing and presentation
antigen proc. & pres. of exog. pept. ant. via MHC-II
T-helper 1 type immune response
detection of bacterium
antigen proc. & pres. of peptide antigen
protein peptidyl-prolyl isomerization
basic amino acid transport
macrophage activation
z-score
