# Supplementary material for: Single-cell atlas of transcriptomic vulnerability across brain disorders
Source: Nature. 2026 Sep 23;657(8133):988–1002. doi: 10.1038/s41586-025-09573-z (PMC13600946; doi:10.1038/s41586-025-09573-z)
Supplement: Supplementary file 3 [file 41586_2025_9573_MOESM1_ESM.zip › 41586_2025_9573_MOESM1_ESM/2024-07-15352B-s1/PsychAD_Supplementary_F17_v1.pdf]

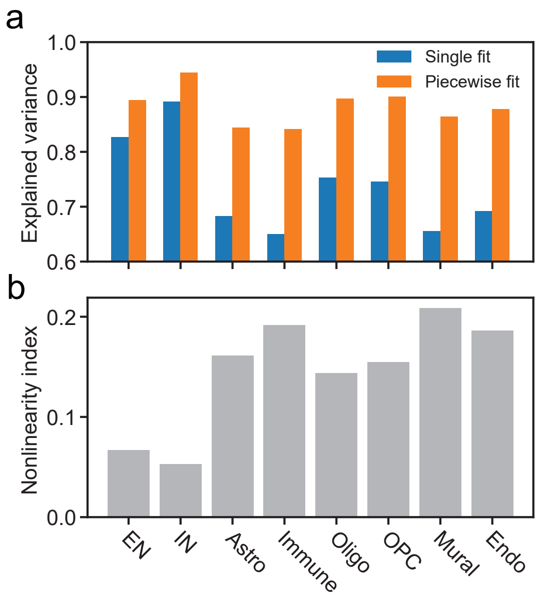
a
b
Explained variance
1.0
0.9
0.8
0.7
0.6
Single fit
Piecewise fit
Nonlinearity index
0.2
0.1
0.0
EN
IN
Astro
Immune
Oligo
OPC
Mural
Endo
