# Supplementary material for: Single-cell atlas of transcriptomic vulnerability across brain disorders
Source: Nature. 2026 Sep 23;657(8133):988–1002. doi: 10.1038/s41586-025-09573-z (PMC13600946; doi:10.1038/s41586-025-09573-z)
Supplement: Supplementary file 3 [file 41586_2025_9573_MOESM1_ESM.zip › 41586_2025_9573_MOESM1_ESM/2024-07-15352B-s1/PsychAD_Supplementary_F18_v1.pdf]

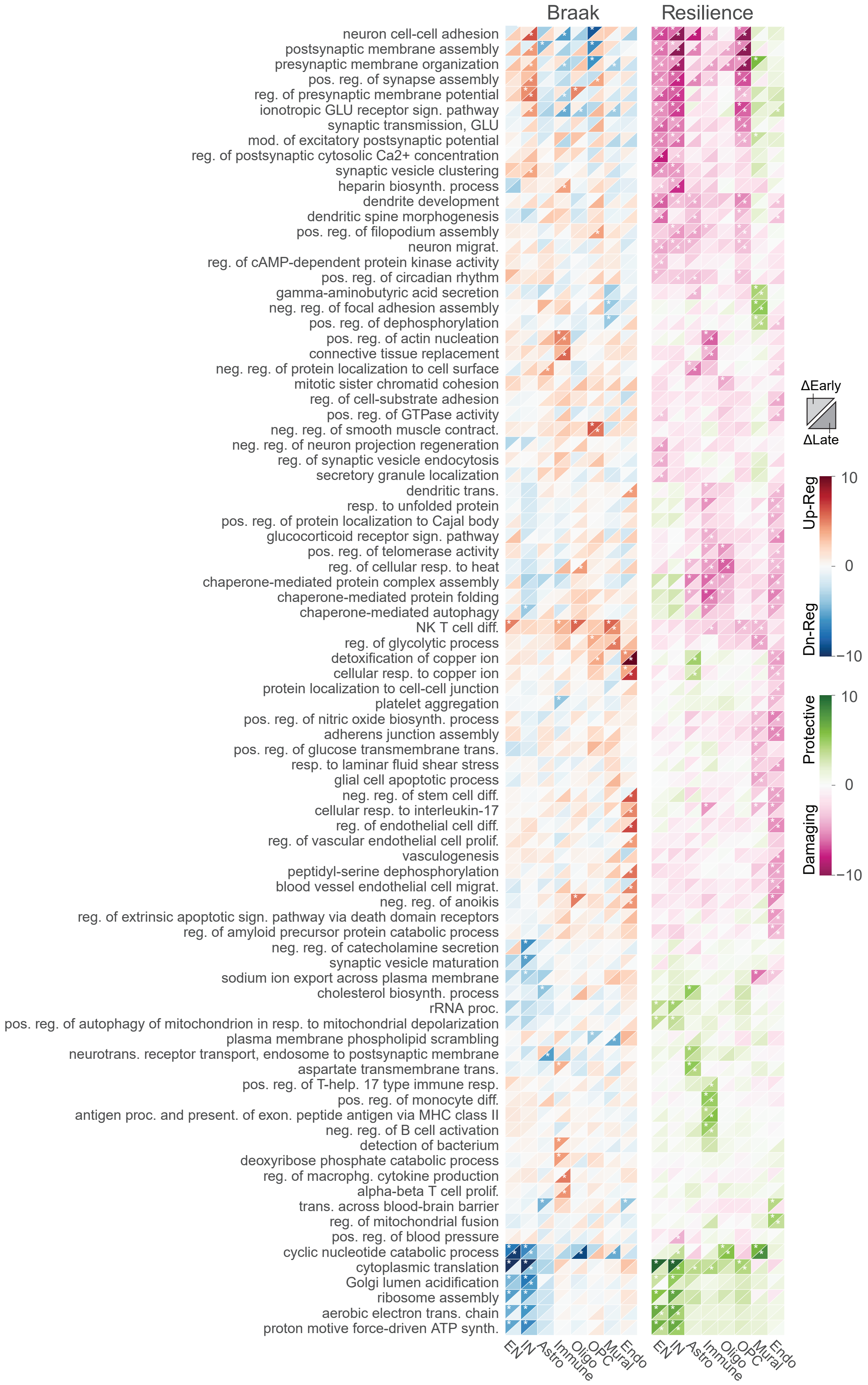
Braak
Resilience
neuron cell-cell adhesion
postsynaptic membrane assembly
presynaptic membrane organization
pos. reg. of synapse assembly
reg. of presynaptic membrane potential
ionotropic GLU receptor sign. pathway
synaptic transmission, GLU
mod. of excitatory postsynaptic potential
reg. of postsynaptic cytosolic Ca2+ concentration
synaptic vesicle clustering
heparin biosynth. process
dendrite development
dendritic spine morphogenesis
pos. reg. of filopodium assembly
neuron migrat.
reg. of cAMP-dependent protein kinase activity
pos. reg. of circadian rhythm
gamma-aminobutyric acid secretion
neg. reg. of focal adhesion assembly
pos. reg. of dephosphorylation
pos. reg. of actin nucleation
connective tissue replacement
neg. reg. of protein localization to cell surface
mitotic sister chromatid cohesion
reg. of cell-substrate adhesion
pos. reg. of GTPase activity
neg. reg. of smooth muscle contract.
neg. reg. of neuron projection regeneration
reg. of synaptic vesicle endocytosis
secretory granule localization
dendritic trans.
resp. to unfolded protein
pos. reg. of protein localization to Cajal body
glucocorticoid receptor sign. pathway
pos. reg. of telomerase activity
reg. of cellular resp. to heat
chaperone-mediated protein complex assembly
chaperone-mediated protein folding
chaperone-mediated autophagy
NK T cell diff.
reg. of glycolytic process
detoxification of copper ion
cellular resp. to copper ion
protein localization to cell-cell junction
platelet aggregation
pos. reg. of nitric oxide biosynth. process
adherens junction assembly
pos. reg. of glucose transmembrane trans.
resp. to laminar fluid shear stress
glial cell apoptotic process
neg. reg. of stem cell diff.
cellular resp. to interleukin-17
reg. of endothelial cell diff.
reg. of vascular endothelial cell prolif.
vasculogenesis
peptidyl-serine dephosphorylation
blood vessel endothelial cell migrat.
neg. reg. of anoikis
reg. of extrinsic apoptotic sign. pathway via death domain receptors
reg. of amyloid precursor protein catabolic process
neg. reg. of catecholamine secretion
synaptic vesicle maturation
sodium ion export across plasma membrane
cholesterol biosynth. process
rRNA proc.
pos. reg. of autophagy of mitochondrion in resp. to mitochondrial depolarization
plasma membrane phospholipid scrambling
neurotrans. receptor transport, endosome to postsynaptic membrane
aspartate transmembrane trans.
pos. reg. of T-help. 17 type immune resp.
pos. reg. of monocyte diff.
antigen proc. and present. of exon. peptide antigen via MHC class II
neg. reg. of B cell activation
detection of bacterium
deoxyribose phosphate catabolic process
reg. of macrophg. cytokine production
alpha-beta T cell prolif.
trans. across blood-brain barrier
reg. of mitochondrial fusion
pos. reg. of blood pressure
cyclic nucleotide catabolic process
cytoplasmic translation
Golgi lumen acidification
ribosome assembly
aerobic electron trans. chain
proton motive force-driven ATP synth.
EN IN Astro Immune Oligo OPC Mural Endo
EN IN Astro Immune Oligo OPC Mural Endo
ΔEarly
ΔLate
Up-Reg
Dn-Reg
10
0
−10
Protective
Damaging
10
0
−10
