# Supplementary material for: Single-cell atlas of transcriptomic vulnerability across brain disorders
Source: Nature. 2026 Sep 23;657(8133):988–1002. doi: 10.1038/s41586-025-09573-z (PMC13600946; doi:10.1038/s41586-025-09573-z)
Supplement: Supplementary file 3 [file 41586_2025_9573_MOESM1_ESM.zip › 41586_2025_9573_MOESM1_ESM/2024-07-15352B-s1/PsychAD_Supplementary_F19_v1.pdf]

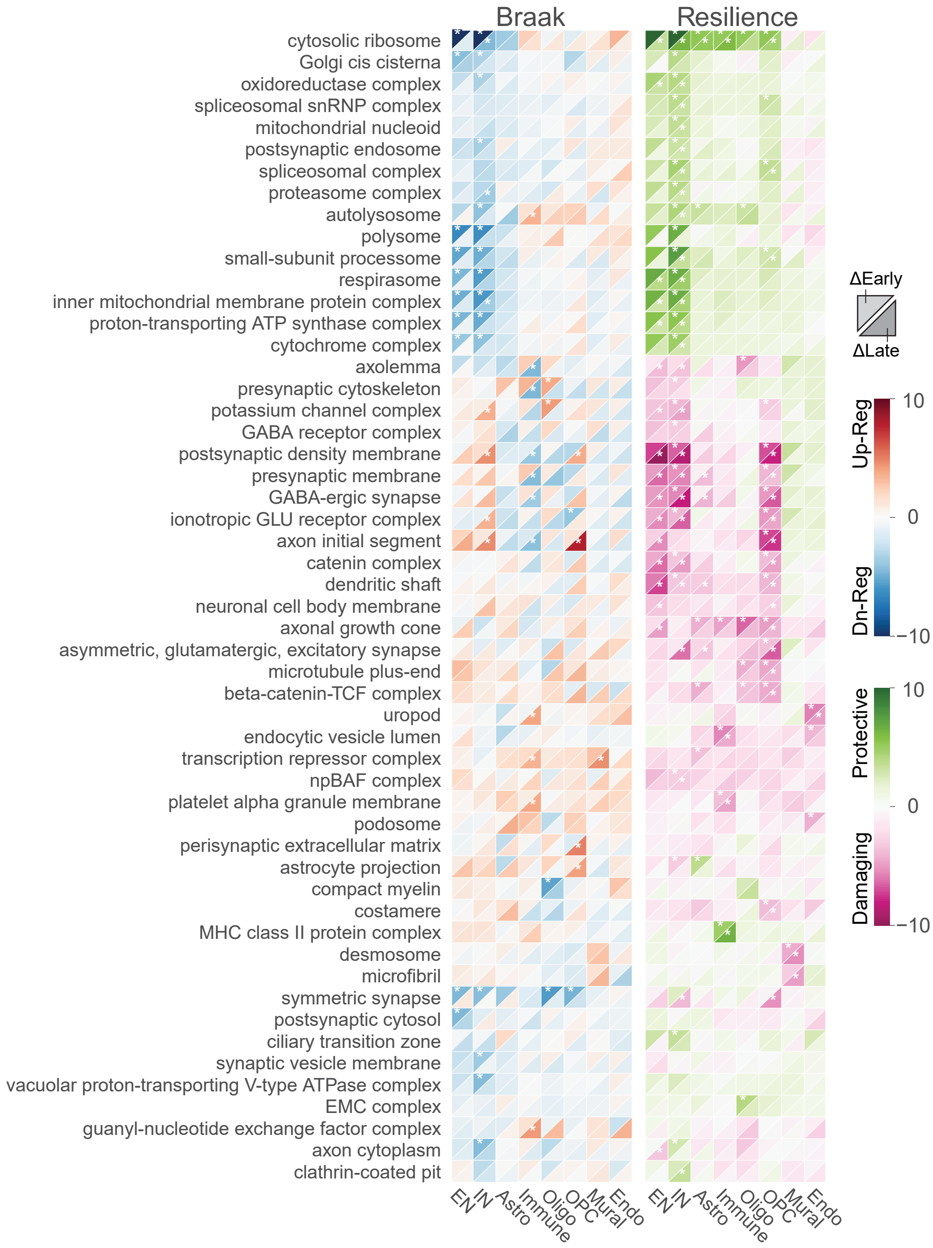

Braak
Resilience
cytosolic ribosome
Golgi cis cisterna
oxidoreductase complex
spliceosomal snRNP complex
mitochondrial nucleoid
postsynaptic endosome
spliceosomal complex
proteasome complex
autolysosome
polysome
small-subunit processome
respirasome
inner mitochondrial membrane protein complex
proton-transporting ATP synthase complex
cytochrome complex
axolemma
presynaptic cytoskeleton
potassium channel complex
GABA receptor complex
postsynaptic density membrane
presynaptic membrane
GABA-ergic synapse
ionotropic GLU receptor complex
axon initial segment
catenin complex
dendritic shaft
neuronal cell body membrane
axonal growth cone
asymmetric, glutamatergic, excitatory synapse
microtubule plus-end
beta-catenin-TCF complex
uropod
endocytic vesicle lumen
transcription repressor complex
npBAF complex
platelet alpha granule membrane
podosome
perisynaptic extracellular matrix
astrocyte projection
compact myelin
costamere
MHC class II protein complex
desmosome
microfibril
symmetric synapse
postsynaptic cytosol
ciliary transition zone
synaptic vesicle membrane
vacuolar proton-transporting V-type ATPase complex
EMC complex
guanyl-nucleotide exchange factor complex
axon cytoplasm
clathrin-coated pit
EN
IN
Astro
Immune
Oligo
OPC
Mural
Endo
EN
IN
Astro
Immune
Oligo
OPC
Mural
Endo
ΔEarly
ΔLate
Up-Reg
Dn-Reg
10
0
−10
Protective
Damaging
10
0
−10
