# Supplementary material for: Single-cell atlas of transcriptomic vulnerability across brain disorders
Source: Nature. 2026 Sep 23;657(8133):988–1002. doi: 10.1038/s41586-025-09573-z (PMC13600946; doi:10.1038/s41586-025-09573-z)
Supplement: Supplementary file 3 [file 41586_2025_9573_MOESM1_ESM.zip › 41586_2025_9573_MOESM1_ESM/2024-07-15352B-s1/PsychAD_Supplementary_F1_v1.pdf]

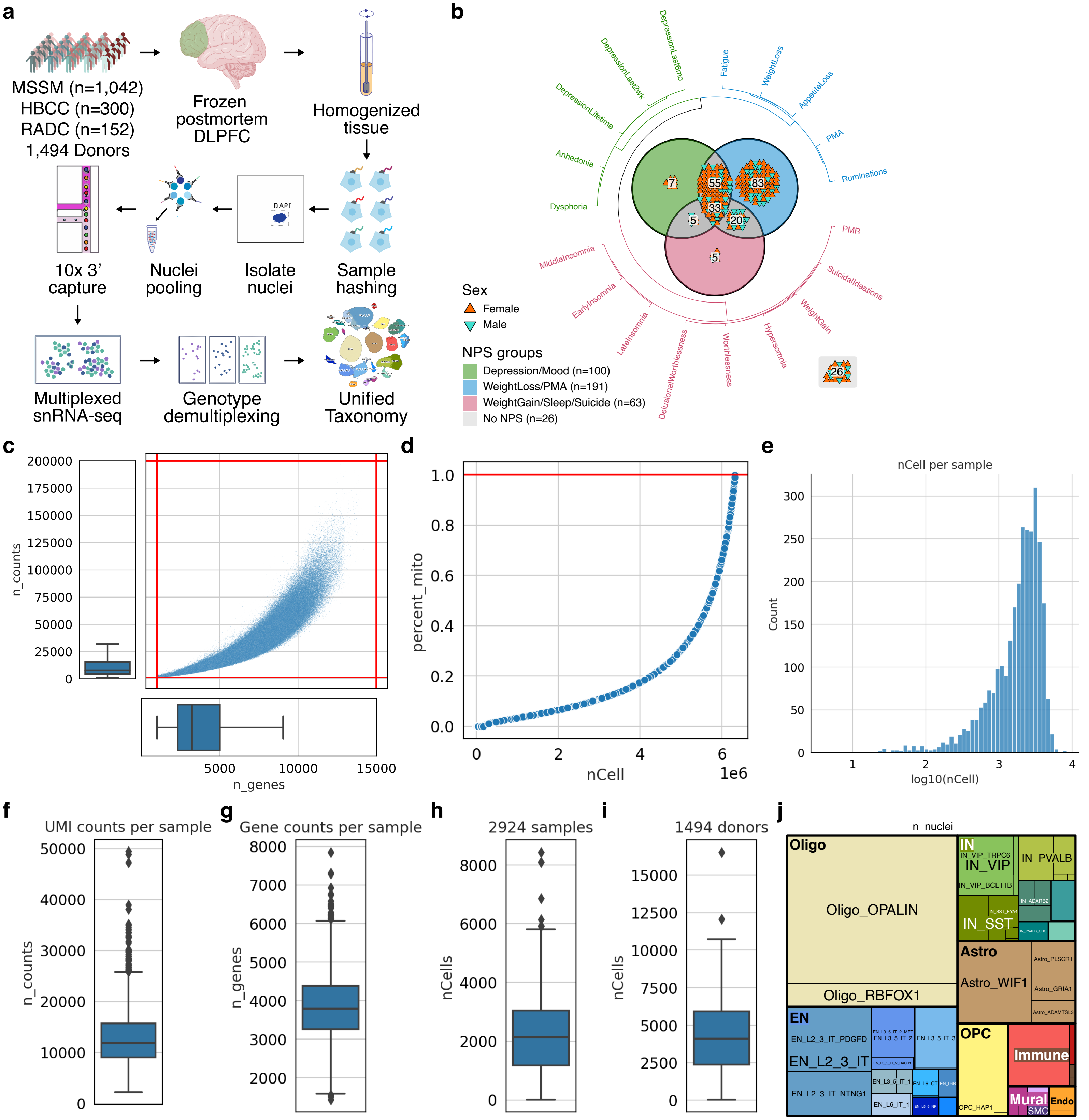

a
MSSM (n=1,042)
HBCC (n=300)
RADC (n=152)
1,494 Donors
Frozen postmortem DLPFC
Homogenized tissue
Sample hashing
Isolate nuclei
DAPI
Nuclei pooling
10x 3' capture
Multiplexed snRNA-seq
Genotype demultiplexing
Unified Taxonomy
b
Sex
Female
Male
NPS groups
Depression/Mood (n=100)
WeightLoss/PMA (n=191)
WeightGain/Sleep/Suicide (n=63)
No NPS (n=26)
DepressionLast6mo
DepressionLast2wk
DepressionLifetime
Anhedonia
Dysphoria
Fatigue
WeightLoss
AppetiteLoss
PMA
Ruminations
PMR
Suicidalideations
WeightGain
Hypersomnia
Worthlessness
DelusionalWorthlessness
LateInsomnia
EarlyInsomnia
MiddleInsomnia
c
n_counts
n_genes
d
percent_mito
nCell
1e6
e
nCell per sample
Count
log10(nCell)
f
UMI counts per sample
n_counts
g
Gene counts per sample
n_genes
h
2924 samples
nCells
i
1494 donors
nCells
j
n_nuclei
Oligo
Oligo_OPALIN
Oligo_RBFOX1
IN
IN_VIP
IN_PVALB
IN_SST
Astro
Astro_WIF1
EN
EN_L2_3_IT
EN_L2_3_IT_PDGFD
EN_L2_3_IT_NTNG1
OPC
Immune
Mural
Endo
SMC
