# Supplementary material for: Single-cell atlas of transcriptomic vulnerability across brain disorders
Source: Nature. 2026 Sep 23;657(8133):988–1002. doi: 10.1038/s41586-025-09573-z (PMC13600946; doi:10.1038/s41586-025-09573-z)
Supplement: Supplementary file 3 [file 41586_2025_9573_MOESM1_ESM.zip › 41586_2025_9573_MOESM1_ESM/2024-07-15352B-s1/PsychAD_Supplementary_F20_v1.pdf]

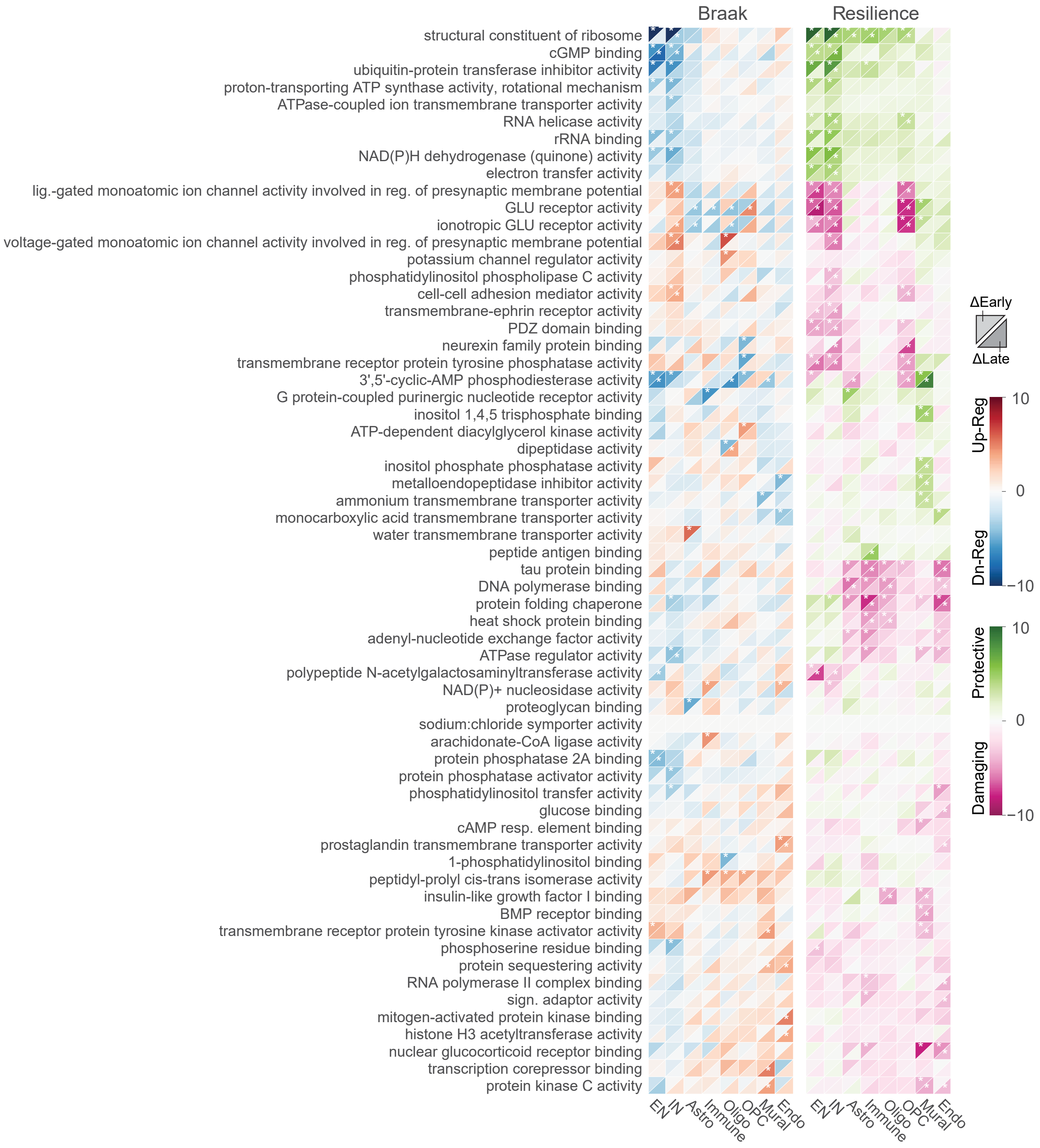
Braak
Resilience
structural constituent of ribosome
cGMP binding
ubiquitin-protein transferase inhibitor activity
proton-transporting ATP synthase activity, rotational mechanism
ATPase-coupled ion transmembrane transporter activity
RNA helicase activity
rRNA binding
NAD(P)H dehydrogenase (quinone) activity
electron transfer activity
lig.-gated monoatomic ion channel activity involved in reg. of presynaptic membrane potential
GLU receptor activity
ionotropic GLU receptor activity
voltage-gated monoatomic ion channel activity involved in reg. of presynaptic membrane potential
potassium channel regulator activity
phosphatidylinositol phospholipase C activity
cell-cell adhesion mediator activity
transmembrane-ephrin receptor activity
PDZ domain binding
neurexin family protein binding
transmembrane receptor protein tyrosine phosphatase activity
3',5'-cyclic-AMP phosphodiesterase activity
G protein-coupled purinergic nucleotide receptor activity
inositol 1,4,5 trisphosphate binding
ATP-dependent diacylglycerol kinase activity
dipeptidase activity
inositol phosphate phosphatase activity
metalloendopeptidase inhibitor activity
ammonium transmembrane transporter activity
monocarboxylic acid transmembrane transporter activity
water transmembrane transporter activity
peptide antigen binding
tau protein binding
DNA polymerase binding
protein folding chaperone
heat shock protein binding
adenyl-nucleotide exchange factor activity
ATPase regulator activity
polypeptide N-acetylgalactosaminyltransferase activity
NAD(P)+ nucleosidase activity
proteoglycan binding
sodium:chloride symporter activity
arachidonate-CoA ligase activity
protein phosphatase 2A binding
protein phosphatase activator activity
phosphatidylinositol transfer activity
glucose binding
cAMP resp. element binding
prostaglandin transmembrane transporter activity
1-phosphatidylinositol binding
peptidyl-prolyl cis-trans isomerase activity
insulin-like growth factor I binding
BMP receptor binding
transmembrane receptor protein tyrosine kinase activator activity
phosphoserine residue binding
protein sequestering activity
RNA polymerase II complex binding
sign. adaptor activity
mitogen-activated protein kinase binding
histone H3 acetyltransferase activity
nuclear glucocorticoid receptor binding
transcription corepressor binding
protein kinase C activity
EN IN Astro Immune Oligo OPC Mural Endo
EN IN Astro Immune Oligo OPC Mural Endo
ΔEarly
ΔLate
Up-Reg
Dn-Reg
10
0
−10
Protective
Damaging
10
0
−10
