# Supplementary material for: Single-cell atlas of transcriptomic vulnerability across brain disorders
Source: Nature. 2026 Sep 23;657(8133):988–1002. doi: 10.1038/s41586-025-09573-z (PMC13600946; doi:10.1038/s41586-025-09573-z)
Supplement: Supplementary file 3 [file 41586_2025_9573_MOESM1_ESM.zip › 41586_2025_9573_MOESM1_ESM/2024-07-15352B-s1/PsychAD_Supplementary_F21_v1.pdf]

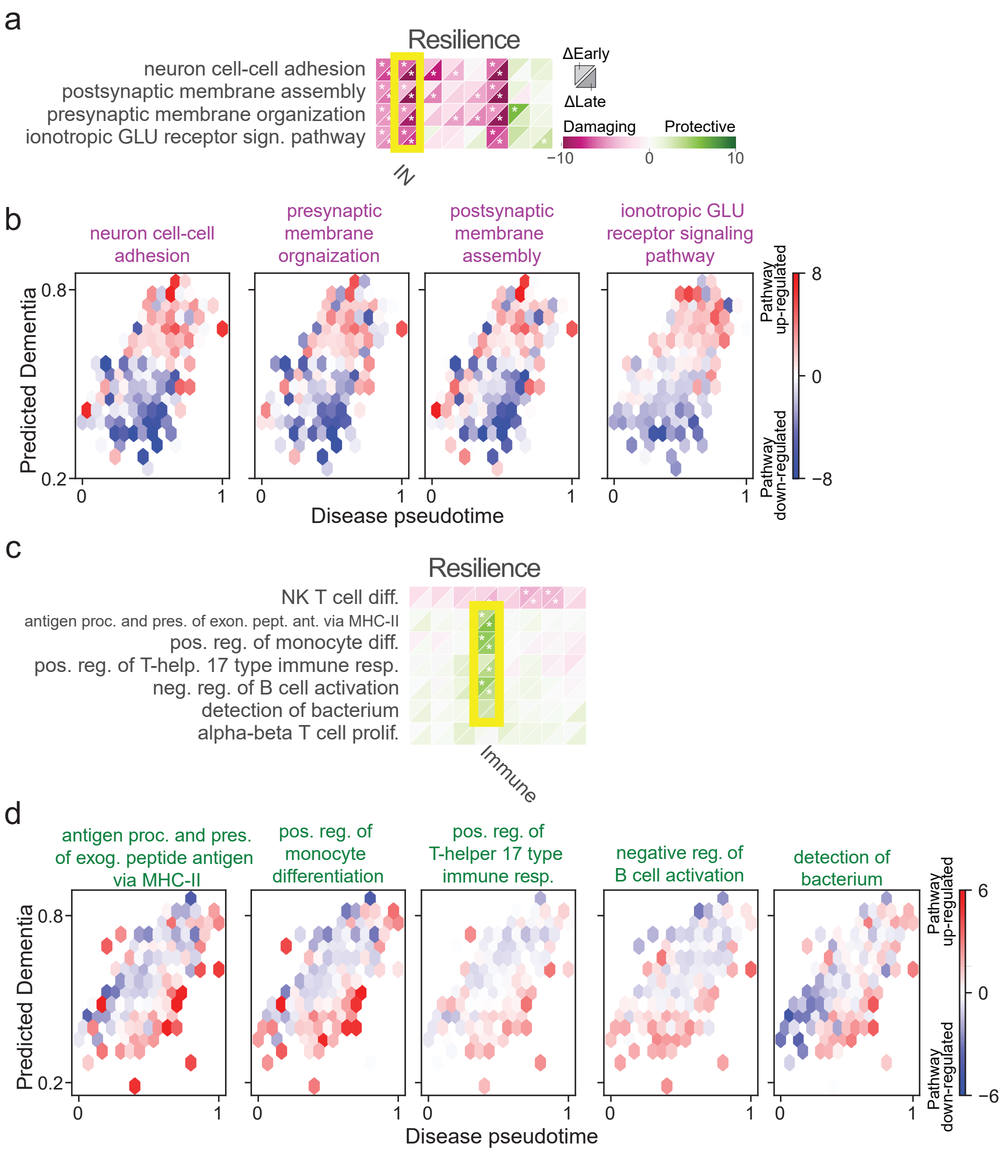

a
Resilience
neuron cell-cell adhesion
postsynaptic membrane assembly
presynaptic membrane organization
ionotropic GLU receptor sign. pathway
IN
ΔEarly
ΔLate
Damaging
Protective
−10
0
10
b
neuron cell-cell adhesion
presynaptic membrane orgnaization
postsynaptic membrane assembly
ionotropic GLU receptor signaling pathway
Predicted Dementia
0.8
0.2
0
1
Disease pseudotime
Pathway up-regulated
Pathway down-regulated
8
0
−8
c
Resilience
NK T cell diff.
antigen proc. and pres. of exon. pept. ant. via MHC-II
pos. reg. of monocyte diff.
pos. reg. of T-help. 17 type immune resp.
neg. reg. of B cell activation
detection of bacterium
alpha-beta T cell prolif.
Immune
d
antigen proc. and pres. of exog. peptide antigen via MHC-II
pos. reg. of monocyte differentiation
pos. reg. of T-helper 17 type immune resp.
negative reg. of B cell activation
detection of bacterium
Predicted Dementia
0.8
0.2
0
1
Disease pseudotime
Pathway up-regulated
Pathway down-regulated
6
0
−6
