# Supplementary material for: Single-cell atlas of transcriptomic vulnerability across brain disorders
Source: Nature. 2026 Sep 23;657(8133):988–1002. doi: 10.1038/s41586-025-09573-z (PMC13600946; doi:10.1038/s41586-025-09573-z)
Supplement: Supplementary file 3 [file 41586_2025_9573_MOESM1_ESM.zip › 41586_2025_9573_MOESM1_ESM/2024-07-15352B-s1/PsychAD_Supplementary_F22_v1.pdf]

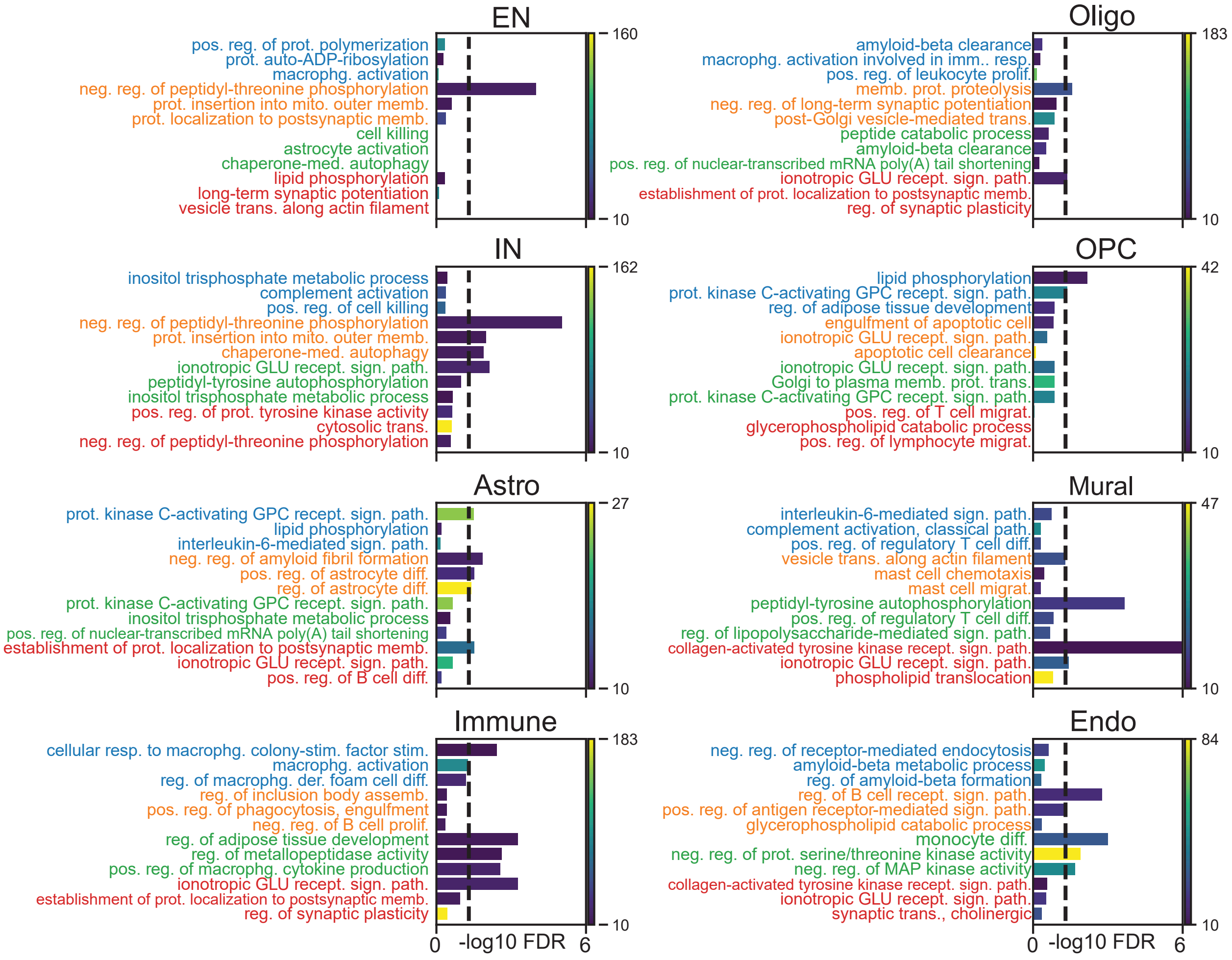

EN
pos. reg. of prot. polymerization
prot. auto-ADP-ribosylation
macrophg. activation
neg. reg. of peptidyl-threonine phosphorylation
prot. insertion into mito. outer memb.
prot. localization to postsynaptic memb.
cell killing
astrocyte activation
chaperone-med. autophagy
lipid phosphorylation
long-term synaptic potentiation
vesicle trans. along actin filament
160
10
Oligo
amyloid-beta clearance
macrophg. activation involved in imm.. resp.
pos. reg. of leukocyte prolif.
memb. prot. proteolysis
neg. reg. of long-term synaptic potentiation
post-Golgi vesicle-mediated trans.
peptide catabolic process
amyloid-beta clearance
pos. reg. of nuclear-transcribed mRNA poly(A) tail shortening
ionotropic GLU recept. sign. path.
establishment of prot. localization to postsynaptic memb.
reg. of synaptic plasticity
183
10
IN
inositol trisphosphate metabolic process
complement activation
pos. reg. of cell killing
neg. reg. of peptidyl-threonine phosphorylation
prot. insertion into mito. outer memb.
chaperone-med. autophagy
ionotropic GLU recept. sign. path.
peptidyl-tyrosine autophosphorylation
inositol trisphosphate metabolic process
pos. reg. of prot. tyrosine kinase activity
cytosolic trans.
neg. reg. of peptidyl-threonine phosphorylation
162
10
OPC
lipid phosphorylation
prot. kinase C-activating GPC recept. sign. path.
reg. of adipose tissue development
engulfment of apoptotic cell
ionotropic GLU recept. sign. path.
apoptotic cell clearance
ionotropic GLU recept. sign. path.
Golgi to plasma memb. prot. trans.
prot. kinase C-activating GPC recept. sign. path.
pos. reg. of T cell migrat.
glycerophospholipid catabolic process
pos. reg. of lymphocyte migrat.
42
10
Astro
prot. kinase C-activating GPC recept. sign. path.
lipid phosphorylation
interleukin-6-mediated sign. path.
neg. reg. of amyloid fibril formation
pos. reg. of astrocyte diff.
reg. of astrocyte diff.
prot. kinase C-activating GPC recept. sign. path.
inositol trisphosphate metabolic process
pos. reg. of nuclear-transcribed mRNA poly(A) tail shortening
establishment of prot. localization to postsynaptic memb.
ionotropic GLU recept. sign. path.
pos. reg. of B cell diff.
27
10
Mural
interleukin-6-mediated sign. path.
complement activation, classical path.
pos. reg. of regulatory T cell diff.
vesicle trans. along actin filament
mast cell chemotaxis
mast cell migrat.
peptidyl-tyrosine autophosphorylation
pos. reg. of regulatory T cell diff.
reg. of lipopolysaccharide-mediated sign. path.
collagen-activated tyrosine kinase recept. sign. path.
ionotropic GLU recept. sign. path.
phospholipid translocation
47
10
Immune
cellular resp. to macrophg. colony-stim. factor stim.
macrophg. activation
reg. of macrophg. der. foam cell diff.
reg. of inclusion body assemb.
pos. reg. of phagocytosis, engulfment
neg. reg. of B cell prolif.
reg. of adipose tissue development
reg. of metallopeptidase activity
pos. reg. of macrophg. cytokine production
ionotropic GLU recept. sign. path.
establishment of prot. localization to postsynaptic memb.
reg. of synaptic plasticity
183
10
0
-log10 FDR
6
Endo
neg. reg. of receptor-mediated endocytosis
amyloid-beta metabolic process
reg. of amyloid-beta formation
reg. of B cell recept. sign. path.
pos. reg. of antigen receptor-mediated sign. path.
glycerophospholipid catabolic process
monocyte diff.
neg. reg. of prot. serine/threonine kinase activity
neg. reg. of MAP kinase activity
collagen-activated tyrosine kinase recept. sign. path.
ionotropic GLU recept. sign. path.
synaptic trans., cholinergic
84
10
0
-log10 FDR
6
