# Supplementary material for: Single-cell atlas of transcriptomic vulnerability across brain disorders
Source: Nature. 2026 Sep 23;657(8133):988–1002. doi: 10.1038/s41586-025-09573-z (PMC13600946; doi:10.1038/s41586-025-09573-z)

a

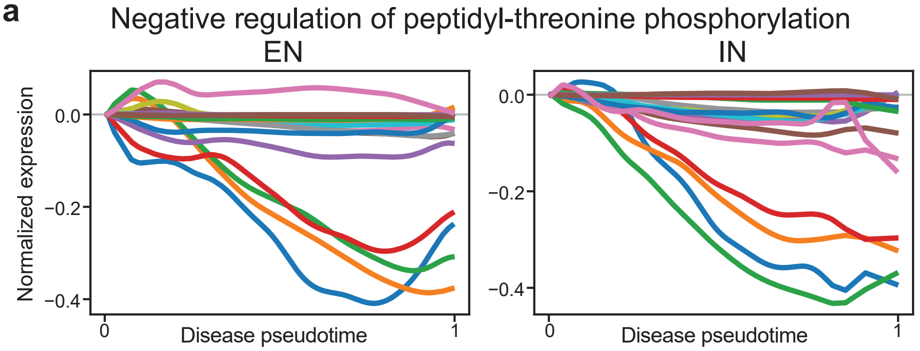

b

Gene	AD GWAS	EN Early Decrease Pct.	IN Early Decrease Pct.
CALM1	0.102	99.9%	99.9%
SPRED1	0.143	99.9%	99.4%
SPRED2	0.0008	99.8%	99.9%
CALM3	0.031	99.4%	99.5%
DMTN	0.029	89.9%	79.7%
SPRY2	0.451	87.2%	89.3%
CADM4	0.059	81.0%	94.3%
DGKQ	4.19e-07	78.1%	75.9%
PPP2R5D	0.341	75.1%	69.8%
EIF4G1	0.006	70.9%	80.8%
SIRT2	0.881	70.4%	71.6%
SMAD7	0.171	48.5%	42.9%
INPP5K	0.009	48.3%	47.4%
TTC36	0.741	39.8%	39.9%
CALM2	0.697	38.6%	15.9%
PPEF2	0.137	17.7%	7.2%
PARD3	0.068	8.0%	86.9%

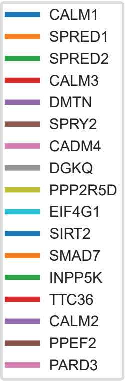

Supplement: Supplementary file 3 [file 41586_2025_9573_MOESM1_ESM.zip › 41586_2025_9573_MOESM1_ESM/2024-07-15352B-s1/PsychAD_Supplementary_F23_v1.pdf]
