# Supplementary material for: Single-cell atlas of transcriptomic vulnerability across brain disorders
Source: Nature. 2026 Sep 23;657(8133):988–1002. doi: 10.1038/s41586-025-09573-z (PMC13600946; doi:10.1038/s41586-025-09573-z)
Supplement: Supplementary file 3 [file 41586_2025_9573_MOESM1_ESM.zip › 41586_2025_9573_MOESM1_ESM/2024-07-15352B-s1/PsychAD_Supplementary_F24_v1.pdf]

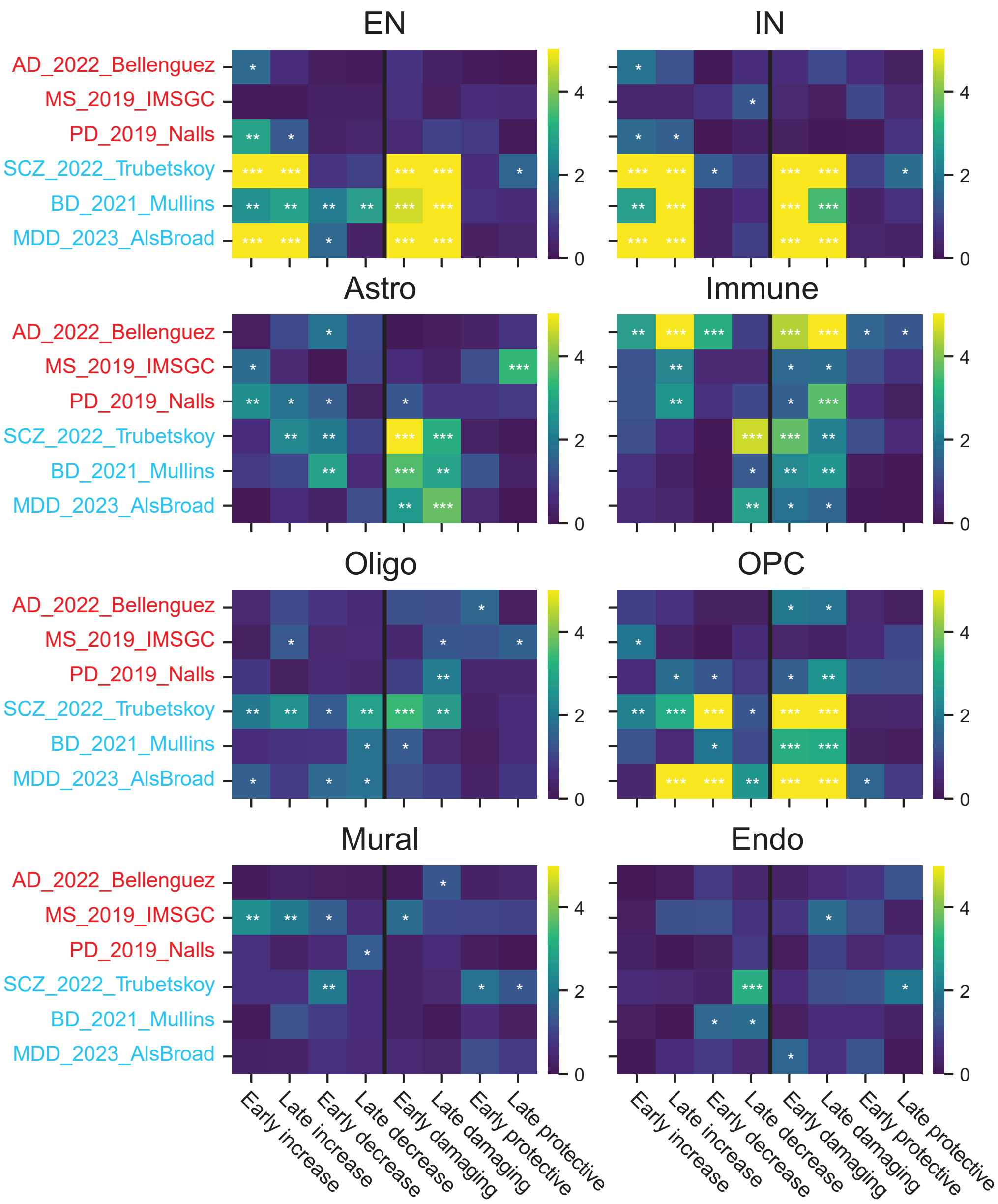
EN
IN
Astro
Immune
Oligo
OPC
Mural
Endo
AD_2022_Bellenguez
MS_2019_IMSGC
PD_2019_Nalls
SCZ_2022_Trubetskoy
BD_2021_Mullins
MDD_2023_AlsBroad
Early increase
Late increase
Early decrease
Late decrease
Early damaging
Late damaging
Early protective
Late protective
0
2
4
