# Supplementary material for: Single-cell atlas of transcriptomic vulnerability across brain disorders
Source: Nature. 2026 Sep 23;657(8133):988–1002. doi: 10.1038/s41586-025-09573-z (PMC13600946; doi:10.1038/s41586-025-09573-z)
Supplement: Supplementary file 3 [file 41586_2025_9573_MOESM1_ESM.zip › 41586_2025_9573_MOESM1_ESM/2024-07-15352B-s1/PsychAD_Supplementary_F25_v1.pdf]

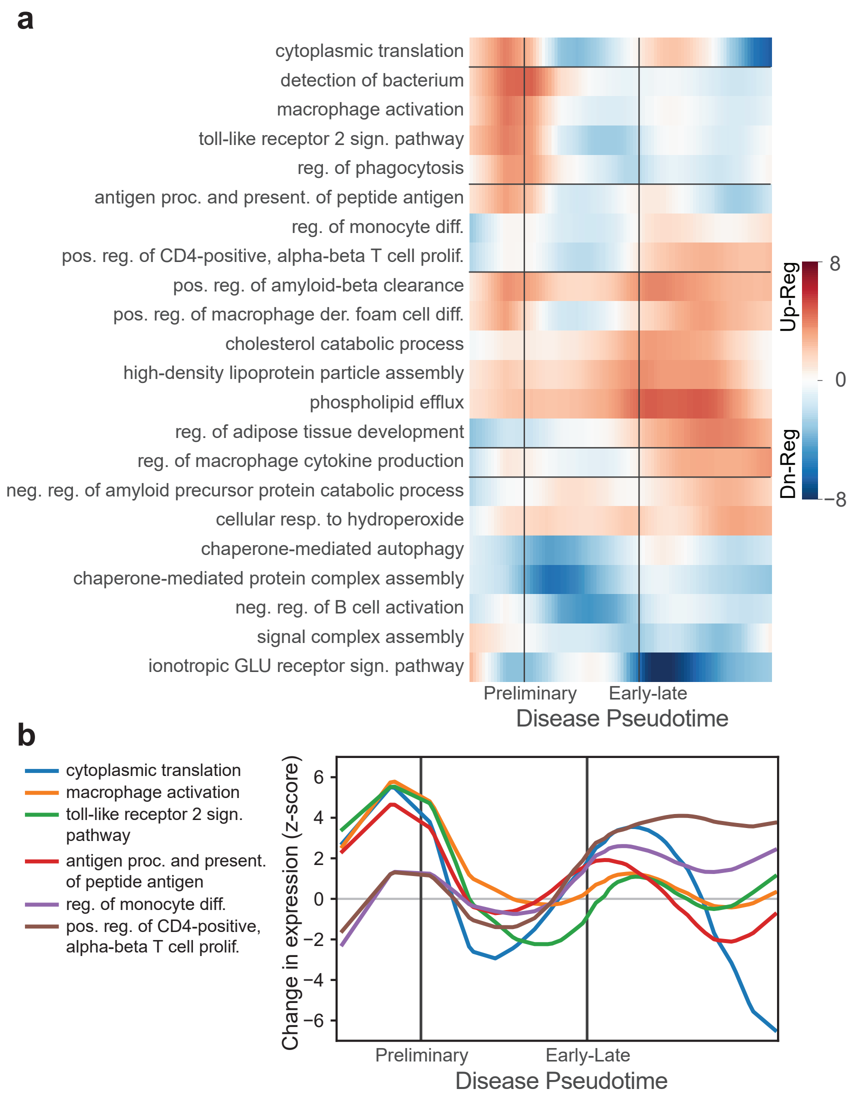
a
cytoplasmic translation
detection of bacterium
macrophage activation
toll-like receptor 2 sign. pathway
reg. of phagocytosis
antigen proc. and present. of peptide antigen
reg. of monocyte diff.
pos. reg. of CD4-positive, alpha-beta T cell prolif.
pos. reg. of amyloid-beta clearance
pos. reg. of macrophage der. foam cell diff.
cholesterol catabolic process
high-density lipoprotein particle assembly
phospholipid efflux
reg. of adipose tissue development
reg. of macrophage cytokine production
neg. reg. of amyloid precursor protein catabolic process
cellular resp. to hydroperoxide
chaperone-mediated autophagy
chaperone-mediated protein complex assembly
neg. reg. of B cell activation
signal complex assembly
ionotropic GLU receptor sign. pathway
Preliminary
Early-late
Disease Pseudotime
Up-Reg
Dn-Reg
8
0
−8
b
cytoplasmic translation
macrophage activation
toll-like receptor 2 sign. pathway
antigen proc. and present. of peptide antigen
reg. of monocyte diff.
pos. reg. of CD4-positive, alpha-beta T cell prolif.
Change in expression (z-score)
6
4
2
0
−2
−4
−6
Preliminary
Early-Late
Disease Pseudotime
