# Supplementary material for: Single-cell atlas of transcriptomic vulnerability across brain disorders
Source: Nature. 2026 Sep 23;657(8133):988–1002. doi: 10.1038/s41586-025-09573-z (PMC13600946; doi:10.1038/s41586-025-09573-z)
Supplement: Supplementary file 3 [file 41586_2025_9573_MOESM1_ESM.zip › 41586_2025_9573_MOESM1_ESM/2024-07-15352B-s1/PsychAD_Supplementary_F26_v1.pdf]

a

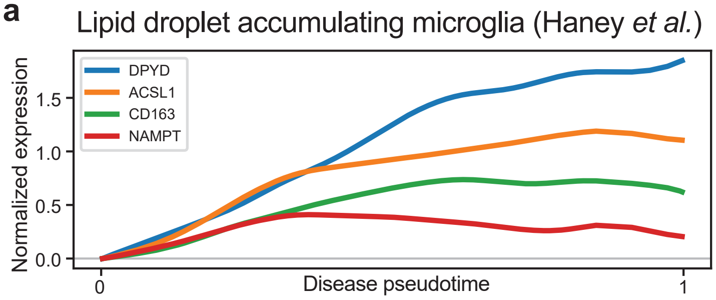

b

Gene	AD GWAS	Early Increase Pct.	Late Increase Pct.
DPYD	0.548	99.99%	99.90%
ACSL1	0.010	99.97%	99.44%
CD163	0.366	99.95%	22.07%
NAMPT	0.553	99.24%	0.52%

c

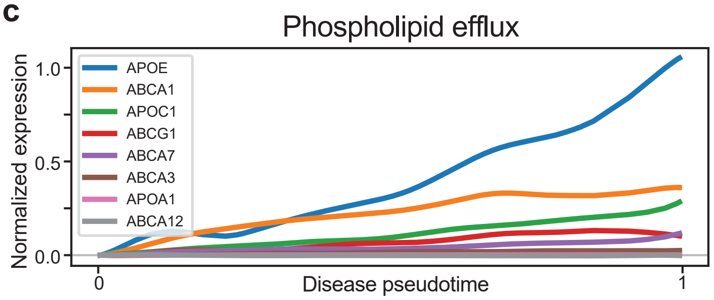

d

Gene	AD GWAS	Early Increase Pct.	Late Increase Pct.
APOE	5e-10	99.68%	99.94%
ABCA1	3.67e-06	98.96%	97.60%
APOC1	5e-10	96.92%	97.37%
ABCG1	0.096	95.98%	95.51%
ABCA7	1.01e-12	87.21%	92.68%
ABCA3	0.091	86.30%	74.48%
APOA1	0.360	56.17%	48.61%
ABCA12	0.141	32.41%	35.74%
