# Supplementary material for: Single-cell atlas of transcriptomic vulnerability across brain disorders
Source: Nature. 2026 Sep 23;657(8133):988–1002. doi: 10.1038/s41586-025-09573-z (PMC13600946; doi:10.1038/s41586-025-09573-z)
Supplement: Supplementary file 3 [file 41586_2025_9573_MOESM1_ESM.zip › 41586_2025_9573_MOESM1_ESM/2024-07-15352B-s1/PsychAD_Supplementary_F27_v1.pdf]

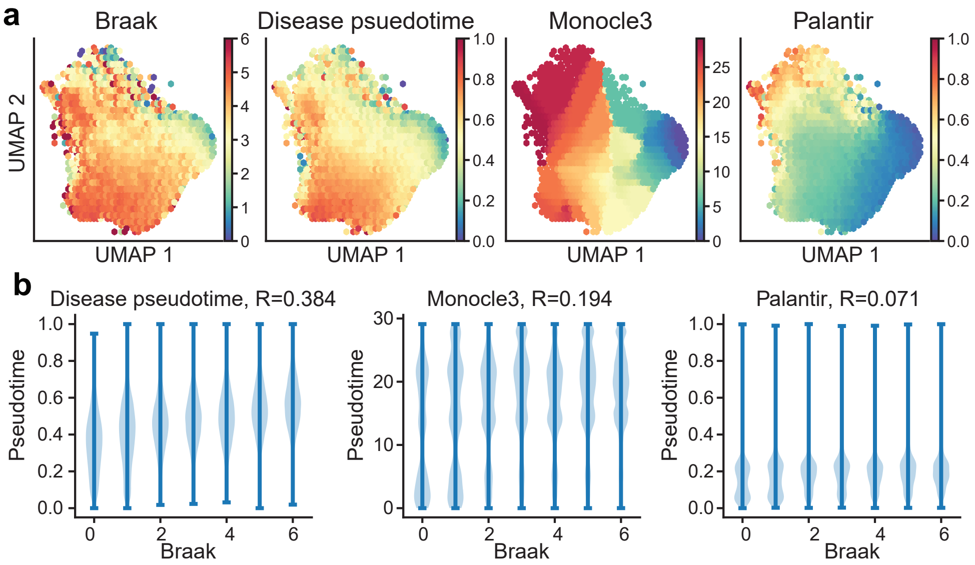

a
Braak
Disease psuedotime
Monocle3
Palantir
UMAP 2
UMAP 1
b
Disease pseudotime, R=0.384
Monocle3, R=0.194
Palantir, R=0.071
Pseudotime
Braak
