# Supplementary material for: Single-cell atlas of transcriptomic vulnerability across brain disorders
Source: Nature. 2026 Sep 23;657(8133):988–1002. doi: 10.1038/s41586-025-09573-z (PMC13600946; doi:10.1038/s41586-025-09573-z)
Supplement: Supplementary file 3 [file 41586_2025_9573_MOESM1_ESM.zip › 41586_2025_9573_MOESM1_ESM/2024-07-15352B-s1/PsychAD_Supplementary_F28_v1.pdf]

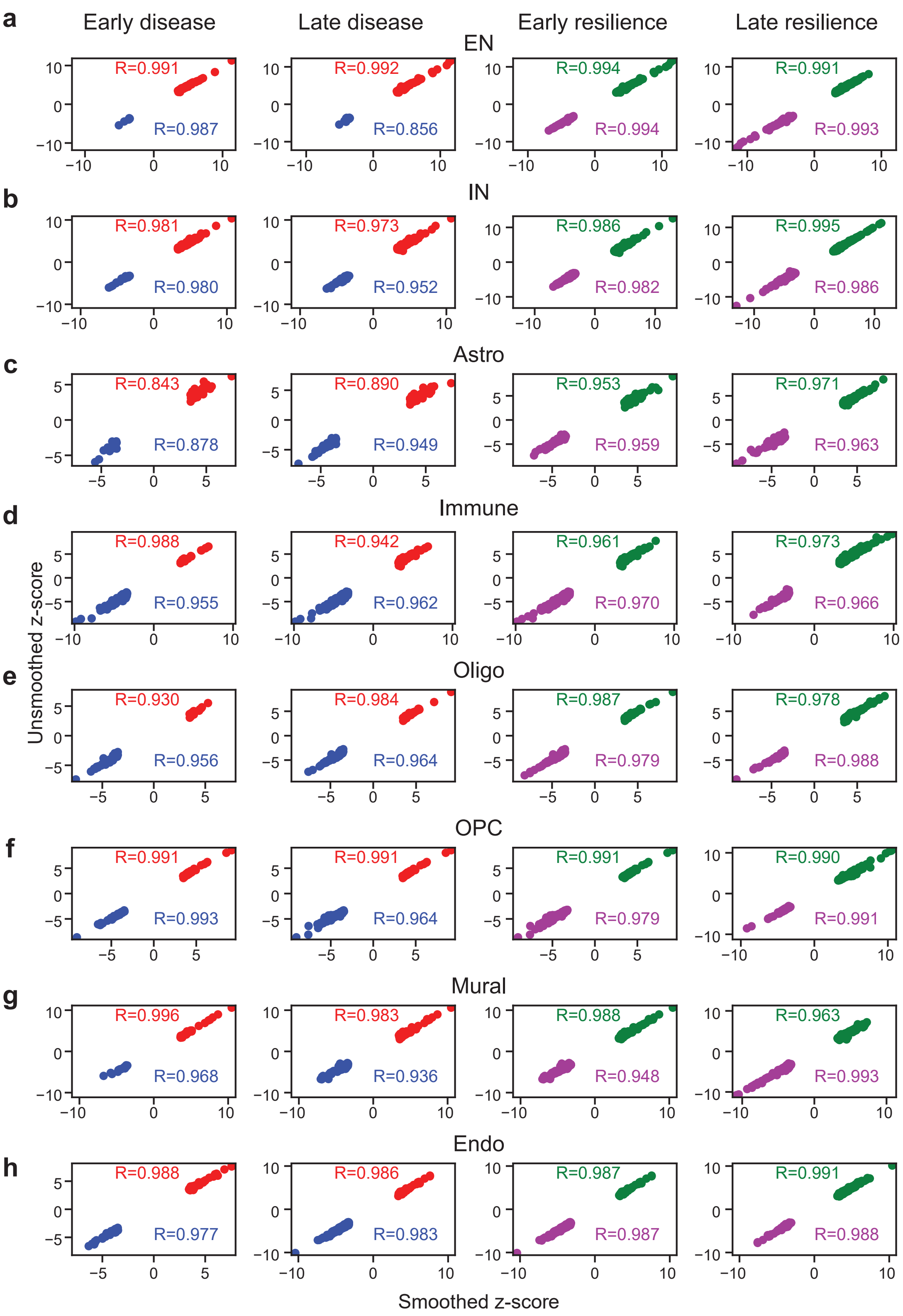

a
Early disease
Late disease
Early resilience
Late resilience
EN
R=0.991
R=0.987
R=0.992
R=0.856
R=0.994
R=0.994
R=0.991
R=0.993
b
IN
R=0.981
R=0.980
R=0.973
R=0.952
R=0.986
R=0.982
R=0.995
R=0.986
c
Astro
R=0.843
R=0.878
R=0.890
R=0.949
R=0.953
R=0.959
R=0.971
R=0.963
d
Immune
R=0.988
R=0.955
R=0.942
R=0.962
R=0.961
R=0.970
R=0.973
R=0.966
e
Oligo
R=0.930
R=0.956
R=0.984
R=0.964
R=0.987
R=0.979
R=0.978
R=0.988
f
OPC
R=0.991
R=0.993
R=0.991
R=0.964
R=0.991
R=0.979
R=0.990
R=0.991
g
Mural
R=0.996
R=0.968
R=0.983
R=0.936
R=0.988
R=0.948
R=0.963
R=0.993
h
Endo
R=0.988
R=0.977
R=0.986
R=0.983
R=0.987
R=0.987
R=0.991
R=0.988
Unsmoothed z-score
Smoothed z-score
