# Supplementary material for: Single-cell atlas of transcriptomic vulnerability across brain disorders
Source: Nature. 2026 Sep 23;657(8133):988–1002. doi: 10.1038/s41586-025-09573-z (PMC13600946; doi:10.1038/s41586-025-09573-z)
Supplement: Supplementary file 3 [file 41586_2025_9573_MOESM1_ESM.zip › 41586_2025_9573_MOESM1_ESM/2024-07-15352B-s1/PsychAD_Supplementary_F2_v1.pdf]

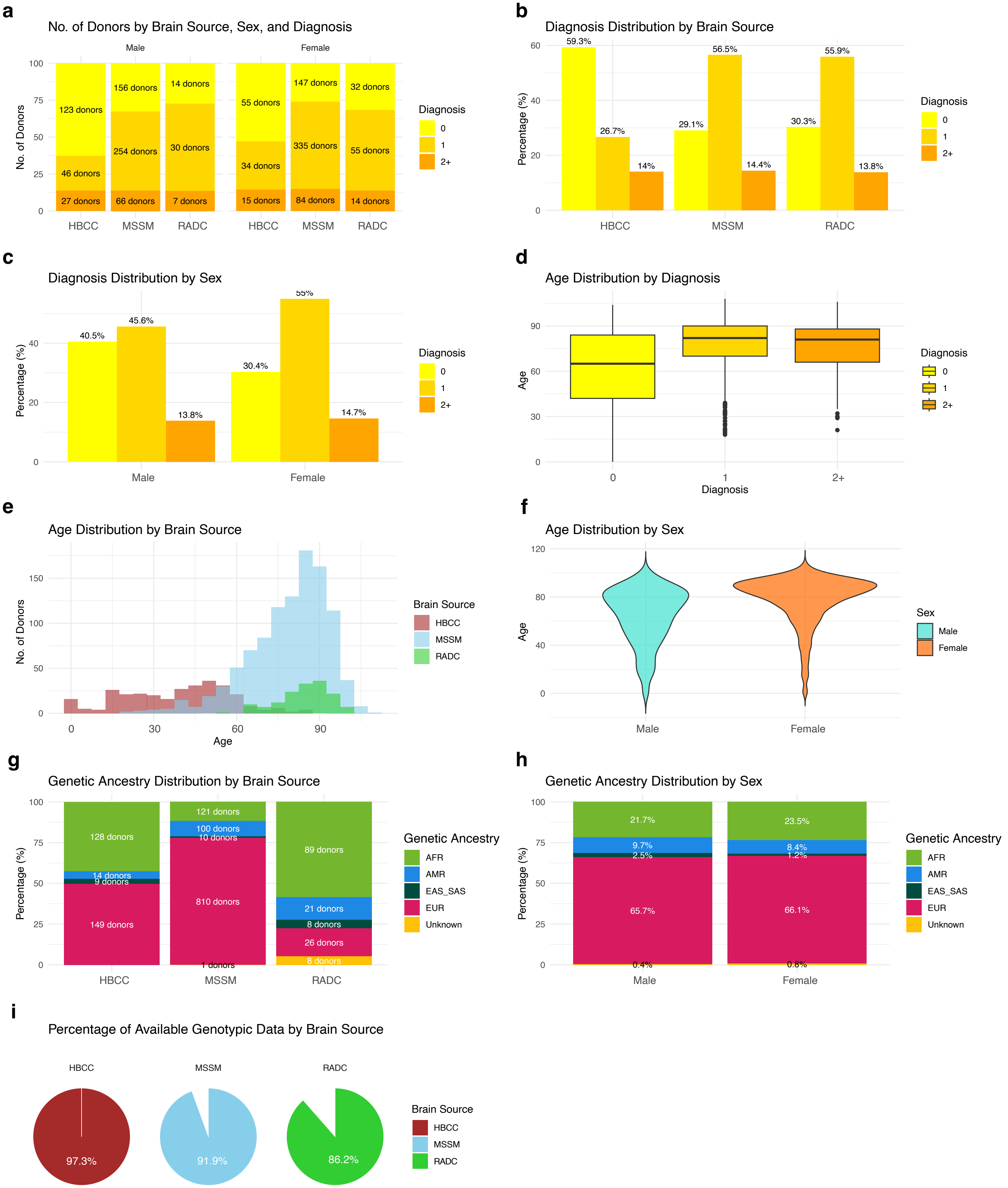

a
No. of Donors by Brain Source, Sex, and Diagnosis
Male
Female
No. of Donors
123 donors
46 donors
27 donors
156 donors
254 donors
66 donors
14 donors
30 donors
7 donors
55 donors
34 donors
15 donors
147 donors
335 donors
84 donors
32 donors
55 donors
14 donors
HBCC
MSSM
RADC
Diagnosis
0
1
2+
b
Diagnosis Distribution by Brain Source
Percentage (%)
59.3%
26.7%
14%
29.1%
56.5%
14.4%
30.3%
55.9%
13.8%
c
Diagnosis Distribution by Sex
40.5%
45.6%
13.8%
30.4%
55%
14.7%
Male
Female
d
Age Distribution by Diagnosis
Age
Diagnosis
e
Age Distribution by Brain Source
No. of Donors
Age
Brain Source
HBCC
MSSM
RADC
f
Age Distribution by Sex
Sex
Male
Female
g
Genetic Ancestry Distribution by Brain Source
128 donors
14 donors
9 donors
149 donors
121 donors
100 donors
10 donors
810 donors
1 donors
89 donors
21 donors
8 donors
26 donors
8 donors
Genetic Ancestry
AFR
AMR
EAS_SAS
EUR
Unknown
h
Genetic Ancestry Distribution by Sex
21.7%
9.7%
2.5%
65.7%
0.4%
23.5%
8.4%
1.2%
66.1%
0.8%
i
Percentage of Available Genotypic Data by Brain Source
97.3%
91.9%
86.2%
