# Supplementary material for: Single-cell atlas of transcriptomic vulnerability across brain disorders
Source: Nature. 2026 Sep 23;657(8133):988–1002. doi: 10.1038/s41586-025-09573-z (PMC13600946; doi:10.1038/s41586-025-09573-z)
Supplement: Supplementary file 3 [file 41586_2025_9573_MOESM1_ESM.zip › 41586_2025_9573_MOESM1_ESM/2024-07-15352B-s1/PsychAD_Supplementary_F30_v1.pdf]

BD FACSDiva 8.0.2

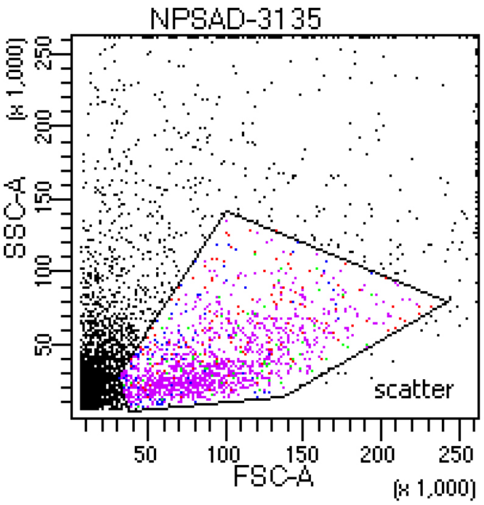

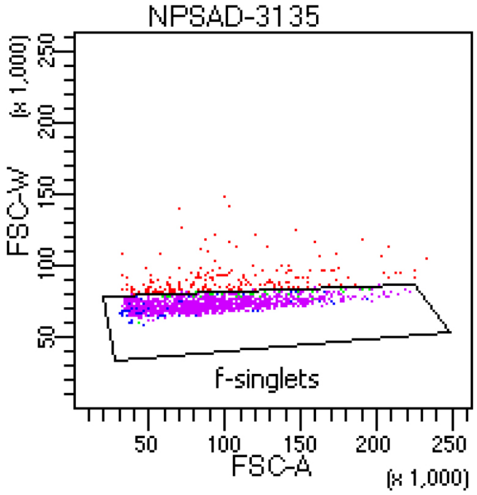

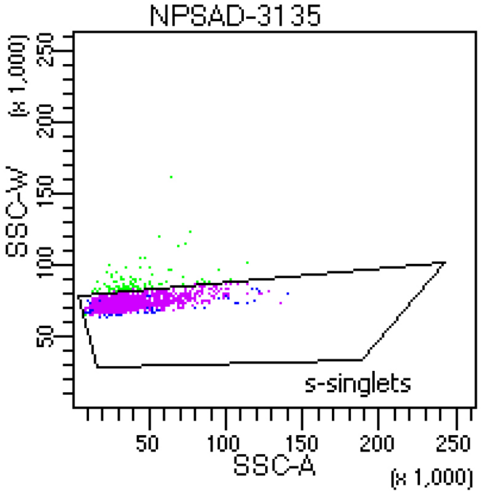

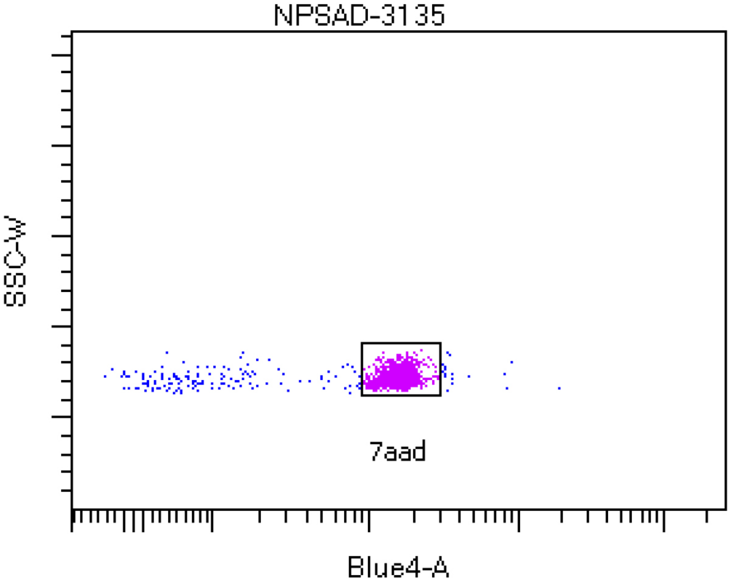

Tube: 3135

Population	#Events	%Parent	%Total
All Events	5,000	####	100.0
scatter	1,553	31.1	31.1
f-singlets	1,356	87.3	27.1
s-singlets	1,259	92.8	25.2
7aad	1,102	87.5	22.0
