# Supplementary material for: Single-cell atlas of transcriptomic vulnerability across brain disorders
Source: Nature. 2026 Sep 23;657(8133):988–1002. doi: 10.1038/s41586-025-09573-z (PMC13600946; doi:10.1038/s41586-025-09573-z)
Supplement: Supplementary file 3 [file 41586_2025_9573_MOESM1_ESM.zip › 41586_2025_9573_MOESM1_ESM/2024-07-15352B-s1/PsychAD_Supplementary_F3_v1.pdf]

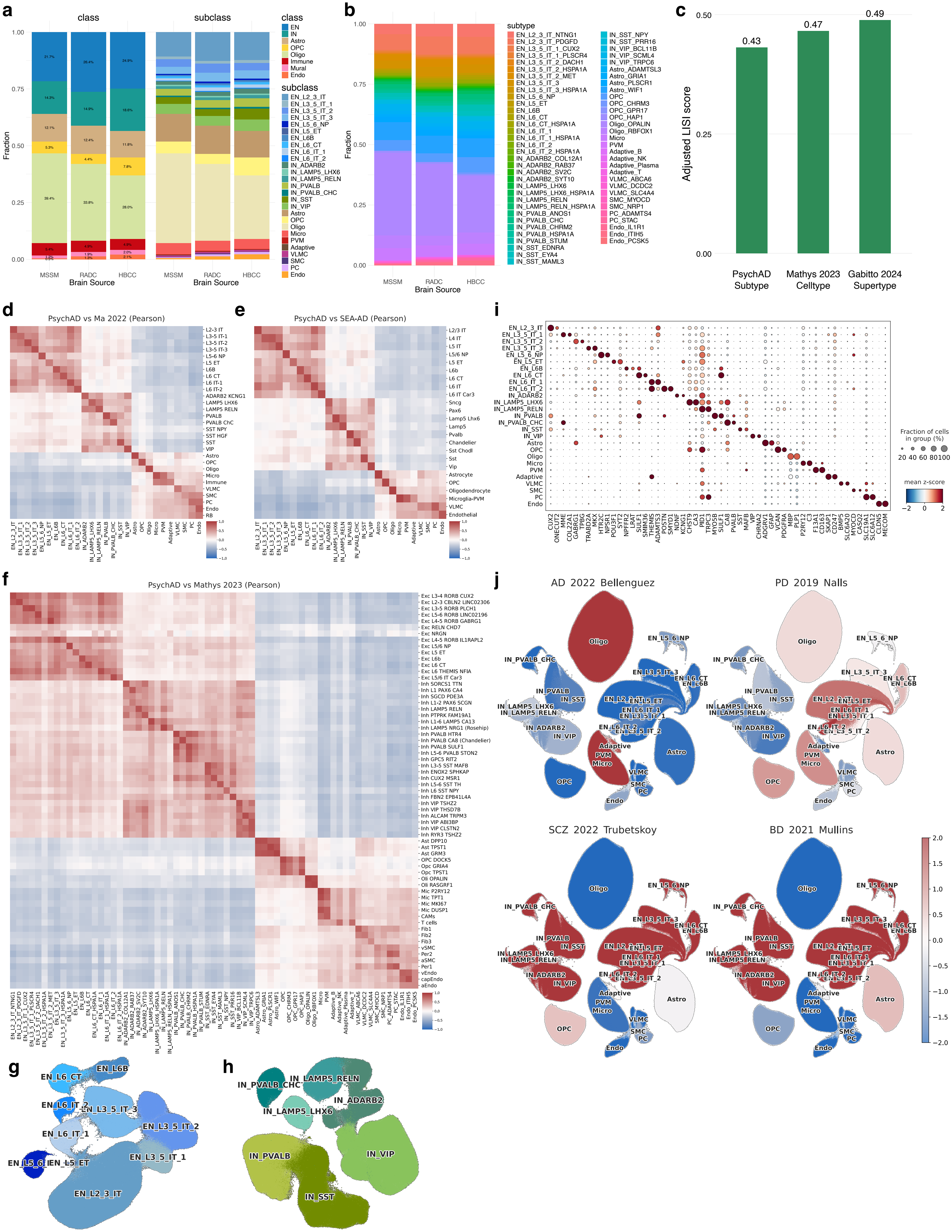
a
class
subclass
Fraction
Brain Source
MSSM
RADC
HBCC
class
EN
IN
Astro
OPC
Oligo
Immune
Mural
Endo
subclass
EN_L2_3_IT
EN_L3_5_IT_1
EN_L3_5_IT_2
EN_L3_5_IT_3
EN_L5_6_NP
EN_L5_ET
EN_L6B
EN_L6_CT
EN_L6_IT_1
EN_L6_IT_2
IN_ADARB2
IN_LAMP5_LHX6
IN_LAMP5_RELN
IN_PVALB
IN_PVALB_CHC
IN_SST
IN_VIP
Astro
OPC
Oligo
Micro
PVM
Adaptive
VLMC
SMC
PC
Endo
b
subtype
EN_L2_3_IT_NTNG1
EN_L2_3_IT_PDGFD
EN_L3_5_IT_1_CUX2
EN_L3_5_IT_1_PLSCR4
EN_L3_5_IT_2_DACH1
EN_L3_5_IT_2_HSPA1A
EN_L3_5_IT_2_MET
EN_L3_5_IT_3
EN_L3_5_IT_3_HSPA1A
EN_L5_6_NP
EN_L5_ET
EN_L6B
EN_L6_CT
EN_L6_CT_HSPA1A
EN_L6_IT_1
EN_L6_IT_1_HSPA1A
EN_L6_IT_2
EN_L6_IT_2_HSPA1A
IN_ADARB2_COL12A1
IN_ADARB2_RAB37
IN_ADARB2_SV2C
IN_ADARB2_SYT10
IN_LAMP5_LHX6
IN_LAMP5_LHX6_HSPA1A
IN_LAMP5_RELN
IN_LAMP5_RELN_HSPA1A
IN_PVALB_ANOS1
IN_PVALB_CHC
IN_PVALB_CHRM2
IN_PVALB_HSPA1A
IN_PVALB_STUM
IN_SST_EDNRA
IN_SST_EYA4
IN_SST_MAML3
IN_SST_NPY
IN_SST_PRR16
IN_VIP_BCL11B
IN_VIP_SCML4
IN_VIP_TRPC6
Astro_ADAMTSL3
Astro_GRIA1
Astro_PLSCR1
Astro_WIF1
OPC
OPC_CHRM3
OPC_GPR17
OPC_HAP1
Oligo_OPALIN
Oligo_RBFOX1
Micro
PVM
Adaptive_B
Adaptive_NK
Adaptive_Plasma
Adaptive_T
VLMC_ABCA6
VLMC_DCDC2
VLMC_SLC4A4
SMC_MYOCD
SMC_NRP1
PC_ADAMTS4
PC_STAC
Endo_IL1R1
Endo_ITIH5
Endo_PCSK5
c
Adjusted LISI score
0.43
0.47
0.49
PsychAD Subtype
Mathys 2023 Celltype
Gabitto 2024 Supertype
d
PsychAD vs Ma 2022 (Pearson)
e
PsychAD vs SEA-AD (Pearson)
f
PsychAD vs Mathys 2023 (Pearson)
g
h
i
Fraction of cells in group (%)
20 40 60 80 100
mean z-score
j
AD 2022 Bellenguez
PD 2019 Nalls
SCZ 2022 Trubetskoy
BD 2021 Mullins
