# Supplementary material for: Single-cell atlas of transcriptomic vulnerability across brain disorders
Source: Nature. 2026 Sep 23;657(8133):988–1002. doi: 10.1038/s41586-025-09573-z (PMC13600946; doi:10.1038/s41586-025-09573-z)
Supplement: Supplementary file 3 [file 41586_2025_9573_MOESM1_ESM.zip › 41586_2025_9573_MOESM1_ESM/2024-07-15352B-s1/PsychAD_Supplementary_F4_v4.pdf]

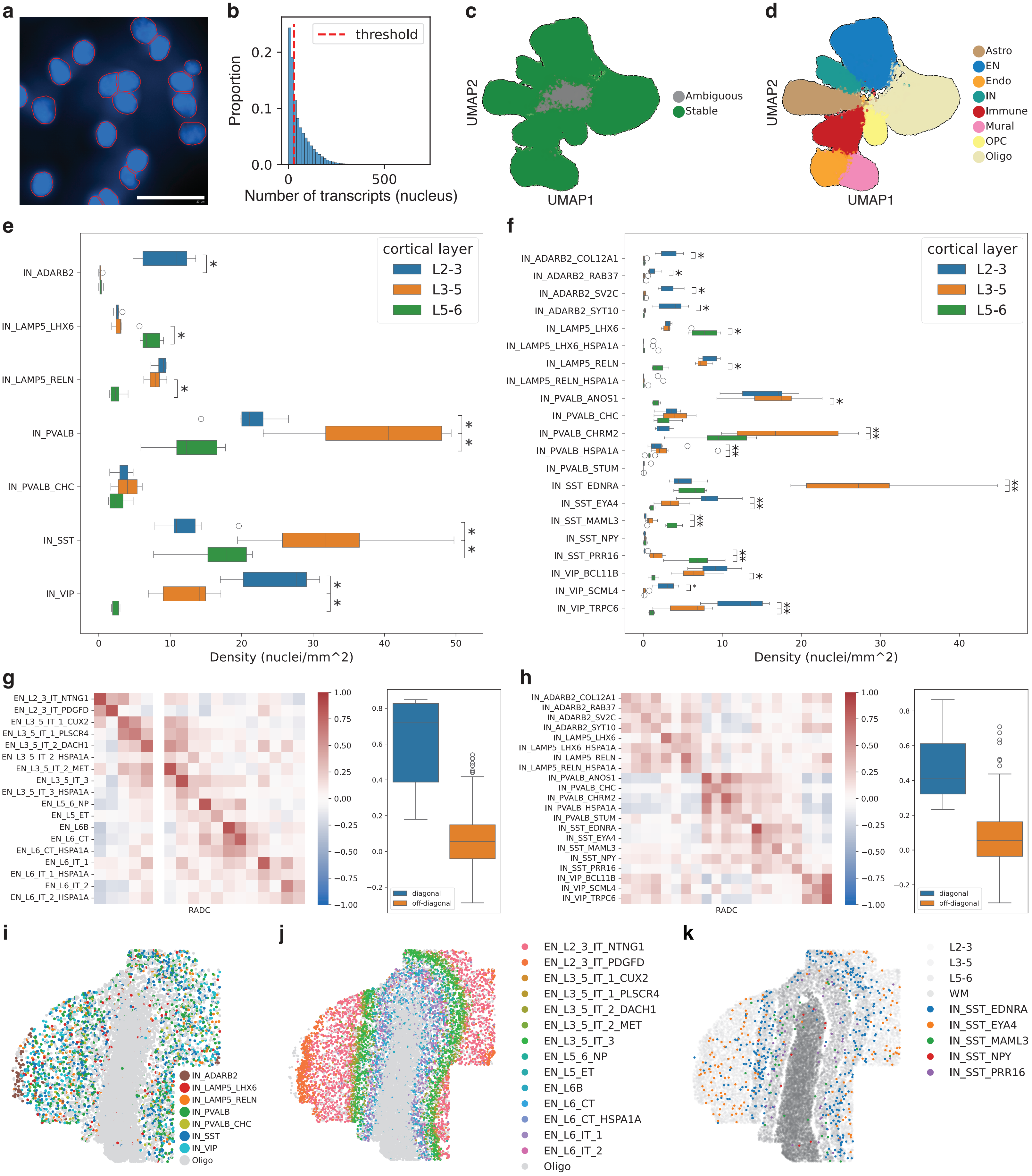
a
b
threshold
Proportion
Number of transcripts (nucleus)
c
Ambiguous
Stable
UMAP1
UMAP2
d
Astro
EN
Endo
IN
Immune
Mural
OPC
Oligo
e
cortical layer
L2-3
L3-5
L5-6
IN_ADARB2
IN_LAMP5_LHX6
IN_LAMP5_RELN
IN_PVALB
IN_PVALB_CHC
IN_SST
IN_VIP
Density (nuclei/mm^2)
f
IN_ADARB2_COL12A1
IN_ADARB2_RAB37
IN_ADARB2_SV2C
IN_ADARB2_SYT10
IN_LAMP5_LHX6
IN_LAMP5_LHX6_HSPA1A
IN_LAMP5_RELN
IN_LAMP5_RELN_HSPA1A
IN_PVALB_ANOS1
IN_PVALB_CHC
IN_PVALB_CHRM2
IN_PVALB_HSPA1A
IN_PVALB_STUM
IN_SST_EDNRA
IN_SST_EYA4
IN_SST_MAML3
IN_SST_NPY
IN_SST_PRR16
IN_VIP_BCL11B
IN_VIP_SCML4
IN_VIP_TRPC6
g
EN_L2_3_IT_NTNG1
EN_L2_3_IT_PDGFD
EN_L3_5_IT_1_CUX2
EN_L3_5_IT_1_PLSCR4
EN_L3_5_IT_2_DACH1
EN_L3_5_IT_2_HSPA1A
EN_L3_5_IT_2_MET
EN_L3_5_IT_3
EN_L3_5_IT_3_HSPA1A
EN_L5_6_NP
EN_L5_ET
EN_L6B
EN_L6_CT
EN_L6_CT_HSPA1A
EN_L6_IT_1
EN_L6_IT_1_HSPA1A
EN_L6_IT_2
EN_L6_IT_2_HSPA1A
RADC
diagonal
off-diagonal
h
i
IN_ADARB2
IN_LAMP5_LHX6
IN_LAMP5_RELN
IN_PVALB
IN_PVALB_CHC
IN_SST
IN_VIP
Oligo
j
EN_L2_3_IT_NTNG1
EN_L2_3_IT_PDGFD
EN_L3_5_IT_1_CUX2
EN_L3_5_IT_1_PLSCR4
EN_L3_5_IT_2_DACH1
EN_L3_5_IT_2_MET
EN_L3_5_IT_3
EN_L5_6_NP
EN_L5_ET
EN_L6B
EN_L6_CT
EN_L6_CT_HSPA1A
EN_L6_IT_1
EN_L6_IT_2
Oligo
k
L2-3
L3-5
L5-6
WM
IN_SST_EDNRA
IN_SST_EYA4
IN_SST_MAML3
IN_SST_NPY
IN_SST_PRR16
