# Supplementary material for: Single-cell atlas of transcriptomic vulnerability across brain disorders
Source: Nature. 2026 Sep 23;657(8133):988–1002. doi: 10.1038/s41586-025-09573-z (PMC13600946; doi:10.1038/s41586-025-09573-z)
Supplement: Supplementary file 3 [file 41586_2025_9573_MOESM1_ESM.zip › 41586_2025_9573_MOESM1_ESM/2024-07-15352B-s1/PsychAD_Supplementary_F5_v2.pdf]

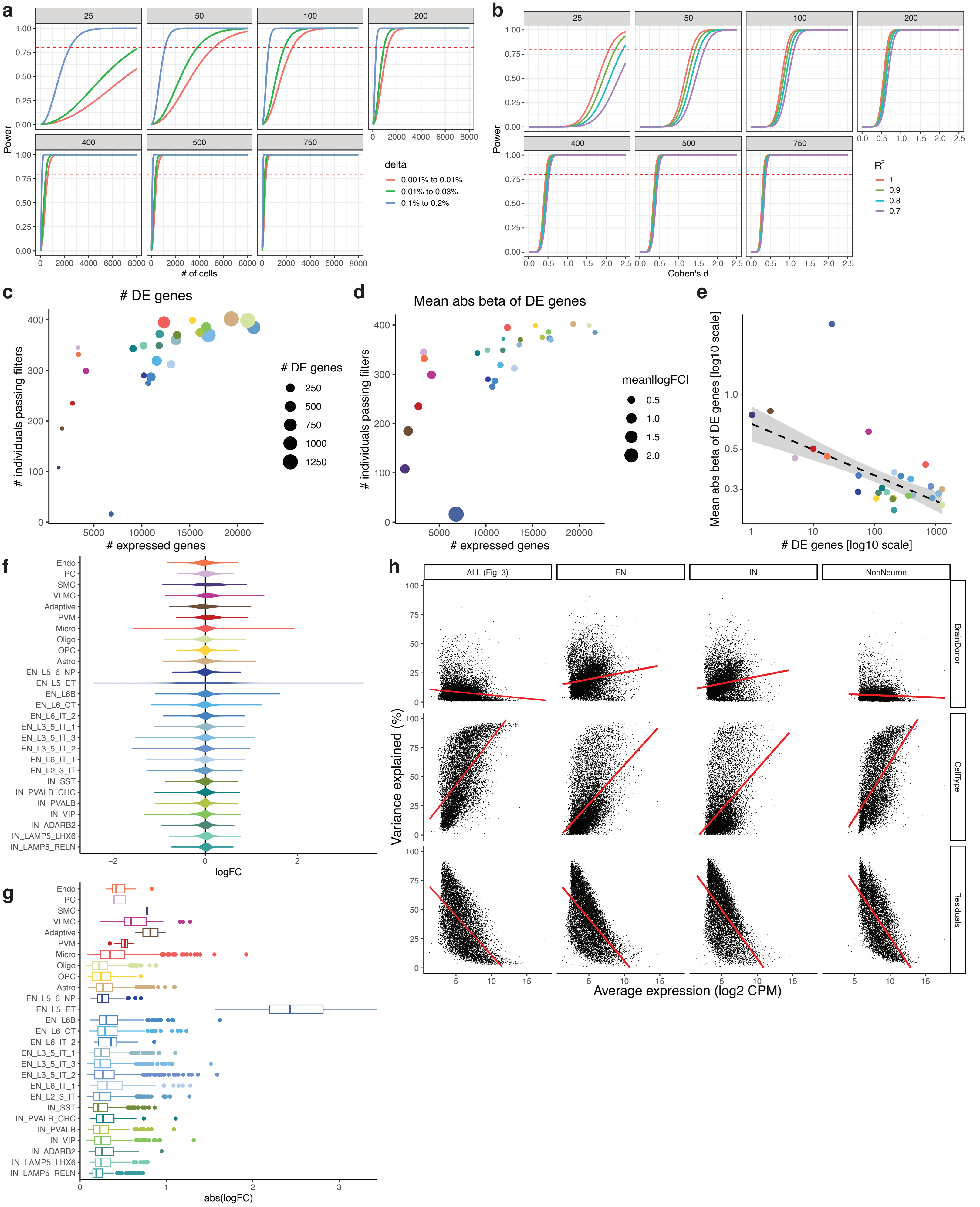
a
Power
# of cells
delta
0.001% to 0.01%
0.01% to 0.03%
0.1% to 0.2%
b
Power
Cohen's d
R^2
c
DE genes
individuals passing filters
expressed genes
d
Mean abs beta of DE genes
meanIlogFCI
e
Mean abs beta of DE genes [log10 scale]
DE genes [log10 scale]
f
logFC
g
abs(logFC)
h
ALL (Fig. 3)
EN
IN
NonNeuron
BrainDonor
CellType
Residuals
Variance explained (%)
Average expression (log2 CPM)
