# Supplementary material for: Single-cell atlas of transcriptomic vulnerability across brain disorders
Source: Nature. 2026 Sep 23;657(8133):988–1002. doi: 10.1038/s41586-025-09573-z (PMC13600946; doi:10.1038/s41586-025-09573-z)
Supplement: Supplementary file 3 [file 41586_2025_9573_MOESM1_ESM.zip › 41586_2025_9573_MOESM1_ESM/2024-07-15352B-s1/PsychAD_Supplementary_F6_v2.pdf]

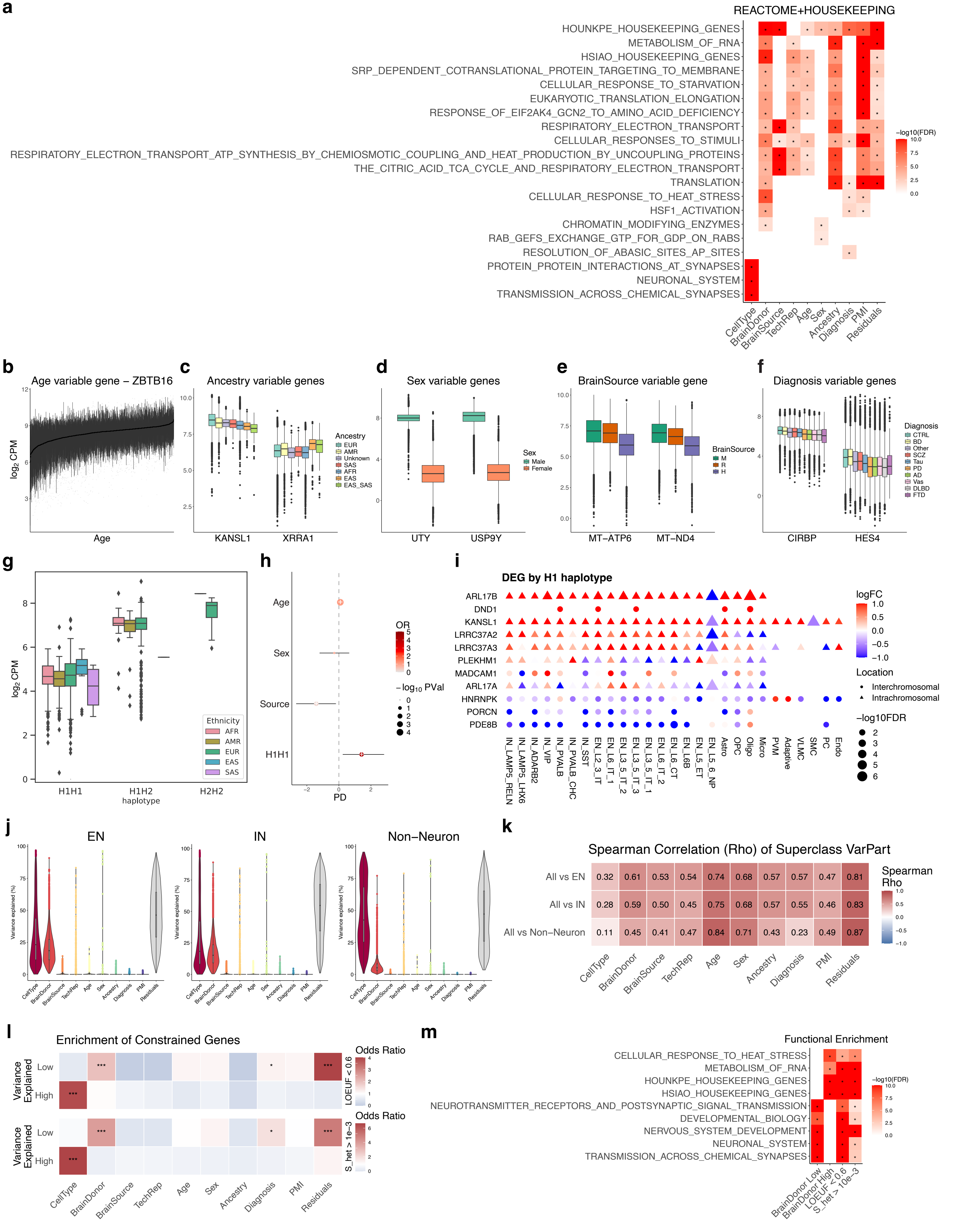

a
REACTOME+HOUSEKEEPING
HOUNKPE_HOUSEKEEPING_GENES
METABOLISM_OF_RNA
HSIAO_HOUSEKEEPING_GENES
SRP_DEPENDENT_COTRANSLATIONAL_PROTEIN_TARGETING_TO_MEMBRANE
CELLULAR_RESPONSE_TO_STARVATION
EUKARYOTIC_TRANSLATION_ELONGATION
RESPONSE_OF_EIF2AK4_GCN2_TO_AMINO_ACID_DEFICIENCY
RESPIRATORY_ELECTRON_TRANSPORT
CELLULAR_RESPONSES_TO_STIMULI
RESPIRATORY_ELECTRON_TRANSPORT_ATP_SYNTHESIS_BY_CHEMIOSMOTIC_COUPLING_AND_HEAT_PRODUCTION_BY_UNCOUPLING_PROTEINS
THE_CITRIC_ACID_TCA_CYCLE_AND_RESPIRATORY_ELECTRON_TRANSPORT
TRANSLATION
CELLULAR_RESPONSE_TO_HEAT_STRESS
HSF1_ACTIVATION
CHROMATIN_MODIFYING_ENZYMES
RAB_GEFS_EXCHANGE_GTP_FOR_GDP_ON_RABS
RESOLUTION_OF_ABASIC_SITES_AP_SITES
PROTEIN_PROTEIN_INTERACTIONS_AT_SYNAPSES
NEURONAL_SYSTEM
TRANSMISSION_ACROSS_CHEMICAL_SYNAPSES
CellType
BrainDonor
BrainSource
TechRep
Age
Sex
Ancestry
Diagnosis
PMI
Residuals
−log10(FDR)
10.0
7.5
5.0
2.5
0.0
b
Age variable gene − ZBTB16
log2 CPM
Age
c
Ancestry variable genes
KANSL1
XRRA1
Ancestry
EUR
AMR
Unknown
SAS
AFR
EAS
EAS_SAS
d
Sex variable genes
UTY
USP9Y
Sex
Male
Female
e
BrainSource variable gene
MT−ATP6
MT−ND4
BrainSource
M
R
H
f
Diagnosis variable genes
CIRBP
HES4
Diagnosis
CTRL
BD
Other
SCZ
Tau
PD
AD
Vas
DLBD
FTD
g
log2 CPM
Ethnicity
AFR
AMR
EUR
EAS
SAS
H1H1
H1H2
H2H2
haplotype
h
Age
Sex
Source
H1H1
PD
OR
−log10 PVal
i
DEG by H1 haplotype
ARL17B
DND1
KANSL1
LRRC37A2
LRRC37A3
PLEKHM1
MADCAM1
ARL17A
HNRNPK
PORCN
PDE8B
IN_LAMP5_RELN
IN_LAMP5_LHX6
IN_ADARB2
IN_VIP
IN_PVALB
IN_PVALB_CHC
IN_SST
EN_L2_3_IT
EN_L6_IT_1
EN_L3_5_IT_2
EN_L3_5_IT_3
EN_L3_5_IT_1
EN_L6_IT_2
EN_L6_CT
EN_L6B
EN_L5_ET
EN_L5_6_NP
Astro
OPC
Oligo
Micro
PVM
Adaptive
VLMC
SMC
PC
Endo
logFC
Location
Interchromosomal
Intrachromosomal
−log10FDR
j
EN
IN
Non−Neuron
Variance explained (%)
k
Spearman Correlation (Rho) of Superclass VarPart
All vs EN 0.32 0.61 0.53 0.54 0.74 0.68 0.57 0.57 0.47 0.81
All vs IN 0.28 0.59 0.50 0.45 0.75 0.68 0.57 0.55 0.46 0.83
All vs Non−Neuron 0.11 0.45 0.41 0.47 0.84 0.71 0.43 0.23 0.49 0.87
Spearman Rho
l
Enrichment of Constrained Genes
Variance Explained
Low
High
Odds Ratio
LOEUF < 0.6
S_het > 1e−3
m
Functional Enrichment
CELLULAR_RESPONSE_TO_HEAT_STRESS
METABOLISM_OF_RNA
HOUNKPE_HOUSEKEEPING_GENES
HSIAO_HOUSEKEEPING_GENES
NEUROTRANSMITTER_RECEPTORS_AND_POSTSYNAPTIC_SIGNAL_TRANSMISSION
DEVELOPMENTAL_BIOLOGY
NERVOUS_SYSTEM_DEVELOPMENT
NEURONAL_SYSTEM
TRANSMISSION_ACROSS_CHEMICAL_SYNAPSES
BrainDonor Low
BrainDonor High
LOEUF < 0.6
S_het > 10e−3
−log10(FDR)
