# Supplementary material for: Single-cell atlas of transcriptomic vulnerability across brain disorders
Source: Nature. 2026 Sep 23;657(8133):988–1002. doi: 10.1038/s41586-025-09573-z (PMC13600946; doi:10.1038/s41586-025-09573-z)
Supplement: Supplementary file 3 [file 41586_2025_9573_MOESM1_ESM.zip › 41586_2025_9573_MOESM1_ESM/2024-07-15352B-s1/PsychAD_Supplementary_F7_v2.pdf]

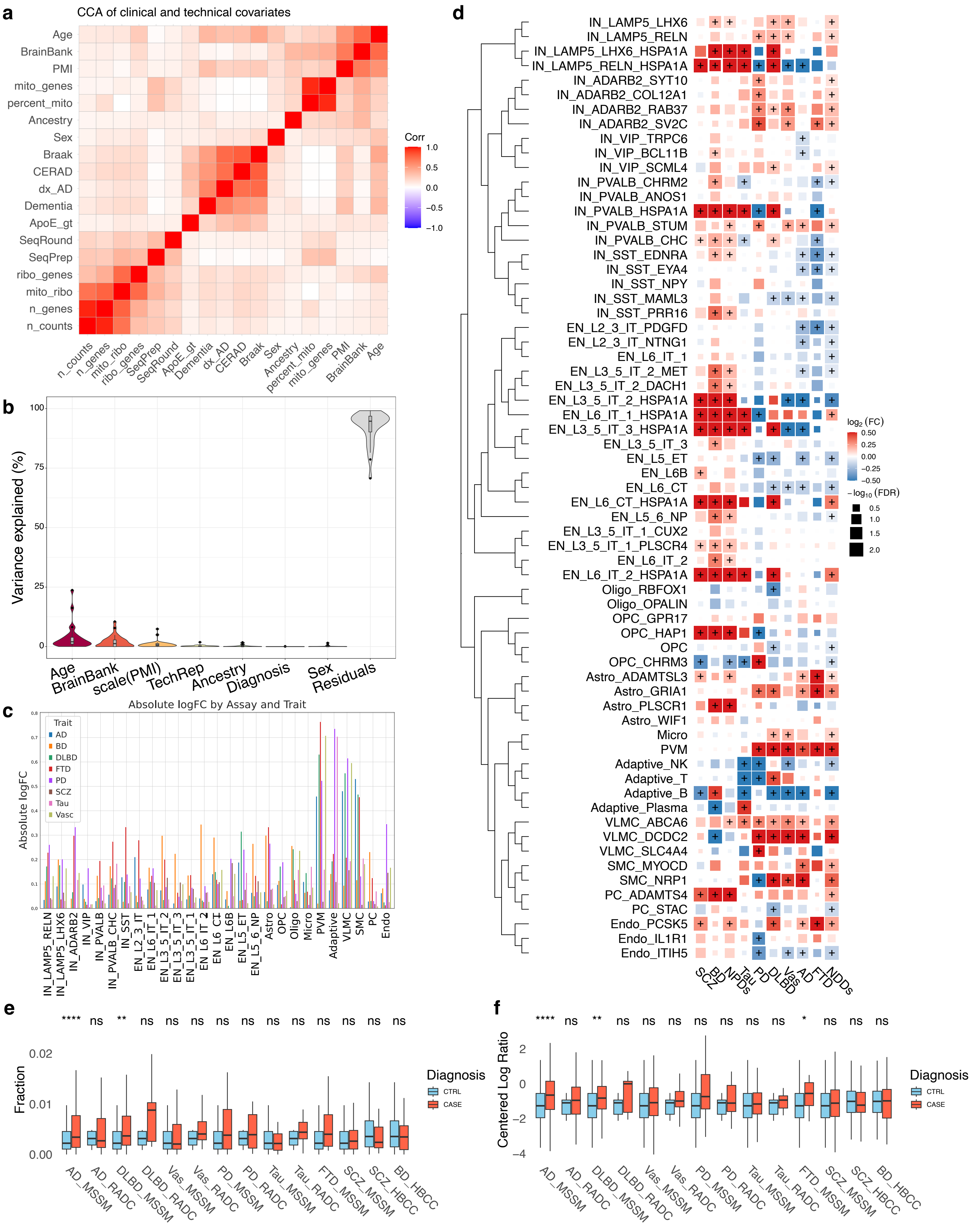
a
CCA of clinical and technical covariates
Age
BrainBank
PMI
mito_genes
percent_mito
Ancestry
Sex
Braak
CERAD
dx_AD
Dementia
ApoE_gt
SeqRound
SeqPrep
ribo_genes
mito_ribo
n_genes
n_counts
Corr
1.0
0.5
0.0
−0.5
−1.0
b
Variance explained (%)
100
75
50
25
0
Age
BrainBank
scale(PMI)
TechRep
Ancestry
Diagnosis
Sex
Residuals
c
Absolute logFC by Assay and Trait
Absolute logFC
Trait
AD
BD
DLBD
FTD
PD
SCZ
Tau
Vasc
IN_LAMP5_RELN
IN_LAMP5_LHX6
IN_ADARB2
IN_VIP
IN_PVALB
IN_PVALB_CHC
IN_SST
EN_L2_3_IT
EN_L6_IT_1
EN_L3_5_IT_2
EN_L3_5_IT_3
EN_L3_5_IT_1
EN_L6_IT_2
EN_L6_CT
EN_L6B
EN_L5_ET
EN_L5_6_NP
Astro
OPC
Oligo
Micro
PVM
Adaptive
VLMC
SMC
PC
Endo
d
IN_LAMP5_LHX6
IN_LAMP5_RELN
IN_LAMP5_LHX6_HSPA1A
IN_LAMP5_RELN_HSPA1A
IN_ADARB2_SYT10
IN_ADARB2_COL12A1
IN_ADARB2_RAB37
IN_ADARB2_SV2C
IN_VIP_TRPC6
IN_VIP_BCL11B
IN_VIP_SCML4
IN_PVALB_CHRM2
IN_PVALB_ANOS1
IN_PVALB_HSPA1A
IN_PVALB_STUM
IN_PVALB_CHC
IN_SST_EDNRA
IN_SST_EYA4
IN_SST_NPY
IN_SST_MAML3
IN_SST_PRR16
EN_L2_3_IT_PDGFD
EN_L2_3_IT_NTNG1
EN_L6_IT_1
EN_L3_5_IT_2_MET
EN_L3_5_IT_2_DACH1
EN_L3_5_IT_2_HSPA1A
EN_L6_IT_1_HSPA1A
EN_L3_5_IT_3_HSPA1A
EN_L3_5_IT_3
EN_L5_ET
EN_L6B
EN_L6_CT
EN_L6_CT_HSPA1A
EN_L5_6_NP
EN_L3_5_IT_1_CUX2
EN_L3_5_IT_1_PLSCR4
EN_L6_IT_2
EN_L6_IT_2_HSPA1A
Oligo_RBFOX1
Oligo_OPALIN
OPC_GPR17
OPC_HAP1
OPC
OPC_CHRM3
Astro_ADAMTSL3
Astro_GRIA1
Astro_PLSCR1
Astro_WIF1
Micro
PVM
Adaptive_NK
Adaptive_T
Adaptive_B
Adaptive_Plasma
VLMC_ABCA6
VLMC_DCDC2
VLMC_SLC4A4
SMC_MYOCD
SMC_NRP1
PC_ADAMTS4
PC_STAC
Endo_PCSK5
Endo_IL1R1
Endo_ITIH5
SCZ
BD
NPDs
Tau
PD
DLBD
Vas
AD
FTD
NDDs
log2 (FC)
0.50
0.25
0.00
−0.25
−0.50
−log10 (FDR)
0.5
1.0
1.5
2.0
e
**** ns ** ns ns ns ns ns ns ns ns ns ns ns
Fraction
0.02
0.01
0.00
Diagnosis
CTRL
CASE
AD_MSSM
AD_RADC
DLBD_MSSM
DLBD_RADC
Vas_MSSM
Vas_RADC
PD_MSSM
PD_RADC
Tau_MSSM
Tau_RADC
FTD_MSSM
SCZ_MSSM
SCZ_HBCC
BD_HBCC
f
**** ns ** ns ns ns ns ns ns ns * ns ns ns
Centered Log Ratio
2
0
−2
−4
Diagnosis
CTRL
CASE
AD_MSSM
AD_RADC
DLBD_MSSM
DLBD_RADC
Vas_MSSM
Vas_RADC
PD_MSSM
PD_RADC
Tau_MSSM
Tau_RADC
FTD_MSSM
SCZ_MSSM
SCZ_HBCC
BD_HBCC
