# Supplementary material for: Single-cell atlas of transcriptomic vulnerability across brain disorders
Source: Nature. 2026 Sep 23;657(8133):988–1002. doi: 10.1038/s41586-025-09573-z (PMC13600946; doi:10.1038/s41586-025-09573-z)
Supplement: Supplementary file 3 [file 41586_2025_9573_MOESM1_ESM.zip › 41586_2025_9573_MOESM1_ESM/2024-07-15352B-s1/PsychAD_Supplementary_F8_v1.pdf]

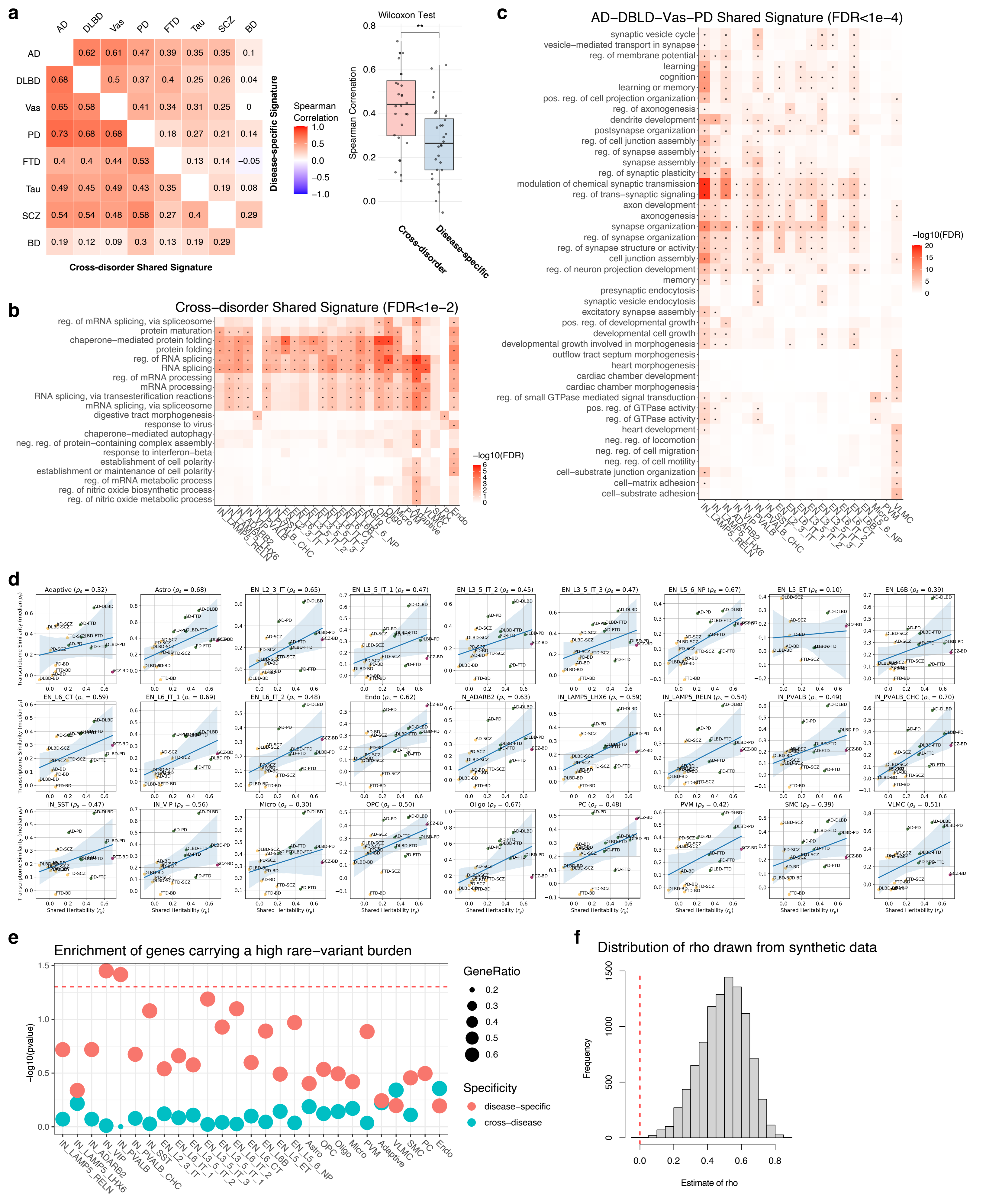
a
AD
DLBD
Vas
PD
FTD
Tau
SCZ
BD
Cross-disorder Shared Signature
Disease-specific Signature
Spearman Correlation
Wilcoxon Test
Spearman Correlation
Cross-disorder
Disease-specific
c
AD–DBLD–Vas–PD Shared Signature (FDR<1e–4)
−log10(FDR)
b
Cross–disorder Shared Signature (FDR<1e–2)
−log10(FDR)
d
Transcriptome Similarity (median ρs)
Shared Heritability (rg)
e
Enrichment of genes carrying a high rare–variant burden
−log10(pvalue)
GeneRatio
Specificity
disease–specific
cross–disease
f
Distribution of rho drawn from synthetic data
Frequency
Estimate of rho
