# Supplementary material for: Single-cell atlas of transcriptomic vulnerability across brain disorders
Source: Nature. 2026 Sep 23;657(8133):988–1002. doi: 10.1038/s41586-025-09573-z (PMC13600946; doi:10.1038/s41586-025-09573-z)
Supplement: Supplementary file 3 [file 41586_2025_9573_MOESM1_ESM.zip › 41586_2025_9573_MOESM1_ESM/2024-07-15352B-s1/PsychAD_Supplementary_F9_v3.pdf]

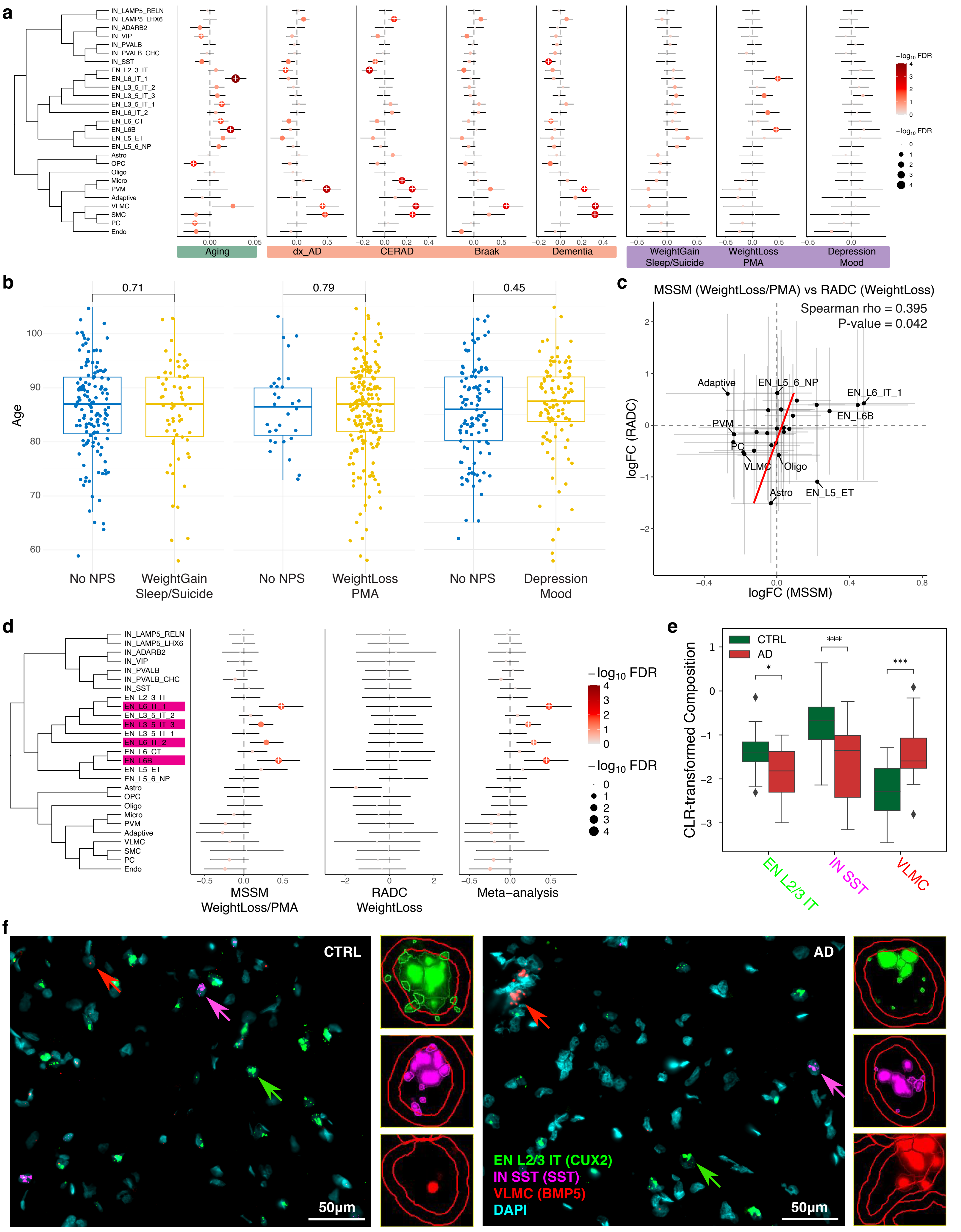
a
IN_LAMP5_RELN
IN_LAMP5_LHX6
IN_ADARB2
IN_VIP
IN_PVALB
IN_PVALB_CHC
IN_SST
EN_L2_3_IT
EN_L6_IT_1
EN_L3_5_IT_2
EN_L3_5_IT_3
EN_L3_5_IT_1
EN_L6_IT_2
EN_L6_CT
EN_L6B
EN_L5_ET
EN_L5_6_NP
Astro
OPC
Oligo
Micro
PVM
Adaptive
VLMC
SMC
PC
Endo
Aging
dx_AD
CERAD
Braak
Dementia
WeightGain
Sleep/Suicide
WeightLoss
PMA
Depression
Mood
−log10 FDR
b
0.71
0.79
0.45
Age
No NPS
WeightGain
Sleep/Suicide
WeightLoss
PMA
Depression
Mood
c
MSSM (WeightLoss/PMA) vs RADC (WeightLoss)
Spearman rho = 0.395
P-value = 0.042
logFC (RADC)
logFC (MSSM)
Adaptive
EN_L5_6_NP
EN_L6_IT_1
EN_L6B
PVM
PC
VLMC
Oligo
Astro
EN_L5_ET
d
MSSM
WeightLoss/PMA
RADC
WeightLoss
Meta−analysis
−log10 FDR
e
CTRL
AD
CLR-transformed Composition
EN L2/3 IT
IN SST
VLMC
f
CTRL
AD
50μm
EN L2/3 IT (CUX2)
IN SST (SST)
VLMC (BMP5)
DAPI
