# Supplementary material for: Single-cell atlas of transcriptomic vulnerability across brain disorders
Source: Nature. 2026 Sep 23;657(8133):988–1002. doi: 10.1038/s41586-025-09573-z (PMC13600946; doi:10.1038/s41586-025-09573-z)
Supplement: Supplementary file 3 [file 41586_2025_9573_MOESM1_ESM.zip › 41586_2025_9573_MOESM1_ESM/2024-07-15352B-s1/Supp-figure-legends.docx]

SUPPLEMENTARY INFORMATION

# Supplementary Figure Legends

#### **Supplementary Fig. 1.** **(a)** Extended schematic overview of snRNA-seq experiments leading to unified taxonomy. Shadow figure of Fig. 1a. **(b)** Venn diagram visualization of donor breakdown by NPS categories and sex. Shadow figure of Fig. 1e. **(c)** Distribution of gene counts by UMI counts after QC of 6,320,459 nuclei. **(d)** Cumulative nuclei count by percent mitochondrial genes. **(e)** Distribution of nuclei counts per sample. **(f)** Distribution of UMI counts per sample. **(g)** Distribution of gene counts per sample. **(h)** Distribution of nuclei count per sample. **(i)** Distribution of nuclei count per donor. **(j)** Treemap visualization of the unified taxonomy representing the relative proportion and the hierarchy of cell types.

**Supplementary Fig. 2**. Comprehensive demographic and extended overview of the PsychAD cohort. This figure provides a detailed breakdown of the demographic, diagnostic, and genotypic characteristics of donors from three brain sources (HBCC, MSSM, and RADC). **(a)** Bar plots showing the number of donors by brain source, stratified by sex (male and female) and diagnosis (0: no diagnosis, 1: single diagnosis, 2+: multiple diagnoses). **(b)** Percentage distribution of donors by brain source, stratified by number of diagnoses (0, 1, and 2+). **(c)** Percentage distribution of diagnoses (0, 1, and 2+) by sex (male and female), highlighting diagnostic differences between sexes. **(d)** Box plots of age distribution by diagnosis category (0, 1, and 2+), illustrating the relationship between age and diagnostic burden. **(e)** Histogram of age distribution by brain source, showing the variation in donor ages for HBCC, MSSM, and RADC cohorts. **(f)** Age distribution by sex (male and female) across all donors. **(g)** Distribution of genetic ancestry across the three brain sources; AFR (African), AMR (Admixed American), EAS_SAS (East and South Asian), EUR (European), and Unknown. The number of donors in each category is indicated. **(h)** Distribution of genetic ancestry by sex (male and female), with percentages displayed for each genetic ancestry category. **(i)** Percentage of donors with available genotypic data per brain source.

#### **Supplementary Fig. 3**. **(a)** Cell type composition from each brain source using class- and subclass-level cellular taxonomy. **(b)** Cell type composition from each brain source using subtype-level cellular taxonomy. **(c)** Evaluation of PsychAD subtype-level annotation using two independent published datasets. Barplot showing the adjusted Local Inverse Simpson’s Index (LISI) scores^113^ for PsychAD subtype, ROSMAP^1^ celltype, and SEA-AD^2^ supertype annotations. LISI quantifies the local label separability of cell groupings based on annotated cell types or subtypes. To account for the inflation of LISI values in datasets with more fine-grained annotations, mean LISI was normalized by the natural logarithm of the number of clusters (adjusted LISI = mean LISI divided by log of the number of clusters). Lower adjusted LISI scores indicate greater local mixing relative to annotation granularity, suggesting better integration or less artificial separation. PsychAD subtype exhibited the lowest adjusted LISI, followed by ROSMAP celltype and SEA-AD supertype. Comparison of subclass-level cellular taxonomy with **(d)** Ma *et al.* 2022^10^ and **(e)** SEA-AD study^2^. **(f)** Comparison of subtype-level cellular taxonomy with Mathys *et al.* 2023^1^. **(g)** UMAP clustering of EN lineage, focusing on the diversity of 10 subclasses of excitatory neurons. **(h)** UMAP clustering of IN lineage, focusing on the diversity of 7 subclasses of inhibitory neurons. **(i)** Expression and fraction of marker gene expression in 27 subclasses. Expression z-scaled and averaged across nuclei. **(j)** Disease enrichment scores based on scDRS (disease risk scoring) using GWAS studies for AD^129^, PD^132^, SCZ^137^, and BD^138^.

**Supplementary Fig. 4**. Validation of cellular taxonomy using spatial transcriptomics. **(a)-(d)** Processing and QC of Xenium in situ spatial transcriptomics data. **(a)** Example image to demonstrate nuclei segmentation. The DAPI signal is shown in blue, and boundaries of segmented nuclei are shown as red outlines. Scale bar 20 µm. **(b)** Distribution of transcript counts per nucleus across all samples. The chosen threshold (30 counts) is shown as a red dotted line for reference. **(c)** UMAP of all Xenium nuclei containing >30 transcripts. Clusters were assigned “stable” or “ambiguous” labels based on how many of their nuclei were mapped to the same class using scANVI (**Methods**). **(d)** The same UMAP as (c), after removing ambiguous clusters and nuclei (mismatch between nuclei cluster and scANVI labels). Nuclei are colored based on their assigned class. **(e)** Comparison of nuclei density of IN subclasses by cortical layers (**Methods**). Note that y axes are in units of 1/μm^2^ scaled with a factor of 1e-5 (equivalent to 10 nuclei per mm^2^). Paired-sample Wilcoxon rank sum testing between adjacent cortical layers. Asterisk denotes P value < 0.05. **(f)** Comparison of nuclei density of IN subtypes by cortical layer for IN subtypes, similarly to the subclass-level analysis in (e). **(g-h)** Concordance of snRNA-seq and Xenium **(g)** EN and **(h)** IN subtypes. Pearson correlation between each pair of subtypes between Xenium (rows) and the RADC cohort snRNA-seq (columns) at pseudo-bulk level (see **Methods**). Adjacent box-plots compare the diagonal and off-diagonal elements. **(i)** Example spatial distribution of IN subclasses. A single tissue section measured with Xenium in situ spatial transcriptomics data. **(j)** Example spatial distribution of EN subtypes. **(k)** Example spatial distribution for SST-expressing IN subtypes. Coarse cortical layer structure is shown in greyscale.

**Supplementary Fig. 5**. Statistical power and effect size considerations in single-cell analyses. **(a)** Power to identify changes in cellular composition. Total number of cells per individual is shown on the x-axis and change in composition (i.e. delta) is shown by colored lines. Numbers in grey panels indicate sample size in each disease class, i.e. “25” indicates 25 cases and 25 controls. **(b)** Power to identify changes in gene expression. Standardized effect size is shown in Cohen’s d. Power calculations use the approach of Mandric et al.^178^ to model the noise in single cell assays using the squared correlation between measured and true gene expression. Numbers in grey panels indicate sample size in each disease class, as in panel a. **(c-d)** Variation in power across cell types for analysis of AD versus controls. Each point represents a cell type and with the number of expressed genes and individuals passing filters for cell and read counts. The size of the points indicates **(c)** Number of DEGs and **(d)** Mean absolute effect size of genes that pass FDR 5% cutoff. Each point is a cell subclass colored as in the main text. **(e)** Negative association between number of DEGs and mean absolute value of estimated effect size. Each point is a cell subclass colored as in the main text. **(f-g)** Estimated effect size for AD vs control comparison. **(f)** Estimated effect size for all genes. **(g)** Absolute estimated effect size for DEGs. **(h)** Relationship between the percentage of variation explained by mean expression (log_2_ counts per million). Rows are stratified by model coefficients; brain donors (BrainDonor) and cell types (CellTypes). Gene expression variance explained by these variables is computed for all subclasses (as shown in Fig. 3a) and three superclasses (as shown in Supplementary Fig. 6j), namely, Excitatory Neurons (EN), Inhibitory Neurons (IN), and Non-Neurons (NonNeuron). For example, the variance of superclass “EN” is computed across all EN subclasses. The same is true for “IN”. “NonNeuron” comprises all subclasses but EN and IN subclasses.

**Supplementary Fig. 6**. **(a)** Functional enrichment of top variable genes identified by variance partition analysis. For each covariate, genes in the top 10% quantile were selected for functional enrichment using Reactome^179^ and housekeeping gene sets from previous literature. HSIAO_HOUSEKEEPING_GENES: housekeeping genes identified as expressed across 19 normal tissues^176^, HOUNKPE_HOUSEKEEPING_GENES: 1,130 human and mouse housekeeping genes^177^. **(b)** Gene with the highest variation across age. **(c)** Gene with the highest variation across genetic ancestry. **(d)** Gene with the highest variation across sex. **(e)** Gene with the highest variation across brain sources. **(f)** Gene with the highest variation across diagnosis. **(g)** Normalized expression of ARL17B gene within L2-3 IT excitatory neuron subclass stratified by genetic ancestry. **(h)** The logistic regression coefficients for PD risk using the haplotype of MAPT locus. **(i)** Differentially expressed genes by dosage of H1 allele using additive effects. Intrachromosomal effects are genes in the same chromosome as MAPT locus (17q21). Otherwise, interchromosomal effects. All genes shown have FDR < 0.05. **(j)** Variance partition analysis within three superclasses, namely, Excitatory Neurons (EN), Inhibitory Neurons (IN), and Non-Neurons (Non-Neuron). **(k)** Sensitivity analyses showing the robustness of variance explained estimates. Spearman correlation of variance explained estimates between all subclasses model (All) vs. EN, IN, and Non-Neuron superclass models. **(l)** Enrichment of constrained genes measured by either the gnomAD^42^ Loss-of-function Observed/Expected Upper-bound Fraction (LOEUF) score < 0.6 or GeneBayes^43^ S_het_ > 1e-3. Hypergeometric test with P value ≤ 0.05 *, ≤ 0.01 **, ≤ 0.001 ***. **(m)** Functional enrichment of genes with low donor variation (bottom 10% quantile; BrainDonor Low), high donor variation (top 10% quantile; BrainDonor High), and constrained genes defined by gnomAD^42^ pLoF (LOEUF < 0.6) or GeneBayes^43^ (S_het_ > 1e-3).

#### **Supplementary Fig. 7.** Cross-disorder variation of cell type composition comparing eight different NDDs and NPDs against common neurotypical controls. **(a)** Canonical Correlation Analysis (CCA) matrix showing the relationships between various clinical and demographic variables for covariate selection. The color intensity indicates the strength of the correlation, with red representing positive correlations and blue negative correlations. **(b)** Variance partition of cell type composition, displaying the variance captured by different covariates in the formula. **(c)** Bar plot depicting the absolute logFC values of the eight different NDDs and NPDs across the cell types. **(d)** Variation in cell type composition for each subtype in eight different diseases. NDDs and NPDs indicate meta-analysis using broad disease categories. Color intensity indicates effect size and dot size reflects the statistical significance of correlations. **(e-f)** Compositional variation of VLMC subclass for eight different diseases and brain sources. T-test with P value ≤ 0.05 *, ≤ 0.01 **, ≤ 0.001 ***, ≤ 0.0001 ****. Using **(e)** fraction or **(f)** centered log ratio (CLR).

#### **Supplementary Fig. 8**. Cross-disorder shared and disease-specific gene expression signature. **(a)** Heatmap of pairwise Spearman correlation between diseases using cross-disorder shared genes (squares below the diagonal) and disease-specific genes (squares above the diagonal). Wilcoxon rank-sum test between two gene groups. **(b)** Extended **Fig. 5a** with all 27 subclasses and without reduction in similar GO terms. **(c)** Extended **Fig. 5c** with pathways implicated by shared signatures from AD, DLBD, Vas, and PD. **(d)** Correlation between shared heritability and transcriptome similarity per cell subclass. **(e)** Enrichment of genes carrying a high rare-variant burden. Hypergeometric tests were performed on genes with cross-disorder shared effects and genes with disease-specific effects for each subclass. Rare-variant association results were downloaded from Genebass^180^ (https://app.genebass.org/). For gene-based burden testing, we utilized the results from the Sequence Kernel Association Test - Optimal (SKAT-O), which utilized both mean and variance of the variant effects. Only predicted loss-of-function (LoF) variants for rare-variant association testing were considered. We defined a high rare-variant burden as genes with genome-wide significance (SKAT-O P value < 2.5 x 10-7). GeneRatio is the fraction of genes-of-interest (either shared or disease-specific genes) with a high rare-variant burden. **(f)** Distribution of the Spearman correlation coefficients from 10,000 synthetic datasets. Synthetic values were drawn from normal distribution centered at each estimate with the corresponding standard error. The Spearman correlation between the sampled transcriptome similarity and shared heritability was computed for each of 10,000 synthetic datasets. Using a normal approximation of the rho statistic, we can reject the null hypothesis that rho is less than zero with P value 1.27e-4.

**Supplementary Fig. 9**. Compositional variation across AD neuropathology. **(a)** Extended data figure of **Fig. 6a**. Compositional variation analysis using normal aging, different measures of AD pathology (binary AD diagnosis (dx_AD), CERAD score, Braak staging, and ordinal dementia scale), and three categories of NPS within AD. **(b)** Age distribution of AD cases with or without three NPS categories. **(c)** Comparison of compositional variation between MSSM and RADC for WeightLoss phenotype. Due to the lack of exact WeightLoss/PMA phenotype in RADC, WeightLoss was binarized from body mass index (BMI) within AD cases. **(d)** Fixed-effect meta-analysis between MSSM WeightLoss/PMA and RADC WeightLoss. **(e)** Validation of compositional changes using RNAscope (see **Methods**). Cell counts were quantified based on specific markers - CUX2 (L2-3 IT neurons), SST (somatostatin interneurons), and BMP5 (VLMCs). Cell fractions were transformed using the centered log-ratio (CLR) method. Statistical differences between AD cases (n=5) and controls (n=5) were assessed using the Mann–Whitney–Wilcoxon test with P value ≤ 0.05 *, ≤ 0.01 **, ≤ 0.001 ***. Total n=48 ROIs were examined from two sections per donor and 2-3 ROIs imaged per section. Each ROI included 200 to 500 fields of views of 40X objective. **(f)** Representative immunofluorescence images comparing AD and neurotypical control dissections.

**Supplementary Fig. 10**. Comparison of compositional variation analysis with previous studies - DLPFC snRNA-seq data from **(a)** ROSMAP (Mathys et al. 2023)^1^ and **(b)** SEA-AD (Gabitto et al. 2024)^2^. **(c)** Comparison of compositional variation analysis using the MERFISH spatial transcriptomics dataset included in the SEA-AD study^2^. Spearman correlation between this study (x-axis) and previous studies (y-axis) using 3 different measures of AD pathology (CERAD score, Braak staging, and ordinal Dementia scale). ROSMAP used clinical diagnoses of AD, MCI, and NCI, while SEA-AD used clinical diagnosis of dementia as a measure of cognitive decline. Further details regarding compositional analysis of the MERFISH data and joint statistical testing can be found in **Methods**.

#### **Supplementary Fig. 11**. Transcriptomic changes across AD neuropathology. **(a)** Distribution of disease effect sizes by cell class, stratified by the direction of effect (color, red: up, blue: down). **(b)** Extended data figure for **Fig. 6d**. DEGs in AD phenotypes. Meta-analysis between brain banks. Top genes with FDR < 0.01 and effect size ≥ 0.35. **(c)** Functional enrichment analysis of DEGs by subclass using GO BP pathways. Hypergeometric test with FDR ≤ 0.01 shown.

#### **Supplementary Fig. 12.** **(a)** Comparison of differential expression signatures with previous studies – ROSMAP (Mathys et al. 2023)^1^ and SEA-AD (Gabitto et al. 2024)^2^. Pairwise Spearman correlation between PsychAD subclass and ROSMAP Major Cell Type. CERAD as a measure of disease severity. Asterisks indicate the best matching cell type based on **Supplementary Table 11**. **(b)** Pairwise Spearman correlation between PsychAD subclass and SEA-AD subclass. CERAD as a measure of disease severity. Asterisks indicate the best matching cell type based on **Supplementary Table 11**. **(c)** Comparison of various disease signatures (CERAD, Braak, Dementia) between matched (diagonal, asterisks) and unmatched (off-diagonal) cell types. Mann–Whitney U test. P value ≤ 1.0e-4 ****. **(d)** Comparison of differentially expressed genes between PsychAD Microglia and ROSMAP Mic using CERAD as a measure of disease severity. Spearman correlation (rho) shown. **(e)** Comparison of differentially expressed genes between PsychAD Microglia and SEA-AD Microglia-PVM using CERAD as a measure of disease severity. Spearman correlation (rho) shown.

**Supplementary Fig. 13**. **(a)** Estimated effect sizes from differential expression analysis of AD vs control. Comparison of AD models excluding (x-axis) vs including (y-axis) APOE genotype as a covariate. Each point represents a gene. Both analyses included the following covariates: age, sex, PMI, log(n_genes), percent_mito, mito_genes, ribo_genes, and mito_ribo. **(b)** Correlation between PRS scores calculated including (x-axis) vs excluding (y-axis) APOE locus.

**Supplementary Fig. 14.** The effect of different training methods on model prediction correlations and accuracy for the Immune cell class. **(a)** Correlation between Braak (x-axis) and dementia (y-axis) model predictions at the cell-level (left panel) and donor-averaged level (right panel) for models in which all cells are equally sampled during training. To reduce the figure size, only 1 out of 20 cells shown in the left panel. **(b)** Same as (a), except for models for which all combinations of Braak stage and dementia status are equally sampled during training. **(c)** Accuracy at the cell-level (left: Braak Pearson R, right: Dementia classification accuracy) for models in which all cells are equally sampled during training (blue), and for which all combinations of Braak stage and dementia status are equally sampled during training (orange). **(d)** Same as (c), except showing accuracy at the donor-averaged level.

#### **Supplementary Fig. 15.** Comparing neural network model predictions to PLS regression. **(a)** Dementia classification accuracy (green) and Pearson correlation between actual and predicted Braak values (green) for our network model and PLS regression. Results shown for the Immune cell class shown. **(b)** Scatter plot comparing the donor-averaged Braak predictions (x-axis) and dementia predictions (y-axis) for our network model (left) and PLS regression (right). The Pearson correlation is shown for both models. **(c)** Scatter plot showing the disease pseudotime Zenith z-score for our network model (x-axis) and PLS (y-axis). The left panel shows pathways with similar trends between Braak and dementia (i.e. expression increases with Braak and is associated with greater dementia or decreases with Braak and is associated with less dementia). The right panel shows pathways with diverging trends (i.e. expression increases with Braak and is associated with less dementia or decreases with Braak and is associated with greater dementia). Red (blue) dots indicate whether the z-score was positive (negative). The mean difference and Wilcoxon P value comparing the network model and PLS z-scores is shown. Results are shown for changes in the early disease state. All significant pathways (FDR < 0.05 for either model, either for changes in disease (i.e. Braak) or resilience), are included. **(d)** The top ten pathways with the greatest difference between the z-scores from our network model (blue) and the PLS model (orange). White asterisks indicate whether the pathway was significant (FDR < 0.05).

#### **Supplementary Fig. 16.** Identifying trajectory transition points. **(a)** Smoothed gene trajectories of all coding genes across all eight cell classes were concatenated and projected onto the first three principal components. Each dot indicates a donor, and the hue indicates the disease pseudotime prediction averaged across all cell classes. The cyan circle indicates the "preliminary" transition point, and the magenta circle indicates the "early-to-late" transition point. **(b)** The explained variance ratio of the first three principal components.

#### **Supplementary Fig. 17.** Calculating the nonlinearity index. **(a)** For each cell class, we fit each gene trajectory along the disease pseudotime axis with a linear model, and with a piecewise model consisting of two linear functions. For each cell class, we chose the transition time between the two linear functions of the piecewise model by determining the transition that resulted in the greatest explained variance. The explained variance of the single linear model is shown in blue, and the explained variance of the piecewise model in orange. **(b)** The nonlinearity index is the difference between the explained variance of the piecewise and single linear models.

#### **Supplementary Fig. 18.** Extended data for **Fig. 7e**. Expanded list of all top GO BP pathways. Upper left triangles indicate the early phases, and lower right triangles indicate the late phases of the AD trajectory predicted by Braak and dementia resilience. Hue indicates the z-score, and stars indicate FDR < 0.05.

#### **Supplementary Fig. 19.** Pathway enrichment using the top GO CC pathways. Upper left triangles indicate the early phases, and lower right triangles indicate the late phases of the AD trajectory predicted by Braak and dementia resilience. Hue indicates the z-score, and stars indicate FDR < 0.05.

#### **Supplementary Fig. 20.** Pathway enrichment using the top GO MF pathways. Upper left triangles indicate the early phases, and lower right triangles indicate the late phases of the AD trajectory predicted by Braak and dementia resilience. Hue indicates the z-score, and stars indicate FDR < 0.05.

**Supplementary Fig. 21. Visualization of protective and damaging pathways. (a)** Extracted from Fig 7e., highlighting damaging synaptic pathways associated with the IN cell class. **(b)** Hexbin plots showing the mean pathway enrichment scores of four different synaptic pathways as a function of predicted Braak stage (x-axis) and predicted dementia status (y-axis). The color of each hexagon indicates the mean pathway enrichment from donors with similar disease pseudotime and predicted dementia scores. Enrichment scores were calculated for each donor by calculating the donor-averaged expression, subtracting the mean expression across all donors, and then calculating the Zenith z-score on the resultant expression values. Enrichment of four synaptic pathways as a function of the disease pseudotime (x-axis) and the predicted Dementia score (y-axis). The color at each location indicates the mean pathway enrichment (Zenith z-score). **(c)** Same as (a), except highlighting protective pathways of the Immune cell class. **(d)** Same as (b), except for the protective immune pathways.

#### **Supplementary Fig. 22.** Pathway enrichment across all cell classes using only pathways enriched in AD risk genes. Pathways were only included if the AD GWAS^129^ z-scores of the genes in the pathway were significantly greater (p < 0.01, one-sided t-test) than all other coding genes not in the pathway (Methods). Text colored by the four trajectory modules as in Fig. 7g (blue: early increasing, orange: early decreasing, green: late increasing, red: late decreasing). Hue indicates the number of genes in the pathway. The dashed vertical lines indicate FDR = 0.05.

#### **Supplementary Fig. 23.** Trajectories for genes in the negative regulation of peptidyl-threonine GO BP pathway for the neuron cell classes. **(a)** Trajectories across disease pseudotime for all genes in the pathway for both the EN (left) and IN (right) cell classes. Gene expression was normalized so that values are zero at the start of the disease pseudotime trajectory. **(b)** The percentile of each gene in the pathway ranked according to its early slope. 100% indicates the gene with the most negative slope among all 17,265 coding genes, while 0% represents the gene with the greatest positive slope. Results shown for both the EN and IN cell classes. The AD GWAS^61,129^ P value of each gene is shown.

**Supplementary Fig. 24.** Enrichment of heritability estimates (MAGMA) results across all cell classes; three NDDs (shown in red) and three NPDs (shown in cyan) are shown. Similar to Fig. 7, the top 250 genes in each category are used to calculate the enrichment FDR. Full results are shown in Supplementary Table 8. Hue indicates the negative log10 FDR (capped at 5). The four columns to the left (early/late increasing/decreasing) refer to changes in disease pseudotime. The four columns to the right (early/late damaging/protective) refer to resilience. FDR ≤ 0.05 *, ≤ 0.01 **, ≤ 0.001 ***.

**Supplementary Fig. 25.** Dynamics of the immune response using pathways that are either (1) significantly enriched for Alzheimer’s disease (AD) risk genes, or (2) among the key immune and metabolic pathways highlighted previously in Fig. 7e. **(a)** Change in pathway gene expression calculated using a sliding window across disease pseudotime. Hue indicates Zenith z-score. Vertical lines (indicated by preliminary and early-late) are the transition points identified in Fig. 7c. **(b)** Similar to (a), but only the immune specific pathways. The biphasic response suggests two distinct phases of the immune response.

**Supplementary Fig. 26.** Trajectories for genes involved in lipid droplet accumulation and efflux for the immune cell class. **(a)** Trajectories across disease pseudotime for four genes associated with a microglia lipid-droplet phenotype^61^. Gene expression was normalized so that values are zero at the start of the disease pseudotime trajectory. **(b)** The percentile of the four genes ranked according to its early and late slopes. 100% indicates the gene with the greatest slope among all 17,265 coding genes, while 0% represents the gene with the most negative slope across all coding genes. The AD GWAS^61,129^ P value of each gene is shown. **(c-d)** Similar to (a-b), except showing all genes in the GO BP *phospholipid efflux* pathway.

**Supplementary Fig. 27** Comparing model predictions to existing single cell trajectory inference methods. **(a)** UMAP projections of the microglia cell subtype with the color at each location indicating the mean (from left to right) actual Braak values, the disease pseudotime neural network model predictions, Monocle3 and Palantir pseudotime values. **(b)** Distribution of (from left to right) the disease pseudotime neural network model predictions, Monocle3 pseudotime values and Palantir pseudotime values. The Pearson correlation coefficient is shown for each method.

#### **Supplementary Fig. 28.** Comparing smoothed and unsmoothed approaches to measuring changes in gene expression and resilience. Each circle represents a GO BP pathway, in which the x-axis indicates the Zenith z-score using the smoothed approach, and the y-axis indicates the z-score using the unsmoothed approach (see Methods). Only pathways for which the Zenith FDR calculated using the smoothed approach was below 0.05 were included. The columns, from left-to-right, show the early and late changes in gene expression with disease pseudotime, and the early and late resilience scores. For disease pseudotime, red circles indicate increasing pathways, and blue circles indicate decreasing pathways. For resilience, green circles indicate protective pathways, and magenta circles indicate damaging pathways. The Pearson correlation (R), measured separately for increasing and decreasing, or protective and damaging, pathways is shown in each panel. Each row shows the results for one cell class.

#### **Supplementary Fig. 29**. Neural network model training curves across the cell classes. (a-h) Training curves for each cell class. The left panel shows the cross-validated Pearson correlation between actual and predicted Braak values. Predictions are averaged at the donor-level. The middle panel shows the dementia classification accuracy, and the right panel shows the correlation between the donor-averaged Braak and dementia model predictions. The x-axis shows the number of training epochs.

#### **Supplementary Fig. 30**. Nuclei were purified using Fluorescently activated nuclei sorting (FANS). Following tissue homogenization, nuclei were labelled with 7-Aminoactinomycin D (7-aad). Cell debris was identified and excluded via forward scatter-area/side scatter-area (FSC-A/SSC-A) gating, while singlets were selected using a sequential combination of forward scatter-area (FSC-A)/ FSC-width (FSC-W) gating, followed by side scatter-area (SSC-A)/ SSC-width (SSC-W) gating. 7-aad positive nuclei were collected and subjected to snRNA-seq.

# Glossary of Abbreviations

DLPFC: Dorsolateral prefrontal cortex

MSSM: Mount Sinai NIH Neurobiobank

HBCC: Human Brain Collection Core

RADC: Rush Alzheimer’s Disease Center

NDD: Neurodegenerative diseases

NPD: Neuropsychiatric diseases

NPS: Neuropsychiatric symptoms

PMA: Psychomotor agitation

PMR: Psychomotor retardation

AD: Alzheimer’s disease

DLBD: Diffuse Lewy body disease

Vas: Vascular dementia

PD: Parkinson’s disease

Tau: Tauopathy

FTD: Frontotemporal dementia

SCZ: Schizophrenia

BD: Bipolar disorder

MS: Multiple sclerosis

Epilepsy: Epilepsy focal

ALS: Amyotrophic lateral sclerosis

MDD: Major depressive disorder

ASD: Autism spectrum disorder

ADHD: Attention deficit hyperactivity disorder

Education: Educational attainment

IQ: Intelligence

OCD: Obsessive compulsive disorder

Tourettes: Tourette Syndrome

T2D: Type 2 diabetes mellitus

RArthritis: Rheumatoid arthritis

IBD: Inflammatory bowel disease

UC: Ulcerative colitis

NFT: Neurofibrillary tangle

MCI: Mild cognitive impairment

PMI: Postmortem interval

AFR: African

AMR: Ad Mixed American

EAS: East Asian

SAS: South Asian

EUR: European

EN: Glutamatergic excitatory neuron

EN_L2_3_IT: Layer 2-3 Intratelencephalic Excitatory Neuron

EN_L3_5_IT_1: Layer 3-5 Intratelencephalic Excitatory Neuron 1

EN_L3_5_IT_2: Layer 3-5 Intratelencephalic Excitatory Neuron 2

EN_L3_5_IT_3: Layer 3-5 Intratelencephalic Excitatory Neuron 3

EN_L5_6_NP: Layer 5-6 Near-Projecting Excitatory Neuron

EN_L5_ET: Layer 5 Extratelencephalic Excitatory Neuron

EN_L6B: Layer 6B Excitatory Neuron

EN_L6_CT: Layer 6 Corticothalamic Excitatory Neuron

EN_L6_IT_1: Layer 6 Intratelencephalic Excitatory Neuron 1

EN_L6_IT_2: Layer 6 Intratelencephalic Excitatory Neuron 2

IT: intra-telencephalic

ET: extra-telencephalic

NP: near projecting

CT: corticothalamic

IN: GABAergic inhibitory neuron

IN_ADARB2: ADARB2 Inhibitory Neuron

IN_LAMP5_LHX6: Ivy cell (LAMP5 LHX6 Inhibitory Neuron)

IN_LAMP5_RELN: Neurogliaform cell (LAMP5 RELN Inhibitory Neuron)

IN_PVALB: Basket Cell (PVALB Inhibitory Neuron)

IN_PVALB_CHC: Chandelier Cell (PVALB CHC Inhibitory Neuron)

IN_SST: Martinotti and Non-Martinotti cell (SST Inhibitory Neuron)

IN_VIP: VIP Inhibitory Neuron

Astro: Astrocyte

OPC: Oligodendrocyte Progenitor Cell

Oligo: Oligodendrocyte

Micro: Microglia

PVM: Perivascular Macrophage

Adaptive: Adaptive Immune Cell

VLMC: Vascular Leptomeningeal Cell

SMC: Smooth Muscle Cell

PC: Pericyte

Endo: Endothelial Cell

SRP: Signal-recognition particle

Pos: Positive

Neg: Negative

Reg: Regulation

Resp: Response

FDR: False discovery rate

CCA: Canonical Correlation Analysis

HVG: Highly variable genes

kNN: k-nearest-neighbor

UMAP: Uniform Manifold Approximation and Projection

GO: Gene Ontology

BP: Biological Process

MF: Molecular Function

CC: Cellular Component
