# Supplementary material for: Single-cell atlas of transcriptomic vulnerability across brain disorders
Source: Nature. 2026 Sep 23;657(8133):988–1002. doi: 10.1038/s41586-025-09573-z (PMC13600946; doi:10.1038/s41586-025-09573-z)
Supplement: Supplementary file 4 [file 41586_2025_9573_MOESM3_ESM.zip › 2024-07-15352A-s3/supp-table-legends.docx]

SUPPLEMENTARY INFORMATION

# Supplementary Tables

**Supplementary Table 1**. Clinical and technical metadata of 1,494 donors in the PsychAD cohort. Metadata includes binary contrasts and continuous variables used in the study. Columns named crossDis_AD, crossDis_SCZ, crossDis_DLBD, crossDis_Vas, crossDis_BD, crossDis_Tau, crossDis_PD, and crossDis_FTD describe donors used in cross-disorder analysis depicted in Fig. 1c as binary contrasts. The value 1 indicates cases while 0 indicates disease-free controls used for the contrast. Columns named Tier1_crossDis, Tier2_AD, and Tier3_NPS indicate individuals included in 3-tier analyses.

**Supplementary Table 2**. Hierarchical cellular taxonomy of the PsychAD snRNA-seq data. Metadata including parent-child relationships, color hex codes, and number of nuclei per subtype.

**Supplementary Table 3**. Compositional variation analysis across 8 cross-disorder traits.

**Supplementary Table 4**. Differentially expressed genes across 8 cross-disorder traits. Columns named crossDis_PP and crossDis_cat indicate posterior probability of each gene having cross-disorder shared effect and category of shared or non-shared used for the analysis.

**Supplementary Table 5**. Compositional variation analysis across AD phenotypes.

**Supplementary Table 6**. Differentially expressed genes across AD phenotypes.

**Supplementary Table 7**. The top 50 GO BP pathways for each cell class for early/late increases/decreases in disease pseudotime, and for early/late dementia resilience

**Supplementary Table 8**. Gene-set enrichment analysis using GWAS summary data for each cell class. GWAS scores are calculated using the top 250 coding genes with the greatest slopes measured relative to early/late increases/decreases in disease pseudotime, and the top 250 early/late protective/damaging coding genes.

**Supplementary Table 9**. The top 250 coding genes with the greatest slopes measured relative to early/late increases/decreases in disease pseudotime, and the top 250 early/late protective/damaging coding genes.

**Supplementary Table 10**. List of gene markers used from Xenium human brain custom panel.

**Supplementary Table 11**. Comparison of PsychAD cellular taxonomy to published snRNA-seq datasets (SEA-AD Gabitto 2024 and ROSMAP Mathys 2023).

**Supplementary Table 12**. The QC statistics of median gene and UMI counts, median percent mitochondrial genes, and cell counts, separated by 2,924 single-cell libraries utilized, before and after the removal of low-quality cells.

**Supplementary Table 13**. Causal mediation analysis involving PRS, cell type composition, pathology, and dementia status. Estimates of direct and mediating effects and their statistical significance.

# Glossary of Abbreviations

DLPFC: Dorsolateral prefrontal cortex

MSSM: Mount Sinai NIH Neurobiobank

HBCC: Human Brain Collection Core

RADC: Rush Alzheimer’s Disease Center

NDD: Neurodegenerative diseases

NPD: Neuropsychiatric diseases

NPS: Neuropsychiatric symptoms

PMA: Psychomotor agitation

PMR: Psychomotor retardation

AD: Alzheimer’s disease

DLBD: Diffuse Lewy body disease

Vas: Vascular dementia

PD: Parkinson’s disease

Tau: Tauopathy

FTD: Frontotemporal dementia

SCZ: Schizophrenia

BD: Bipolar disorder

MS: Multiple sclerosis

Epilepsy: Epilepsy focal

ALS: Amyotrophic lateral sclerosis

MDD: Major depressive disorder

ASD: Autism spectrum disorder

ADHD: Attention deficit hyperactivity disorder

Education: Educational attainment

IQ: Intelligence

OCD: Obsessive compulsive disorder

Tourettes: Tourette Syndrome

T2D: Type 2 diabetes mellitus

RArthritis: Rheumatoid arthritis

IBD: Inflammatory bowel disease

UC: Ulcerative colitis

NFT: Neurofibrillary tangle

MCI: Mild cognitive impairment

PMI: Postmortem interval

AFR: African

AMR: Ad Mixed American

EAS: East Asian

SAS: South Asian

EUR: European

EN: Glutamatergic excitatory neuron

EN_L2_3_IT: Layer 2-3 Intratelencephalic Excitatory Neuron

EN_L3_5_IT_1: Layer 3-5 Intratelencephalic Excitatory Neuron 1

EN_L3_5_IT_2: Layer 3-5 Intratelencephalic Excitatory Neuron 2

EN_L3_5_IT_3: Layer 3-5 Intratelencephalic Excitatory Neuron 3

EN_L5_6_NP: Layer 5-6 Near-Projecting Excitatory Neuron

EN_L5_ET: Layer 5 Extratelencephalic Excitatory Neuron

EN_L6B: Layer 6B Excitatory Neuron

EN_L6_CT: Layer 6 Corticothalamic Excitatory Neuron

EN_L6_IT_1: Layer 6 Intratelencephalic Excitatory Neuron 1

EN_L6_IT_2: Layer 6 Intratelencephalic Excitatory Neuron 2

IT: intra-telencephalic

ET: extra-telencephalic

NP: near projecting

CT: corticothalamic

IN: GABAergic inhibitory neuron

IN_ADARB2: ADARB2 Inhibitory Neuron

IN_LAMP5_LHX6: Ivy cell (LAMP5 LHX6 Inhibitory Neuron)

IN_LAMP5_RELN: Neurogliaform cell (LAMP5 RELN Inhibitory Neuron)

IN_PVALB: Basket Cell (PVALB Inhibitory Neuron)

IN_PVALB_CHC: Chandelier Cell (PVALB CHC Inhibitory Neuron)

IN_SST: Martinotti and Non-Martinotti cell (SST Inhibitory Neuron)

IN_VIP: VIP Inhibitory Neuron

Astro: Astrocyte

OPC: Oligodendrocyte Progenitor Cell

Oligo: Oligodendrocyte

Micro: Microglia

PVM: Perivascular Macrophage

Adaptive: Adaptive Immune Cell

VLMC: Vascular Leptomeningeal Cell

SMC: Smooth Muscle Cell

PC: Pericyte

Endo: Endothelial Cell

SRP: Signal-recognition particle

Pos: Positive

Neg: Negative

Reg: Regulation

Resp: Response

FDR: False discovery rate

CCA: Canonical Correlation Analysis

HVG: Highly variable genes

kNN: k-nearest-neighbor

UMAP: Uniform Manifold Approximation and Projection

GO: Gene Ontology

BP: Biological Process

MF: Molecular Function

CC: Cellular Component
